# Organotellurium Probes Enable One-step Single-cell Analysis of Post-translational Modification

**DOI:** 10.1021/jacs.5c19824

**Published:** 2026-02-17

**Authors:** Yuanzhe Chen, Kris Elbein, Sneha Venkatachalapathy, Ellen L. Lorimer, Andrea M. Sprague-Getsy, Shelby A. Auger, Zoë A. Maxwell, Mohammad Rashidian, James L. Hougland, Carol L. Williams, Edgar A. Arriaga, Mark D. Distefano

**Affiliations:** † Department of Chemistry, 5635 University of Minnesota-Twin Cities, Minneapolis, Minnesota 55455, United States; ‡ Department of Pharmacology and Toxicology, 5506 Medical College of Wisconsin, Milwaukee, Wisconsin 53226, United States; § Department of Chemistry, 2029 Syracuse University, Syracuse, New York 13244, United States; ∥ Department of Biology, Syracuse University, Syracuse, New York 13244, United States; ⊥ Department of Cancer Immunology and Virology, 1855 Dana-Farber Cancer Institute, Boston, Massachusetts 02115, United States

## Abstract

Protein prenylation is a widespread post-translational modification (PTM) that regulates membrane association and signaling; dysregulation of this process leads to a variety of diseases. Metabolic labeling with probes containing bioorthogonal functionality has revolutionized the study of many protein modifications, including prenylation. However, that approach requires two steps, including metabolic incorporation and subsequent bioorthogonal reaction to install chemical reporters. Here, we present the development and application of tellurium-containing isoprenoid analogues that can be incorporated through a single enzymatic step and enable the direct quantification of prenylation at the single-cell level by mass cytometry. This robust methodology was examined in a variety of cell lines and used to show that prenylation levels are perturbed in autophagy-deficient L6 cells, a model for certain features of aging. Modification of tellurium-labeled proteins through the oxidation-controlled strain-promoted tellurophene-alkyne cycloaddition reaction also enabled the identification of prenylation targets by chemical proteomics. This methodology bridges proteomic and multiplexed single-cell analyses, opening up promising avenues for exploring a variety of post-translational modifications.

## Graphical abstract

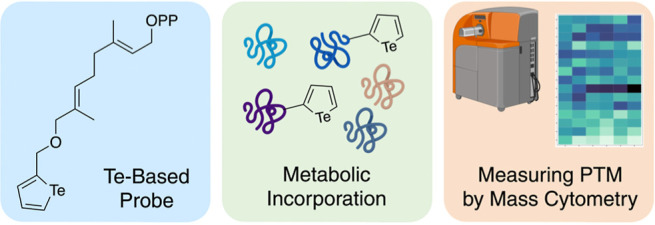

## Introduction

Protein prenylation is a post-translational modification in which a hydrophobic isoprenoid group, either a 15-carbon farnesyl or a 20-carbon geranylgeranyl moiety, is covalently attached to a specific cysteine residue near the C-terminus of substrate proteins, using farnesyl diphosphate (FPP) and/or geranylgeranyl diphosphate (GGPP) as natural isoprenoid donors (Figure 1A).^1–4^ This modification plays a crucial role in facilitating membrane association and modulating protein–protein interactions, thereby impacting numerous cellular processes, including signal transduction,^5^ intracellular transport,^6^ and cytoskeletal organization.^7^ Farnesyltransferase (FTase) and geranylgeranyltransferase type 1 (GGTase-I) are responsible for transferring single farnesyl or geranylgeranyl groups to proteins bearing a canonical “CaaX” motif (Figure 1B). A third enzyme, geranylgeranyltransferase type 2 (GGTase-II), also known as RabGGTase, operates in conjunction with Rab escort proteins (REPs) to sequentially attach two geranylgeranyl groups to Rab GTPases, which serve key regulatory functions in almost all membrane trafficking (Figure 1C). An additional enzyme, GGTase-III transfers a geranylgeranyl group onto a farnesylated precursor.^8^ Dysregulation of prenylation has been implicated in a wide range of diseases, including cancer,^9^ progeria,^10^ and aging^11,12^ As a result, targeting the prenylation process to disrupt membrane localization and concomitant protein signaling has emerged as a promising therapeutic strategy. Many oncogenic proteins, including members of the Ras superfamily, require prenylation for the downstream signaling. Farnesyltransferase inhibitors (FTIs) have been extensively studied as a therapeutic strategy for numerous diseases ranging from cancer to parasitic infections.^13,14^ In 2021, Lonafarnib was approved for the treatment of progeria, a premature aging disease, and Tipifarnib has received a fast-track designation from the FDA for head and neck squamous cell carcinoma.^15,16^

**Figure 1:**
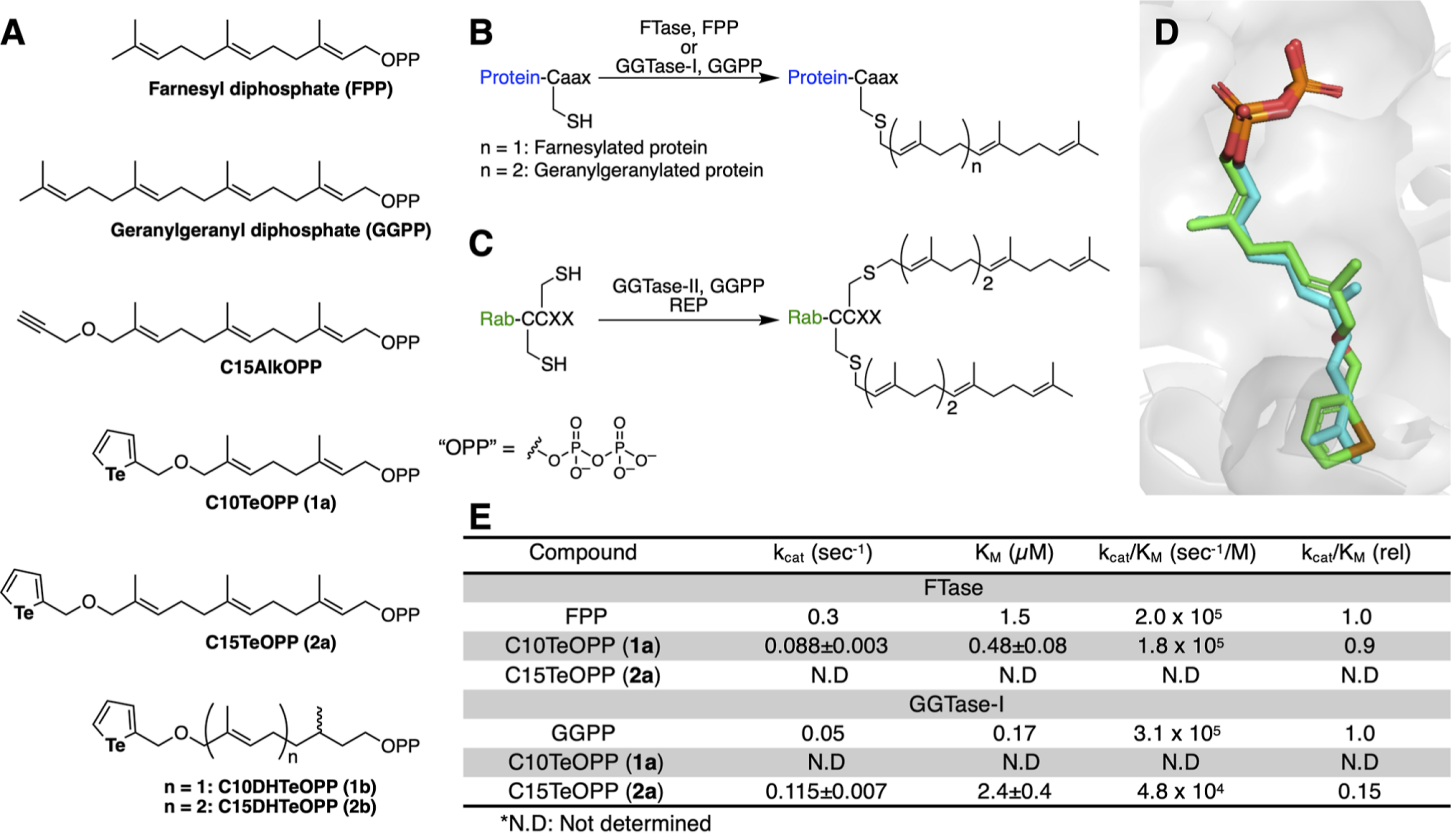
Reactions, isoprenoid substrates, and probes used to study protein prenylation. (A) Structure of the natural prenyl group donors farnesyl diphosphate (FPP) and geranylgeranyl diphosphate (GGPP), previously reported alkyne-functionalized probe C15AlkOPP, new tellurium-functionalized probes C10TeOPP (**1a**) and C15TeOPP (**2a**), and the corresponding dihydro probes C10DHTeOPP (**1b**) and C15DHTeOPP (**2b**). (B) Scheme of farnesylation and geranylgeranylation type 1 reactions catalyzed by FTase and GGTase-I, respectively. (C) Scheme of geranylgeranylation type II reaction catalyzed by GGTase-II in the presence of Rab escort protein (REP). (D) Computational modeling showing the docking of **1a** (C: green; Te: brown) into the FPP binding pocket of FTase (1JCR, gray) superimposed on the native substrate FPP (C: blue); for both **1a** and FPP, O: red; P: orange. (E) Kinetic parameters for **1a** and **2a** evaluated using FTase and GGTase-I, values for natural substrates FPP^35^ and GGPP^36^ were obtained from published works. For certain enzyme–substrate pairs, kinetic parameters could not be determined (N.D) due to slow reaction rates and lack of saturation resulting in poor curve fitting.

The advent of bioorthogonal chemistry has transformed the analysis of protein prenylation. In such methods, synthetic probes bearing small functional groups including azides^17,18^ and alkynes^19–22^ are metabolically incorporated into proteins *in cellulo* or even *in vivo*.^23,24^ Subsequent click reactions with biotin-based reagents followed by enrichment and quantitative proteomic experiments have enabled the identification and monitoring of specific prenylated proteins, while derivatization with fluorescent compounds has been used to measure global levels of prenylation and to image their location in cells and tissue samples. This latter approach is well suited for single-cell analysis via flow cytometry, as it can measure prenylation levels in individual cells within a population.^25^ Single-cell analysis is capable of detecting cell-to-cell variability, ensuring that the properties being investigated are not confounded by population heterogeneity.^26,27^

While flow cytometry is useful for single-cell studies, the number of cellular markers that can be examined in such experiments is generally limited to less than 10 due to spectral overlap of the fluorophores.^28^ Recently, mass cytometry methods have been developed to address this limitation.^29^ In this technique, antibodies functionalized with chelated metal ions in lieu of fluorophores are used for marker detection.^30^ Cells are nebulized and vaporized, and the resulting metal ions are detected using a time-of-flight mass spectrometer. Since such instruments have unit mass resolution, a much larger number of analytes/parameters can be detected, enabling more complex mixtures of cells and subpopulations to be analyzed. Previously, this method was used to measure prenylation levels by metabolic incorporation of an alkyne-containing isoprenoid analogue (C15AlkOPP, Figure 1A) followed by click reaction with an azide-modified chelator bearing a Tb ion.^31^ This two-step procedure was necessary, since the bulky nature of the substrate-chelator conjugate precludes direct enzymatic recognition and incorporation. Beyond this limitation, the two-step strategy introduces other notable drawbacks, including increased workflow complexity, nonspecific chemical tagging, and difficulty in detecting low-abundance targets.

To overcome those limitations and streamline the process, we turned our attention to tellurophene, a compact organotellurium heterocycle. Prior work by Bassan et al. demonstrated the use of a tellurophene-modified noncanonical amino acid that served as a phenylalanine surrogate to monitor protein synthesis using mass cytometry.^32^ Tellurophene offers excellent stability under cellular conditions and is well suited for detection via inductively coupled plasma time-of-flight mass spectrometry (CyTOF).^33,34^

In this work, we report the design and evaluation of two tellurophene-containing isoprenoids, C10TeOPP (**1a**) and C15TeOPP (**2a**, Figure 1A), which enable one-step labeling of farnesylated and geranylgeranylated proteins in living cells. These probes serve as effective FPP and GGPP surrogates with distinct substrate preferences based on their isoprenoid length, allowing for selective and orthogonal labeling of prenylated proteins. To serve as nonreactive controls, a new class of isoprenoid derivatives, denoted “dihydro probes”, was prepared (**1b** and **2b**, Figure 1A). Except for a single double bond, these latter compounds retain all structural elements found in their enzymatically reactive counterparts, **1a** and **2a**, but cannot be incorporated by prenyltransferases, allowing signal distinction arising from enzymatic incorporation and nonspecific cellular retention in mass cytometry experiments. The enzymatic compatibility of these tellurium-containing compounds was evaluated through steady-state kinetic assays and *in vitro* labeling of the peptides and purified proteins. Their utility in single-cell mass cytometry was established using acute myeloid leukemia-3 (AML-3) cells as the primary model to quantify prenylation at the cellular level. In addition, the recently reported tellurophene–bicyclononyne (BCN) reaction offered a selective approach to modify tellurophene-labeled proteins for in-gel fluorescence detection and protein identification via chemical proteomics. The mass cytometric analyses were then extended to additional cell lines to study probe performance in multiple biological contexts. To explore potential biological relevance, these new probes were applied in studies of autophagy-related gene 7 knockout (Atg7 KO) L6 cells, where reductions in prenylation signals, supported by chemical proteomics, were observed in models of decreased autophagic activity. Together, these studies demonstrated that tellurophene-modified isoprenoids provide a powerful one-step strategy to analyze protein prenylation in a multiplexed analysis, with applications in both basic biology and disease-related pathways. This approach should be readily transferable to the study of other post-translational modifications.

## Results and Discussion

### Design and Synthesis of Tellurophene-Functionalized Prenylation Probes

Previous works reported from our laboratory has employed isoprenoid diphosphates bearing terminal modifications.^36–41^ An ether linkage was typically used to attach the desired functionality owing to its synthetic accessibility, structural flexibility, and stability under cellular conditions. Similar considerations were employed here for the design of new Te-containing probes. To access the steric consequences of introducing tellurophene groups into isoprenoid structures, molecular volumes for a series of isoprenoid analogues were calculated and compared with the parent compounds farnesol (C_15_) and geranylgeraniol (C_20_) (Table S1). Relative to farnesol (three isoprene units; 208 Å^3^), an analogue comprised of two isoprene units and a propargyl ether (C10AlkOH)^42^ is 18.0 Å^3^ smaller, whereas the corresponding compound containing three isoprene units (C15AlkOH)^42^ is 45 Å^3^ larger. In contrast, C10TeOH (two isoprene units and a hydroxymethyl tellurophene, compound **18**) is only 18 Å^3^ larger than farnesol. Other analogues with bioorthogonal functionality, including C10NorOH^40^ (two isoprene units and a norbornene group) and C10TCOOH^43^ (two isoprene units and a cyclooctene group), are substantially larger, with volume increases of 34 and 44 Å^3^, respectively. Collectively, these comparisons indicate that a hydroxymethyl tellurophene group is a relatively small perturbation and closely approximates the size of a native isoprene unit. The hydrophobicity of these analogues was also analyzed by determining their ClogP values (Table S1). Those calculations revealed that C10TeOH (ClogP = 2.79) was slightly less polar than C10AlkOH (ClogP = 2.36) but more polar than farnesol (ClogP = 5.00), indicating that a hydroxymethyl tellurophene moiety confers increased polarity compared to a natural isoprene unit. A computational docking study was then performed with C10TeOPP (**1a**) and FTase, and the computed poses were compared to the natural isoprenoid substrate FPP (Figure 1D). The highest-scoring pose of **1a** showed good alignment with FPP within the enzyme active site, where the tellurophene scaffold fits well within the binding pocket. This suggested that **1a** could be positioned favorably for enzymatic turnover despite the difference in hydrophobicity noted above.

Synthesis of the tellurophene-containing alcohol precursor was derived from procedures reported by Edgar et al. using a four-step route (Scheme 1).^44^ A triisopropylsilyl (TIPS)-protected diyne **5** was first synthesized using the Cadiot–Chodkiewicz coupling reaction. To prepare the tellurophene motif, elemental tellurium (Te^0^) was reduced using Rongalite (CH_3_NaO_3_S·2H_2_O) under basic aqueous conditions to form Te^2–^, which was subsequently reacted *in situ* with the deprotected diyne **6** to afford tellurophene compound **7** bearing a hydroxyl group handle. Preparation of isoprenoid precursors was accomplished following a three-step process similar to that used for the synthesis of related alkyne-containing analogues (Scheme 1).^39,41^ Commercially available geraniol **8** was used for the synthesis of C10 compound **1a** and trans*, trans*-farnesol **9** was employed for C15 compound **2a**, to yield THP-protected isoprenoids **12** and **13** with terminal hydroxyl groups. These intermediates were subsequently converted to chlorides **14** and **15** using tosyl chloride^45^ and then reacted with tellurophene **7** to form the ether-linked products using potassium *tert*-butoxide. The tellurophene-functionalized isoprenoids **16** and **17** were then converted to the desired diphosphates following a three-step procedure involving THP-deprotection, alcohol conversion to the corresponding bromide, and finally displacement with [(*n*-butyl)_4_]_3_P_2_HO_7_ (Scheme 1). The purification of the final products was achieved by first using Dowex ion exchange column to yield the corresponding ammonium salts, followed by chromatography on cellulose to isolate the desired diphosphate compounds **1a** and **2a**.^46^ For structural analysis, ^1^H NMR data collected in D_2_O, CD_3_OD, and DMSO-*d*_6_ along with ^13^C NMR and ^31^P NMR spectra obtained in D_2_O were used. A combination of several 2D NMR experiments was used to assign the spectra of intermediates **18** and **19** (Table S2) that facilitated the assignments for **1a** and **2a.** High-resolution mass spectrometry (HRMS) data displaying the characteristic isotopic pattern of tellurium, with the observed masses originating from the three most abundant tellurium isotopes were all in good agreement with the calculated values.

**Scheme 1:**
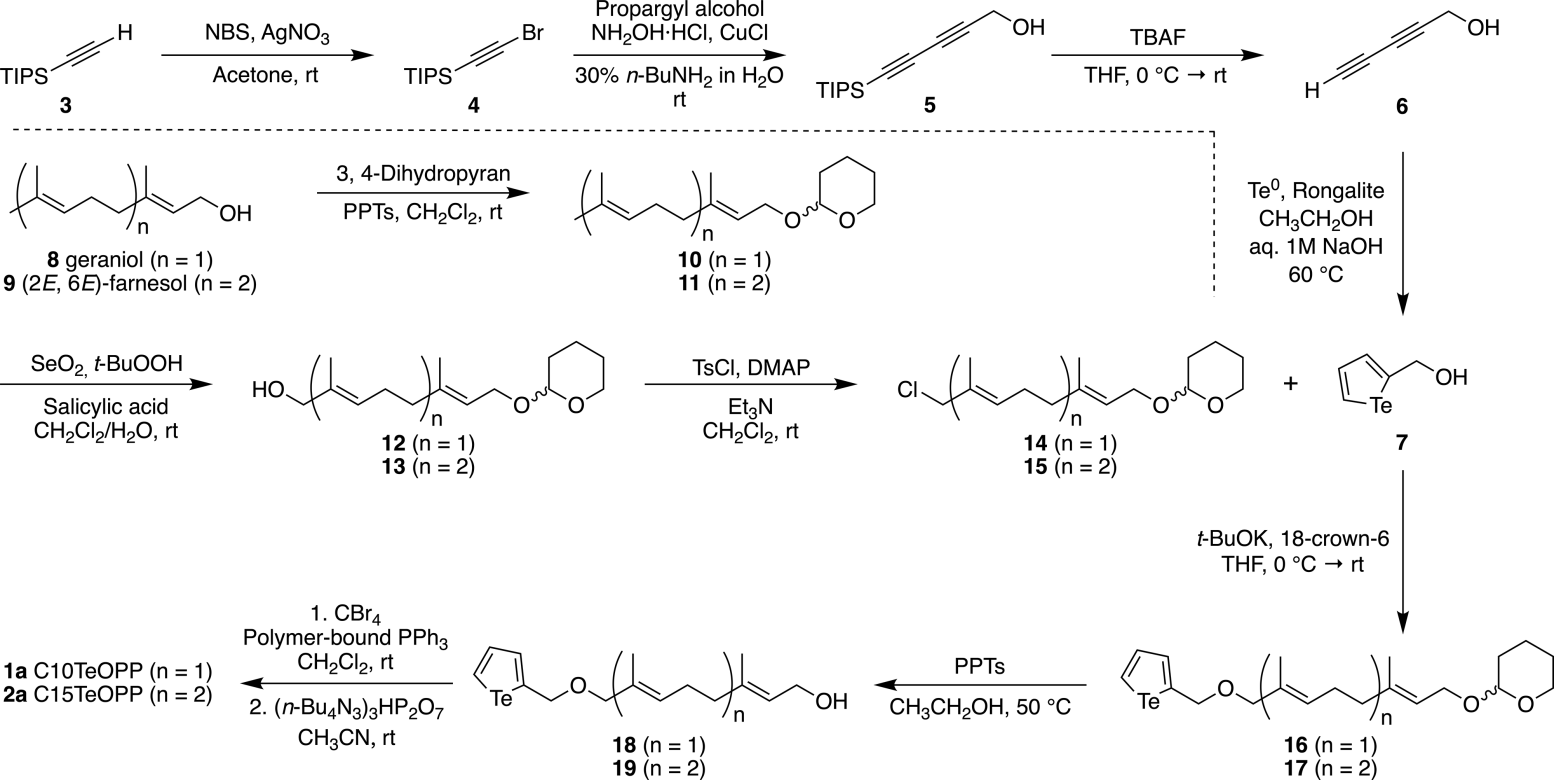
Synthetic Route for the Preparation of Tellurophene 7 and the Isoprenoid Precursors 14 and 15 en Route to Prenylation Probes C10TeOPP (**1a**) and C15TeOPP (**2a**); NBS: *N*-Bromosuccinimide; TBAF: Tetrabutylammonium Fluoride; PPTs: Pyridinium *p*-Toluenesulfonate; TsCl: *p*-Toluenesulfonyl Chloride; DMAP: 4-(Dimethylamino) Pyridine

### Design and Synthesis of Dihydro Probes for Background Assessment

In fluorescence-based analytical techniques, such as immunohistochemistry, fluorescence microscopy, and flow cytometry, as well as heavy metal-based mass cytometry, background signals arising from nonspecific retention of reporter molecules within cells are a common problem. A variety of strategies have been employed to evaluate the extent of such background.^31,47,48^ Here, a novel strategy was employed to address this issue. Previous work reported by Gibbs and co-workers demonstrated that FTase displayed no detectable activity (at least 450-fold less than FPP) with homoallylic substrate analogues.^49^ This can be attributed to the fact that prenyltransferases require an allylic diphosphate to stabilize carbocationic character in the transition state for the efficient enzymatic reaction.^50–52^ Hence, we reasoned that dihydro analogues lacking the C2–C3 alkene could be useful to measure the levels of background cellular labeling. Such compounds have physical properties nearly identical with those of their alkene-containing homologues but should not be enzymatically processed. Accordingly, the synthesis of dihydro analogues **1b** and **2b** was undertaken. Preparation of C10DHTeOPP (**1b**, Figure 1A) started with commercially available β-citronellol in place of geraniol to provide the isoprenoid backbone. To synthesize the longer analogue C15DHTeOPP (**2b**, Figure 1A), dihydro farnesol **22** was first prepared by adapting a three-step synthetic route previously reported by Arpicco et al. (Scheme S1).^53^ The syntheses were then completed using essentially the same routes employed for **1a** and **2a** (Scheme S2). It should be noted that **1b** and **2b** are racemic mixtures due to the stereogenic center at C3. The use of a THP protecting group in the synthetic route led to many of the intermediates being produced as mixtures of diastereomers because of the additional chirality at C1′. This was further complicated by alkene isomerism at the distal alkene formed during the hydroxylation reaction. To characterize the synthetic intermediates, spectral assignments were first established for **30** and **31**, which are key intermediates bearing both the tellurophene moiety and the THP protecting group. Using a combination of 1D and 2D NMR techniques, the major resonances were assigned (Table S2) and subsequently used to guide the analysis of the remaining intermediates and final products. The removal of the THP group near the end of the synthetic route substantially simplified the spectral analysis of downstream products. Finally, it should be noted that while these dihydro compounds may have some inhibitory activity toward prenyltransferases, preliminary experiments with an alkyne-containing dihydro analogue related to **1b** indicate only weak inhibition toward FTase *in vitro* (IC_50_ > 25 μM). It is possible that the two enantiomers of **1b** or **2b** may exhibit different inhibitory activities, as studies of related hydroxyfarnesylphosphonates have shown that different alkene isomers (*E* or *Z* at C2) exhibit strikingly different levels of FTase inhibition.^54^ Although methods to access enantiomerically pure forms of **1b** and **2b** have been developed,^55,56^ they were not pursued here to avoid significantly lengthening the synthetic route.

### Characterizing Prenyltransferase Specificity toward Tellurium-Modified Isoprenoid Analogues

Initial evaluation of the new tellurium-containing compounds as prenyltransferase substrates was performed using an established *in vitro* fluorescence assay with a dansylated “CaaX” peptide sequence.^39^ In this assay, covalent modification of the cysteine residue by a hydrophobic isoprenoid group enhances the fluorescence of the nearby dansyl group (Ds). Using Ds-GCVLS, a well-characterized substrate for farnesyltransferase (FTase), we observed dramatically more efficient incorporation of the shorter **1a** over longer **2a**, suggesting that **1a** is a more selective FTase substrate (Figure S1, panels A and C). Complementary experiments using Ds-GCVLL, a known substrate for GGTase-I, revealed an opposite trend where the longer **2a** produced a more rapid increase in dansyl fluorescence compared to **1a** (Figure S1, panels E and G). This is consistent with known substrate preference of GGTase-I for the longer isoprenoid GGPP compared with the shorter FPP. For dihydro probes **1b** and **2b**, no fluorescence change was detected with 20 nM enzyme (Figure S1, panels B and F). When elevated enzyme concentrations of FTase were tested (500 nM), no detectable reaction with **1b** or **2b** was observed (Figure S1, panel D). Based on those data, we estimate that the dihydro analogues are at least 1000-fold less reactive than FPP. Similar results were obtained in experiments with higher GGTase concentrations (200 nM; Figure S1, panel H). Collectively, these findings are consistent with prior reports on homoallylic substrates by Placzak et al. and indicate that the dihydro analogues are not enzymatically incorporated under biologically relevant conditions.

Next, the fluorescence-based assay was performed over a range of substrate concentrations to determine the kinetic parameters for the tellurium-containing substrates (Figures 1E and S2). For FTase, **1a** manifested a catalytic efficiency (*k*_cat_/*K*_M_) of 90% when compared to the natural substrate, FPP.^35^ In this case, a 3.4-fold decrease in *k*_cat_ was largely offset by a 3.1-fold decrease in *K*_M_. For GGTase-I, using **2a** as a substrate, the catalytic efficiency was 15% compared to GGPP.^36^ Interestingly, in this case, the *k*_cat_ value using **2a** was 2.3-fold higher relative to that of GGPP. However, the overall efficiency was decreased due to a 14-fold increase in *K*_M_ for **2a**. It is worth noting that while the *K*_M_ for **2a** is higher than the corresponding value for GGPP, metabolic labeling experiments are typically performed on cells using 5 to 10 μM of probe. Under such conditions, it is likely that the intracellular concentration of **2a** is on the order of 1 μM, meaning that the enzyme is nearly half saturated and the reaction rate with **2a** would be comparable to that with GGPP. Finally, while some incorporation of **1a** by GGTase-I and **2a** by FTase was observed, their slow reaction rates and lack of saturation precluded measurement of any catalytic parameters. Overall, these results suggest that **1a** and **2a** are orthogonal substrates for FTase and GGTase-I, respectively.

Since the above experiments were carried out using short peptide substrates, *in vitro* labeling was also explored using a larger protein substrate. For that purpose, a nanobody engineered with a C-terminal CVIA prenylation motif was employed.^57^ An *in vitro* prenylation reaction using *y*FTase and **1a** with the nanobody proceeded smoothly, as evidenced by the expected mass shift (Δ *m*/*z* = 344 Da, Figure S3A) upon prenylation, and a clear mobility shift shown via SDS-PAGE (Figure S3B).

Having established efficient reactivity of **1a** and **2a** *in vitro*, we next focused on *in cellulo* reactions. For these experiments, the well-documented change in electrophoretic mobility that occurs upon protein prenylation was exploited.^39^ Thus, COS-7 cells were pretreated with 25 μM lovastatin to inhibit endogenous FPP/GGPP synthesis, thereby reducing *in cellulo* farnesylation of H-Ras and geranylgeranylation of Rap1B. Cells were subsequently treated with varying concentrations of either a tellurium-containing probe or a natural isoprenoid in an attempt to restore prenylation. For H-Ras, farnesylation inhibited by 25 μM lovastatin was rescued by the addition of FPP as well as by C10TeOPP (**1a**) to some extent, but not by the longer lipids GGPP or C15TeOPP (**2a**) (Figures 2A and S4). This is consistent with the fact that H-Ras is prenylated only by FTase whose endogenous substrate is FPP. In contrast, Rap1B geranylgeranylation inhibited by 25 μM lovastatin was rescued efficiently by either GGPP or **2a**, to a lesser extent by FPP, but not at all by **1a** (Figures 2B and S4). Those observations are in line with previous works showing that Rap 1B is recognized only by GGTase-I, which uses GGPP as the prenyl group donor. These results are in good agreement with the aforementioned kinetic data and support the idea that **1a** and **2a** are orthogonal with respect to different prenylation enzymes.

**Figure 2:**
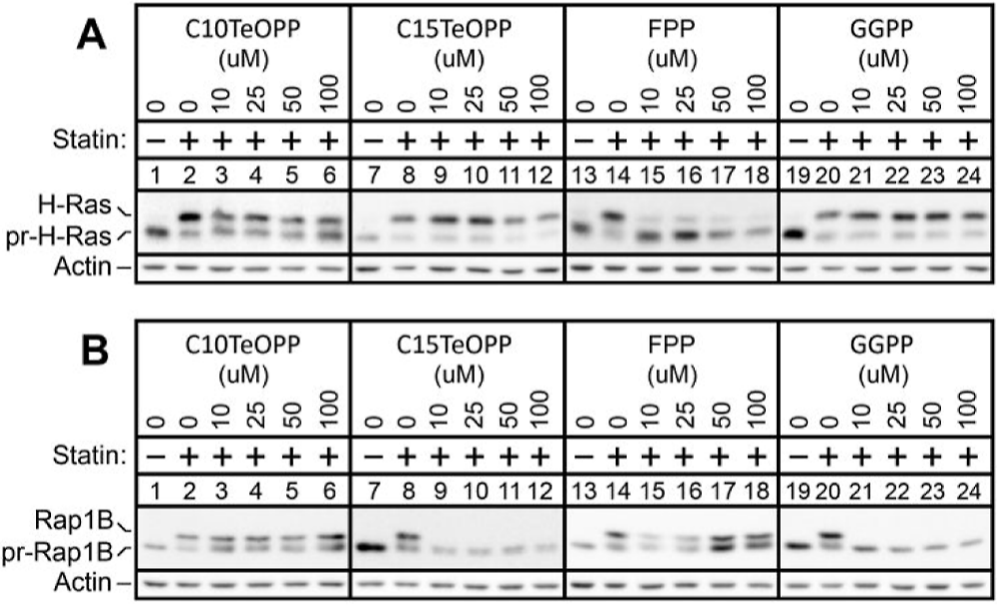
Western blot analysis of prenylation states of H-Ras and Rap 1B proteins. (A) Analysis of H-Ras prenylation. (B) Analysis of Rap1B prenylation. COS-7 cells were subjected to statin-induced prenylation inhibition and attempted rescue with different isoprenoid diphosphates. Cells were treated with 25 μM lovastatin for 1.5 h (except for the samples indicated with -) followed by supplementation with different isoprenoids for an additional 18 h while retaining the statin. Cells were then harvested, and the lysates were fractionated via SDS-PAGE and transferred to PVDF membranes. Proteins were visualized by immunoblotting with H-Ras and Rap1B antibodies. The positions (mobilities) of the prenylated (indicated with “pr-”) and unprenylated protein forms are noted on the left side of each image.

To further demonstrate *in cellulo* incorporation, competition experiments were performed using an alkyne-functionalized probe. Here, COS-7 cells were cotreated with a tellurium probe and C15AlkOPP, followed by the CuAAC reaction with TAMRA-Azide on the resulting lysate to visualize alkyne-labeled proteins (Figure S5). Co-treatment with **1a** led to a dose-dependent reduction in the TAMRA signal, particularly in the 50–75 kDa range, which is enriched in farnesylated proteins. Co-treatment with **2a** reduced protein labeling primarily around the 25 kDa region, where many known GGTase-I and RabGGTase substrates can be found.

### Global and Protein-Level Prenylation Quantification in AML-3 Cells

We next focused on the application of new tellurium probes in CyTOF experiments to detect prenylation in intact cells. Cultured AML-3 cells (a human acute myeloid leukemia cell line) were treated with varying concentrations of either **1a** or **2a**, followed by live/dead cell staining, fixation, permeabilization, and DNA staining. A parallel sample was treated with the same concentration of the corresponding dihydro probe **1b** or **2b** and subjected to the same workflow. Of note, although the tellurophene group was introduced through metabolic labeling using a one-step enzymatic approach, cell fixation, and permeabilization steps were retained in the workflow to maintain compatibility with existing antibody-based probes for CyTOF in future experiments. Gratifyingly, signals from several major tellurium isotopes (^122^Te, ^124^Te, ^125^Te, ^126^Te, ^128^Te, and ^130^Te, Figure S6) were detected in both **1a**- or **2a-**treated samples. Compared to their corresponding dihydro analogs, the C10 and C15 probes exhibited markedly stronger signals, over 14-fold and 4.6-fold higher, respectively, when the experiments were performed at a probe concentration of 5 μM (Figure 3A,B). Interestingly, the data from the dihydro compounds showed that C15DHTeOPP (**2b**) generates a ca. 2-fold higher level of signal compared to C10DHTeOPP (**1b**). This may be attributed to its increased hydrophobicity, which likely promotes nonspecific interactions and associations with cellular membranes or hydrophobic compartments within the cell. Despite this, the clear signal differences between substrates (**1a** and **2a**) compared with the control dihydro probes (**1b** and **2b**) were encouraging and support the utility of these tellurium analogs in cellular labeling applications.

**Figure 3:**
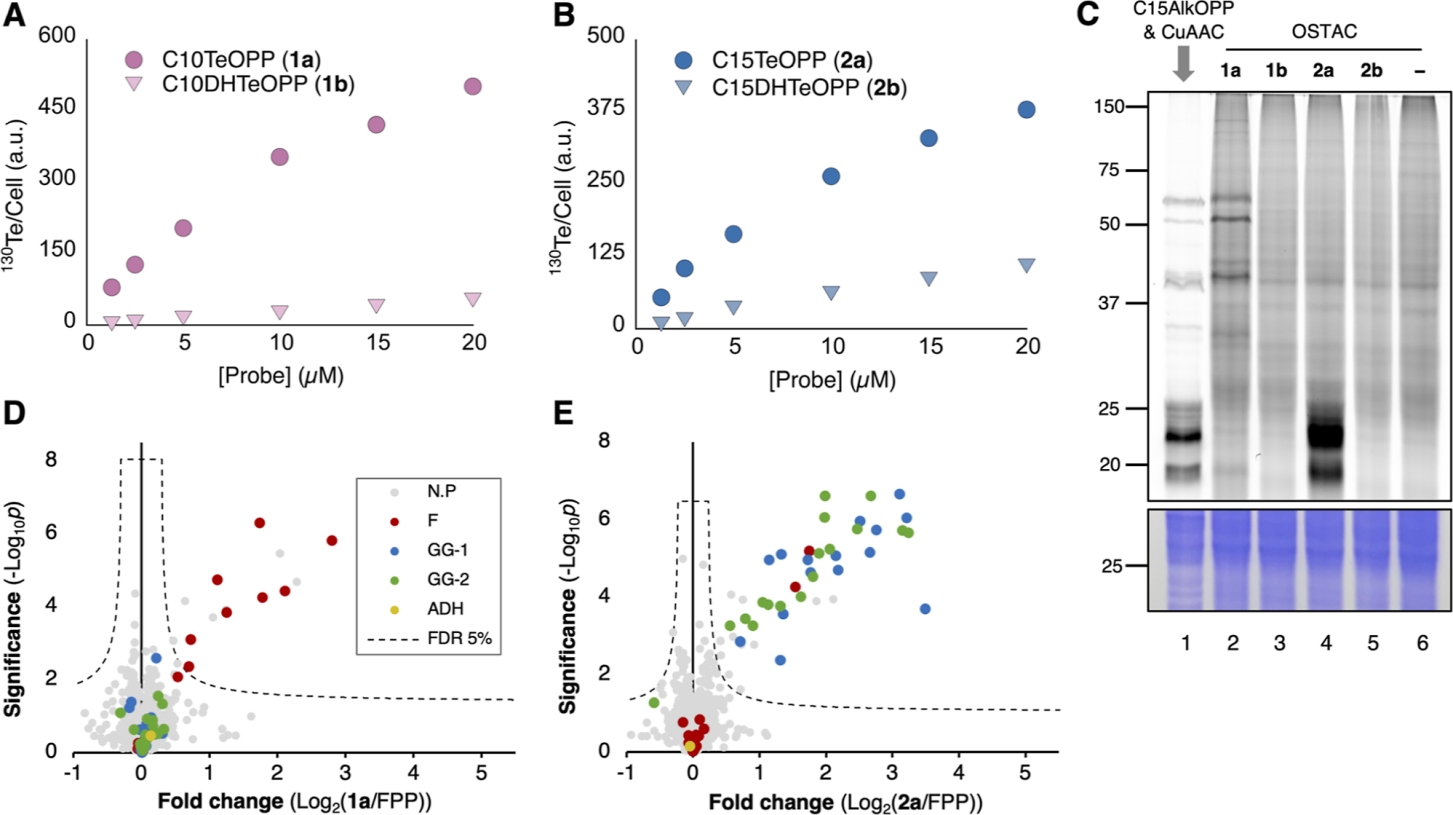
Evaluation of tellurium probe labeling in AML-3 cells. (A) Mass cytometry analysis of cells treated with C10TeOPP (**1a**) or its nonreactive analog C10DHTeOPP (**1b**) over a range of probe concentrations. (B) Mass cytometry analysis of cells treated with C15TeOPP (**2a**) or its nonreactive analog C15DHTeOPP (**2b**) over a range of probe concentrations. Cells were treated for 24 h, and the tellurium signal was analyzed using a standard CyTOF workflow; (C) Comparing the metabolic labeling pattern of C15AlkOPP and tellurium probes: cells were treated with 10 μM probes for 24 h, followed by cell lysis and CuAAC reaction for the C15AlkOPP-treated sample (Lane 1) with 25 μM TAMRA-Azide, or OSTAC reaction for tellurium probe-treated samples (Lane 2–5) with 25 μM BCN-TAMRA; “no probe” sample (Lane 6, indicated by -) was subjected to the same OSTAC reaction. Top panel: TAMRA fluorescence scan; bottom panel: Coomassie blue staining showing total protein loading; (D,E) Identification of tellurium probe-labeled proteins via chemical proteomics using BCN-Biotin sulfone and TMT 10-plex design showing proteins labeled by **1a** (panel D) and **2a** (panel E). FPP-treated samples served as the control in both experiments. Volcano plots were generated from a two-tailed *t*-test comparing the normalized TMT reporter ion intensities across three biological replicates per condition, using FDR = 5% and s0 = 0.1. Color scheme: FTase substrates (F, red); GGTase-I substrates (GG-1, blue); GGTase-II substrates (GG-2, green); yeast alcohol dehydrogenase internal standard (ADH, SwissProt P00330, yellow), and proteins not reported as prenylation substrates (N.P., gray).

The preceding CyTOF experiments provided indirect evidence of the incorporation of the tellurium-containing probes into prenylated proteins. However, they did not unambiguously identify the labeled proteins within the cells, highlighting the need for complementary chemical methods to verify target engagement. Recent work by Bu et al. described a bioorthogonal tellurophene-based conjugation reaction, termed oxidation-controlled, strain-promoted tellurophene-alkyne cycloaddition (OSTAC).^58^ This chemistry enables direct modification of tellurophene-labeled proteins using a bicyclononyne (BCN)-containing reagent upon the oxidation of tellurophene with *N*-chlorosuccinimide (NCS), thereby offering a strategy to selectively modify probe-labeled proteins for downstream fluorescence-based detection or affinity-based enrichment and identification. To this end, cells were treated with tellurium-containing probes and subjected to an OSTAC reaction after lysis using BCN-TAMRA (Scheme S3). In-gel fluorescence analysis revealed distinctly different labeling patterns for **1a** and **2a** compared to the labeling obtained using C15AlkOPP (Figure 3C). Specifically, labeling by **1a** (Figure 3C, lane 2) was observed almost exclusively in the 50–75 kDa range (characteristic of farnesylated proteins), while labeling by **2a** (Figure 3C, lane 4) occurred predominantly in the 20–25 kDa range (where many geranylgeranylated proteins migrate). Those results are in stark contrast to those obtained with C15AlkOPP (Figure 3C, lane 1) which is a well-characterized pan-prenylation probe that labels both farnesylated and geranylgeranylated proteins. In addition, cells treated with either **1b** or **2b** under the same conditions did not exhibit any labeling above the background, demonstrating that these dihydro compounds cannot be enzymatically incorporated.

To elucidate the identities of tellurium probe-labeled proteins, chemical proteomics analysis was performed. In this case, BCN-biotin sulfone (Scheme S4), a preoxidized form of BCN-biotin was employed. This reagent was chosen to prevent unwanted biotin oxidation to biotin sulfoxide under OSTAC reaction conditions, which would otherwise diminish its binding affinity toward avidin.^58,59^ Probe-treated cells and FPP-treated cells (each prepared in triplicate) were lysed and subjected to the OSTAC reaction with BCN-biotin sulfone followed by enrichment with Neutravidin beads, washing, and on-bead digestion with trypsin. The resulting peptides were labeled with tandem mass tag (TMT) reagents, multiplexed, fractionated under high-pH reversed-phase conditions, and underwent LC–MS analysis using a data-dependent acquisition strategy with multinotch MS^3^ for TMT reporter ion quantification. Protein profiling with C10TeOPP (**1a**) identified a total of 18 known farnesylation substrates, 8 of which were significantly enriched (Figure 3D). Although a total of 31 geranylgeranylation substrates were observed, none were detected using **1a** with statistical confidence. In contrast, profiling with C15TeOPP (**2a**) revealed significant enrichment of 15 GGTase-I substrates and 16 RabGGTase substrates, while only 2 farnesylation substrates appeared in the enriched region (Figure 3E). A complete list of proteins identified is provided in Table S3.

Next, the effects of substrate competitors and prenyltransferase inhibitors were examined by using both mass cytometry and OSTAC-based in-gel fluorescence detection. Thus, AML-3 cells were treated with **1a** or **2a** along with FPP or GGPP as substrate competitors, or with selective enzyme inhibitors Tipifarnib (FTase inhibitor) or GGTI-298 (GGTase-I inhibitor). In the case of C10TeOPP (**1a**), a clear reduction in signal/probe labeling was observed in response to Tipifarnib treatment whereas no changes were observed using GGTI-298 (Figures 4 and S7A,C,D). This is consistent with the idea that the incorporation of **1a** occurs predominantly via FTase. The competition experiment between **1a** and FPP did not result in significant signal reduction, which was probably attributable to the higher affinity of **1a** for FTase relative to FPP (compare *K*_M_ = 0.48 μM for **1a** versus 1.5 μM for FPP, Figure 1E). Interestingly, cotreatment with **1a** and GGPP led to a noticeable signal reduction observed via mass cytometry but not using the gel-based method. This discrepancy may be due to competition between GGPP and **1a** for nonspecific binding sites within cells. Such effects would be expected to appear in mass cytometry experiments performed on intact cells in contrast to gel-based measurements that are conducted under denaturing conditions. For C15TeOPP (**2a**), inclusion of GGPP but not FPP led to a significant decrease in probe labeling observed in both types of experiments. Treatment with GGTI-298 caused a reduction in the incorporation of **2a** but not **1a**, consistent with the former being a substrate for GGTase but not the latter. In contrast, inclusion of Tipifarnib resulted in decreased labeling by both **1a** and **2a**. Since **1a** is an FTase substrate, such a reduction was expected. For **2a**, the decrease may result from an intracellular accumulation of FPP (due to FTase inhibition), which could shift the metabolic flux toward increased GGPP production, causing competition.

**Figure 4:**
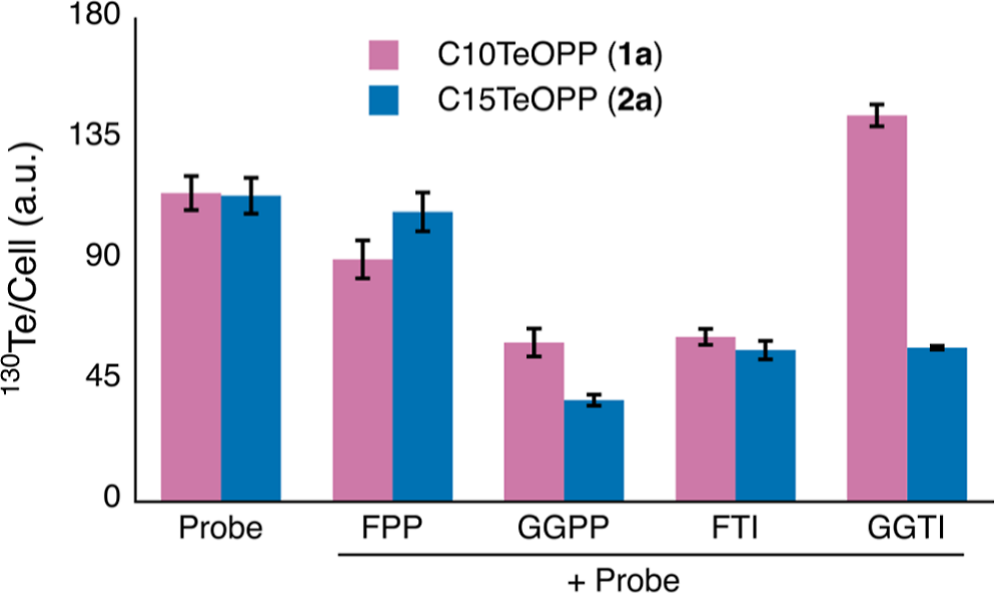
Substrate competition and enzymatic inhibition assays of tellurium probes. Mass cytometry was used to quantify total prenylation levels at the single-cell level. AML-3 cells were treated with 5 μM probe for 24 h. Natural isoprenoid FPP or GGPP as a substrate competitor, or enzyme inhibitor concentrations were used at 10 μM, and were cotreated with the tellurium probes. FTI: Tipifarnib; GGTI: GGTI-298.

Overall, the data reported above illustrate the impressive differential selectivity of FTase for C10TeOPP (**1a**) versus that of geranylgeranylation enzymes for C15TeOPP (**2a**). Of particular significance, the dramatically smaller sample size required for CyTOF analysis should be noted. Whereas the gel-based experiments shown here require at least 5 × 10^6^ cells and current proteomic workflows demand 4–7 times more material, cytometric analysis can be performed with as low as ∼1 × 10^6^ cells. This dramatically reduced sample requirement greatly enhances the utility of that approach, particularly in contexts where the biological material is scarce such as experiments with primary cells, purified populations obtained via fluorescence-activated cell sorting (FACS), or animal tissues. The ability to monitor global levels of farnesylation and geranylgeranylation in those systems using this new method should be highly impactful.

### Broadening the Scope to Other Cellular Models

After the methodology was established in AML-3 cells, the new tellurium-containing probes were studied in several other cell lines to examine the generality and robustness of this approach. Three additional cell lines, including MOLM-13 (human acute myeloid leukemia), COS-7 (African green monkey kidney fibroblast-like cell line), and AML-12 (alpha mouse liver 12), were examined to sample different tissue origins and adherent versus nonadherent cell types. For MOLM-13, mass cytometric analysis following treatment with both substrate probes and their nonreactive dihydro analogs yielded signal patterns comparable to those observed in AML-3 cells (Figures 3A,B and 5). However, the level of background signal due to the dihydro probe was somewhat higher (42% here versus 7% for AML-3 in the case of C10 compound pairs **1a** and **1b**). To evaluate nonspecific binding in more detail, an additional detergent treatment step (0.1% Triton X-100 in PBS) was included in the workflow to facilitate the removal of noncovalently bound probes from the samples. A substantial reduction in signal from dihydro probe-treated samples was observed after adding this step, suggesting that a significant portion of these signals arises from nonspecific cellular membrane binding. In contrast, signals from the enzymatically processed substrate probe **1a** remained unchanged in those cells, indicating a predominantly covalent incorporation. Interestingly, in AML-3 cells, a noticeable signal reduction was observed in samples treated with **1a** or **2a** after the Triton wash, indicating that a portion of the tellurium signal originated from an unincorporated probe that can be removed through detergent treatment (Figure S8). In substrate competition and enzyme inhibition experiments in AML-3 cells (Figures 4 and S7), the extent of signal reduction observed in mass cytometry did not always match the gel-based results, as a residual signal persisted even in the presence of competitors or inhibitors. These findings support the hypothesis that despite a portion of the CyTOF signal observed in AML-3 cells was coming from nonspecifically retained probes, the majority of the signal reflects genuine prenylation-related activity.

**Figure 5:**
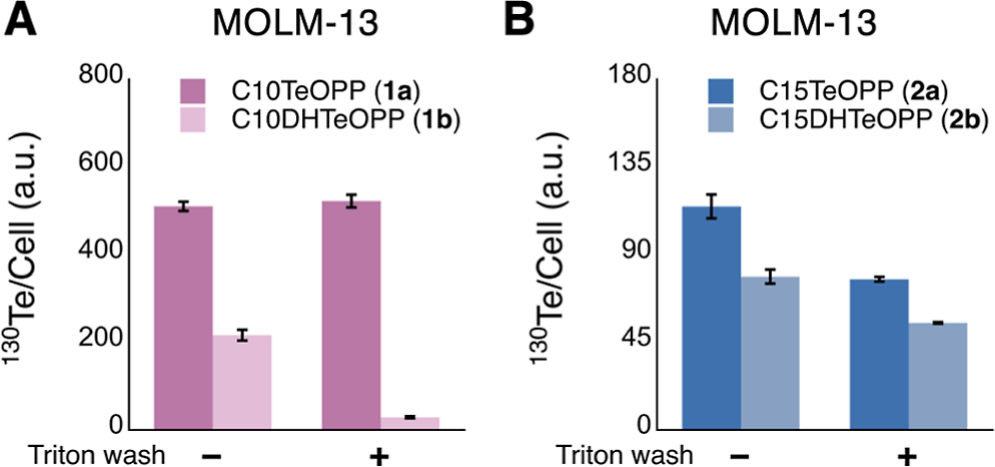
Mass cytometry analysis of tellurium probes performed in MOLM-13 cells. (A) Cells were treated with 10 μM of C10TeOPP (**1a**) or its nonreactive analog C10DHTeOPP (**1b**) for 24 h. (B) Cells were treated with 10 μM of C15TeOPP (**2a**) and C15DHTeOPP (**2b**) for 24 h. In both experiments, for each replicate, half of the sample was subjected to a Triton X-100 wash after fixation and permeabilization while for the other half, the Triton treatment was omitted.

Switching to adherent cell lines, probe incorporation was first examined in COS-7 via an in-gel fluorescence analysis (Figure S9). Protein labeling patterns were comparable to those observed in AML-3 cells (Figure 3C), with **1a** and **2a** exhibiting the selective labeling of distinct farnesylated and geranylgeranylated protein subsets, whereas the alkyne-functionalized C15AlkOPP labeled both types, consistent with its pan-prenylation selectivity. No detectable protein labeling was observed in samples treated with the dihydro probes, consistent with the *in vitro* data showing that these latter compounds are not prenyltransferase substrates.

In contrast, in mass cytometry experiments, unexpectedly high levels of signal from the dihydro probe compared to their corresponding substrate analogs was observed (Figures 6A and S10). These signals persisted even after washing with Triton X-100 or other detergents (data not shown), indicating that these compounds may have strong noncovalent interactions with certain hydrophobic or membrane-associated regions within the cells. In AML-12 cells, tellurium signals from **1a** relative to its dihydro analog **1b** showed better substrate-to-background ratios compared to COS-7 cells, both before and after the Triton wash (Figure 6B). This indicates a lower level of nonspecific retention for the dihydro probe in AML-12. Interestingly, when COS-7 cells were cotreated with substrate probe **1a** and FTase inhibitor Tipifarnib, no discernible difference in the signal was observed prior to Triton washing. However, a signal difference became apparent after Triton treatment (Figure S11), indicating that a substantial portion of the prewash signal originated from prenylation-independent probe retention. Nonetheless, the postwash data still captured prenylation-dependent changes, demonstrating that the system retains sensitivity to pharmacological treatments despite some discernible background interference likely arising from nonspecific probe retention. In general, the availability of the dihydro probes that report on the level of background labeling is a powerful feature of this mass cytometry approach, enabling analysis of global prenylation levels and evaluation of tellurophene-based compounds performance across different cell lines, where background labeling reflects the interaction of the probe with nonspecific targets.

**Figure 6:**
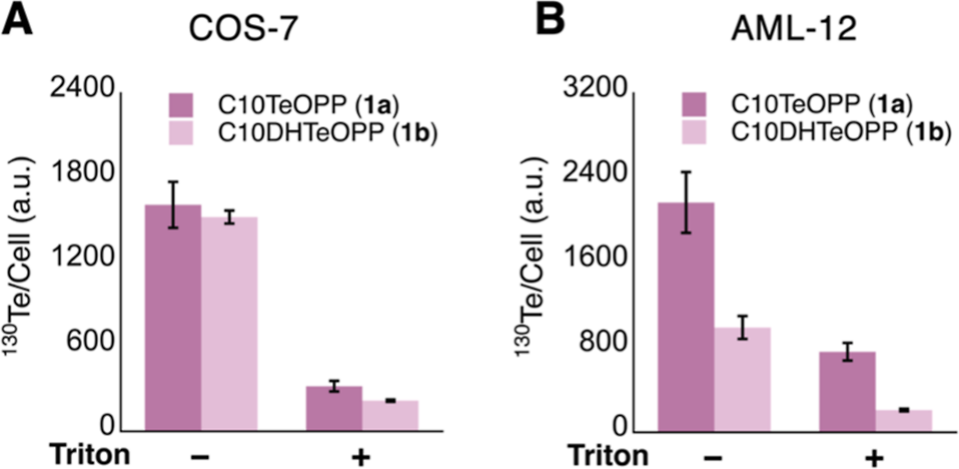
Mass cytometry analysis with tellurium probes performed in COS-7 (panel A) and AML-12 (panel B). Cells were treated with 10 μM of C10TeOPP (**1a**) or its nonreactive analog C10DHTeOPP (**1b**) for 24 h. In both experiments, for each replicate, half of the sample was subjected to a Triton X-100 wash prior to fixation and for the other half, the Triton treatment was omitted.

### Disrupted Autophagy Modulates Protein Prenylation Profiles in L6 Cells

Autophagy is a vital cellular process that maintains homeostasis by degrading and recycling damaged or surplus intracellular components.^60^ This lysosome-dependent process involves the formation of double-membrane autophagosomes that engulf cytoplasmic cargo including dysfunctional organelles and misfolded proteins, which subsequently fuse with the lysosome to facilitate degradation and reuse of cellular material. This process is tightly regulated by several autophagy-related genes (ATGs), among which ATG7 plays a critical role. ATG7 is essential for two ubiquitin-like conjugation systems, the ATG8/ATG3 pair and ATG12/ATG10 pair, which drive autophagosome formation and expansion.^61^ Loss of ATG7 results in impaired autophagosome biogenesis and disruption of autophagy pathways and has been linked to diverse physiological and pathological conditions.^62^ Since multiple prenylated small GTPases, including several Rab proteins, are central regulators of autophagy-related vesicle trafficking,^63,64^ disrupting the autophagy process is likely to have an impact on the post-translational modification of these proteins.^60,63^

To investigate how compromised autophagy could affect the prenylation process, the Atg7 gene was functionally knocked out in L6 cells (immortalized rat skeletal myoblast cell line) through a single point mutation resulting in a biallelic single base pair deletion in exon 2 generated by CRISPR-Cas-9 technology.^48^ Previously, this cell model was shown to reduce the gene expression of ATG7 to 37% as well as the LC3-II/LC3-I ratio, thus rendering the cell line functional with reduced autophagy.^48^ Mass cytometry analysis using the tellurium-based probes developed here revealed a marked reduction of probe incorporation in Atg7 knockout (Atg7 KO) cells compared with wild type (WT) controls. Both C10TeOPP (**1a**) and C15TeOPP (**2a**) showed decreased signal levels to 67% and 88%, respectively, compared to WT cells) in the knockout model (Figures S12A and S7A), indicating global attenuations of farnesylation and geranylgeranylation under autophagy-deficient conditions. These findings were further corroborated by chemical proteomics using the alkyne-functionalized C15AlkOPP probe. Profiling experiments successfully identified 36 prenylated proteins in WT L6 cells and 44 prenylated proteins in Atg7 KO L6 cells (grouped data, Figure S13; full protein list in Table S3), demonstrating effective probe incorporation into prenyltransferase substrates. In the comparative analysis between two cell models (Figure 7B; full protein list in Table S3), reduced labeling of several prenylated proteins was observed in the Atg7 KO cells, including Rab2a and 2b,^65^ Rab7a,^66,67^ Rab10,^68^ and Rab13,^69^ ^,^ ^70^ which are required for canonical autophagy.

**Figure 7:**
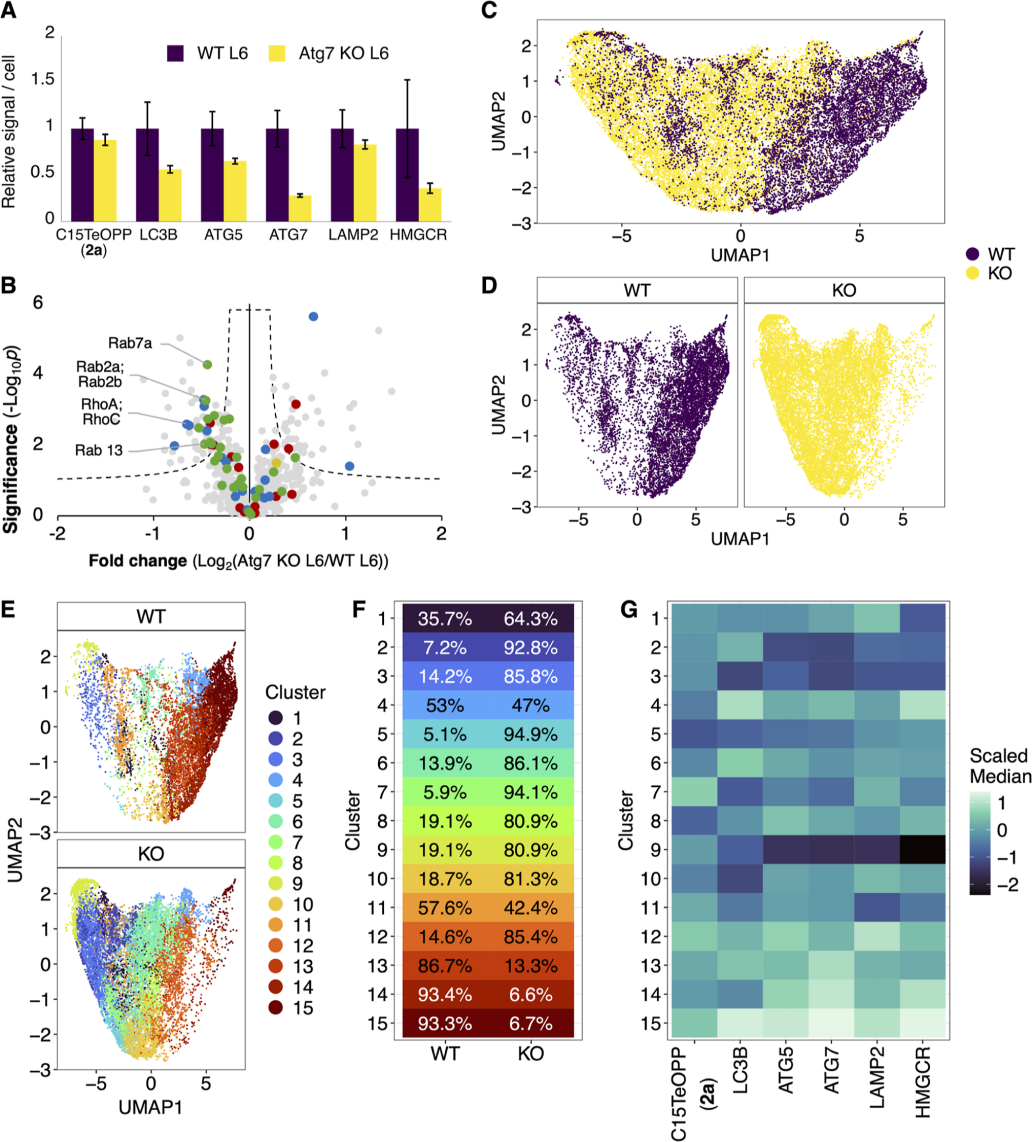
Analysis of prenylation in wild type and Atg7 KO L6 cells. (A) Mass cytometry analysis shows Atg7 KO cells exhibits reduced incorporation of C15TeOPP (**2a**) and lower levels of several autophagy-related proteins as well as HMGCR. Signal intensities from probes and antibodies are shown relative to WT. (B) Proteomic analysis with C15AlkOPP reveals diminished labeling of several prenylated proteins in the Atg7 KO model. Volcano plots were generated using a two-tailed *t*-test comparing normalized TMT reporter ion intensities across three biological replicates per cell model (FDR = 5%, s_0_ = 0.1). Color scheme: FTase substrates (red); GGTase-I substrates (blue); GGTase-II substrates (green); yeast alcohol dehydrogenase internal standard (ADH, SwissProt P00330, yellow); proteins not reported as prenylation substrates (gray). (C,D) Dimensionality reduction shows clear separation of WT L6 (yellow) and Atg7 KO L6 (purple) cell populations. UMAP plots are shown with samples overlaid (C) or separated by cell line (D). (E–G) Clustering analysis reveals differences within each cell line. (E) UMAP plots of WT and Atg7 KO lines, color-coded by cluster. (F) Proportion of cells in each cluster derived from each sample. (G) Heatmaps showing individual marker levels across clusters.

Mass cytometry experiments with the nonreactive dihydro probes yielded additional features of probe behaviors. C15DHTeOPP (**2b**) produced similar levels of signal in both WT and Atg7 KO cells (Figure S12B), indicating that the signal likely originated from nonprenyltransferase-dependent activity. Interestingly, C10DHTeOPP (**1b**) produced a measurable difference between the two cell models (Figure S12A). One possible explanation is that perturbed autophagy alters how cells internalize or retain certain isoprenoid analogs.

In addition, the efficiency of removing nonreactive probes may depend on their subcellular localization in each cell type, which could in turn influence how effectively the washes in the CyTOF sample preparation workflow remove unincorporated compounds.

### Exploiting the Power of Mass Cytometry and Tellurium Probes

To investigate the compatibility of the new prenylation probes with conventional antibody-based mass cytometry reagents, additional experiments with C15TeOPP (**2a**) were performed in combination with several antibodies targeting key autophagy markers (Table S4). As expected, knocking out Atg7 reduced its expression level (Figure 7A). Other autophagy-related proteins were studied, including ATG5, which is activated by ATG7 to form a complex that elongates the phagophore membrane in the creation of the autophagosome; LC3B, another protein involved in building the phagophore membrane; and lysosome-associated membrane protein 2 (LAMP2), a lysosomal protein with three isoforms, each playing critical roles in autophagy.^71^ All three proteins manifested decreased levels in the Atg7 KO line (Figure 7A). In addition to the aforementioned autophagy-related proteins, 3-hydroxy-3-methylglutaryl-CoA reductase (HMGCR), a key protein in the mevalonate pathway through which cholesterol and the isoprenoid substrates, FPP and GGPP, for prenylation are synthesized,^4^ was also impacted. HMGCR plays a critical role in the mevalonate pathway and is a target for treatments to reduce cholesterol.^72^ Levels of HMGCR decreased with a reduced level of Atg7 (Figure 7A). Similarly, the C15TeOPP probe had decreased incorporation in the Atg7 KO cells. The decreased incorporation of the C15TeOPP probe could result from either increased competition from GGPP, the endogenous substrate, or from decreased levels of prenyltransferase activity. Since HMGCR levels were decreased in the KO line, which would reduce the endogenous FPP and GGPP levels, it is likely that the decrease in C15TeOPP incorporation represents an overall reduction in protein geranylgeranylation. It should be noted that this mass cytometric analysis involved the simultaneous quantification of 8 cellular markers (including cell viability analysis by cisplatin and cell identification by DNA-intercalator-Ir), a challenging task using conventional flow cytometry. Taken together, these results highlight that the newly developed tellurium-based probe is compatible with conventional mass cytometry measurements of protein levels and that disrupting autophagy decreases prenylation. Overexpression of components in the mevalonate pathway has previously been shown to induce senescence via mitochondrial alterations which increase the release of reactive oxygen species (ROS).^73^ Autophagy has been shown to decrease with age but also decrease in a dysfunctional way with senescence.^74^ Future studies could include investigating the triaxial relationship among autophagy, the mevalonate pathway, and senescence, including exploring whether downregulation of autophagy to an extent or via a specific target could help rescue senescent cells or prevent senescence.

The true power of mass cytometry measurements lies not only in the ability to measure multiple analytes simultaneously but also in the use of that information to understand their relationships in biological processes. Dimensional reduction via Uniform Manifold Approximation and Projection (UMAP) demonstrates distinctions between the WT and Atg7 KO cells (Figure 7C,D). These distinctions are in line with the decrease in transcript abundance of autophagy and the mevalonate pathways. (Figure 7A). ATG7 and C15TeOPP have similar staining patterns with higher levels of incorporation on the right side of the UMAP (Figure S14). Other markers also follow this trend (Figure S15).

To investigate the relationship between autophagy and prenylation further, PhenoGraph was used to cluster the cells (Figure 7E). The result shows a similar pattern between two cell models as is seen purely with dimensional reduction (Figure 7C,D), with the lower numbered clusters (clusters 2, 3, 5, 6, 7, 8, 9, 10, and 12) containing more cells from the Atg7 KO populations, while the higher numbered clusters (clusters 13, 14, and 15) pull cells from the WT populations (Figure 7E,F). For instance, 92.8% of cells in cluster 2 are from the KO cell line, whereas 93.3% of cells in cluster 15 are from the WT samples.

Different clusters that pull from the same population reveal differences within the cell model. For example, clusters 2, 3, 8, and 9 are all majority cells from the KO line. However, while clusters 2, 3, and 9 have lower levels of markers in general, cluster 8 has higher levels of ATG5 and HMGCR (Figure 7G). This difference in marker levels of individual clusters could arise from differences of individual cells within the population (for example, due to differences in stages of the cell cycle or levels of Atg7 gene after the point mutation deletion), but it points to relationships between markers. For instance, cluster 8 also has much lower levels of prenylation, which might suggest some other regulator that was not measured is impacting cell protein expression. Cluster 7, interestingly, is almost entirely from the Atg7 KO cell line and shows the reverse relationship of cluster 8, with higher levels of prenylation but lower levels of most other markers. In the future, studies with additional markers, including those for phases of the cell cycle and certain specific prenylated proteins, should help elucidate these relationships. At this stage, these results show that combined measurements of prenylation with autophagy markers present a promising avenue for exploring this complex process.

## Conclusions

In this study, we introduced a new class of tellurium-containing isoprenoid probes that enables the one-step enzymatic installation of a compact reporter group onto prenylated proteins. These probes provide selective labeling of farnesylation and geranylgeranylation substrates and are compatible with mass cytometry workflows. Compared to conventional gel-based or proteomic methods, this approach requires substantially fewer cells and uses a more streamlined sample preparation process without employing additional chemical modification, opening the possibility of interrogating prenylation dynamics in scarce or heterogeneous samples, including primary cells and purified populations. In addition, utilizing these tellurium probes with gel-based analysis or chemical proteomics allowed their specificity to be directly evaluated, demonstrating their ability to label a broad range of prenylated substrates and establishing these compounds as versatile tools for prenylation research.

Application of these probes to an autophagy-deficient model demonstrated that the disruption of ATG7 expression leads to changes in the prenylation profiles. Mass cytometry revealed reduced probe incorporation in Atg7 KO L6 cells, while proteomic analysis identified diminished labeling of multiple Rab family proteins involved in autophagy regulation. These results provide direct evidence that autophagy and prenylation are interconnected processes with impaired autophagy leading to reduced prenylation activity. The simultaneous measurement of prenylation alongside autophagy-related proteins and mevalonate pathway components further underscores the capacity of this platform to interrogate pathway-level relationships in complex cellular systems.

More broadly, the tellurium-based strategy described here should be readily adaptable to other types of post-translational modifications, such as glycosylation, ubiquitination, or other types of lipidation, including palmitoylation. Given the successful incorporation of related isoprenoid probes into live mice,^23,24^ future extensions of these prenylation probes could include application to imaging mass cytometry, enabling spatially resolved analysis of prenylation and related biomarkers in tissues. Together, these advances expand the chemical biology toolkit for studying protein prenylation and open new opportunities for exploring post-translational modifications in both normal physiology and disease.

## Supplementary Material





## Data Availability

The proteomic data have been deposited in the PRIDE repository via the ProteomeXchange platform under accession numbers PXD068330 and PXD037896. All data supporting the findings of this study are openly accessible through the Data Repository for the University of Minnesota (DRUM) at 10.13020/626z-dw82.

## Notes

Calculated properties of isoprenoid alcohols; enzyme kinetic analysis; *in vitro* prenylation; quantitative analysis of western blots; mass cytometry analysis and heat maps of markers; prenylomic analysis of WT and Atg7 KO L6 cells; substrate competition and enzymatic inhibition assays; material and methods; synthetic methods for C10TeOPP, C15TeOPP, C10DHTeOPP, C15DHTeOPP, BCN-TAMRA, and BCN-Biotin sulfone; and associated spectral data of synthetic compounds (PDF)
Lists of proteomic results for all proteins in all experiments (XLSX)

## #

Binghamton University, 4400 Vestal Parkway East, PO Box 6000, Binghamton, NY 13902-6000

## ¶

Biotechne, 614 McKinley Pl NE, Minneapolis, MN 55413.
