# Supplementary material for: Organotellurium Probes Enable One-step Single-cell Analysis of Post-translational Modification
Source: J Am Chem Soc. 2026 Feb 17;148(10):10627–39. doi: 10.1021/jacs.5c19824 (PMC13003479; doi:10.1021/jacs.5c19824)
Supplement: Supplementary file 1 [file ja5c19824_si_002.pdf]

Organotellurium Probes Enable One-step Single-cell Analysis of Post-translational Modification

Yuanzhe Chen[a], Kris Elbein[a], Sneha Venkatachalapathy[a], Ellen L. Lorimer[b], Andrea M. Sprague-Getsy[c], Shelby A. Auger[a], Zoë A. Maxwell[a], Mohammad Rashidian[d], James L. Hougland[c], Carol L. Williams[b], Edgar A. Arriaga[a], and Mark D. Distefano[a]*

[a] Department of Chemistry, University of Minnesota-Twin Cities, Minneapolis, Minnesota, 55455, United States

[b] Department of Pharmacology and Toxicology, Medical College of Wisconsin, Milwaukee, Wisconsin, 53226, United States

[c] Department of Chemistry, Syracuse University, Syracuse, New York 13244, United States; Department of Biology, Syracuse University, Syracuse, New York 13244, United States

[d] Department of Cancer Immunology and Virology, Dana-Farber Cancer Institute, Boston, Massachusetts, 02115, United States

Table of Contents

Table S1. Calculated properties of natural isoprenoid alcohols and functionalized isoprenoid analogs.......7

Scheme S1: Synthetic route for the preparation of (±)-2,3-dihydrofarnesol (**22**) 8

Scheme S2: Synthetic route for the preparation of C10DHTeOPP (**1b**) and C15DHTeOPP (**2b**) 8

Table S2. ^{1}H and ^{13}C NMR peak assignments for compounds **18**, **19**, **30**, and **31** 9

Figure S1: Initial analysis of tellurium probes as substrates for *y*FTase or *r*GGTase-I..............................11

Figure S2: Isoprenoid concentration dependence of rFTase or rGGTase I-catalyzed reactions with different tellurium-containing isoprenoids. 12

Figure S3: *In vitro* prenylation of engineered VHH antibody with C10TeOPP (**1a**) and FTase 12

Figure S4: Quantitative analysis of western blots used to determine the extent of H-Ras and Rap1B prenylation after inhibition with 25 μM lovastatin and subsequent treatment with different isoprenoids..13

Figure S5: Competition experiment of tellurium probes **1a** and **2a** against C15AlkOPP in COS-7...........14

Figure S6: Mass cytometry analysis of AML-3 cells treated with C10TeOPP (**1a**)...................................15

Scheme S3: Synthetic route for the preparation of BCN-TAMRA (**35**)...16

Scheme S4: Synthetic route for the preparation of BCN-Biotin sulfone (**37**)..16

Table S3: Summary of proteomic data 16

Figure S7: Substrate competition and enzymatic inhibition assays of tellurium probes analyzed by in-gel fluorescence with SDS-PAGE 17

Figure S8: Mass cytometry analysis of AML-3 cells treated with C10TeOPP (**1a**) or C10DHTeOPP (**1b**) 18

Figure S9: Comparison of the metabolic labeling pattern obtained in COS-7 cells using C15AlkOPP or tellurium-containing probes..18

Figure S10: Mass cytometry analysis of C15TeOPP (**2a**) and C15DHTeOPP (**2b**) labeling in COS-7 over a concentration range...19

Figure S11: Mass cytometry analysis of COS-7 cells subjected to metabolic labeling with C10TeOPP (**1a**) or C10DHTeOPP (**1b**) in the absence or presence of Tipifarnib 19

Figure S12: Mass cytometry analysis of WT L6 and Atg7 KO cells with tellurium-containing probes.....20

Figure S13. Proteomic analysis using C15AlkOPP showing prenylated proteins detected in WT L6 cells and Atg7 KO cells..20

Table S4. Antibodies used for mass cytometry analysis 21

Figure S14. Marker levels obtained via mass cytometry after dimensional reduction...............................22

Figure S15. Heat maps of marker levels after dimensional reduction...22

Materials and Methods for Biological Experiments 23

Suspension Cell Culture, Probe Treatment and Harvesting 23

Adherent Cell Culture, Probe Treatment and Harvest....24
Mass Cytometry Sample Preparation....24
Mass Cytometry Data Acquisition and Normalization....25
Mass Cytometry Manual Data Gating....25
In-gel Fluorescence Analysis....25
Enrichment of Probe Labeled Proteins and On-bead Digestion....26
Isobaric Labeling of Peptides and Proteomic Sample Preparation....27
LC-MS^3 Data Acquisition....27
Proteomic Data Processing....28
In Vitro Prenylation Reaction of Nanobody....28
Initial Analysis of Prenyltransferase Activity with Te-Containing Analogues....29
Analysis of Enzymatic Activity of **1a** and **2a** to Determine Kinetic Parameters....29
General Materials and Instrumentation for Synthetic Procedures....30
Synthesis of Tellurophene Precursor (**7**)....31
Synthesis of C10TeOPP (**1a**) and C15TeOPP (**2a**)....32
Synthesis of C10DHTeOPP (**1b**) and C15DHTeOPP (**2b**)....37
Synthesis of BCN-TAMRA (**35**)....46
Synthesis of BCN-Biotin sulfone (**37**)....46
Spectral Characterization of Synthetic Compounds....48
Compound **1a** ^{1}H NMR in D_2O with water suppression....48
Compound **1a** ^{1}H NMR in D_2O....49
Compound **1a** ^{1}H NMR in DMSO-*d*6....50
Compound **1a** ^{1}H NMR in CD_3OD....51
Compound **1a** ^{1}H-^{1}H COSY NMR in DMSO-*d*6....52
Compound **1a** ^{1}H-^{1}H COSY NMR in CD_3OD....53
Compound **1a** ^{13}C NMR in D_2O....54
Compound **1a** ^{31}P NMR in D_2O with Na_2HPO_4 as internal standard....55
Compound **1a** HR-ESI-MS spectrum....56
Compound **1b** ^{1}H NMR in D_2O with water suppression....57
Compound **1b** ^{1}H NMR in DMSO-*d*6....58
Compound **1b** ^{13}C NMR in D_2O....59
Compound **1b** ^{31}P NMR in D_2O with Na_2HPO_4 as internal standard....60
Compound **1b** HR-ESI-MS spectrum....61

Compound **2a** ^{1}H NMR in D_2O with water suppression....62
Compound **2a** ^{1}H NMR in D_2O 63
Compound **2a** ^{1}H NMR in CD_3OD....64
Compound **2a** ^{1}H-^{1}H COSY NMR in CD_3OD....65
Compound **2a** ^{13}C NMR in D_2O 66
Compound **2a** ^{31}P NMR in D_2O with Na_2HPO_4 as internal standard....67
Compound **2a** HR-ESI-MS spectrum 68
Compound **2b** ^{1}H NMR in D_2O with water suppression 69
Compound **2b** ^{1}H NMR in D_2O 70
Compound **2b** ^{13}C NMR in D_2O 71
Compound **2b** ^{13}C DEPT135 NMR in D_2O 72
Compound **2b** ^{31}P NMR in D_2O with Na_2HPO_4 as internal standard 73
Compound **2b** HR-ESI-MS spectrum 74
Compound **16** ^{1}H NMR in $CDCl_3$....75
Compound **16** ^{13}C NMR in $CDCl_3$....76
Compound **16** ^{13}C DEPT90 NMR in $CDCl_3$....77
Compound **16** ^{13}C DEPT135 NMR in $CDCl_3$....78
Compound **16** ^{1}H-^{1}H COSY NMR in $CDCl_3$....79
Compound **16** ^{1}H-^{13}C HSQC NMR in $CDCl_3$....80
Compound **17** ^{1}H NMR in $CDCl_3$....81
Compound **17** ^{13}C NMR in $CDCl_3$....82
Compound **17** ^{13}C DEPT90 NMR in $CDCl_3$....83
Compound **17** ^{13}C DEPT135 NMR in $CDCl_3$....84
Compound **17** ^{1}H-^{1}H COSY NMR in $CDCl_3$....85
Compound **17** ^{1}H-^{13}C HSQC NMR in $CDCl_3$....86
Compound **18** ^{1}H NMR in $CDCl_3$....87
Compound **18** ^{13}C NMR in $CDCl_3$....88
Compound **18** ^{13}C DEPT90 NMR in $CDCl_3$....89
Compound **18** ^{13}C DEPT135 NMR in $CDCl_3$....90
Compound **18** ^{1}H-^{1}H COSY NMR in $CDCl_3$....91
Compound **18** ^{1}H-^{13}C HSQC NMR in $CDCl_3$....92
Compound **19** ^{1}H NMR in $CDCl_3$....93
Compound **19** ^{13}C NMR in $CDCl_3$....94

Compound **19** ^{13}C DEPT90 NMR in $CDCl_3$ 95
Compound **19** ^{13}C DEPT135 NMR in $CDCl_3$ 96
Compound **19** ^{1}H-^{1}H COSY NMR in $CDCl_3$ 97
Compound **19** ^{1}H-^{13}C HSQC NMR in $CDCl_3$ 98
Compound **24** ^{1}H NMR in $CDCl_3$ 99
Compound **24** ^{13}C NMR in $CDCl_3$ 100
Compound **24** ^{13}C DEPT90 NMR in $CDCl_3$ 101
Compound **24** ^{13}C DEPT135 NMR in $CDCl_3$ 102
Compound **24** ^{1}H-^{1}H COSY NMR in $CDCl_3$ 103
Compound **24** ^{1}H-^{13}C HSQC NMR in $CDCl_3$ 104
Compound **24** ^{1}H-^{13}C HMBC NMR in $CDCl_3$ 105
Compound **25** ^{1}H NMR in $CDCl_3$ 106
Compound **25** ^{13}C NMR in $CDCl_3$ 107
Compound **25** ^{13}C DEPT90 NMR in $CDCl_3$ 108
Compound **25** ^{13}C DEPT135 NMR in $CDCl_3$ 109
Compound **26** ^{1}H NMR in $CDCl_3$ 110
Compound **26** ^{13}C NMR in $CDCl_3$ 111
Compound **26** ^{13}C DEPT90 NMR in $CDCl_3$ 112
Compound **26** ^{13}C DEPT135 NMR in $CDCl_3$ 113
Compound **26** ^{1}H-^{1}H COSY NMR in $CDCl_3$ 114
Compound **26** ^{1}H-^{13}C HSQC NMR in $CDCl_3$ 115
Compound **26** ^{1}H-^{13}C HMBC NMR in $CDCl_3$ 116
Compound **27** ^{1}H NMR in $CDCl_3$ 117
Compound **27** ^{13}C NMR in $CDCl_3$ 118
Compound **27** ^{13}C DEPT90 NMR in $CDCl_3$ 119
Compound **27** ^{13}C DEPT135 NMR in $CDCl_3$ 120
Compound **27** ^{1}H-^{1}H COSY NMR in $CDCl_3$ 121
Compound **27** ^{1}H-^{13}C HSQC NMR in $CDCl_3$ 122
Compound **27** ^{1}H-^{13}C HMBC NMR in $CDCl_3$ 123
Compound **28** ^{1}H NMR in $CDCl_3$ 124
Compound **28** ^{13}C NMR in $CDCl_3$ 125
Compound **29** ^{1}H NMR in $CDCl_3$ 126
Compound **29** ^{13}C NMR in $CDCl_3$ 127

Compound **30** ^{1}H NMR in $CDCl_3$ 128
Compound **30** ^{13}C NMR in $CDCl_3$ 129
Compound **30** ^{13}C DEPT90 NMR in $CDCl_3$ 130
Compound **30** ^{13}C DEPT135 NMR in $CDCl_3$ 131
Compound **30** ^{1}H-^{1}H COSY NMR in $CDCl_3$ 132
Compound **30** ^{1}H-^{13}C HSQC NMR in $CDCl_3$ 133
Compound **30** ^{1}H-^{13}C HMBC NMR in $CDCl_3$ 134
Compound **31** ^{1}H NMR in $CDCl_3$ 135
Compound **31** ^{13}C NMR in $CDCl_3$ 136
Compound **31** ^{13}C DEPT90 NMR in $CDCl_3$ 137
Compound **31** ^{13}C DEPT135 NMR in $CDCl_3$ 138
Compound **31** ^{1}H-^{1}H COSY NMR in $CDCl_3$ 139
Compound **31** ^{1}H-^{13}C HSQC NMR in $CDCl_3$ 140
Compound **31** ^{1}H-^{13}C HMBC NMR in $CDCl_3$ 141
Compound **32** ^{1}H NMR in $CDCl_3$ 142
Compound **32** ^{13}C NMR in $CDCl_3$ 143
Compound **33** ^{1}H NMR in $CDCl_3$ 144
Compound **33** ^{13}C NMR in $CDCl_3$ 145
Compound **33** ^{13}C DEPT135 NMR in $CDCl_3$ 146
Compound **33** ^{1}H-^{1}H COSY NMR in $CDCl_3$ 147
Compound **33** ^{1}H-^{13}C HSQC NMR in $CDCl_3$ 148
References 149

Table S1. Calculated properties of natural isoprenoid alcohols and functionalized isoprenoid analogs. Calculated logP (ClogP) values were obtained using ChemDraw. Molecular volumes were calculated using Schrödinger volume_calc.py in ligand mode. "Δ Size" values represent the difference in calculated volume relative to farnesol (ΔFOH) or geranylgeraniol (ΔGGOH).

		ClogP	Calculated Volume (Å^3)	Δ FOH	Δ GGOH
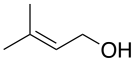	Isopentenyl alcohol	0.938	86.0	-122	-180
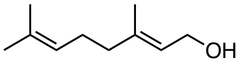	Geraniol	2.969	148.0	-60.0	-118.0
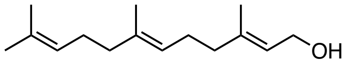	Farnesol	5.000	208.0	\	-58.0
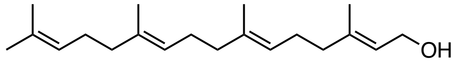	Geranylgeraniol	7.031	266.0	58.0	\
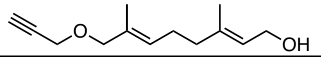	C10AlkOH	2.358	190.0	-18.0	-76.0
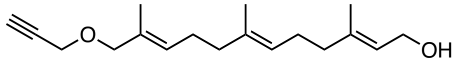	C15AlkOH	4.389	253.0	45.0	-13.0
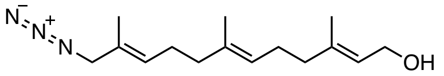	C15AziOH	5.473	234.0	26.0	-32.0
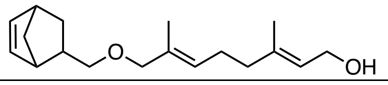	C10NorOH	4.199	242.0	34.0	-24.0
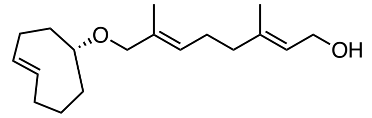	C10TCOOH	4.423	252.0	44.0	-14.0
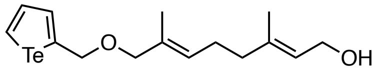	C10TeOH	**2.792**	**226.0**	**18.0**	**-40.0**
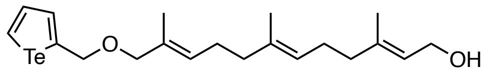	C15TeOH	**4.823**	**288.0**	**80.0**	**22.0**
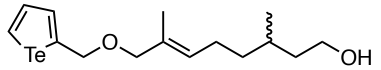	C10DHTeOH	3.076	219.0	11.0	-47.0
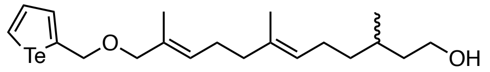	C15DHTeOH	5.107	298.0	90.0	32.0
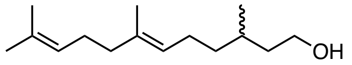	Dihydro Farnesol	5.284	207.0	-1.0	-59.0
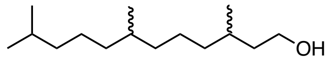		6.252	214.0	6.0	-52.0
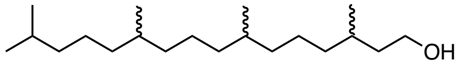		8.767	273.0	65.0	7.0
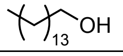		6.642	216.0	8.0	-50.0
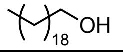		9.287	274.0	66.0	8.0

Scheme S1: Synthetic route for the preparation of (±)-2,3-dihydrofarnesol (**22**). This strategy was adapted from work by Arpicco et al.[1]

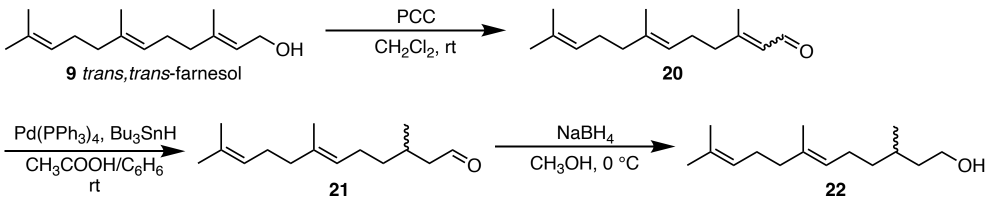

Scheme S2: Synthetic route for the preparation of C10DHTeOPP (**1b**) and C15DHTeOPP (**2b**).

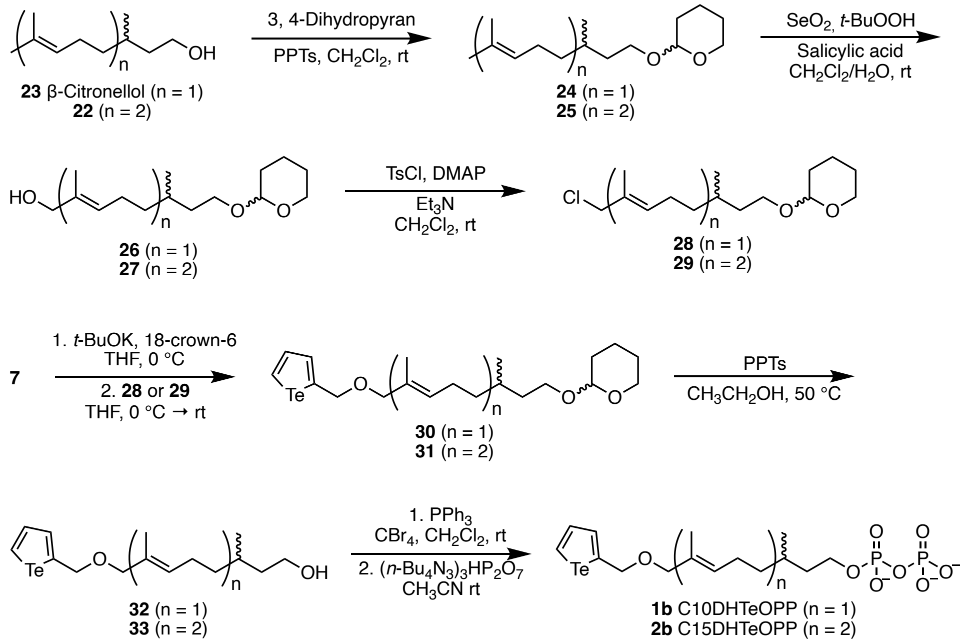

Table S2. ^{1}H and ^{13}C NMR peak assignments for compounds **18**, **19**, **30**, and **31**.[a]

**A**	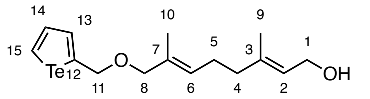 Compound **18**		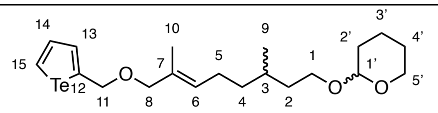 Compound **30**	
**Carbon #**	**^{13}C Shift (type)**	**^{1}H Shift (multiplicity)**	**^{13}C Shift (type)**	**^{1}H Shift (multiplicity)**
1	59.55 (CH_2)	4.15 (t)	62.50 & 62.48 (CH_2)[b]	3.87 (m), 3.50 (m)[c]
2	128.03 (CH)	5.41 (m)	36.80 & 36.74 (CH_2)[b]	1.41 (m), 1.23 (m)[c]
3	133.68 (C_q)	\	29.85 & 29.83 (CH)[b]	1.55 (m)
4	39.21 (CH_2)	2.09 (m)	37.03 & 36.89 (CH_2)[b]	1.41 (m), 1.23 (m)[c]
5	26.04 (CH_2)	2.19 (m)	25.30 & 25.26 (CH_2)[b]	2.06 (m)
6	123.91 (CH)	5.41 (m)	129.14 (CH)	5.40 (dt)
7	139.38 (C_q)	\	131.80 (C_q)	\
8	71.87 (CH_2)	4.63 (s)	71.76 (CH_2)	4.62 (s)
9	14.24 (CH_3)	1.67 (s)	19.78 & 19.68 (CH_3)	0.91 (d)
10	16.38 (CH_3)	1.69 (s)	14.14 (CH_3)	1.67 (s)
11	76.27 (CH_2)	3.95 (s)	76.43 (CH_2)	3.95 (s)
12	149.50 (C_q)	\	149.64 (C_q)	\
13	132.40 (CH)	7.46 (m)	133.60 (CH)	7.46 (m)
14	137.18 (CH)	7.69 (dd)	137.16 (CH)	7.68 (dd)
15	125.56 (CH)	8.83 (dd)	125.43 (CH)	8.82 (dd)
1’			99.13 & 98.97 (CH)	4.57 (dt)
2’			30.95 (CH_2)	1.55 (m)
3’			25.66 (CH_2)	1.64 (m)
4’			19.86 & 19.84 (CH_2)	1.82 (m), 1.71 (m)[c]
5’			66.09 & 65.99 (CH_2)	3.78 (m), 3.40 (m)[c]

[a]Assignments are based on analysis of the 1D ^{1}H and ^{13}C spectra including 2D data where applicable.

[b]For compound **30**, the compound exists as a mixture of diastereomeric species due to two stereogenic centers; accordingly, two resonances are assigned to a single carbon.

[c]The assignment of two proton NMR resonances in a single methylene group arises from diastereotopic protons.

**B**	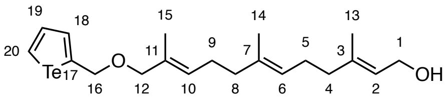 Compound **19**		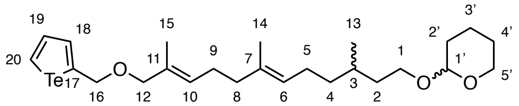 Compound **31**[c]	
**Carbon #**	**^{13}C Shift (type)**	**^{1}H Shift (multiplicity)**	**^{13}C Shift (type)**	**^{1}H Shift (multiplicity)**
1	59.56 (CH_2)	4.15 (t)	62.49 & 62.47 (CH_2)[c]	3.87 (m), 3.50 (m)[d]
2	123.55 (CH)	5.41 (m)	37.39 & 37.26 & 36.84 & 36.76 (CH_2)[b]	1.39 (m), 1.19 (m)[d]
3	139.85 (C_q)[a]	\	29.87 & 29.82 (CH)[c]	1.54 (m)
4	39.66 or 39.39 (CH_2)[b]	2.12 (m) or 2.04 (m)[b]	37.39 & 37.26 & 36.84 & 36.76 (CH_2)[b]	1.39 (m), 1.19 (m)[d]
5	26.44 or 26.40 (CH_2)[b]	2.12 (m) or 2.04 (m)[b]	26.45 (CH_2)	2.05 (m)
6	124.27 (CH)	5.14 (m)	125.20 (CH)	5.12 (t)
7	135.12 (C_q)[a]	\	131.91 (C_q)	\
8	39.66 or 39.39 (CH_2)[b]	2.12 (m) or 2.04 (m)[b]	39.45 (CH_2)	2.05 (m)
9	26.44 or 26.40 (CH_2)[b]	2.12 (m) or 2.04 (m)[b]	25.53 (CH_2)	2.05 (m)
10	128.57 (CH)	5.41 (m)	128.72 (CH)	5.40 (t)
11	131.97 (C_q)[a]	\	134.51 & 134.49 (C_q)[c]	\
12	71.73 (CH_2)	4.62 (s)	71.66 (CH_2)	4.61 (s)
13	16.43 (CH_3)	1.68 (s)	19.83 & 19.73 (CH_3)[c]	0.90 (d)
14	16.14 (CH_3)	1.62 (s)	14.17 (CH_3)	1.68 (s)
15	14.20 (CH_3)	1.68 (s)	16.08 (CH_3)	1.61 (s)
16	76.36 (CH_2)	3.96 (s)	76.40 (CH_2)	3.96 (s)
17	149.61 (C_q)	\	149.66 (C_q)	\
18	133.62 (CH)	7.46 (m)	133.56 (CH)	7.46 (m)
19	137.17 (CH)	7.68 (dd)	137.15 (CH)	7.68 (dd)
20	125.53 (CH)	8.83 (dd)	125.48 (CH)	8.82 (dd)
1’			99.11 & 98.95 (CH)[c]	4.57 (dt)
2’			30.96 (CH_2)	1.72 (m), 1.54 (m)[d]
3’			25.67 (CH_2)	1.64 (m)
4’			19.83 & 19.73 (CH_2)[c]	1.83 (m), 1.72 (m)[d]
5’			66.15 & 66.04 (CH_2)[c]	3.78 (m), 3.41 (m)[d]

[a]For compound **19**, the ^{13}C assignments for the quaternary carbons C3, C7, and C11 are provisional, as unambiguous differentiation of these signals is not possible based on the available data.

[b]Due to spectral overlap, it is not possible to unambiguously assign this position.

[c]For compound **31**, the compound exists as a mixture of diastereomeric species due to two stereogenic centers; accordingly, two resonances are assigned to a single carbon.

[d]The assignment of two proton NMR resonances in a single methylene group arises from diastereotopic protons.

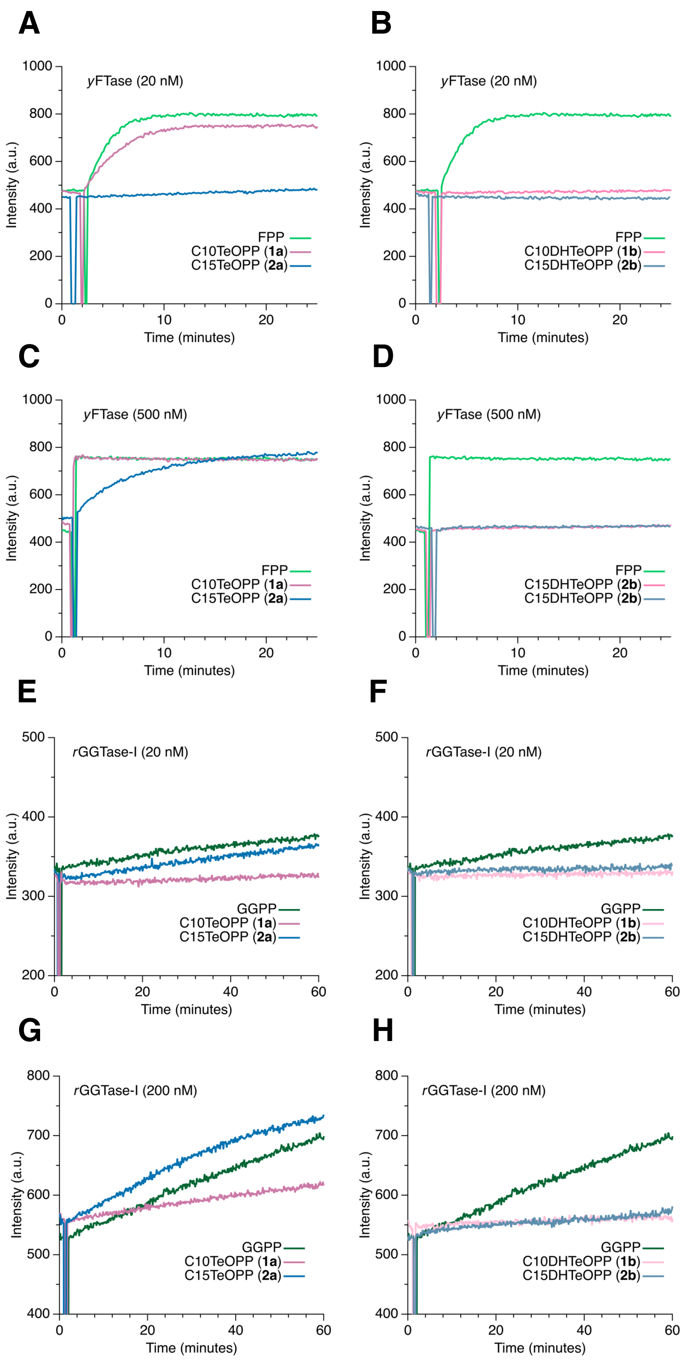

Figure S1: Initial analysis of tellurium probes as substrates for *y*FTase (Panel A - D) or *r*GGTase-I (Panel E - H). Each reaction contained 10 µM FPP, GGPP, or a tellurium probe, with 2.4 µM Ds-GCVLS for assays using *y*FTase or Ds-GCVLL for assays using *r*GGTase-I. Enzyme concentrations were held at 20 nM (panels A, B, E and F), 500 nM (panels C and D) or 200 nM (panels G &H). Experiments with *y*FTase were performed in duplicate, with one representative progress curve shown here. Experiments with *r*GGTase-I were performed once.

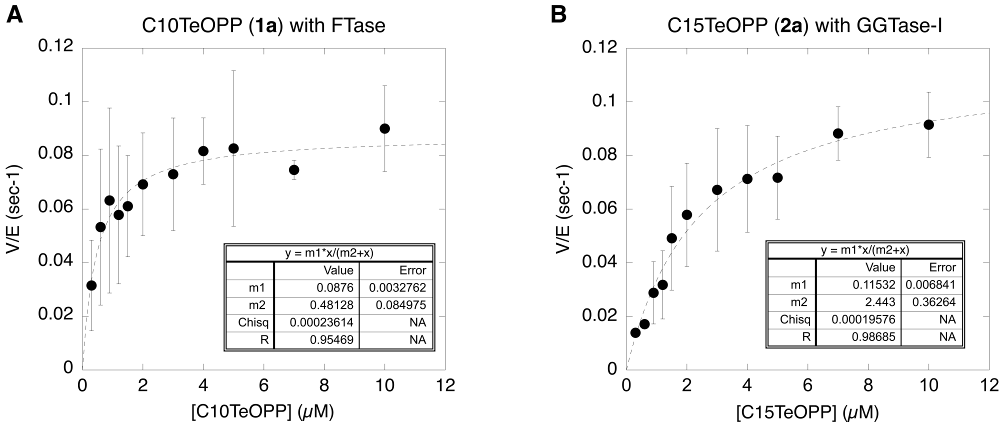

Figure S2: Isoprenoid concentration dependence of rFTase or rGGTase I-catalyzed reactions with different tellurium-containing isoprenoids. (A) Analysis of C10TeOPP (**1a**) as a substrate for FTase; (B) Analysis of C15TeOPP (**2a**) as a substrate for *r*GGTase-I. Reactions with FTase were performed with Ds-GCVLS while reactions using GGTase-I were performed with Ds-GCVLL. Note: Since these are bi-substrate reactions and were only performed at a single peptide concentration, the kinetic constants reported are apparent values.

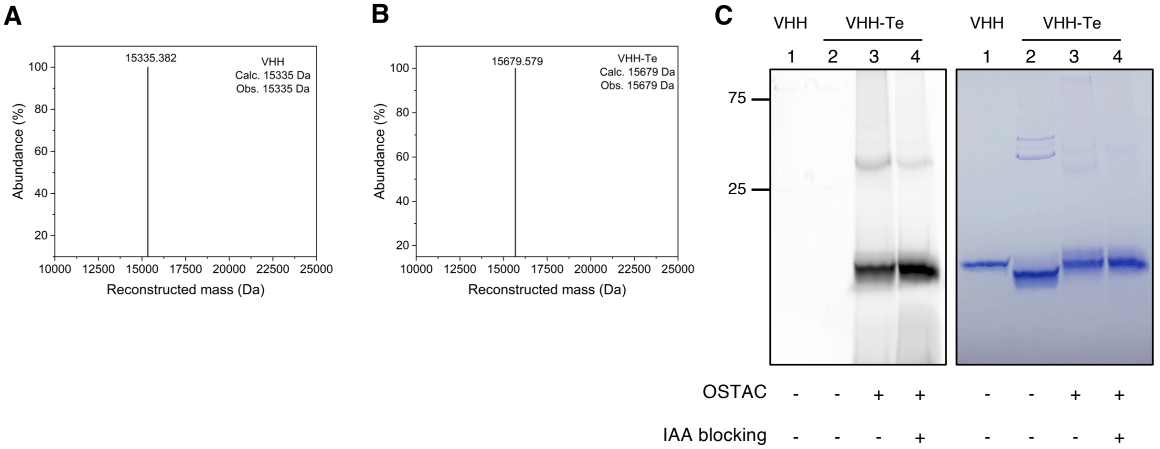

Figure S3: *In vitro* prenylation of engineered VHH antibody with C10TeOPP (**1a**) and FTase. (A and B) LC-MS analysis of VHH nanobody before (panel A) and after prenylation reaction (panel B) showing the desired mass shift (Δ m/z = 344 Da); (C) SDS-PAGE analysis of VHH antibody before (Lane 1) and after prenylation (Lane 2). The resulting VHH-Te conjugate was also subjected to OSTAC reaction with 25 µM BCN-TAMRA and in-gel fluorescence analysis (Lanes 3 and 4). In lane 4, VHH-Te was pre-treated with iodoacetamide (5 mM, 20 min) prior to the OSTAC reaction. The gel was scanned with a fluorescent gel scanner (Typhoon FLA 9500, GE Healthcare) for detection of TAMRA fluorescence (left panel), then stained with Coomassie blue (right panel).

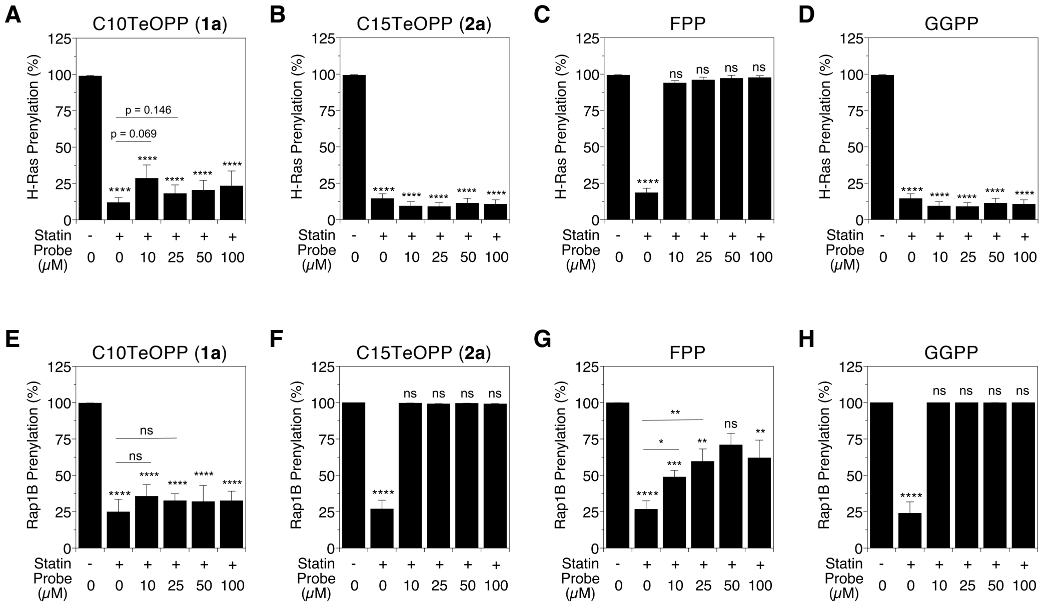

Figure S4: Quantitative analysis of western blots used to determine the extent of H-Ras (Panel A – D) and Rap1B (Panel E – F) prenylation after inhibition with 25 µM lovastatin and subsequent treatment with different isoprenoids. COS-7 cells were treated with lovastatin 1.5 h (except for the sample indicated by -) followed by supplementation with different isoprenoids for 18 h while retaining the statin. Cells were then lysed, the lysates fractionated via SDS-PAGE, blotted to PVDF membranes and visualized via western blotting followed by imaging and quantitative analysis. (A and E) Cells treated with C10TeOPP (**1a**); (B and F) Cells treated with C15TeOPP (**2a**); (C and G) Cells treated FPP; (D and H) Cells treated with GGPP. These experiments were performed with four biological replicates and the average values graphed with the standard error of the mean (SEM) indicated by error bars, in panel F and G, error bars are present but too small to be visible at the scale shown. The statistical analysis shown compares the extent of prenylation in the sample observed in the absence of lovastatin treatment (first column in each graph) with that observed under the conditions indicated. Symbol meaning: ns: $p > 0.05$; *: $p \leqslant 0.05$; **: $p \leqslant 0.01$; ***: $p \leqslant 0.001$; ****: $p \leqslant 0.0001$. The p values for comparisons between samples treated with statin in the absence versus presence of 10 µM or 20 µM C10TeOPP analyzed for H-Ras prenylation (Panel A) were also calculated and found to be 0.069 and 0.146, respectively. This means the 10 µM C10TeOPP sample is just outside the 95% confidence interval (93%) and hence has some, but limited significance. The p values for comparisons between samples treated with statin in the absence versus presence of 10 µM or 20 µM C10TeOPP analyzed for Rap1B prenylation (Panel E) were also calculated and found to be 0.224 and 0.167, respectively. Therefore, these samples are even outside the 90% confidence interval and hence lack any statistical significance. Finally, the p values for comparisons between samples treated with statin in the absence versus presence of 10 µM or 20 µM FPP analyzed for Rap1B prenylation (Panel G) were also calculated and found to be 0.048 and 0.006, respectively. Hence, these differences are statistically significant. This last result can be attributed to the fact that isoprenoid production is not completely eliminated using 25 µM statin and that sufficient amounts of IPP are available to elongate the exogenous FPP to GGPP, providing some rescue.

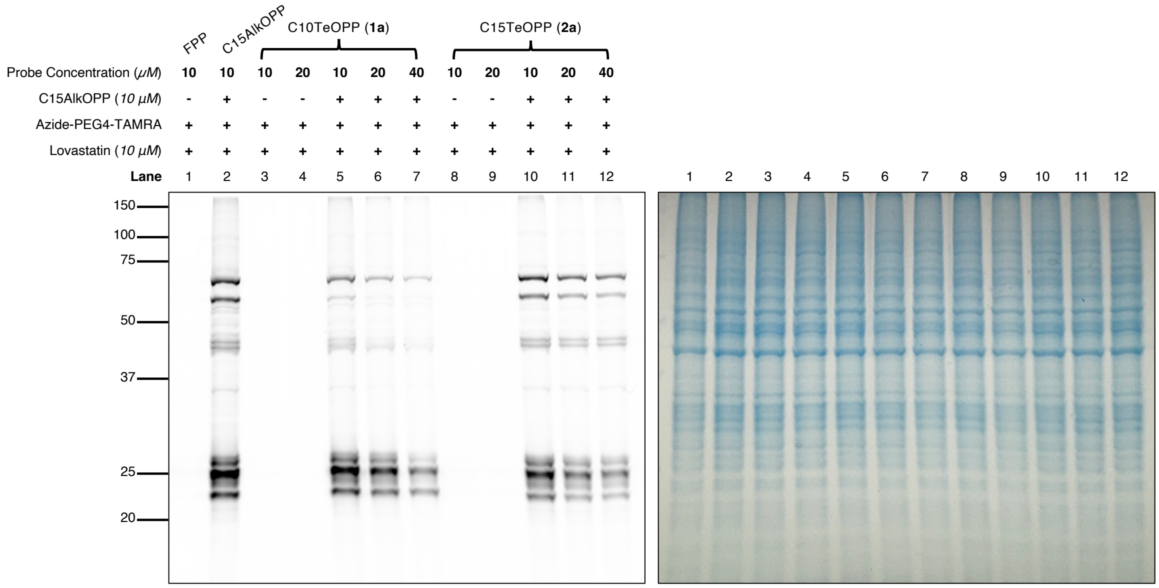

Figure S5: Competition experiment of tellurium probes **1a** and **2a** against C15AlkOPP in COS-7. Cells were first subjected to a Lovastatin treatment for 4 h to inhibit the endogenous FPP/GGPP synthesis. Cells were then co-treated with 10 μM C15AlkOPP and various concentrations of tellurium probes **1a** or **2a** for 24 h while retaining the lovastatin in the media, followed by cell lysis and CuAAC reaction with 25 μM TAMRA-Azide with the resulting cell lysate. The lysates were then resolved via 12% SDS-PAGE and scanned with a fluorescent gel scanner (Typhoon FLA 9500, GE Healthcare) for detection of TAMRA fluorescence (left panel) followed by staining with Coomassie blue to show total protein loading (right panel).

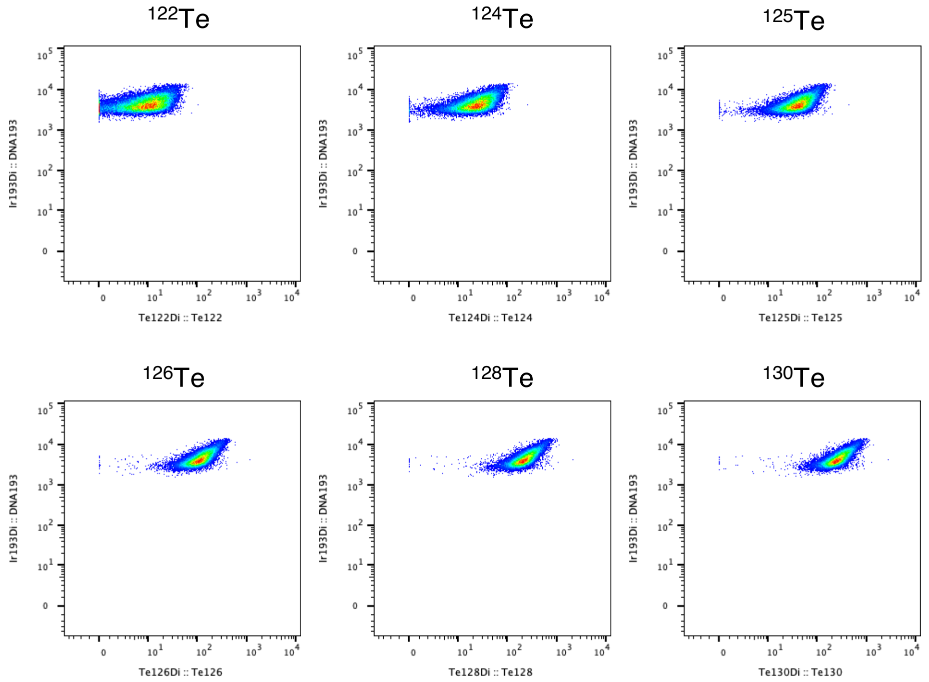

Figure S6: Mass cytometry analysis of AML-3 cells treated with C10TeOPP (**1a**). Data from each of the major tellurium isotope channels is shown. The ^{193}Ir channel was used to identify single cells in these graphs.

Scheme S3: Synthetic route for the preparation of BCN-TAMRA (**35**).

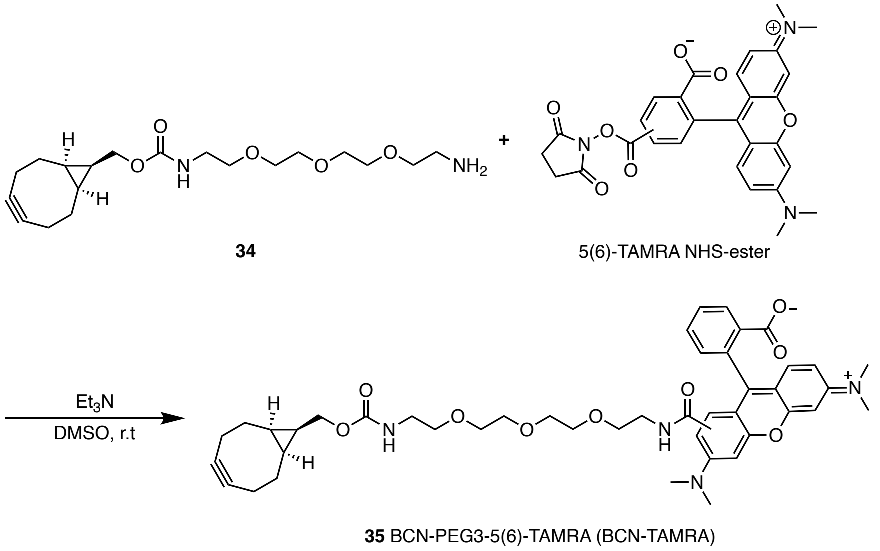

Scheme S4: Synthetic route for the preparation of BCN-Biotin sulfone (**37**).

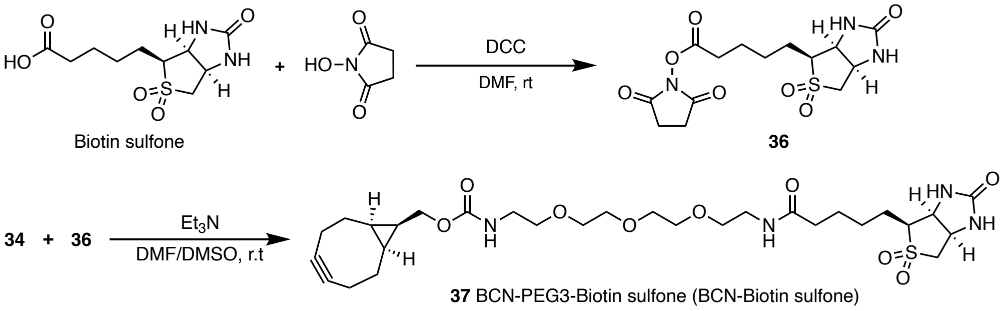

Table S3: Summary of proteomic data.

Comprehensive lists of proteins identified across all proteomic experiments are provided in **Table S3**, as a separate supplementary .xlsx file named "Table S3_Te_Proteomic summary"

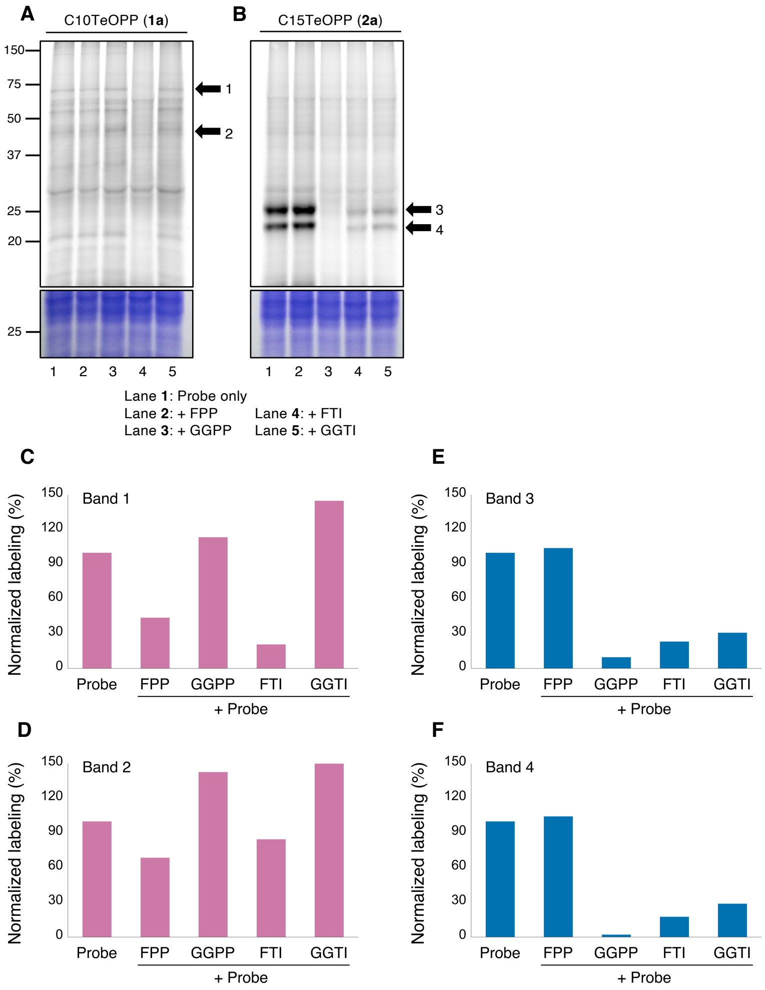

Figure S7: Substrate competition and enzymatic inhibition assays of tellurium probes analyzed by in-gel fluorescence with SDS-PAGE. Data are shown for C10TeOPP (**1a**, panel A) and C15TeOPP (**2a**, panel B). Densitometry was performed in ImageJ on two bands from panel A (band 1, panel C; band 2, panel D) and two bands from panel B (band 3, panel E; band 4, panel F). Cells were treated with probes (10 µM) for 24 h. Natural isoprenoids FPP or GGPP (substrate competitors) or enzyme inhibitors were used at 10 µM and co-treated with the tellurium probes. FTI: Tipifarnib; GGTI: GGTI-298.

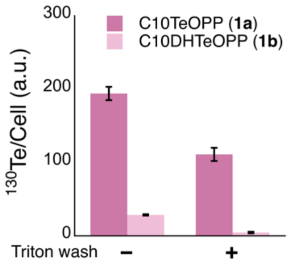

Figure S8: Mass cytometry analysis of AML-3 cells treated with C10TeOPP (**1a**) or C10DHTeOPP (**1b**). Cells were treated with 10 µM of 1a or its non-reactive analog 1b for 24 h. For each replicate, half of the sample was subjected to a wash using 0.1% Triton X-100 in PBS (w/v) following fixation and permeabilization while the other half was not.

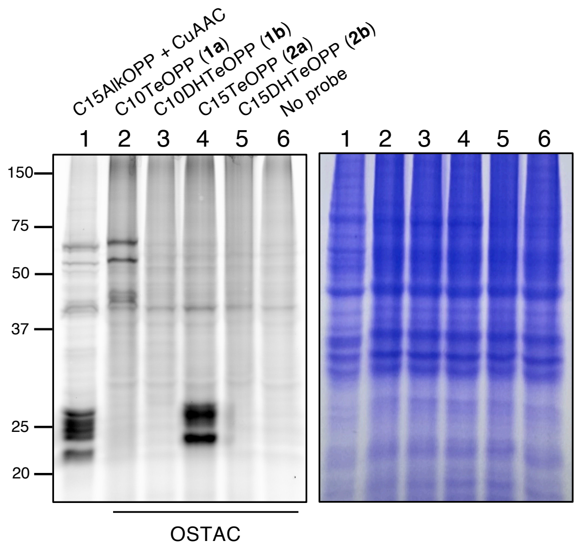

Figure S9: Comparison of the metabolic labeling pattern obtained in COS-7 cells using C15AlkOPP or tellurium-containing probes. Cells were treated with probes (10 µM) for 24 h, followed by cell lysis and CuAAC reaction for the C15AlkOPP treated sample (Lane 1) with 25 µM TAMRA-Azide, or OSTAC reaction for tellurium-containing probe treated samples (Lane 2-5) with 25 µM BCN-TAMRA; The "no probe" sample (Lane 6, indicated by -) was subjected to the same OSTAC reaction. Left panel: TAMRA fluorescence scan; Right panel: Coomassie blue staining showing total protein loading.

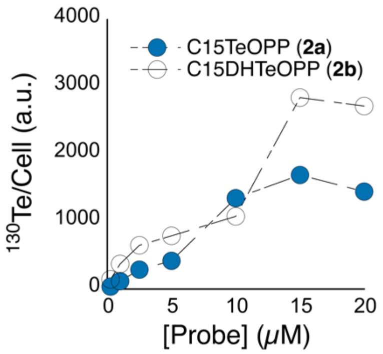

Figure S10: Mass cytometry analysis of C15TeOPP (**2a**) and C15DHTeOPP (**2b**) labeling in COS-7 over a concentration range. Cells were treated with probe or the dihydro counterpart for 24 h and processed using the standard CyTOF workflow.

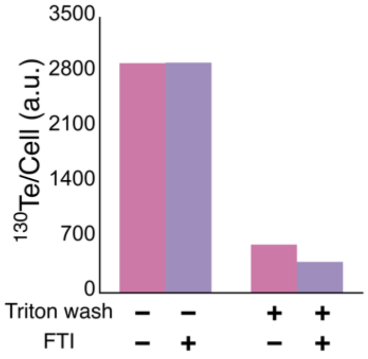

Figure S11: Mass cytometry analysis of COS-7 cells subjected to metabolic labeling with C10TeOPP (**1a**) or C10DHTeOPP (**1b**) in the absence or presence of Tipifarnib (10 μM). Cells were co-treated with probe or the dihydro counterpart (10 μM) for 24 h. For each replicate, half of the sample was subjected to a wash using 0.1% Triton X-100 in PBS (w/v) following fixation and permeabilization while the other half was not.

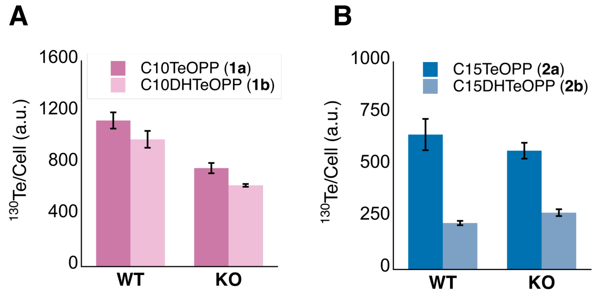

Figure S12: Mass cytometry analysis of WT L6 and Atg7 KO cells with tellurium-containing probes. Cells were treated with the probe or the dihydro counterpart (5 μM) for 24 h. Panel A: Results obtained using C10TeOPP (**1a**) or C10DHTeOPP (**1b**). Panel B: Results obtained with C15TeOPP (**2a**) or C15DHTeOPP (**2b**).

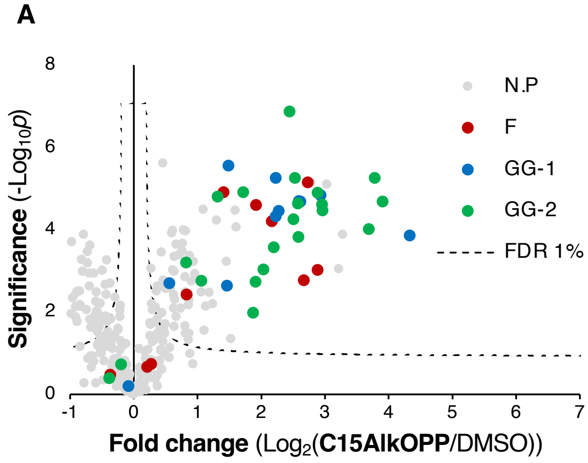

Figure S13. Proteomic analysis using C15AlkOPP showing prenylated proteins detected in WT L6 cells and Atg7 KO cells. (A) Results obtained using WT L6 cells. (B) Results obtained using Atg7 KO cells. For these experiments, the labeling of cells treated with probe (in triplicate) was compared to the labeling obtained in control samples treated with DMSO (also in triplicate). For each probe, prenylomic analysis was carried out by labeling the 6 samples (3 probe-treated samples and 3 control samples) with TMT 6-plex reagents followed by mixing and quantitative MS^3 analysis. Volcano plots were generated from a two-tailed t-test comparing the normalized TMT reporter ion intensities across three biological replicates per condition, using FDR = 1% and s0 = 0.1. Color scheme: FTase substrates (red); GGTase-I substrates (blue); GGTase-II substrates (green); proteins not reported as prenylation substrates (grey).

Table S4. Antibodies used for mass cytometry analysis.

Target	Tag	Manufacturer	Clone (Catalog if Polyclonal)
LC3B	142Nd	Novus	Polyclonal (NB100-2220)
ATG5	149Sm	Novus	Polyclonal (NB110-53818)
ATG7	160Gd	Abcam	EPR6251
LAMP2	162Dy	Sigma	Polyclonal (L0668)
HMGCR	172Yb	Abcam	
Rabbit Isotype[a]	173Yb	Invitrogen	Polyclonal (02-6102)

[a]The rabbit isotype was included to measure nonspecific antibody interactions and was not included in the subsequent data analysis.

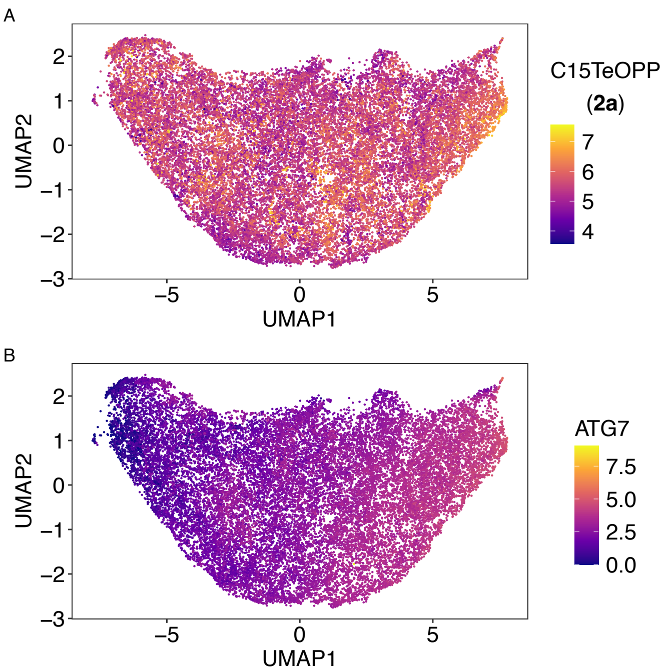

Figure S14. Marker levels obtained via mass cytometry after dimensional reduction. (A) Heat map of marker levels obtained with the C15TeOPP probe. (B) Heat map of marker levels obtained with the Atg7 antibodies.

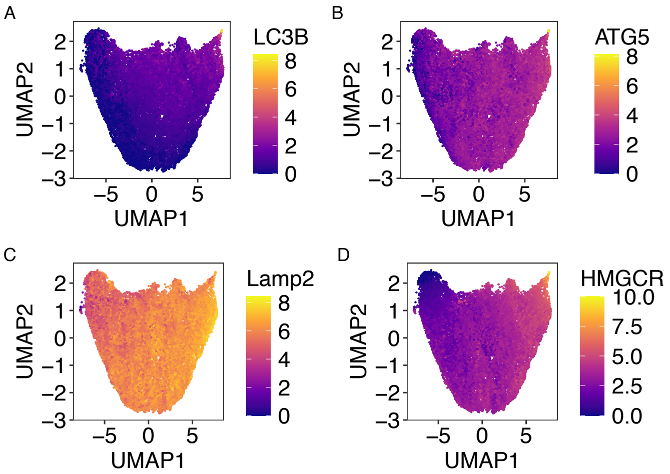

Figure S15. Heat maps of marker levels after dimensional reduction. Levels of (A) LC3B, (B) Atg5, (C) Lamp2, and (D) HMGCR are presented on UMAP plots.

Materials and Methods for Biological Experiments

For all mass cytometry experiments and gel-based experiments, aqueous solutions were prepared using ultrapure water obtained from a Milli-Q system (Millipore, 13-14 MΩ·cm at 25 °C) unless otherwise noted. For the proteomic experiments, all solvents used were LC–MS grade and purchased from Supelco or Fisher Scientific and all aqueous solutions were prepared using LC–MS grade water. All procedures were performed at room temperature unless otherwise noted.

Roswell Park Memorial Institute 1640 (RPMI-1640) medium with 25 mM 2-[4-(2-hydroxyethyl)piperazin-1-yl]ethanesulfonic acid (HEPES), Dulbecco's Modified Eagle Medium (DMEM), Dulbecco's Modified Eagle Medium/nutrient mixture F-12 medium (DMEM/F-12), insulin-transferrin-selenium (ITS, 100X), dexamethasone (10 mM in DMSO), penicillin-streptomycin-glutamine (100X), penicillin-streptomycin, trypsin (2.5%), Dulbecco's phosphate-buffered saline (DPBS) and phosphate-buffered saline (10x, pH 7.4) were purchased from Gibco. Pierce 16% formaldehyde (w/v, methanol-free), BCA protein assay kits, phenylmethylsulfonyl fluoride (PMSF) protease inhibitor, Pierce NeutrAvidin agarose resin (capacity: ~20 µg Biotin per mL resin, supplied as 50% slurry), Pierce spin columns, LC-MS grade formic acid, TMT 10-plex isobaric label reagent set (1 x 0.8 mg per tag, 90100, Lot # AA401548) and 50% hydroxylamine buffer were purchased from Thermofisher. Fetal bovine serum (FBS), saponin, Triton x-100, Benzonase nuclease, protease inhibitor cocktail, protein standard (200 mg/mL of BSA), ProteoExtract protein precipitation kit, iodoacetamide, tris(2-carboxyethyl)phosphine hydrochloride (TCEP), *N*-chlorosuccinimide, 1 M triethylammonium bicarbonate (TEAB) buffer, ammonium formate and Empore SPE disks (polystyrene-divinylbenzene, SDB-XC) were purchased from Sigma-Aldrich. Heat-inactivated fetal bovine serum (HI-FBS) was purchased from Genesee Scientific. Tris[(1-benzyl-1H-1,2,3-triazol-4-yl)methyl]amine (TBTA), Lovastatin, Tipifarnib and GGTI-298 (trifluoroacetate salt) were purchased from Cayman Chemical. Each compound was dissolved in DMSO for experiments. Cisplatin was obtained either as Cell-ID Cisplatin solution (5 mM) from Standard Biotools, or as a pharmaceutical secondary standard in solid form (PHR1624, Supelco). The latter was solubilized using DMSO to prepare a 5 mM aliquoted stock solution, which was stored in -20 °C. Cell-ID Intercalator-Ir (125 µM), Maxpar fix and perm buffer, and EQ four element calibration beads were purchased from Standard Biotools. Phosphate buffered saline containing sodium azide (10x, pH 7.2, 0.2 M potassium phosphate, 1.5 M sodium chloride, 0.1% (w/v) sodium azide/NaN_3) was purchased from Rockland and was diluted to PBS-A (PBS with NaN_3) using MQ water for any mass cytometry sample preparation. Bovine serum albumin (BSA) was purchased from Jackson ImmunoResearch. Cell staining buffer (CSB) was made in-house with PBS-A and 0.5% (w/v) BSA and stored at 2 – 8 °C. PBS/Tween-20 10x solution (contains 0.5% Tween 20, P21200) was purchased from Research Products International and was diluted to PBS-T using MQ water. Sodium dodecyl sulfate, urea, ammonium persulfate, 30% acrylamide and bis-acrylamide solution (29:1), and 4x Laemmli sample buffer were purchased from Bio-rad. Dithiothreitol (DTT) was obtained from Fisher Scientific. Sequencing grade modified trypsin was purchased from Promega. MassPREP ADH digestion standard was obtained from Waters. TAMRA-PEG3-Azide (TAMRA-Azide, CAS: 1228100-59-1) was purchased from Broadpharm.

Suspension Cell Culture, Probe Treatment and Harvesting

AML-3 cell and MOLM-13 cell (human acute myeloid leukemia cell lines) were obtained from Dr. David Fruman at the University of California, Irvine. Cells were cultured in RPMI-1640 medium supplied with

25 mM HEPES, 10% (v/v) HI-FBS and 1% (v/v) of 100x penicillin-streptomycin-glutamine stock, and were maintained in a humidified incubator with 5% CO_2 at 37 °C. Cells were maintained at a density between 0.5 x 10^6 and 2.0 x 10^6 cells/mL in T75 vented-cap flasks, counted and diluted every 2 days to maintain logarithmic growth. Cells were allowed to grow for 7 days before any experiment was performed.

For mass cytometry, in-gel fluorescence, and proteomic experiments, cells were first pelleted at 250 x *g* for 5 min, resuspended in fresh medium to a density between 0.8 x 10^6 and 1.0 x 10^6 cells/mL, and transferred to a T25 or T75 flask, followed by probe treatment and/or lovastatin, prenyltransferase inhibitor, or prenyltransferase competitor treatment prior to probe treatment when needed. Cells were harvested via centrifugation at 250 x *g* for 5 min.

Adherent Cell Culture, Probe Treatment and Harvest

COS-7 cell (African green monkey kidney fibroblast-like cell line), AML-12 cell (alpha mouse liver 12 cell line) and L6 cell (rat skeletal muscle myoblast cell line) were purchased from ATCC. Autophagy related protein 7 knockout (Atg7 KO) in the L6 cell line was performed using the CRISPR-Cas9 gene editing system.[2] COS-7, wild type (WT) L6 and Atg7 KO L6 cells were cultured in DMEM medium supplied with 10% (v/v) FBS and 1% (v/v) 10,000 U/mL penicillin-streptomycin. AML-12 cell was cultured in DMEM/F-12 medium supplied with 10% (v/v) FBS, 10 µg/mL insulin, 5.5 µg/mL transferrin, 5 ng/mL selenium, 40 ng/mL dexamethasone, and 1% (v/v) of 100X penicillin-streptomycin stock. All adherent cells were cultured on 100 mm tissue culture-treated dishes. Cells were maintained in a humidified incubator with 5% CO_2 at 37 °C and were passaged every 2 days by using 0.25% trypsin in DPBS to lift the cells. Cells were counted using a Hausser Scientific Bright-Line hemacytometer.

For mass cytometry and in-gel fluorescence experiments, cells were seeded at a density between 0.8 x 10^6 and 1.0 x 10^6 cells per 100 mm plate with 10 mL of fresh medium. Cells were allowed to adhere to the plates overnight. After that, the medium was aspirated and replaced with 5 mL of fresh medium, followed by probe treatment and/or prenyltransferase inhibitor or prenyltransferase competitor treatment when needed. For mass cytometry experiments, cells were harvested using 0.25% trypsin in DPBS to lift the cells. For in-gel fluorescence experiments, cells were harvested using a cell scraper and DPBS to collect the cells. Cells were pelleted via centrifugation at 250 x *g* for 5 min.

Mass Cytometry Sample Preparation

Unless otherwise noted, cells were centrifuged at 250 x *g* for 5 min at rt before fixation with formaldehyde, and centrifuged at 800 x *g* for 5 min at rt after fixation; supernatants were removed by aspiration after centrifugation. Harvested cells were pelleted in 15 mL or 50 mL falcon tubes. Cells were washed with 1.0 x 10^6 cells per mL of PBS-T and pelleted, which was then resuspended in 1.0 x 10^6 cells per mL of PBS-A. Cisplatin solution (1 µL per 1.0 x 10^6 cells) was added. Cell suspensions were incubated for precisely 1 min at rt, followed by quenching with 5 mL per 1.0 x 10^6 cells of CSB. Cells were pelleted and fixed in 1 mL per 1.0 x 10^6 cells of 4% formaldehyde in PBS-A for 15 min at rt on a rotator. Fixed cells were resuspended with PBS-T, transferred to 1.5 mL Eppendorf tubes at 1.0 x 10^6 per tube and pelleted. Cell permeabilization was done with 0.1% saponin (w/v) in PBS-A (1 mL) for 10 min at rt on a rotator. For samples subjected to

Triton wash, permeabilized cells were resuspended in 0.1% Triton x-100 (w/v) in PBS-A (1 mL) for 15 min at rt on a rotator followed by washing with PBS-A (1 mL). DNA intercalator staining was performed by resuspending cells in 1 mL of Maxpar fix and perm buffer supplied with 62.5 nM of Cell-ID Intercalator-Ir overnight at 4 °C. On the day of analysis, cells were washed once with 1 mL of CSB and three time with 1 mL of MQ water. If analysis could not be performed the next day, samples were pelleted after DNA intercalator staining, maintained in fix and perm buffer, and stored at 2–8 °C for up to one week.

Mass Cytometry Data Acquisition and Normalization

Washed cell pellets were resuspended in 1.0 – 1.5 mL of HPLC grade water with 10% EQ four element calibration beads immediately before analysis. Data was recorded on a Fluidigm (now Standard Biotools) CyTOF2 mass cytometer with instrument settings of 30 s acquisition delay, 10 s detector stability delay, noise reduction on, lower convolution threshold of 200, event subtraction of 0, min event duration of 10, max event duration of 150, sigma equals to 3, found event limit of 0, sample loop size equal to 0.5 mL, injection speed 0.045 mL/min. For most experiments reported in this study, only the FCS file was retained and no IMD file was preserved. Data normalization of FCS files was performed on CyTOF software version 6.7.1014 with bead passport EQ-P13H2302_ver2.

Mass Cytometry Manual Data Gating

Data gating was performed in FlowJo using the following steps: (1) selecting events that are high in both ^{191}Ir and ^{193}Ir (both from Cell-ID Intercalator-Ir) for cell identification; (2) selecting events high for ^{193}Ir and low in ^{151}Eu (from EQ four element calibration beads) to exclude cell-bead aggregates; (3) selecting events positive for ^{193}Ir and event length less than or equal to 0.700 ms to exclude potential cell-cell doublets; (4) plotting ^{193}Ir versus ^{195}Pt (from cisplatin) and selecting population with low ^{195}Pt signal, indicating intact membrane integrity and cell viability. Subsequent analysis of mass cytometry data was performed using the median signal intensity of each marker within the gated cell populations.

In-gel Fluorescence Analysis

Each 300 µL aliquot of cell lysis buffer was prepared in PBS and contained 1% SDS, 5 µL of protease inhibitor cocktail, 0.64 µL 1 mM PMSF in DMSO, and 0.26 µL Benzonase nuclease. Cell pellets were resuspended in 300 µL of lysis buffer and lysed by sonication (6–8 pulses, 3 s each, intensity setting 6, with 5 s rest between pulses) using a Fisher Scientific 60 Sonic Dismembrator, while samples were kept on ice. Concentration of protein was determined using BCA assay kits following the manufacturer's protocol. A series of standard protein solutions (0.2, 0.4, 0.8, 1.2, 1.6, and 2.0 mg/mL) was prepared in PBS containing 1% SDS, using a protein standard from Sigma-Aldrich, and was used to calibrate the protein concentration assay.

For oxidation-controlled, strain-promoted tellurophene–alkyne cycloaddition (OSTAC) reactions, 100 µg of protein from cell lysate (quantified by BCA assay) was used and diluted to a final concentration of 1.0 µg/µL. Following each reagent addition, reaction tubes were rotated at rt while being protected from

light. A fresh solution of iodoacetamide (IAA) was prepared in aqueous ammonium bicarbonate buffer (pH 8.0) immediately before use. NCS was dissolved in water at 25 mM with the aid of sonication immediately before use. DBCO-OH and BCN-TAMRA were dissolved in DMSO to a concentration of 5 mM and 10 mM respectively and both stock solutions were aliquoted and stored at -20 °C until use. OSTAC reactions were performed in the following order, with final reagent concentrations and time lengths as indicated: IAA alkylation (1.3 µL of 375 mM solution, 5 mM final) for 20 min, first oxidation with NCS (2.0 µL of 25 mM solution, 500 µM final) and blocking with DBCO-OH (1.0 µL of 5 mM solution, 50 µM final) for 5 min, followed by labeling with BCN-TAMRA (2.5 µL of 1 mM solution, 25 µM final) in the presence of additional NCS (1 µL of 25 mM solution, 250 µM final) for 5 min. Each reaction was quenched by precipitating the proteins using ProteoExtract protein precipitation kits following the manufacturer's protocol.

For copper-catalyzed azide–alkyne cycloaddition (CuAAC) reactions, 100 µg of protein from cell lysate (quantified by BCA assay) was used and diluted to a final concentration of 1.0 µg/µL. The reaction was performed by adding the following reagents sequentially with final concentrations as indicated: TAMRA-N_3 (2.5 µL of 1 mM stock solution in DMSO, 25 µM final), TCEP (2 µL of freshly prepared 50 mM solution in DMSO, 1 mM final), TBTA (2 µL of 10 mM stock solution in DMSO, 200 µM final), and $CuSO_4$ (2 µL of 50 mM aqueous stock solution, 1 mM final). Reaction tubes were rotated at rt while being protected from light for 90 min. The reaction was quenched by precipitating the proteins using ProteoExtract protein precipitation kits following the manufacturer's protocol.

Cell pellets obtained after protein precipitation were air-dried and subsequently dissolved in 40 µL of 50% 4x Laemmli buffer and 50% 0.5 M DTT solution. Samples were heated to 95 °C for 5 min, and 12–15 µL of each sample was loaded onto 12% SDS–PAGE gels. The volume of sample loaded was held consistent across all lanes within each gel. Electrophoresis was performed at 120 V for 120 min or until the tracking dyes had completely migrated out of the gel. TAMRA fluorescence was detected using a Typhoon FLA 9500 instrument (GE Healthcare), followed by Coomassie blue staining to visualize total protein loading. Gel images were processed in ImageJ for whole-image contrast and brightness adjustment.

Enrichment of Probe Labeled Proteins and On-bead Digestion

Cell lysis and protein concentration determination were performed using the same procedures as described in the "In-gel fluorescence analysis" section. For the OSTAC-based proteomic experiments, 2.0 mg of protein from each lysate sample was diluted to a final concentration of 2.0 µg/µL and was subjected to the reaction with final reagent concentrations and time lengths as indicated: IAA alkylation (13.3 µL of 375 mM stock, 5 mM final) for 20 min, first oxidation with NCS (20.0 µL of 25 mM solution, 500 µM final) and blocking with DBCO-OH (10.0 µL of 5 mM solution, 50 µM final) for 5 min, followed by labeling with BCN-Biotin sulfone (10.0 µL of 10 mM solution, 100 µM final) in the presence of additional NCS (10 µL of 25 mM solution, 250 µM final) for 5 min. Reactions were quenched by precipitating the proteins using 8 mL of $CHCl_3$/CH_3OH/PBS (1:4:3 v/v/v) and centrifugation at 4,400 x *g* for 10 min. Supernatants were decanted and the resulting protein disks were air-dried and stored in -20 °C overnight followed by resolubilization in 500 µL of PBS containing 1% SDS with the aid of sonication. Protein concentrations were measured using BCA assays.

Capture of biotinylated proteins was performed in 2 mL protein low-bind Eppendorf tubes. NeutrAvidin agarose resin slurry (200 µL) was added to each tube and the resin was washed using PBS containing 1% SDS(3 x 1 mL) by vigorous shaking and vortexing, followed by brief centrifugation. After allowing the resin to settle for 2 min, the supernatant was carefully removed and discarded. Biotinylated protein samples were diluted to 1.0 µg/µL with PBS containing 1% SDS, 1,000 µg of protein was added to the pre-washed resin and incubated with rotation for 120 min. The supernatant was removed after brief centrifugation and the resin was washed using PBS containing 1% SDS (3 x 1 mL) as described above, followed by one wash with PBS (1 mL). Nonspecifically bound proteins were removed by washing the resin with 8 M urea in 50 mM TEAB (3 x 1 mL), followed by washing with 50 mM TEAB (3 x 1 mL). The resin was then resuspended in 50 mM TEAB (100 µL) and the bound proteins were digested with trypsin (1.5 µg, reconstituted with provided buffer to 0.25 µg/µL) overnight at 37 °C. Digestion was quenched by the addition of 20% HCO_2H/H_2O (v/v, 2.5 µL). The flow-through containing the desired peptides was collected using Pierce spin columns; The resin was washed with 0.5%. HCO_2H/H_2O (v/v, 1 x 100 µL) and 30% CH_3CN/H_2O (v/v, 2 x 100 µL). All flow-throughs from each sample were pooled and lyophilized.

Isobaric Labeling of Peptides and Proteomic Sample Preparation

Lyophilized peptide samples were dissolved in 40 µL of 100 mM TEAB. Peptide concentrations were determined via BCA assay, with a set of protein standards (0.2, 0.4, 0.8, 1.2, 1.6, and 2.0 mg/mL) prepared in 100 mM TEAB. For each TMT reaction, 10 µg of peptides were added to a 0.5 mL protein low-bind Eppendorf tube and diluted to 0.5 µg/µL using 100 mM TEAB. Each sample was supplemented with 150 fmol of ADH digestion standard as an internal standard. TMT reagents were disolved in anhydrous grade CH_3CN following the manufacturer's protocol and 10 µL of TMT reagent solution was added to each sample. TMT 126, 127N and 127C channels were assigned to FPP-treated samples while TMT 128N, 128C and 129N were assigned to C10TeOPP-treated samples and TMT 129C, 130N and 130C were assigned to C15TeOPP-treated samples. Reaction tubes were rotated at rt for 120 min. Reactions were quenched by the addition of 2.5 µL of 5% NH_3OH in H_2O, followed by an additional 15 min of rotation. TMT-labeled peptides were combined, and the tubes were rinsed with 100 mM TEAB (2 x 50 µL). The rinse solutions were pooled with the labeled peptides and lyophilized.

Dried multiplexed peptides were then dissolved in 200 mM aqueous $[NH_4][HCO_2]$ (300 µL, pH 10) and subjected to fractionation using in-house prepared stage tips, packed with three SDB-XC disks that are 1.07 mm i.d x 0.50 mm thickness, in a 200 µL pipette tip. Peptides were fractionated under high-pH reversed-phase conditions with increasing concentrations of CH_3CN in 200 mM aqueous $[NH_4][HCO_2]$ (pH 10) (5%, 10%, 15%, 20%, 22.5%, 27.5% and 80%, v/v, 60 µL for each concentration), resulting in 7 fractions. Two of the fractions (5% and 10% fractions) were combined. Each fraction was lyophilized and redissolved in 0.1% HCO_2H in H_2O (v/v, 30 µL) for LC-MS^3 analysis.

LC-MS^3 Data Acquisition

Fractionated TMT-labeled peptides were analyzed using an Orbitrap Fusion tribrid mass spectrometer (Thermofisher) at the Masonic Cancer Center Mass Spectrometry Laboratory, University of Minnesota. Peptides were separated using an in-house packed C-18 reversed-phase column (75 µm i.d x 45 cm) with a

flow rate of 300 nL/min. Each fraction was analyzed using a 115-min gradient ranging from 0% to 90% of buffer B (CH_3CN with 0.1% HCO_2H) and buffer A (H_2O with 0.1% HCO_2H) and the eluted peptides were directly introduced into the mass spectrometer via electrospray ionization. MS1 scans were collected at 120,000 orbitrap resolution, scan range (m/z) between 320 and 2000, 100 ms maximum injection time, and automatic gain control (AGC) target of 200,000. MS2 scans were collected with collision-induced dissociation (CID) at a normalized collision energy (NCE) of 35%, isolation window of 1.2 m/z, 100 ms maximum injection time, AGC target of 5,000, and 10 ms activation time. Acquisition in MS3 was done by selecting the top 10 precursors for high-collisional energy dissociation (HCD) fragmentation in the orbitrap with 55% NCE, MS2 isolation window of 2 m/z, 120 ms maximum injection time, AGC target of 5,000, and scan range (m/z) between 100 and 500.

Proteomic Data Processing

Raw MS^3 files were uploaded into MaxQuant (version 2.5.1.0) and searched against a nonredundant human database (UP0000000589) from Uniprot. The following parameters were modified: Trypsin/P was selected for digestion with 3 missed cleavages allowed and minimum peptide length of 7 residues; protein FDR was set to 0.5; carbamidomethylation (applied only for the proteomic experiment performed with tellurium probes **1a** and **2a**), oxidation (M) and acetyl (protein N-term) modifications were selected; unique + razor peptides were used for quantification. MaxQuant was run through the MaxQuantCmd.exc on the Agate high performance computing cluster at University of Minnesota Supercomputing Institute. The proteingroup.txt file generated was then processed in Perseus (version 2.0.11). Proteins that were only identified by site, potential contaminants or having reversed peptides were removed. Raw intensity values were $\log_2$-transformed. Proteins with less than 3 out of 9 values for each TMT channel after transformation were removed. Missing values were imputed from the normal distribution derived from the remaining data. Reporter ion (TMT) values were normalized by mean-centering rows and median-centering columns. Statistical analysis was performed using a two-sample *t*-test with a false discovery rate (FDR) of 5% and s0 value of 0.1. Results were then exported to Microsoft Excel for plot generation.

***In Vitro* Prenylation Reaction of Nanobody**

Reactions (1mL) were carried out in Prenylation Buffer (50 mM Tris-HCl, pH = 7.5; 20 mM KCl; 10 mM $MgCl_2$; 5 mM DTT; and 10 μM $ZnCl_2$ final). Prior to initiating the reaction, the solution was incubated on ice for 30 min with 5 μM VHH-CVIA to ensure complete reduction of thiol groups. C10TeOPP **1a** (dissolved $[NH_4][HCO_3]$ (25 mM, pH 10)) was then added to a final concentration of 30 μM, followed by the addition of yeast farnesyltransferase (*y*FTase) at a final concentration of 400 nM. The reaction mixture was incubated in a water bath at 32 °C for 6 h. To remove excess isoprenoid analog, buffer exchange against PBS was performed using Amicon ultrafiltration units with a 10 kDa MWCO. For each exchange cycle, 14.5 mL of fresh PBS was added, and the solution was concentrated to 0.5 mL by centrifugation at 5,000 x *g* for 30 min. This process was repeated three times. The resulting protein solution was analyzed using a 15% SDS-PAGE gel.

Initial Analysis of Prenyltransferase Activity with Te-Containing Analogues

Reactions (500 μL) were carried out in Prenylation Buffer (see above) supplemented with 0.04% (w/v) *n*-dodecyl β-d-glucopyranoside (DDM) prepared in H_2O. Dansylated peptides were added to a final concentration of 2.4 μM, followed by the addition of FPP, GGPP or isoprenoid probes to the desired concentrations. Fluorescence measurements were performed using a Varian Cary Eclipse Fluorescence Spectrophotometer (λ_{ex} = 340 nm, λ_{em} = 505 nm, excitation and emission slit widths = 10 nm). Reactions were initiated by the adding the prenyltransferase enzyme to the desired concentration and the reactions were allowed to proceed for up to 60 min.

Analysis of Enzymatic Activity of 1a and 2a to Determine Kinetic Parameters

The reactivity of tellurium probes **1a** and **2a** were determined with *r*FTase and *r*GGTase-I using dansylated peptide Ds-GCVLS and Ds-GCVLL, respectively, in a previously reported fluorescence-based prenylation assay in 96-well black low adhesion plates.[3]

Molecular Volume Calculation Using Schrödinger

Molecular volumes were calculated using the "volume_calc.py" utility implemented in the Schrödinger software suite. Structures of molecules were drawn in Maestro 2D Sketcher, and energy-minimized using the OPLS4 force field via LigPrep. Volume calculations were performed in ligand mode, in which the volume of the entire molecule is computed, and no spatial constraints or grid-bounding boxes are applied. Molecular volumes were determined by numerical integration on a three-dimensional Cartesian grid. Atomic Van Der Waals radii were assigned according to the Bondi radius set, and hydrogen atoms were excluded from the volume calculation. Overlapping atomic volumes were treated as a single continuous volume. A grid resolution of 1.0 Å was used for all calculations. The total molecular volume was obtained by summing the occupied grid elements and is reported in units of Å^3.

General Materials and Instrumentation for Synthetic Procedures

All reactions described here were performed at rt, protected under a nitrogen atmosphere, and magnetically stirred unless otherwise noted. All water used in synthetic procedures was in-house deionized water unless otherwise noted. All reagents and solvents were purchased from commercial sources (Sigma Aldrich, Oakwood chemical, Fisher Scientific) and used directly without purification unless otherwise noted. Anhydrous grade solvents, geraniol, β-citronellol, tellurium metal (granular, −5-+50 mesh, 99.99% trace metals basis), polymer-bound triphenylphosphine (100-200 mesh, extent of labeling: ~1 - 1.5 mmol/g capacity), $[(n\text{-Bu})_4]_3P_2HO_7$, AmberChrom 50WX8 ion exchange resin (hydrogen form, 50-100 mesh), and cellulose (fibers, medium) were purchased from Sigma Aldrich. *Trans, trans*-farnesol was purchased from Oakwood Chemical. *Endo*-BCN-PEG3-amine (CAS: 1883512-27-3) and 5(6)-TAMRA NHS ester (CAS: 150810-69-8) were purchased from Broadpharm. Biotin sulfone (CAS: 40720-05-6) was purchased from Cayman Chemical. Thin layer chromatography was performed on pre-coated TLC sheets (0.20 mm silica gel 60 with fluorescent indicator UV_{254}) purchased from Macherey-Nagel and visualized using $KMNO_4$ stain, or under UV light for tellurophene-containing compounds. TLC plates were typically developed using 20-25% EtOAc in hexanes (v/v). Flash column chromatography was performed with silica gel (technical grade, pore size 60 Å, 230-400 mesh particle size, 40-63 μm particle size) purchased from Sigma Aldrich.

Deuterated NMR solvents ($CDCl_3$, D_2O, CD_3OD and DMSO-*d*6) were purchased from Cambridge Isotope Laboratories, Inc. Ammonium-*d*4 deuteroxide solution (25 wt. % in D_2O, 99 atom % D) was purchased from Oakwood Chemical. ^{1}H NMR and ^{13}C NMR with proton decoupling were recorded using a 500 MHz Bruker Avance III HD with SampleXpress (500 MHz for ^{1}H NMR and 125 MHz for ^{13}C NMR). All spectra were referenced to the peak resulting from incomplete solvent deuteration with the exception of ^{13}C NMR of diphosphate compounds which were obtained using D_2O as solvent. All reactions reported in this study were performed at least in duplicate to ensure reproducibility. For previously reported compounds, spectral data obtained were in good agreement with literature reports. For the synthesis of **1b** and **2b**, due to the presence of two stereocenters, compound **26 - 31** exists as a mixture of diastereomers. Consequently, the ^{1}H NMR spectra of these intermediates display broadened and partially overlapping resonances. In addition, the ^{13}C NMR spectra exhibit an increased number of carbon signals relative to the number expected for a single stereoisomer, consistent with the presence of diastereomeric species. No attempt was made to separate individual diastereomers, as these compounds were carried forward directly to subsequent deprotection and diphosphorylation steps.

^{31}P NMR spectra were recorded on a 400 MHz Bruker Avance III HD with SampleXpress (162 MHz). All diphosphate compounds were dissolved in aqueous 25 mM NH_4HCO_3 for NMR analysis and any subsequent experiments. The concentrations of the diphosphate compound solutions were measured using ^{31}P NMR with an internal standard made with Na_2HPO_4 in D_2O. A typical ^{31}P NMR sample was prepared by mixing 50 or 100 μL of diphosphate solution with 50 or 100 μL of Na_2HPO_4 standard solution, 50 μL of ammonium-*d*4 deuteroxide solution, and 500 μL of D_2O.

HRMS spectra of all synthetic compounds were recorded on Agilent 7200 GC/QTOF-MS, Bruker BioTOF II ESI/TOF-MS, or Sciex X500R UPLC/QTOF-MS instruments. Measured values are reported to 4 decimal places and are within 5 ppm of the calculated values. All calculated mass values are based on the most abundant isotope. For tellurium-containing compounds, ^{130}Te was used for mass calculations.

Synthesis of Tellurophene Precursor (7)

5-(triisopropylsilyl)penta-2,4-diyn-1-ol (5)

(Previously reported compound)[4]

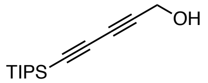

(Triisopropylsilyl) acetylene **3** (5.00 g, 27.4 mmol, 1.00 equiv) was dissolved in acetone (200 mL). $AgNO_3$ (5.61 g, 31.5 mmol, 1.15 equiv) and *N*-bromosuccinimide (4.65 g, 27.4 mmol, 1.00 equiv) were added to the solution and the mixture was stirred vigorously at rt for 3 h. Next, the reaction was quenched with H_2O (250 mL), and the mixture was extracted with hexanes (3 x 100 mL). The combined organic layers were dried over anhydrous $MgSO_4$, filtered, and then concentrated under reduced pressure to yield bromide **4**, which was used directly in the next step without further purification.

CuCl (542 mg, 5.50 mmol, 0.200 equiv) was dissolved in 30% n-butylamine in H_2O (90 mL, v/v), resulting in a blue transparent solution. Hydroxylamine hydrochloride ($NH_2OH{\cdot}HCl$, 2.29 g, 32.9 mmol, 1.20 equiv) was added to eliminate the blue color. The solution was then cooled in an ice-water bath and then propargyl alcohol (1.90 mL, 32.9 mmol, 1.20 equiv) was added slowly, resulting in a yellow opaque mixture. Bromination product **4** (27.4 mmol, assuming 100% conversion from the bromination step, 1.00 equiv) was added dropwise and the mixture was stirred at rt for 1.5 h before quenched with aqueous satd. NH_4Cl (150 mL). The mixture was exacted with EtOAc (3 x 75 mL). The combined organic layers were dried over anhydrous $MgSO_4$, filtered, and concentrated under reduced pressure. Purification via silica gel flash column chromatography and elution with 5% - 20% EtOAc in hexanes (v/v) afforded **5** as a yellow to brown oil (4.53 g, 70% yield over two steps).

Tellurophen-2-ylmethanol (7)

(Previously reported compound)[4]

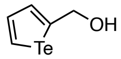

To a solution of **5** (2.24 g, 9.50 mmol, 1.00 equiv) in anhydrous THF (100 mL) maintained in an ice-water bath, tetrabutylammonium fluoride (TBAF, 1.0 M solution in THF, Sigma-Aldrich, 9.5 mL) was added dropwise. The mixture was stirred at rt for 1.5 h before being quenched with satd. NH_4Cl (150 mL). The mixture was extracted with EtOAc (3 x 75 mL). The combined organic layers were washed with 1 M aqueous citric acid (1 x 100 mL), dried over anhydrous $MgSO_4$, filtered, and concentrated under reduced pressure to yield **6** as dark yellow to brown clear oil. The crude product was used directly in the next step without further purification.

Tellurium metal (granular, −5-+50 mesh, 99.99% trace metals basis, 4.85 g, 38.0 mmol, 4.00 equiv) was ground into a fine powder and suspended in 1 M NaOH in H_2O (120 mL) under N_2 (g). Rongalite ($CH_3NaO_3S{\cdot}2H_2O$, 11.7 g, 76.0 mmol, 8.00 equiv) was then added to the reaction flask. The mixture was stirred vigorously and heated to 95 °C for 30 min, during which the color turned dark purple. After that, it was allowed to cool down to 60 °C and stirred for additional 5 min. Compound **6** (9.50 mmol, assuming

100% conversion from the deprotection step) was dissolved in degassed EtOH (8.0 mL) and then added slowly to the purple solution and the reaction was allowed to proceed for 1.5 h at 60 °C before being exposed to air and cooled down to rt. Satd. NH_4Cl (150 mL) was added, the resulting mixture was acidified using 10% HCl, filtered through Celite to remove the precipitated solids and the filtrate was extracted with EtOAc (3 x 75 mL). The combined organic layers were dried over anhydrous $MgSO_4$, filtered, and concentrated under reduced pressure. Purification using silica gel flash column chromatography and elution with 5% - 30% EtOAc in hexanes (v/v) afforded tellurophene **7** as a yellow oil (1.08 g, 54% yield).

Synthesis of C10TeOPP (1a) and C15TeOPP (2a)

(2*E*,6*E*)-2,6-dimethyl-8-((tetrahydro-2*H*-pyran-2-yl)oxy)octa-2,6-dien-1-ol (10)

(Previously reported compound)[5]

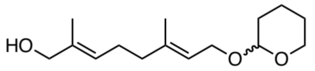

To a solution of geraniol **8** (23.0 g, 149.0 mmol, 1.00 equiv) in CH_2Cl_2 (150 mL) in a loosely capped flask, 3,4-dihydropyran (DHP, 30.0 mL, 324 mmol, 2.20 eq.) and pyridinium *p*-toluenesulfonate (PPTs, 4.90 g, 19.5 mmol, 0.13 equiv) were added. The mixture was stirred at rt for 3 h before being quenched with satd. aqueous $NaHCO_3$ (250 mL). The organic layer was separated, and the aqueous layer was further extracted with CH_2Cl_2 (2 x 100 mL). The combined organic layers were dried over $MgSO_4$ and concentrated under reduced pressure to yield **10** as colorless to yellow clear oil. The crude product was used directly in the next step.

To a solution of **10** (149.0 mmol, assuming 100% conversion from the THP-protection step) in CH_2Cl_2 (ACS grade, 200 mL) in a loosely capped flask, selenium dioxide (1.65 g, 14.9 mmol, 0.100 equiv), salicylic acid (2.06 g, 14.9 mmol, 0.100 equiv), and *tert*-Butyl hydroperoxide solution (70 wt. % in H_2O, Sigma Aldrich, 82.0 mL, 596 mmol, 4.00 equiv) were added sequentially. The mixture was stirred vigorously at rt for 12 hours before being quenched with satd. aqueous $NaHCO_3$ (300 mL). The organic layer was separated, and the aqueous layer was further extracted with CH_2Cl_2 (2 x 150 mL). The combined organic layers were dried over anhydrous $MgSO_4$, filtered, and concentrated under reduced pressure. Purification via silica gel flash column chromatography and elution with 5% - 40% EtOAc in hexanes (v/v) afforded **12** as colorless to yellow oil (11.2 g, 30% yield over two steps).

(2*E*,6*E*,10*E*)-2,6,10-trimethyl-12-((tetrahydro-2*H*-pyran-2-yl)oxy)dodeca-2,6,10-trien-1-ol (13)

(Previously reported compound)[6]

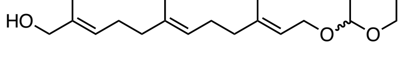

Compound **13** was synthesized using *trans, trans*-farnesol **9** (15.0 g, 67.4 mmol) in place of **8**, following the same two-step synthetic procedure used for **12**. The product was obtained as a clear yellow oil (5.54 g, 25% yield over two steps).

2-(((2*E*,6*E*)-8-chloro-3,7-dimethylocta-2,6-dien-1-yl)oxy)tetrahydro-2*H*-pyran (14)

(Previously reported compound)[7]

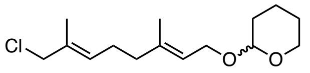

The procedure for this reaction was adapted from published work by Chappe *et. al.* [7]

Riley oxidation product **12** (2.00 g, 7.86 mmol, 1.00 equiv) was dissolved in anhydrous CH_2Cl_2 (12 mL). To the solution, 4-(dimethylamino) pyridine (DMAP) (672 mg, 5.50 mmol, 0.7 equiv), *p*-toluenesulfonyl chloride (TsCl) (1.95 g, 10.2 mmol, 1.30 equiv) and triethylamine (TEA) (1.3 mL, 9.40 mmol, 1.20 equiv) were added sequentially. The reaction was stirred at rt for 3 h, after which the reaction was quenched with H_2O (30 mL). The organic layer was separated, and the aqueous layer was further extracted with CH_2Cl_2 (2 x 30 mL). The combined organic layer was dried over anhydrous $MgSO_4$, filtered, and concentrated under reduced pressure. The crude product was purified using silica gel flash column chromatography and elution with 5% EtOAc in hexanes (v/v) to yield **14** as clear yellow oil (860 mg, 40% yield).

2-(((2*E*,6*E*,10*E*)-12-chloro-3,7,11-trimethyldodeca-2,6,10-trien-1-yl)oxy)tetrahydro-2*H*-pyran (15)

(Previously reported compound)

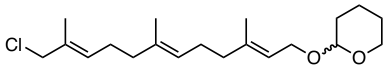

Compound **15** was synthesized using Riley oxidation product **13** (1.90 g, 5.89 mmol) in place of **12**, following the same synthetic procedure used for **14**. The product was obtained as a clear yellow oil (962 mg, 47% yield).

2-(((2*E*,6*E*)-3,7-dimethyl-8-(tellurophen-2-ylmethoxy)octa-2,6-dien-1-yl)oxy)tetrahydro-2*H*-pyran (16)

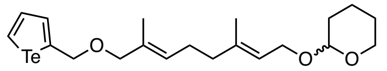

Tellurophene **7** (433 mg, 2.06 mmol, 1.00 equiv) was dissolved in anhydrous tetrahydrofuran (THF, 15 mL) and cooled in ice-bath. Potassium *tert*-butoxide (449 mg, 4.00 mmol, 1.94 equiv), 18-crown-6 (prepared as 90 mM solution in THF, 4.4 mL, 0.40 mmol, 0.20 equiv) were added sequentially. A solution of TsCl chlorination product **14** (845 mg, 3.10 mmol, 1.50 equiv) in anhydrous THF (3 mL) was then added dropwise to the reaction mixture. The mixture was stirred while maintained in ice-bath for 0.5 hour, then at r.t for additional 3 hours. The reaction was quenched with H_2O (50 mL), and the mixture was extracted with CH_2Cl_2 (3 x 30 mL). The combined organic layers were dried over anhydrous $MgSO_4$, filtered, and concentrated under reduced pressure. The crude product was purified using silica gel flash column

chromatography and elution with 5% - 10% EtOAc in hexanes (v/v) to yield **16** as yellow to faint brown oil (748 mg, 80% yield).

^{1}H NMR (500 MHz, $CDCl_3$): δ 8.82 (dd, *J* = 6.8, 1.2 Hz, 1H), 7.68 (dd, *J* = 6.8, 3.9 Hz, 1H), 7.46 (m, 1H), 5.39 (m, 2H), 4.62 (s, 2H), 4.24 (m, 1H), 4.02 (m, 1H), 3.95 (s, 2H), 3.88 (m, 1H), 3.51 (m, 1H), 2.18 (m, 2H), 2.09 (m, 2H), 1.83 (m, 1H), 1.72 (m, 2H), 1.69 (s, 3H), 1.67 (s, 3H), 1.55 (m, 4H).

^{13}C NMR (125 MHz, $CDCl_3$): δ 149.58, 139.89, 137.17, 133.63, 132.27, 128.22, 125.50, 121.10, 98.04, 76.29, 71.77, 63.82, 62.46, 39.33, 30.87, 26.11, 25.65, 19.78, 16.55, 14.19.

HRMS (ESI) m/z: Calcd for $C_{20}H_{30}O_3NaTe^+$ $[M+Na]^+$: 471.1151; found: 471.1137.

2-(((2*E*,6*E*,10*E*)-3,7,11-trimethyl-12-(tellurophen-2-ylmethoxy)dodeca-2,6,10-trien-1-yl)oxy)tetrahydro-2*H*-pyran (17)

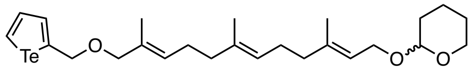

Compound **17** was synthesized using chlorinated product **15** (954 mg, 2.80 mmol) in place of **14**, following the same synthetic procedure as for **16**. The product was obtained as a clear yellow oil (721 mg, 75% yield).

^{1}H NMR (500 MHz, $CDCl_3$): δ 8.82 (dd, *J* = 6.9, 1.2 Hz, 1H), 7.68 (dd, *J* = 6.8, 3.9 Hz, 1H), 7.46 (m, 1H), 5.38 (m, 2H), 5.12 (m, 1H), 4.62 (d, *J* = 1.4 Hz, 2H), 4.23 (m, 1H), 4.01 (m, 1H), 3.96 (d, *J* = 1.1 Hz, 2H), 3.90 (m, 1H), 3.51 (m, 1H), 2.14 (m, 4H), 2.04 (m, 4H), 1.84 (m, 1H), 1.72 (m, 2H), 1.67 (s, 6H), 1.60 (s, 3H), 1.54 (m, 4H).

^{13}C NMR (126 MHz, $CDCl_3$): δ 149.65, 140.33, 137.17, 135.02, 133.60, 133.57, 131.95, 128.65, 125.46, 124.39, 120.78, 97.96, 76.39, 71.68, 63.81, 62.45, 39.77, 39.42, 30.88, 26.46, 25.66, 19.79, 16.58, 16.14, 14.19.

HRMS (ESI) m/z: Calcd for $C_{25}H_{38}O_3NaTe^+$ $[M+Na]^+$: 539.1775; found: 539.1783.

(2*E*,6*E*)-3,7-dimethyl-8-(tellurophen-2-ylmethoxy)octa-2,6-dien-1-ol (18)

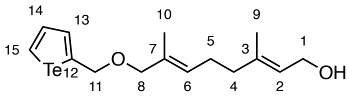

Ether-linked product **16** (569 mg, 1.28 mmol, 1.00 equiv) and PPTs (641 mg, 2.55 mmol, 2.00 equiv) were dissolved in anhydrous ethanol (8 mL) in a loosely capped flask. The mixture was heated to 50°C and stirred for 3 hours. The reaction was quenched with H_2O (50 mL) and the mixture was extracted with CH_2Cl_2 (3 x 30 mL). The combined organic layers were dried over anhydrous $MgSO_4$, filtered, and concentrated under reduced pressure. The crude product was purified using silica gel flash column chromatography and elution with 10% - 25% EtOAc in hexanes (v/v) to yield **18** as clear yellow oil (296 mg, 64% yield).

^{1}H NMR (500 MHz, $CDCl_3$): δ 8.83 (dd, *J* = 6.8, 1.2 Hz, 1H), 7.69 (dd, *J* = 6.8, 3.9 Hz, 1H), 7.46 (m, 1H), 5.41 (m, 2H), 4.63 (d, *J* = 1.4 Hz, 2H), 4.15 (t, *J* = 6.2 Hz, 2H), 3.95 (s, 2H), 2.19 (m, 2H), 2.09 (m, 2H), 1.69 (s, 3H), 1.67 (s, 3H).

^{13}C NMR (125 MHz, $CDCl_3$): δ 149.50 (C12), 139.38 (C7), 137.18 (C14), 133.68 (C3), 132.40 (C13), 128.03 (C2), 125.56 (C15), 123.91 (C6), 76.27 (C11), 71.87 (C8), 59.55 (C1), 39.21 (C4), 26.04 (C5), 16.38 (C10), 14.24 (C9).

HRMS (ESI) m/z: Calcd. for $C_{15}H_{22}O_2NaTe^+$ $[M+Na]^+$: 387.0575; found: 387.0594.

(2*E*,6*E*)-3,7-dimethyl-8-(tellurophen-2-ylmethoxy)octa-2,6-dien-1-ol (19)

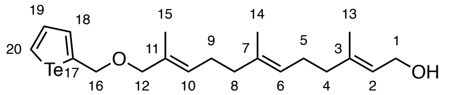

Compound **19** was synthesized using ether-linked product **17** (627 mg, 1.22 mmol) in place of **16**, following the same synthetic procedure used for **18**. The product was obtained as a clear yellow oil (348 mg, 66% yield).

^{1}H NMR (500 MHz, $CDCl_3$): δ 8.83 (dd, *J* = 6.8, 1.2 Hz, 1H), 7.68 (dd, *J* = 6.8, 3.9 Hz, 1H), 7.46 (m, 1H), 5.41 (m, 2H), 5.12 (m, 1H), 4.62 (d, *J* = 1.3 Hz, 2H), 4.15 (t, *J* = 6.1 Hz, 2H), 3.96 (d, *J* = 1.1 Hz, 2H), 2.12 (m, 4H), 2.04 (m, 4H), 1.68 (s, 6H), 1.61 (s, 3H).

^{13}C NMR (126 MHz, $CDCl_3$): δ 149.61 (C17), 139.85 (C3), 137.17 (C19), 135.12 (C7), 133.62 (C18), 131.97 (C11), 128.57 (C10), 125.53 (C15), 124.27 (C6), 123.55 (C2), 76.36 (C16), 71.73 (C12), 59.56 (C1), 39.66 & 39.39 (C4 & C8), 26.44 & 26.40 (C5 & C9), 16.43 & 16.14 (C13 & C14), 14.20 (C15).

HRMS (ESI) m/z: Calcd for $C_{20}H_{30}O_2NaTe^+$ $[M+Na]^+$: 455.1200; found: 455.1196.

(2*E*,6*E*)-3,7-dimethyl-8-(tellurophen-2-ylmethoxy)octa-2,6-dien-1-yl diphosphate (1a)

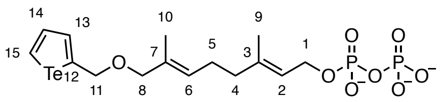

Polymer-bound triphenylphosphine (~1-1.5 mmol/g capacity, 520 mg, ~2.00 equiv of PPh_3) was suspended in anhydrous CH_2Cl_2 (10 mL) under N_2 (g) and the mixture was stirred for 10 min. A solution of THP deprotection product **18** (147 mg, 0.406 mmol, 1.00 equiv) in anhydrous CH_2Cl_2 (1 mL) was then added to the reaction flask, followed by the addition of CBr_4 (206 mg, 0.621 mmol, 1.53 equiv) solubilized in anhydrous CH_2Cl_2 (1 mL). The reaction mixture was stirred at rt for overnight. The resin was removed via filtration, and the filtrate was concentrated under reduced pressure. The resulting crude residue was redissolved in anhydrous CH_3CN (3 mL). Diphosphorylating reagent $[(n\text{-Bu})_4]_3P_2HO_7$ (476 mg, 0.527 mmol, 1.30 equiv) was added to the reaction flask and the mixture was stirred at rt for 24 h. After that, the reaction mixture was concentrated under reduced pressure to afford the crude yellow oil product.

The crude material was first subjected to an Amberchrome ion exchange column. The resin was first treated with 28-30% aqueous NH_4OH (200 mL), followed by column equilibration using 25 mM aqueous NH_4HCO_3 with 2% isopropanol (v/v) (solvent A, 250 mL). The crude product was dissolved in a minimum amount of solvent A, loaded onto the column and eluted as a single fraction using solvent A (200 mL). Formation of desired diphosphate compound was confirmed using negative-mode ESI-MS. The eluent was concentrated under reduced pressure and then lyophilized to obtain a crude solid product. Purification of that material was done using a cellulose column. The column was packed using 10% solvent A in THF (v/v). The same solvent mixture was used to load the lyophilized crude product onto the column by using a minimum amount of 10% solvent A in THF (~ 4 mL). Elution was performed using a gradient of 10% - 30% solvent A in THF (v/v). All fractions collected were checked using negative-mode ESI-MS to identify those containing the pure diphosphate product. Those fractions were pooled, concentrated under reduced pressure, then lyophilized to obtain **1a** as pale white/yellow solid (15.6 mg, 7.4% yield over two steps, quantity determined by ^{31}P NMR using an internal standard).

In some cases, the ^{1}H and ^{13}C NMR peak assignments for **1a** provided below were made using information obtained from the corresponding precursor alcohol **18**.

^{1}H NMR (500 MHz, CD_3OD): δ 8.86 (dd, *J* = 6.8, 1.2 Hz, 1H, H15), 7.65 (dd, *J* = 6.9, 3.9 Hz, 1H, H14), 7.47 (m, 1H, H13), 5.45 (m, 2H, H2 & H6), 4.61 (s, 2H, H8), 4.53 (t, *J* = 6.5 Hz, 2H, H1), 3.95 (s, 2H, H11), 2.20 (m, 2H, H5), 2.09 (m, 2H, H4), 1.71 (s, 3H, H10), 1.68 (s, 3H, H9).

^{1}H NMR (500 MHz, D_2O): δ 8.94 (d, *J* = 6.8 Hz, 1H), 7.67 (dd, *J* = 6.9, 3.8 Hz, 1H), 7.55 (d, *J* = 3.9 Hz, 1H), 5.39 (m, 2H), 4.61 (s, 2H), 4.38 (t, *J* = 6.7 Hz, 2H), 3.93 (s, 2H), 2.11 (m, 4H), 1.64 (s, 3H), 1.59 (s, 3H).

^{1}H NMR (500 MHz, DMSO-*d6*): δ 8.81 (dd, *J* = 6.9, 1.2 Hz, 1H), 7.66 (m, 1H), 7.50 (d, *J* = 3.9 Hz, 1H), 5.38 (t, *J* = 7.1 Hz, 1H), 5.30 (t, *J* = 6.6 Hz, 1H), 4.53 (s, 2H), 4.26 (s, 2H), 3.89 (s, 2H), 2.13 (m, 2H), 2.01 (m, 2H), 1.61 (s, 3H), 1.60 (s, 3H).

^{13}C NMR (126 MHz, D_2O): δ 147.21 (C12), 142.39 (C7), 137.47 (C14), 136.67 (C13), 131.76 (C3), 129.86 (C6), 127.81 (C15), 120.21 (d, $J_{C\text{-}P}$ = 8.3 Hz, C2), 75.70 (C11), 71.01 (C8), 62.47 (C1), 38.40 (C4), 25.44 (C5), 15.66 (C10), 13.49 (C9).

^{31}P NMR (162 MHz, D_2O): δ -6.34 (d, *J* = 22.3 Hz, 1P), -10.39 (d, *J* = 22.5 Hz, 1P).

HRMS (ESI) m/z:

Calcd for $C_{15}H_{23}O_8P_2{}^{130}\mathbf{Te}^-$ $[M+2H]^-$: 522.9936; found: 522.9935.

Calcd for $C_{15}H_{23}O_8P_2{}^{128}\mathbf{Te}^-$ $[M+2H]^-$: 520.9918; found: 520.9899.

Calcd for $C_{15}H_{23}O_8P_2{}^{126}\mathbf{Te}^-$ $[M+2H]^-$: 518.9906; found: 518.9902.

(2*E*,6*E*,10*E*)-3,7,11-trimethyl-12-(tellurophen-2-ylmethoxy)dodeca-2,6,10-trien-1-yl diphosphate (2a)

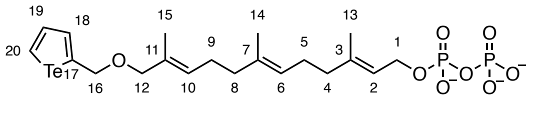

Compound **2a** was synthesized using alcohol **19** (348 mg, 0.809 mmol) in place of **18**, following the same two-step synthetic procedure as used for **1a**. The product was obtained as a pale white solid (29.3 mg, 6% yield over two steps, values determined by ^{31}P NMR with an internal standard).

In some cases, the ^{1}H and ^{13}C NMR peak assignments for **2a** provided below were made using information obtained from the corresponding precursor alcohol **19**.

^{1}H NMR (500 MHz, CD_3OD): δ 8.89 (dd, *J* = 6.9, 1.3 Hz, 1H, H20), 7.67 (m, 1H, H19), 7.49 (m, 1H, H18), 5.45 (m, 2H, H2 & H10), 5.17 (m, 1H, H6), 4.63 (s, 2H, H12), 4.55 (t, *J* = 6.3 Hz, 2H, H1), 3.97 (s, 2H, H16), 2.14 (m, 8H, H4 & H5 & H8 & H9), 1.70 (s, 3H, H14), 1.69 (s, 6H, H13 & H15).

^{1}H NMR (500 MHz, D_2O): δ 8.82 (m, 1H), 7.70 (s, 1H), 7.48 (s, 1H), 5.49 (m, 2H), 5.22 (m, 1H), 4.59 (s, 2H), 4.55 (d, J = 7.1 Hz, 2H), 3.95 (s, 2H), 2.18 (m, 8H), 1.79 (s, 3H), 1.69 (s, 3H), 1.67 (s, 3H).

^{13}C NMR (126 MHz, D_2O): δ 147.10 (C17), 142.65 (C19), 137.42 (C18), 136.63 (C7), 136. 21 (C11), 131.32 (C10), 130.24 (C20), 127.74 (C6), 124.71 (C2), 119.97 (C3), 75.64 (C16), 70.63 (C12), 62.38 (C1), 38.90 (C4 or C8), 38.33 (C4 or C8), 25.79 (C5 or C9), 25.32 (C5 or C9), 15.70 (C13 or C15), 15.15 (C13 or C15), 13.46 (C14).

^{31}P NMR (162 MHz, D_2O): δ -6.38 (d, *J* = 23.0 Hz, 1P), -10.44 (d, *J* = 22.2 Hz, 1P).

HRMS (ESI) m/z:

Calcd for $C_{20}H_{31}O_8P_2$**^{130}Te**$^{-}$ [M+2H]$^{-}$: 591.0562; found: 591.0561.

Calcd for $C_{20}H_{31}O_8P_2$**^{128}Te**$^{-}$ [M+2H]$^{-}$: 589.0544; found: 589.0538.

Calcd for $C_{20}H_{31}O_8P_2$**^{126}Te**$^{-}$ [M+2H]$^{-}$: 587.0532; found: 587.0524.

Synthesis of C10DHTeOPP (1b) and C15DHTeOPP (2b)

(6*E*)-3,7,11-trimethyldodeca-2,6,10-trienal (20)

(Previously reported compound)[1]

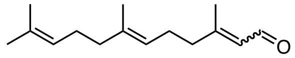

Pyridinium chlorochromate (11.6 g, 54.0 mmol, 2.00 equiv) was dissolved in anhydrous CH_2Cl_2 (60 mL) under N_2 (g). *Trans, trans*-farnesol **9** (6.00 g, 27.0 mmol, 1.00 equiv) was added slowly over 5 min to the reaction flask and the mixture was stirred at rt for 1 h. After that the solvent was removed under reduced pressure and the resulting precipitate was extracted with Et_2O (3 x 75 mL). The combined organic layers were dried over anhydrous $MgSO_4$, filtered, and concentrated under reduced pressure. Purification via silica gel flash column chromatography and elution with 20% EtOAc in hexanes (v/v) afforded **21** as colorless oil (4.20 g, 71% yield).

(*E*)-3,7,11-trimethyldodeca-6,10-dienal (21)

(Previously reported compound)[1]

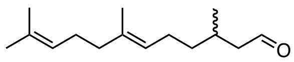

Compound **21** (4.20 g, 19.1 mmol 1.00 equiv) was dissolved in a mixture of HOAc (1.1 mL) and C_6H_6 (63 mL) under Ar (g). $Pd(PPh_3)_4$ (221 mg, 0.191 mmol, 0.01 equiv) was added to the solution and the flask was purged with Ar (g) for 5 min. The reducing agent $(n\text{-Bu})_3SnH$ (containing 0.05% BHT as stabilizer, Sigma-Aldrich, 6.2 mL, 22.8 mmol, 1.19 equiv) was added dropwise to the flask over 5 min. The reaction mixture was stirred at rt for 1 h before being poured into 1:1 (v/v) ice-water/CH_2Cl_2 mixture (68 mL). The resulting aqueous phase was removed, and the organic layer was further washed with brine (2 x 50 mL), dried over anhydrous $MgSO_4$, filtered, and concentrated under reduced pressure. The crude product was directly used in the next step.

(*E*)-3,7,11-trimethyldodeca-6,10-dien-1-ol / (±)-2,3-dihydrofarnesol (22)

(Previously reported compound)[1]

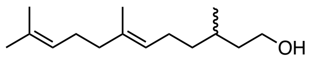

Compound **21** (19.1 mmol, assuming 100% conversion from the previous step, 1.00 equiv) was dissolved in anhydrous CH_3OH (62 mL) under N_2 (g) and cooled in ice-water bath. $NaBH_4$ (1.44 g, 38.0 mmol, 2.00 equiv) was divided into six portions and added sequentially, with each portion introduced only after gas evolution from the previous addition had subsided. The reaction mixture was stirred while maintained in an ice-water bath for one h before being quenched with H_2O (150 mL). The mixture was extracted with CH_2Cl_2 (3 x 70 mL). The combined organic layers were dried over anhydrous $MgSO_4$, filtered, and concentrated under reduced pressure. Purification via silica gel flash column chromatography and elution with 20% EtOAc in hexanes (v/v) afforded **22** as colorless oil (3.38 g, 80% yield over two steps).

(*E*)-2,6-dimethyl-8-((tetrahydro-2*H*-pyran-2-yl)oxy)oct-2-en-1-ol (26)

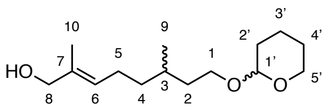

Compound **26** was synthesized using β-citronellol **23** (12.0 g, 76.8 mmol) in place of **8**, following the same two-step synthetic procedure outlined for **12**. Product **26** was obtained as a clear colorless to yellow oil (9.06 g, 46% yield over two steps).

Compound **26** exists as a mixture of four diastereomeric species, arising from two stereogenic centers (C3 and C1'). In the tabulated NMR data below, the presence of two resonances assigned to a single carbon reflects diastereomerism.

^{1}H NMR (500 MHz, $CDCl_3$): δ 5.37 (t, *J* = 7.6 Hz, 1H), 4.55 (td, *J* = 4.4, 2.7 Hz, 1H), 3.96 (s, 2H), 3.84 (m, 1H), 3.75 (m, 1H), 3.48 (m, 1H), 3.38 (m, 1H), 2.02 (m, 2H), 1.80 (m, 1H), 1.68 (m, 1H), 1.64 (s, 3H), 1.54 (m, 1H), 1.37 (m, 2H), 1.20 (m, 1H), 0.89 (d, *J* = 6.6 Hz, 3H).

^{13}**C NMR** (126 MHz, $CDCl_3$): δ 134.67, 126.55, 99.10 & 98.90 (C1'), 69.02 (C8), 66.01 & 65.93 (C5'), 62.49 & 62.42 (C1), 36.94 & 36.78 & 36.70 & 36.64 (C2 & C4), 30.87, 29.74 & 29.72 (C3), 25.58 & 25.12, 19.78 & 19.77 & 19.74 & 19.71 (C9 & C4'), 19.64, 13.72.

HRMS (ESI) m/z: Calcd for $C_{15}H_{28}O_3Na^+$ $[M+Na]^+$: 279.1931; found: 279.1917.

(2*E*,6*E*)-2,6,10-trimethyl-12-((tetrahydro-2*H*-pyran-2-yl)oxy)dodeca-2,6-dien-1-ol (27)

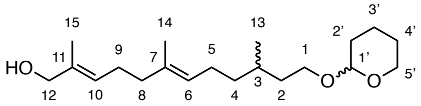

Compound **27** was synthesized using **22** (3.39 g, 15.1 mmol) in place of **8**, following the same two-step synthetic procedure as used for **12**. Intermediate **25** was purified and the spectra data were collected.

Spectral data for (*E*)-2-((3,7,11-trimethyldodeca-6,10-dien-1-yl)oxy)tetrahydro-2*H*-pyran (25)

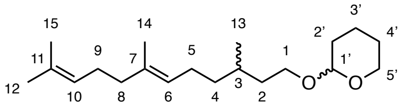

Compound **25** exists as a mixture of four diastereomeric species, arising from two stereogenic centers (C3 and C1'). In the tabulated NMR data below, the presence of two resonances assigned to a single carbon reflects diastereomerism.

^{1}**H NMR** (500 MHz, $CDCl_3$): δ 5.12 (m, 2H), 4.60 (dt, J = 5.0, 2.5 Hz, 1H), 3.90 (m, 1H), 3.81 (m, 1H), 3.53 (m, 1H), 3.43 (m, 1H), 2.04 (m, 6H), 1.84 (m, 1H), 1.75 (m, 1H), 1.70 (s, 3H), 1.66 (m, 1H), 1.62 (s, 6H), 1.55 (m, 3H), 1.41 (m, 2H), 1.24 (m, 3H), 0.93 (d, J = 6.6 Hz, 3H).

^{13}**C NMR** (126 MHz, $CDCl_3$): δ 134.90, 131.43, 124.87, 124.55, 99.11 & 98.95 (C1'), 66.18 & 66.07 (C5'), 62.48, 39.90, 37.40 & 37.26 & 36.86 & 36.79 (C2 & C4), 30.96, 29.83 & 29.79 (C3), 26.89, 25.85, 25.68, 25.50, 19.84, 19.74, 17.83, 16.11.

HRMS (ESI) m/z: Calcd for $C_{20}H_{37}O_2^+$ $[M+H]^+$: 309.2788; found: 309.2780.

The product **27** was obtained as a clear yellow oil (1.16 g, 24 % yield over two steps, mixture of C10=C11 *E/Z* isomers, ~2.8 : 1 ratio).

Compound **27** exists as a mixture of four diastereomeric species, arising from two stereogenic centers (C3 and C1'), as well as *E/Z* isomerization at the C10=C11 double bond. In the tabulated NMR data below, the presence of two resonances assigned to a single carbon reflects diastereomerism. Proton NMR signals uniquely attributable to the *Z* isomer are listed separately. The corresponding *Z*-isomer signals in the carbon NMR spectrum are also reported separately.

^{1}**H NMR** (500 MHz, $CDCl_3$) for *E*-C10=C11 isomer: δ 5.38 (t, J = 7.5 Hz, 1H), 5.12 (m, 1H), 4.56 (m, 1H), 3.98 (s, 2H), 3.86 (m, 1H), 3.77 (m, 1H), 3.50 (m, 1H), 3.40 (m, 1H), 2.05 (m, 6H), 1.82 (m, 1H), 1.69 (m,

1H), 1.66 (s, 3H), 1.63 (m, 1H), 1.59 (s, 3H), 1.53 (m, 5H), 1.40 (m, 2H), 1.18 (m, 2H), 0.90 (d, *J* = 6.6 Hz, 3H).

^{13}C NMR (126 MHz, $CDCl_3$) for *E*-C10=C11 isomer: δ 134.85, 134.48, 126.10, 125.19, 99.08 & 98.94 (C1'), 69.09, 66.11 & 66.04 (C5'), 62.57 & 62.47 (C1), 39.44, 37.32 & 37.16 & 36.82 & 36.73 (C2 & C4), 30.93 & 30.91, 29.74 & 29.68 (C3), 28.32, 26.27, 25.11 & 25.08, 19.86 & 19.80, 19.81 & 19.71 (C13), 16.06, 13.82.

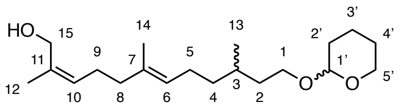

^{1}H NMR (500 MHz, $CDCl_3$) unique peaks for *Z*-C10=C11 isomer: δ 5.40 (t, *J* = 7.5 Hz, 1H), 4.02 (s, 2H), 1.68 (s, 3H), 0.92 (d, *J* = 6.6 Hz, 3H).

^{13}C NMR (126 MHz, $CDCl_3$) for *Z*-C10=C11 isomer: δ 138.78, 132.03, 127.65, 124.27, 99.17 & 98.96 (C1'), 67.44, 66.05 & 65.95 (C5'), 62.57 & 62.47 (C1), 39.44, 37.32 & 37.16 & 36.82 & 36.73 (C2 & C4), 30.93 & 30.91 (C2'), 29.89 & 29.86 (C3), 28.32, 27.27, 25.11 & 25.08, 19.86 & 19.80, 19.81 & 19.71 (C13), 17.79, 13.82.

HRMS (ESI) m/z: Calcd. for $C_{20}H_{36}O_3Na^+$ $[M+Na]^+$: 347.2557; found: 347.2551.

(*E*)-2-((8-chloro-3,7-dimethyloct-6-en-1-yl)oxy)tetrahydro-2*H*-pyran (28)

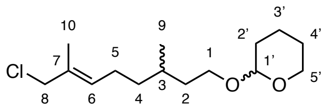

Compound **28** was synthesized using Riley oxidation product **26** (4.00 g, 15.6 mmol) in place of **12**, following the same synthetic procedure as for **14**. The product was obtained as a clear colorless oil (1.75 g, 41% yield).

Compound **28** exists as a mixture of four diastereomeric species, arising from two stereogenic centers (C3 and C1'). In the tabulated NMR data below, the presence of two resonances assigned to a single carbon reflects diastereomerism.

^{1}H NMR (500 MHz, $CDCl_3$): δ 5.52 (t, *J* = 7.5 Hz, 1H), 4.57 (dd, *J* = 4.6, 2.6 Hz, 1H), 4.01 (s, 2H), 3.87 (m, 1H), 3.79 (m, 1H), 3.50 (m, 1H), 3.41 (m, 1H), 2.05 (m, 2H), 1.83 (m, 1H), 1.73 (s, 3H), 1.60 (m, 6H), 1.41 (m, 2H), 1.22 (m, 2H), 0.91 (d, *J* = 6.6 Hz, 3H).

^{13}C NMR (126 MHz, $CDCl_3$): δ 131.61, 131.35, 99.17 & 98.98 (C1'), 66.02, & 65.92 (C5'), 62.55 & 62.51 (C1), 52.77, 36.76 & 36.70 & 36.65 & 36.50 (C2 & C4), 30.95, 29.82 & 29.79 (C3), 25.66, 19.86 & 19.84 & 19.75 & 19.64 (C9 & THP group), 14.22.

HRMS (ESI) m/z: Calcd for $C_{15}H_{27}ClO_2Na^+$ $[M+Na]^+$: 297.1592; found: 297.1581.

2-(((6*E*,10*E*)-12-chloro-3,7,11-trimethyldodeca-6,10-dien-1-yl)oxy)tetrahydro-2*H*-pyran (29)

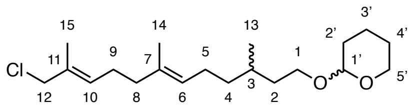

Compound **29** was synthesized using Riley oxidation product **27** (465 mg, 1.43 mmol) in place of **12**, following the same synthetic procedure as for **14**. The product was obtained as a clear colorless oil (349 mg, 71% yield, mixture of C10=C11 *E/Z* isomers, ~3.1 : 1 ratio).

Compound **29** exists as a mixture of four diastereomeric species, arising from two stereogenic centers (C3 and C1'), as well as *E/Z* isomerization at the C10=C11 double bond. In the tabulated NMR data below, the presence of two resonances assigned to a single carbon reflects diastereomerism. Proton NMR signals uniquely attributable to the *Z* isomer are listed separately. The corresponding *Z*-isomer signals in the carbon NMR spectrum are also reported separately.

^{1}H NMR (500 MHz, $CDCl_3$) for *E*-C10=C11 isomer: δ 5.50 (t, *J* = 7.2, 1H), 5.11 (t, *J* = 7.1 Hz, 1H), 4.57 (m, 1H), 4.01 (s, 2H), 3.87 (m, 1H), 3.78 (m, 1H), 3.51 (m, 1H), 3.41 (m, 1H), 2.05 (m, 6H), 1.82 (m, 2H), 1.73 (s, 3H), 1.64 (m, 2H), 1.59 (s, 3H), 1.54 (m, 3H), 1.38 (m, 2H), 1.21 (m, 2H), 0.90 (d, *J* = 6.7 Hz, 3H).

^{13}C NMR (126 MHz, $CDCl_3$) for *E*-C10=C11 isomer: δ 134.15, 132.29, 130.87, 125.46, 99.13 & 98.97 (C1'), 66.14 & 66.05 (C5'), 62.52 & 62.50 (C1), 52.74 (C12), 39.05, 37.35 & 37.21 & 36.84 & 36.77 (C2 & C4), 30.96, 29.84 & 29.80, 26.76, 25.67, 25.50, 19.85 & 19.82, 19.74 & 19.72, 16.06, 14.27.

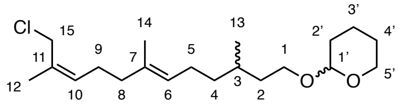

^{1}H NMR (500 MHz, $CDCl_3$) unique peaks for *Z*-C10=C11 isomer: δ 5.53 (t, *J* = 7.2, 1H), 4.05 (s, 2H), 1.69 (s, 3H), 0.91 (d, *J* = 6.7 Hz, 3H),

^{13}C NMR (126 MHz, $CDCl_3$) for *Z*-C10=C11 isomer: 135.55, 134.14, 131.74, 123.87, 99.13 & 98.97 (C1'), 66.14 & 66.05 (C5'), 62.52 & 62.50 (C1), 50.72 (C15), 39.05, 37.35 & 37.21 & 36.84, 36.77 (C2 & C4), 30.96, 29.92 & 29.88, 26.81, 25.86, 25.54, 19.85 & 19.82, 19.74 & 19.72, 17.83, 16.06.

HRMS (ESI) m/z: Calcd for $C_{20}H_{35}ClO_2Na^+$ $[M+Na]^+$: 365.2218; found: 365.2217.

(*E*)-2-((3,7-dimethyl-8-(tellurophen-2-ylmethoxy)oct-6-en-1-yl)oxy)tetrahydro-2*H*-pyran (30)

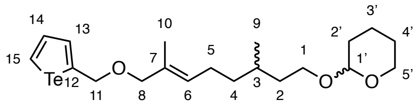

Compound **30** was synthesized using chlorinated product **28** (1.75 g, 6.37 mmol) in place of **14**, following the same synthetic procedure as for **16**. The product was obtained as a clear yellow oil (1.27 g, 90% yield, mixture of C6=C7 *E/Z* isomers, ~2.5 : 1 ratio).

Compound **30** exists as a mixture of four diastereomeric species, arising from two stereogenic centers (C3 and C1'), as well as *E/Z* isomerization at the C6=C7 double bond. In the tabulated NMR data below, the presence of two resonances assigned to a single carbon reflects diastereomerism. Proton NMR signals

uniquely attributable to the *Z* isomer are listed separately. The corresponding *Z*-isomer signals in the carbon NMR spectrum are also reported separately.

^{1}H NMR (500 MHz, $CDCl_3$) for *E*-C6=C7 isomer: δ 8.82 (dd, *J* = 6.8, 1.2 Hz, 1H), 7.68 (dd, *J* = 6.9, 3.9 Hz, 1H), 7.46 (m, 1H), 5.40 (dt, *J* = 7.2, 1.4 Hz, 1H), 4.62 (d, *J* = 1.4 Hz, 2H), 4.57 (dt, *J* = 5.1, 2.8 Hz, 1H), 3.95 (s, 2H), 3.87 (m, 1H), 3.78 (m, 1H), 3.50 (m, 1H), 3.40 (m, 1H), 2.06 (m, 2H), 1.82 (m, 2H), 1.71 (m, 2H), 1.67 (s, 3H), 1.64 (m, 2H), 1.55 (m, 2H), 1.41 (m, 2H), 1.23 (m, 1H), 0.91 (d, *J* = 6.5 Hz, 3H).

^{13}C NMR (126 MHz, CDCl3) for *E*-C6=C7 isomer: δ 149.64 (C12), 137.16 (C14), 133.60 (C13), 131.80 (C7), 129.14 (C6), 125.47 (C15), 99.13 & 98.97 (C1'), 76.43 (C11), 71.76 (C8), 66.09 & 65.99 (C5'), 62.50 & 62.48 (C1), 37.03 & 36.89 (C4), 36.80 & 36.74 (C2), 30.95 (C2'), 29.85 & 29.83 (C3). 25.66 (C3'), 25.30 & 25.26 (C5), 19.86 & 19.84 (C4'), 19.78 & 19.68 (C9), 14.14 (C10).

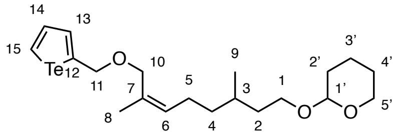

^{1}H NMR (500 MHz, $CDCl_3$) unique peaks for *Z*-C6=C7 isomer: 5.38 (dt, *J* = 7.2, 1.4 Hz), 3.83 (s, 2H), 1.64 (s, 3H), 0.92 (d, *J* = 6.5 Hz, 3H).

^{13}C NMR (126 MHz, CDCl3) for *Z*-C6=C7 isomer: δ 149.64 (C12), 137.16 (C14), 133.60 (C13), 132.31 (C7), 128.29 (C6), 125.47 (C15), 99.13 & 98.97 (C1'), 76.43 (C11), 71.76 (C10), 66.09 & 65.99 (C5'), 62.50 & 62.48 (C1), 37.03 & 36.89 (C4), 36.80 & 36.74 (C2), 30.95 (C2'), 29.85 & 29.83 (C3). 25.66 (C3'), 25.30 & 25.26 (C5), 19.86 & 19.84 (C4'), 19.78 & 19.68 (C9), 15.38 (C8).

HRMS (ESI) m/z: Calcd for $C_{20}H_{32}O_2NaTe^+$ $[M+Na]^+$: 473.1306; found: 473.1306.

2-(((6*E*,10*E*)-3,7,11-trimethyl-12-(tellurophen-2-ylmethoxy)dodeca-6,10-dien-1-yl)oxy)tetrahydro-2*H*-pyran (31)

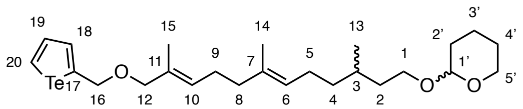

Compound **31** was synthesized using chlorinated product **29** (321 mg, 1.53 mmol) in place of **14**, following the same synthetic procedure as for **16**, with the exception that an excess of tellurophene **7** was used at a 1.5:1 molar ratio relative to compound **29**. The product was obtained as a clear yellow oil (370 mg, 46% yield, mixture of C10=C11 *E/Z* isomers, ~3.6 : 1 ratio).

Compound **31** exists as a mixture of four diastereomeric species, arising from two stereogenic centers (C3 and C1'), as well as *E/Z* isomerization at the C10=C11 double bond. In the tabulated NMR data below, the presence of two resonances assigned to a single carbon reflects diastereomerism. Proton NMR signals uniquely attributable to the *Z* isomer are listed separately. The corresponding *Z*-isomer signals in the carbon NMR spectrum are also reported separately.

^{1}H NMR (500 MHz, $CDCl_3$) for *E*-C10=C11 isomer: δ 8.82 (dd, *J* = 6.9, 1.2 Hz, 1H), 7.68 (dd, *J* = 6.8, 3.9 Hz, 1H), 7.46 (m, 1H), 5.40 (t, *J* = 7.2 Hz, 1H), 5.12 (t, *J* = 7.0 Hz, 1H), 4.61 (s, 2H), 4.57 (dt, *J* = 4.9, 2.5,

2.5 Hz, 1H), 3.96 (s, 2H), 3.87 (m, 1H), 3.78 (m, 1H), 3.50 (m, 1H), 3.41 (m, 1H), 2.05 (m, 6H), 1.83 (m, 1H), 1.72 (m, 3H), 1.68 (s, 3H), 1.64 (m, 2H), 1.61 (s, 3H), 1.54 (m, 2H), 1.39 (m, 2H), 1.19 (m, 1H), 0.90 (d, J = 6.6 Hz, 3H).

^{13}C NMR (126 MHz, $CDCl_3$) for *E*-C10=C11 isomer: δ 149.66 (C17), 137.15 (C19), 134.51 & 134.49 (C11), 133.56 (C18), 131.91 (C7), 128.72 (C10), 125.48 (C20), 125.20 (C6), 99.11 & 98.95 (C1'), 76.40 (C16), 71.66 (C12), 66.15 & 66.04 (C5'), 62.49 & 62.47 (C1), 39.45 (C8), 37.39 & 37.26 & 36.84 & 36.76 (C2 & C4), 30.96 (C2'), 29.87 & 29.82 (C3), 26.45 (C5), 25.67 (C3'), 25.53 (C9), 19.83 & 19.73 (C13 & C4'), 16.08 (C15), 14.17 (C14).

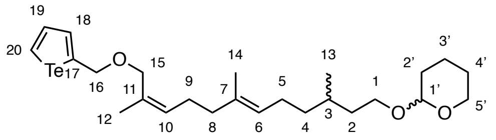

^{1}H NMR (500 MHz, $CDCl_3$) unique peaks for *Z*-C10=C11 isomer: δ 5.42 (t, J = 7.2 Hz, 1H), 4.63 (s, 2H), 3.99 (s, 2H), 0.93 (d, J = 6.6 Hz, 3H),

^{13}C NMR (126 MHz, $CDCl_3$) for *Z*-C10=C11 isomer: δ 149.62 (C17), 137.15 (C19), 135.68 (C11), 133.56 (C18), 131.83 (C7), 129.91 (C10), 125.48 (C20), 124.39 (C6), 99.14 & 98.96 (C1'), 74.69 (C16), 71.92 (C15), 66.09 & 65.97 (C5'), 62.49 & 62.47 (C1), 39.45 (C8), 37.35 & 37.22 & 36.84 & 36.76 (C2 & C4), 30.96 (C2'), 29.97 & 29.94 (C3), 26.45 (C5), 25.67 (C3'), 25.53 (C9), 19.83 & 19.73 (C13 & C4'), 17.89 (C12), 14.17 (C14).

HRMS (ESI) m/z: Calcd for $C_{26}H_{43}O_4Te^+$ $[M+CH_2OH]^+$: 549.2218; found: 549.2209.

(*E*)-3,7-dimethyl-8-(tellurophen-2-ylmethoxy)oct-6-en-1-ol (32)

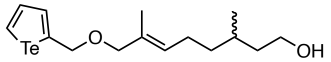

Compound **32** was synthesized using ether-linked product **30** (763 mg, 1.70 mmol) in place of **16**, following the same synthetic procedure as for **18**. The product was obtained as a clear pale-yellow oil (361 mg, 58% yield, mixture of C6=C7 *E/Z* isomers, ~3.2 : 1 ratio).

Compound **32** exists as a mixture of *E/Z* isomers due to isomerization at the C6=C7 double bond. In the tabulated NMR data below, duplicate resonances in the carbon NMR spectrum are observed and correspond to signals from the *E* and *Z* isomers. Proton NMR signals uniquely attributable to the *Z* isomer are listed separately.

^{1}H NMR (500 MHz, $CDCl_3$): δ 8.83 (dd, J = 6.8, 1.2 Hz, 1H), 7.68 (dd, J = 6.8, 3.9 Hz, 1H), 7.46 (m, 1H), 5.40 (m, 1H), 4.63 (s, 2H), 3.96 (s, 2H), 3.75 (s, 2H), 3.68 (m, 2H), 2.05 (m, 2H), 1.68 (d, J = 1.3 Hz, 1H), 1.60 (m, 3H), 1.39 (m, 1H), 1.25 (m, 2H), 1.21 (s, 3H), 0.91 (m, 3H).

^{13}C NMR (126 MHz, $CDCl_3$): δ 149.59, 137.18, 133.63, 133.27, 131.93, 128.95, 128.15, 127.26, 125.50, 76.40, 73.01, 71.83, 68.26, 65.16, 61.29, 40.00, 36.92 & 36.88, 29.35, 27.76, 25.36 & 25.30, 19.73 & 19.65, 14.22 & 14.03.

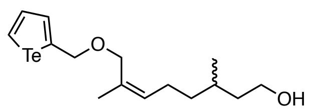

^{1}H NMR (500 MHz, $CDCl_3$) unique peaks for *Z*-C6=C7 isomer: 3.83 (s, 2H)

HRMS (ESI) m/z: Calcd for $C_{15}H_{24}O_2NaTe^+$ $[M+Na]^+$: 389.0731; found: 389.0727.

(6*E*,10*E*)-3,7,11-trimethyl-12-(tellurophen-2-ylmethoxy)dodeca-6,10-dien-1-ol (33)

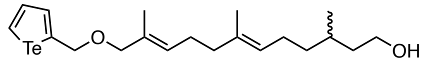

Compound **33** was synthesized using ether-linked product **31** (370 mg, 0.717 mmol) in place of **16**, following the same synthetic procedure as for **18**. The product was obtained as a clear pale-yellow oil (186 mg, 60% yield, mixture of C10=C11 *E/Z* isomers, ~2.4 : 1 ratio).

Compound **33** exists as a mixture of *E/Z* isomers due to isomerization at the C10=C11 double bond. In the tabulated NMR data below, proton NMR signals uniquely attributable to the *Z* isomer are listed separately. The corresponding *Z*-isomer signals in the carbon NMR spectrum are also reported separately.

^{1}H NMR (500 MHz, $CDCl_3$) for *E*-C10=C11 isomer: δ 8.82 (d, J = 6.8 Hz, 1H), 7.68 (dd, J = 6.8, 3.9 Hz, 1H), 7.46 (m, 1H), 5.40 (t, J = 7.2 Hz, 1H), 5.12 (t, J = 7.2 Hz, 1H), 4.62 (s, 2H), 3.96 (s, 2H), 3.67 (m, 2H), 2.05 (m, 6H), 1.67 (s, 3H), 1.60 (s, 3H), 1.59 (m, 2H), 1.37 (m, 2H), 1.20 (m, 1H), 0.90 (d, J = 6.6 Hz, 3H).

^{13}C NMR (126 MHz, $CDCl_3$) for *E*-C10=C11 isomer: δ 149.58, 137.13, 134.58, 133.61, 131.88, 128.64, 125.51, 125.07, 76.35, 71.71, 61.29, 40.01, 39.39, 37.29, 29.29, 28.48, 26.36,19.66, 16.06, 14.16.

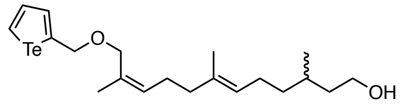

^{1}H NMR (500 MHz, $CDCl_3$) unique peaks for *Z*-C10=C11 isomer: 5.43 (t, J = 7.2 Hz, 1H),), 4.63 (s, 2H), 3.99 (s, 2H), 1.68 (s, 3H), 0.92 (d, J = 6.6 Hz, 3H).

^{13}C NMR (126 MHz, $CDCl_3$) for *Z*-C10=C11 isomer: δ 149.52, 137.13, 135.77, 133.61, 131.85, 129.70, 125.51, 124.33, 74.61, 71.95, 61.23, 39.98, 39.39, 37.24, 29.40, 27.13, 25.86, 19.63, 17.87, 14.16.

HRMS (ESI) m/z: Calcd for $C_{21}H_{35}O_3Te^+$ $[M+CH_2OH]^+$: 465.1643; found: 465.1649.

(*E*)-3,7-dimethyl-8-(tellurophen-2-ylmethoxy)oct-6-en-1-yl diphosphate (1b)

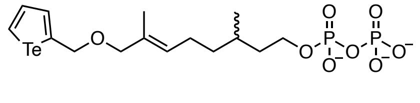

PPh_3 (393 mg, 1.50 mmol, 1.52 equiv) was dissolved in anhydrous CH_2Cl_2 (3 mL). A solution of THP deprotection product **32** (360 mg, 0.989 mmol, 1.00 equiv) in anhydrous CH_2Cl_2 (1 mL) was then added to

the reaction flask, followed by the addition of CBr_4 (397 mg, 1.20 mmol, 1.21 equiv) solubilized in anhydrous CH_2Cl_2 (1 mL). The reaction mixture was stirred for 90 min at rt before being concentrated under reduced pressure. The solid residue was dissolved in a minimal amount of CH_2Cl_2 and loaded onto a short silica gel column. Elution with 5% EtOA in hexanes (v/v) afforded a clear oil product. The resulting oil was redissolved in anhydrous CH_3CN (3 mL). Diphosphorylating reagent $[(n\text{-Bu})_4]_3P_2HO_7$ (1.16 g, 1.29 mmol, 1.30 equiv) was added to the reaction flask and the mixture was stirred for 24 h at rt. After that, the reaction mixture was concentrated under reduced pressure to afford a crude oil product.

The crude material was purified using the same purification procedure for compound **1a** to yield **1b** as a pale white to slight yellow solid (46.9 mg, 9% yield over two steps, values determined by ^{31}P NMR using internal standard).

^{1}H NMR (500 MHz, D_2O with water suppression): δ 8.98 (dd, *J* = 7.0, 1.5 Hz, 1H), 7.70 (dt, *J* = 6.8, 3.2 Hz, 1H), 7.59 (m, 1H), 5.49 (m, 1H), 3.91 (m, 2H), 3.80 (s, 2H), 2.04 (m, 1H), 1.62 (s, 3H), 1.40 (m, 1H), 1.19 (m, 3H), 0.87 (d, *J* = 8.4 Hz, 3H).

^{1}H NMR (500 MHz, DMSO-*d6*) δ 8.81 (dd, *J* = 6.7, 1.3 Hz, 1H), 7.67 (dd, *J* = 6.8, 3.9 Hz, 1H), 7.50 (m, 1H), 5.39 (t, *J* = 7.1 Hz, 1H), 4.54 (s, 2H), 3.89 (s, 2H), 3.72 (s, 2H), 2.01 (m, 2H), 1.60 (s, 3H), 1.32 (m, 2H), 1.13 (s, 3H), 0.85 (d, *J* = 6.6 Hz, 3H).

^{13}C NMR (126 MHz, D_2O): δ 147.22, 137.45, 136.66, 131.16, 127.77, 75.91, 71.02, 64.71, 36.90, 36.04, 28.77, 26.68, 24.67, 18.68, 13.45.

^{31}P NMR (162 MHz, D_2O): δ -6.35 (d, *J* = 21.9 Hz, 1P), -10.32 (d, *J* = 22.1 Hz, 1P).

HRMS (ESI) m/z:

Calcd. for $C_{15}H_{25}O_8P_2{}^{130}Te^-$ $[M+2H]^-$: 525.0092; found: 525.0092.

Calcd. for $C_{15}H_{25}O_8P_2{}^{128}Te^-$ $[M+2H]^-$: 523.0074; found: 523.0071.

Calcd. for $C_{15}H_{25}O_8P_2{}^{126}Te^-$ $[M+2H]^-$: 521.0063; found: 521.0065.

(6*E*,10*E*)-3,7,11-trimethyl-12-(tellurophen-2-ylmethoxy)dodeca-6,10-dien-1-yl diphosphate (2b)

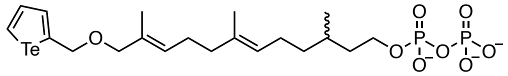

Compound **2b** was synthesized using THP-deprotection product **33** (186 mg, 0.430 mmol) in place of **32**, following the same two-step synthetic procedure used for **1b.** The product was obtained as a pale-yellow solid (70.7 mg, 28% yield over two steps, values determined by ^{31}P NMR using an internal standard; product exists as a mixture of C10=C11 *E/Z* isomers, ~3.7 : 1 ratio, determined by ^{1}H NMR).

^{1}H NMR (500 MHz, D_2O): δ 8.77 (m, 1H), 7.63 (dd, *J* = 7.1, 3.8 Hz, 1H), 7.42 (d, *J* = 4.5 Hz, 1H), 5.38 (t, *J* = 7.1 Hz, 1H), 5.17 (t, *J* = 7.6 Hz, 1H), 4.52 (s, 2H), 3.88 (s, 2H), 2.05 (m, 6H), 1.73 (m, 2H), 1.62 (s, 3H), 1.60 (s, 3H), 1.57 (m, 1H), 1.44 (m, 1H), 1.39 (m, 1H), 1.18 (m, 2H), 0.92 (m, 3H).

^{13}C NMR (126 MHz, D_2O): δ 165.25, 148.70, 137.08, 134.56, 131.62, 128.89, 126.78, 125.47, 124.38, 75.78, 71.86, 64.59, 39.18, 37.41, 29.37, 26.21, 25.32, 18.91, 15.82, 14.02.

^{31}P NMR (162 MHz, D_2O): δ -6.45 (d, *J* = 21.9 Hz, 1P), -10.42 (d, *J* = 21.7 Hz, 1P).

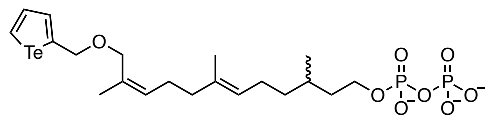

^{1}H NMR (500 MHz, D_2O) unique peaks for *Z*-C10=C11 isomer: δ 5.50 (t, *J* = 7.3 Hz, 1H), 4.61 (s, 2H), 3.95 (s, 2H), 1.66 (s, 3H).

HRMS (ESI) m/z:

Calcd for $C_{20}H_{33}O_8P_2{}^{130}Te^-$ $[M+2H]^-$: 593.0718; found: 593.0717.

Calcd for $C_{20}H_{33}O_8P_2{}^{128}Te^-$ $[M+2H]^-$: 591.0700; found: 591.0698.

Calcd for $C_{20}H_{33}O_8P_2{}^{126}Te^-$ $[M+2H]^-$: 589.0689; found: 589.0716.

Synthesis of BCN-TAMRA (35)

(Previously reported compound)[8]

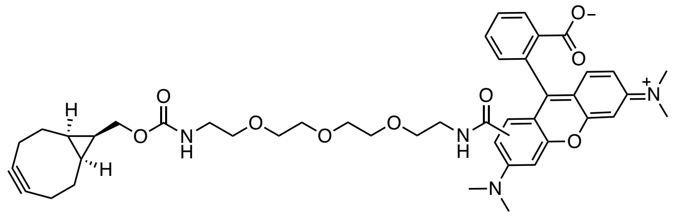

To a solution of *endo*-BCN-PEG3-amine **34** (3.32 mg, 0.009 mmol, 1.00 equiv) in DMSO (1.8 mL) in a capped scintillation vial, 5(6)-TAMRA NHS ester (10.2 mg, 0.0187 mmol, 2.08 equiv) and Et_3N (3.76 µL, 0.0270 mmol, 3.00 equiv) were added. The reaction mixture was stirred at rt for 4 h and the formation of the product **35** was monitored using ESI-MS. Purification was performed using semipreparative HPLC (1 mg sample per injection) with an Agilent ZORBAX 300SB-C18 column (5 µm particle size, 9.4 mm i.d × 250 mm) at a flow rate of 4 mL/min. Solvent A was H_2O with 0.1% TFA (v/v), and solvent B was CH_3CN 0.1% TFA (v/v). The program was as follows: 10 min hold at 1% B, linear gradient to 100% B over 50 min, 10 min hold at 100% B for washing column,1 min ramp down to 1% B, and a 15 min hold at 1% B for equilibration. Fractions were analyzed using ESI-MS to identify those containing the desired product. Those fractions were pooled, concentrated and lyophilized to yield **35** as a pink solid (~3 mg, 42% yield).

HRMS (ESI) m/z: Calcd for $C_{44}H_{53}N_4O_9^+$ $[M+H]^+$: 781.3807; found: 781.3817.

Synthesis of BCN-Biotin sulfone (37)

(Previously reported compound)[8]

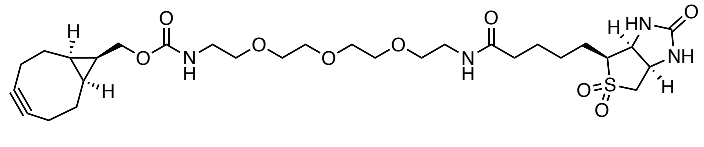

To a solution of biotin sulfone (50.0 mg, 0.181 mmol, 1.00 equiv) and *N*-hydroxysuccinimide (21.0 mg, 0.182 mmol, 1.00 equiv) in DMF (2 mL), *N,N′*-dicyclohexylcarbodiimide (DCC, 49.5 mg, 0.240 mmol, 1.32 equiv) was added portion wise. The reaction mixture was stirred at rt for 16 h. Half of the reaction mixture by volume was taken and transferred to a new vial. To that vial, a solution of *endo*-BCN-PEG3-amine **34** in DMSO (100 mM solution, 700 µL, 0.07 mmol) and 5 drops of Et_3N was added. The reaction mixture was stirred at rt for 5 h and the formation of the product **37** was monitored using ESI-MS. Purification was performed using semipreparative HPLC (1 mg sample per injection) with an Agilent ZORBAX 300SB-C18 column (5 µm particle size, 9.4 mm i.d × 250 mm) at a flow rate of 4 mL/min. Solvent A was H_2O with 0.1% TFA (v/v), and solvent B was CH_3CN 0.1% TFA (v/v). The program used was as follows: 10 min hold at 1% B, linear gradient to 100% B over 50 min, 10 min hold at 100% B for washing column,1 min ramp down to 1% B, and a 15 min hold at 1% B for equilibration. Fractions were analyzed using ESI-MS to identify those containing the desired product. Those fractions were pooled, concentrated and lyophilized to yield **35** as a pink solid (~20 mg, 18% yield).

HRMS (ESI) m/z: Calcd. for $C_{29}H_{46}N_4O_9SNa^+$ $[M+Na]^+$: 649.2878; found: 649.2887.

Spectral Characterization of Synthetic Compounds

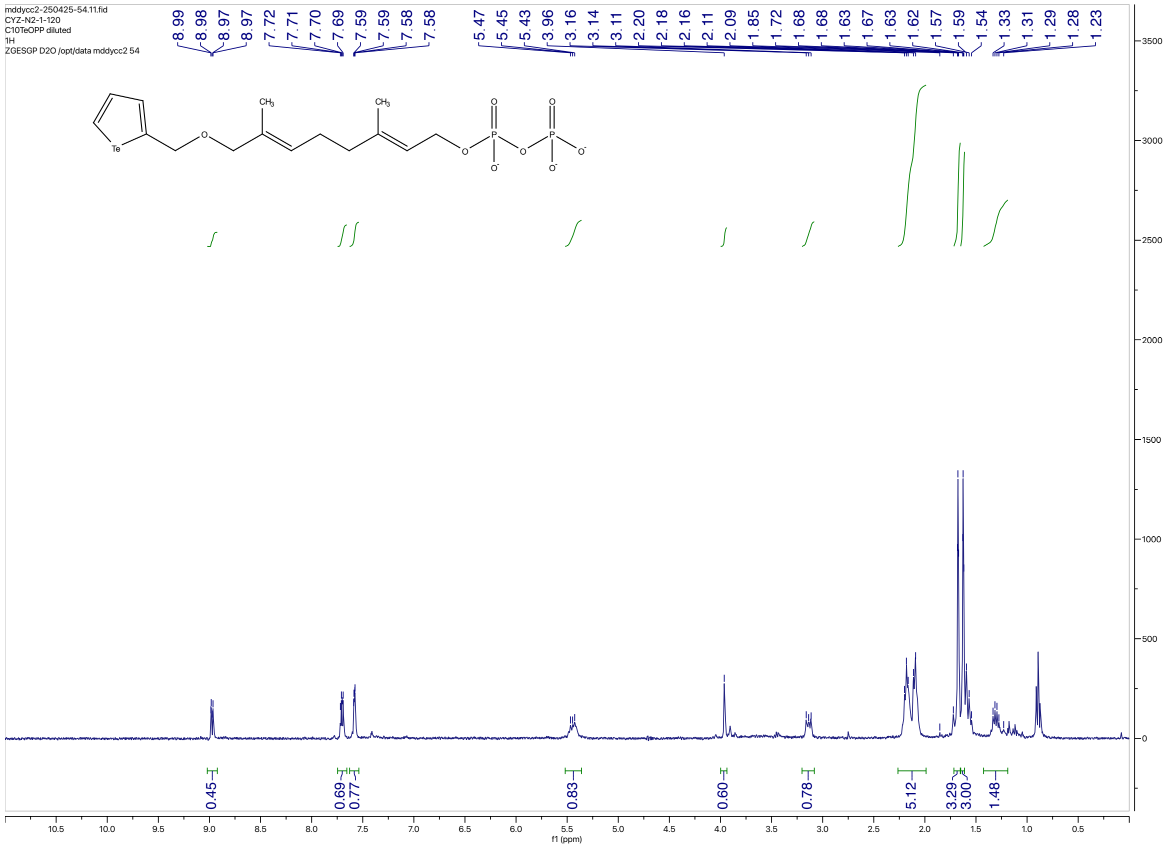

Compound **1a** ^{1}H NMR in D_2O with water suppression

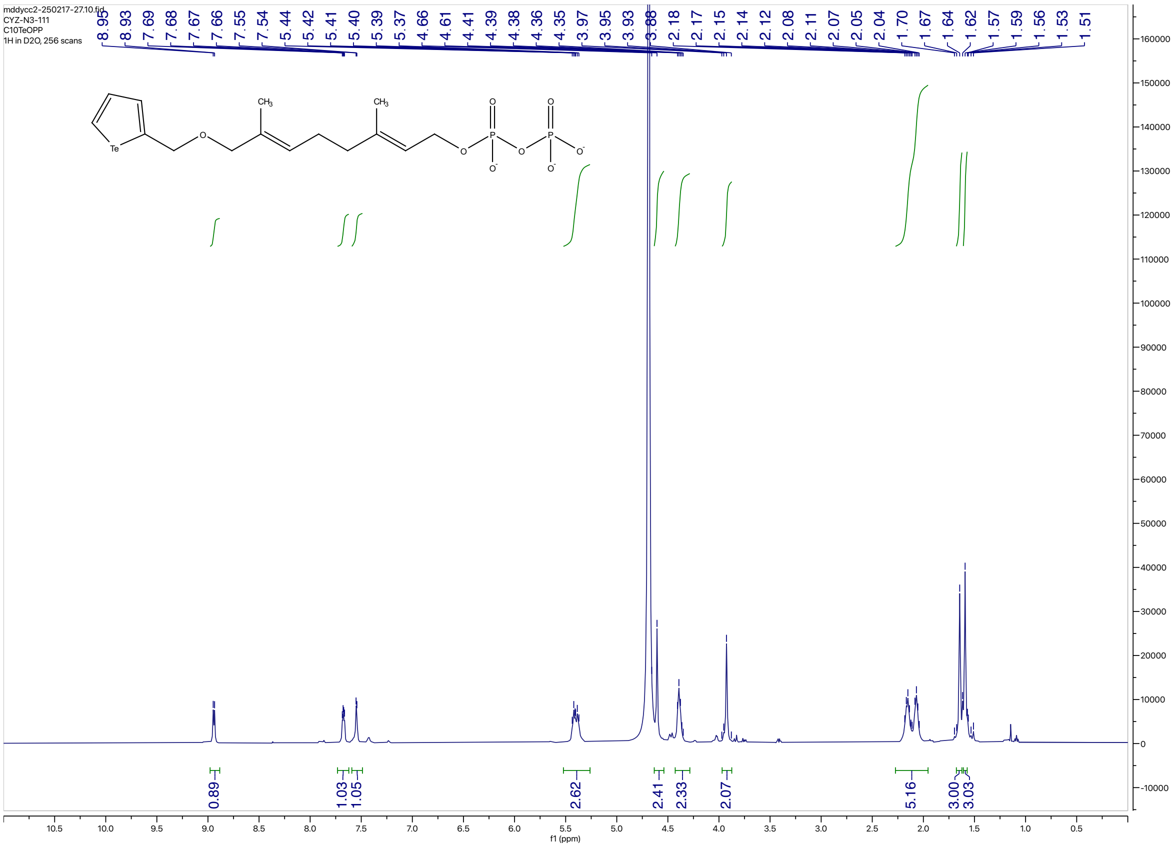

Compound **1a** ^{1}H NMR in D_2O

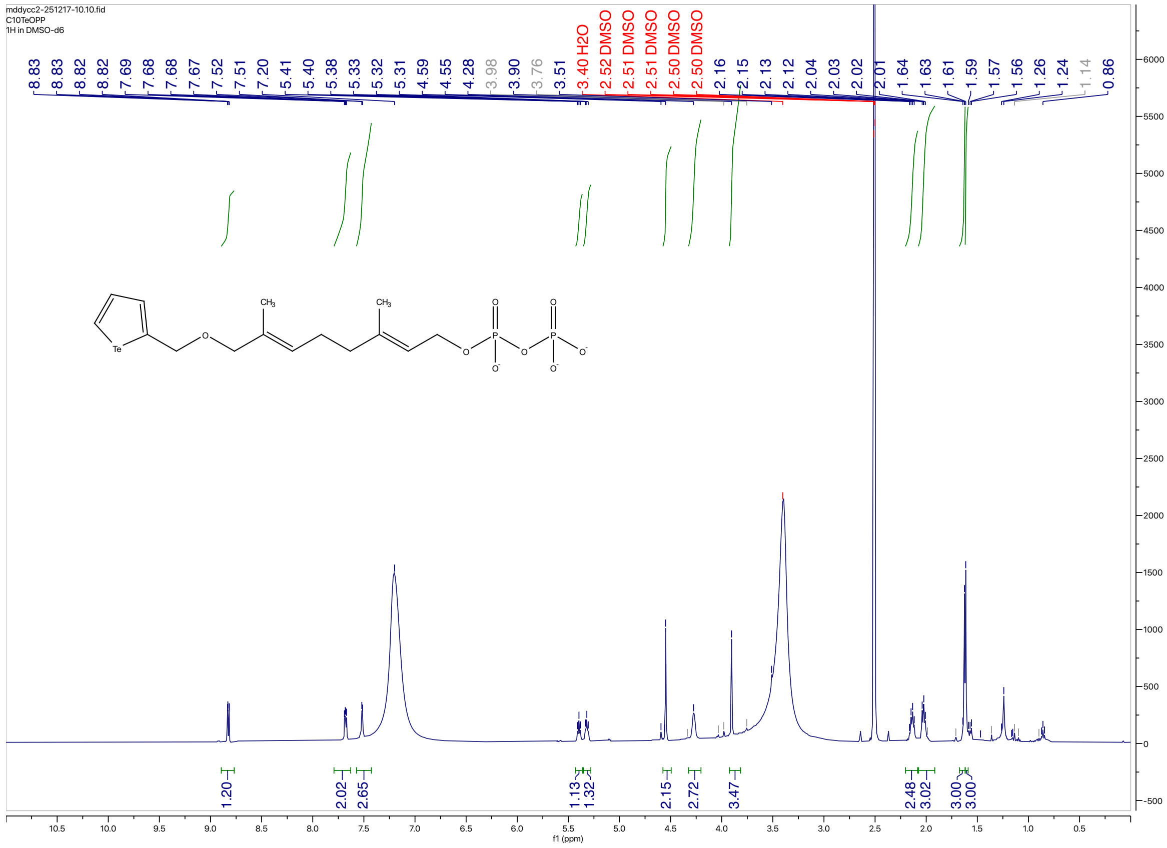

Compound **1a** ^{1}H NMR in DMSO-*d*6

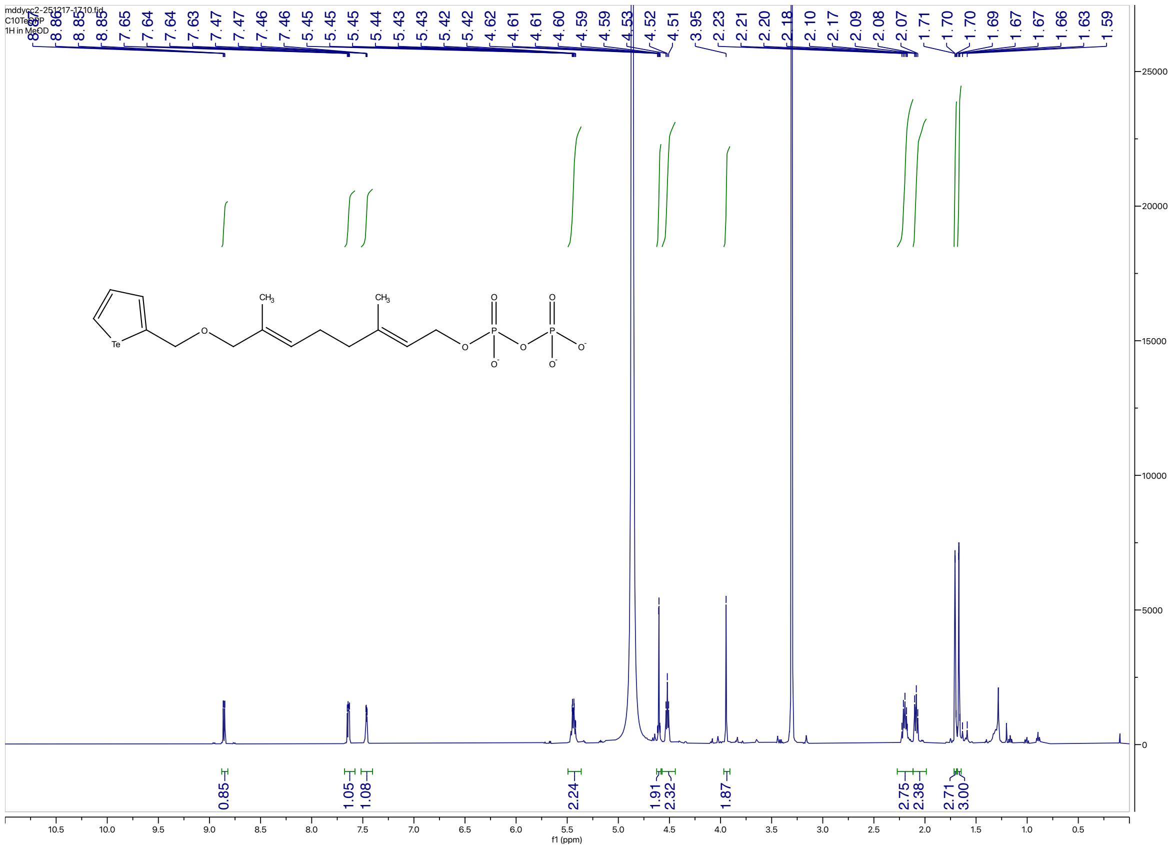

Compound **1a** ^{1}H NMR in CD_3OD

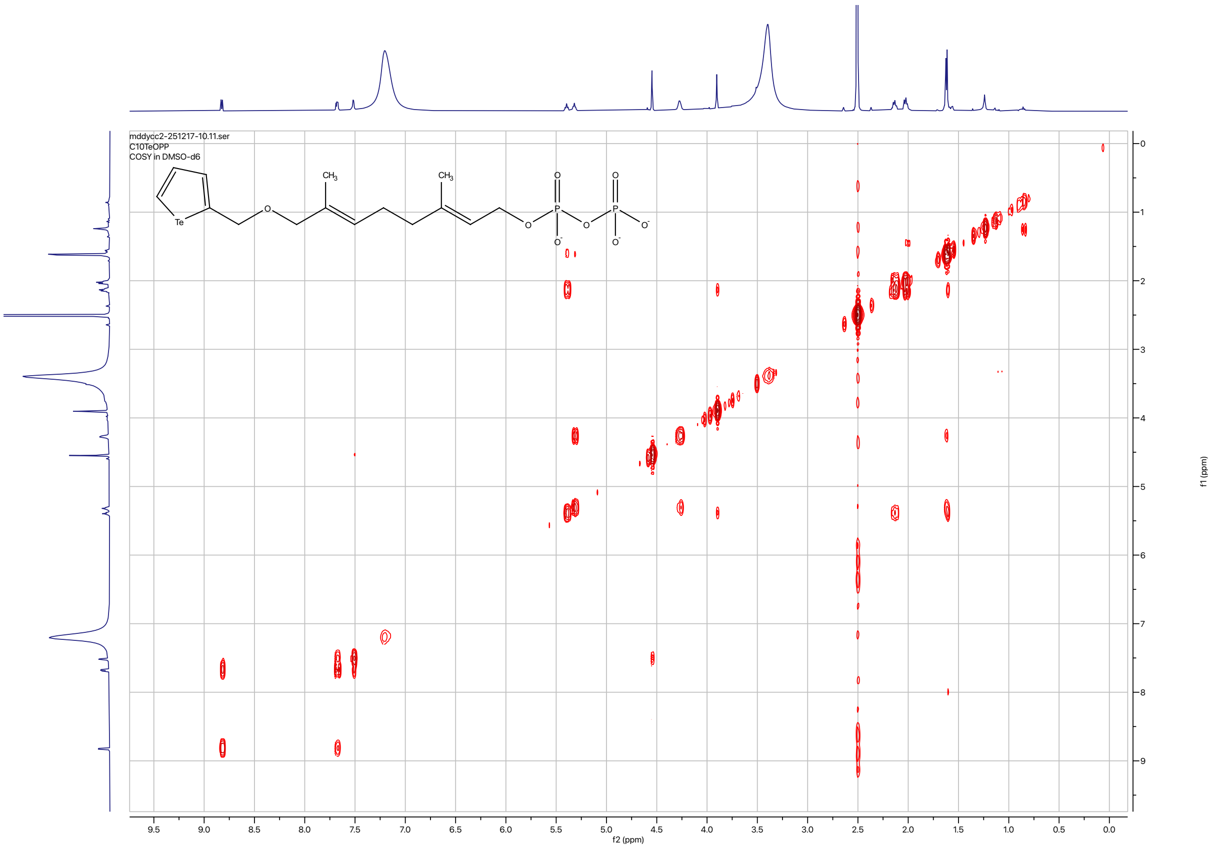

Compound **1a** ^{1}H-^{1}H COSY NMR in DMSO-*d6*

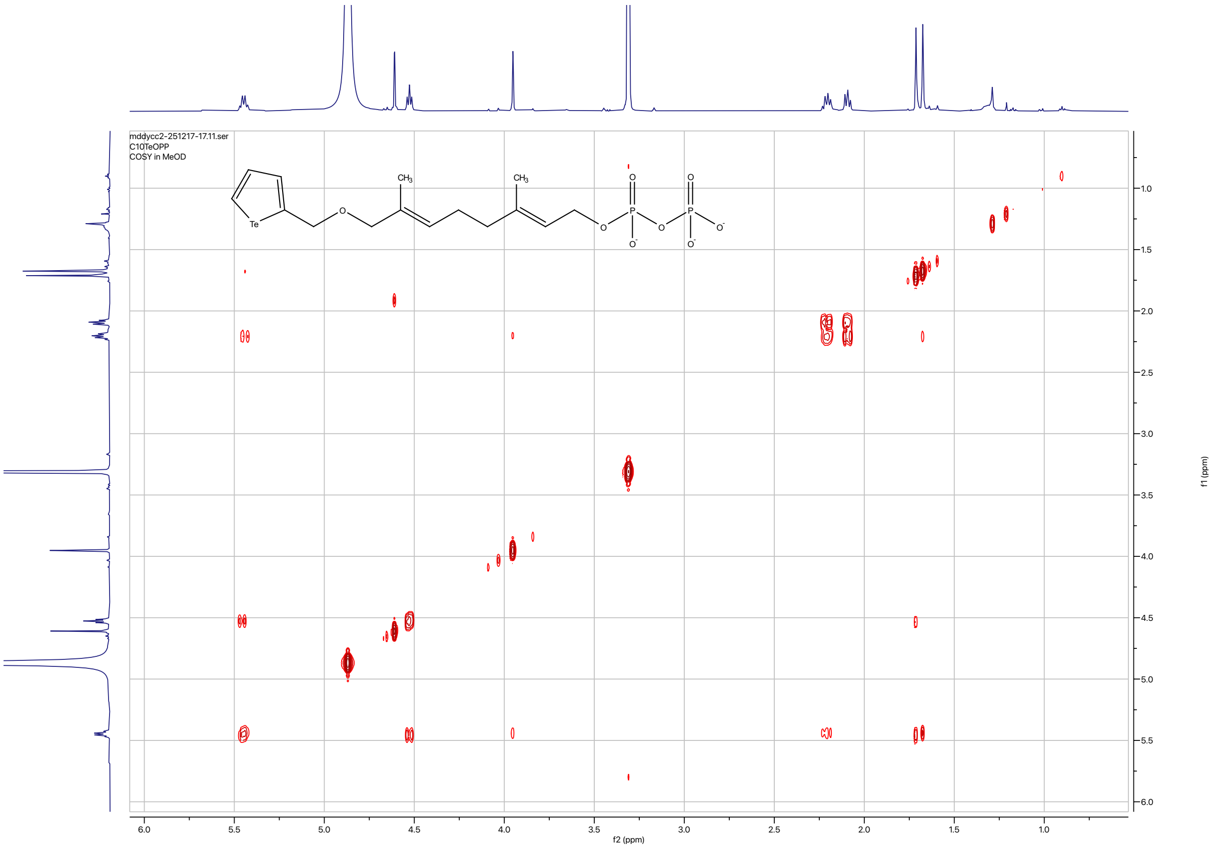

Compound **1a** ^{1}H-^{1}H COSY NMR in CD_3OD

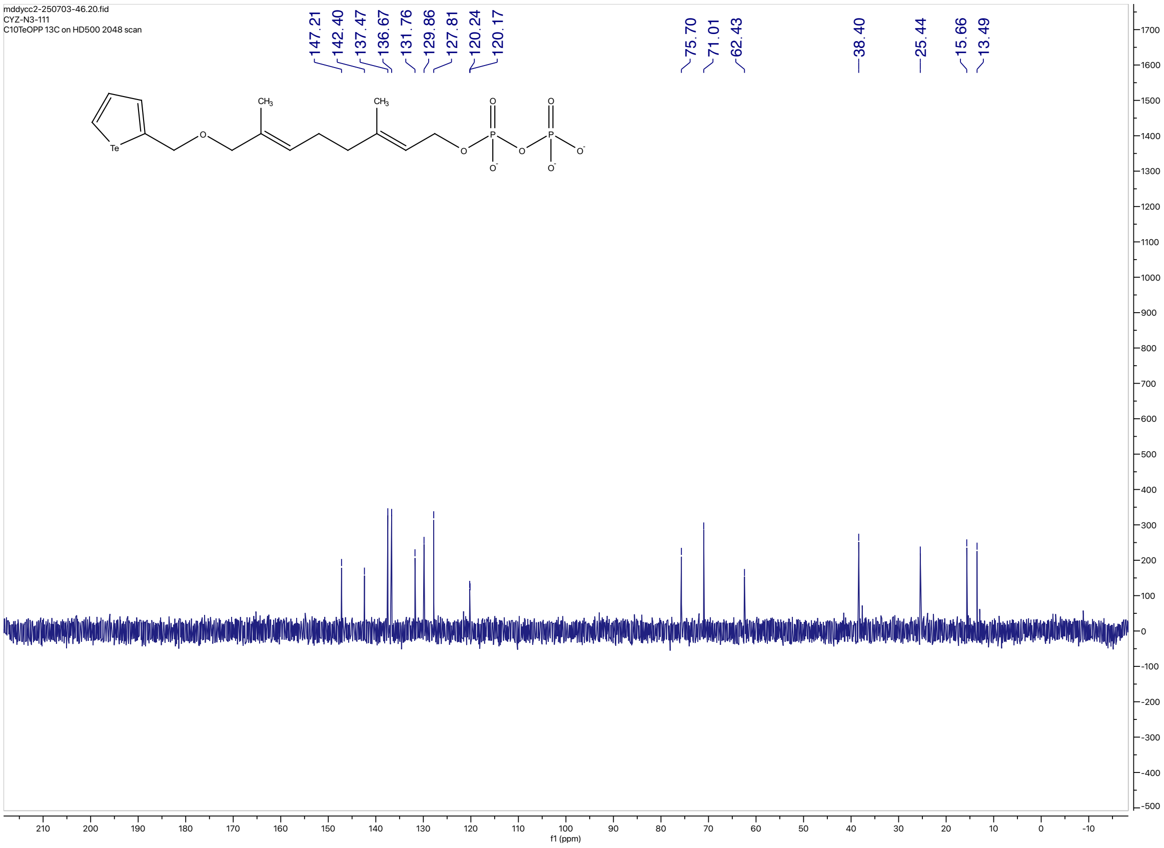

Compound **1a** ^{13}C NMR in D_2O

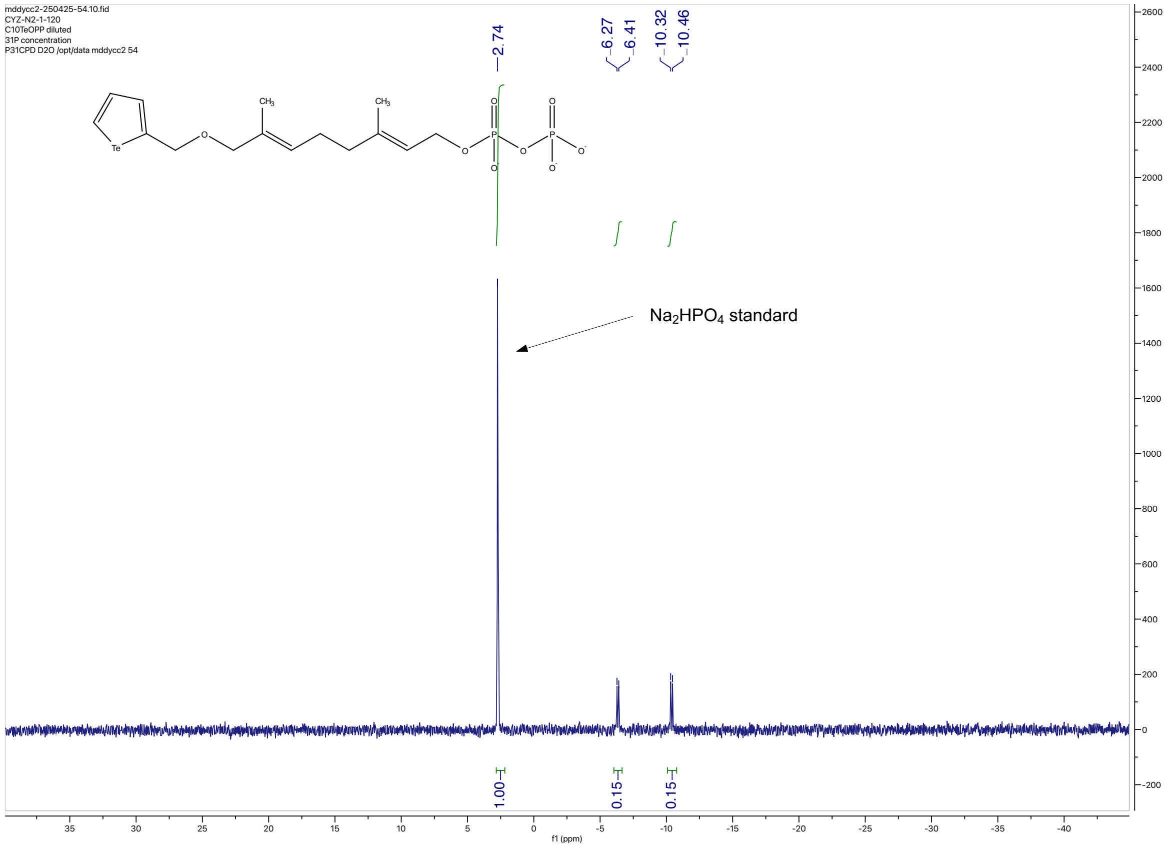

Compound **1a** ^{31}P NMR in D_2O with Na_2HPO_4 as internal standard

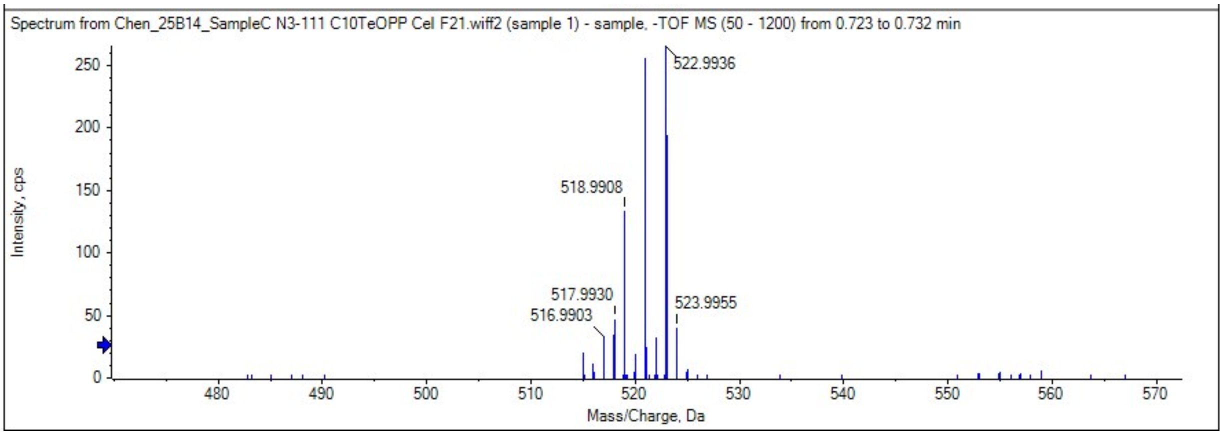

Compound **1a** HR-ESI-MS spectrum

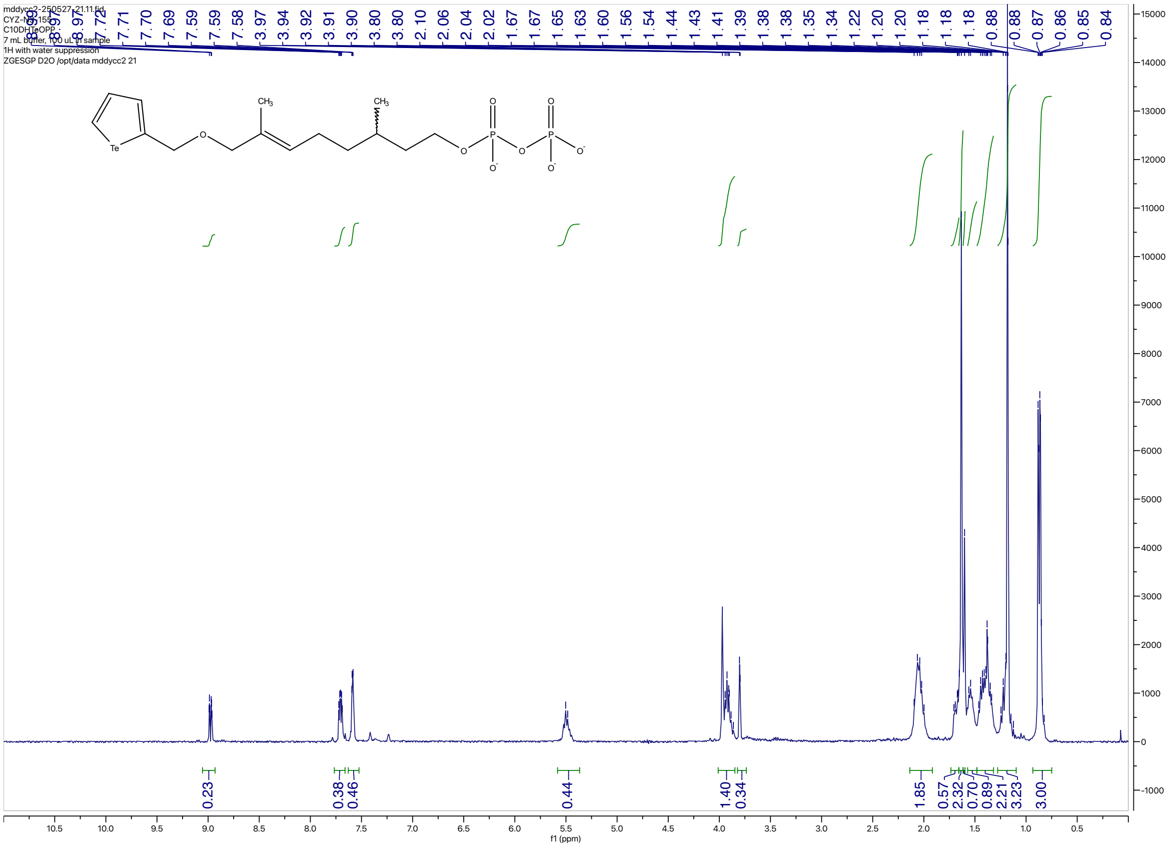

Compound **1b** ^{1}H NMR in D_2O with water suppression

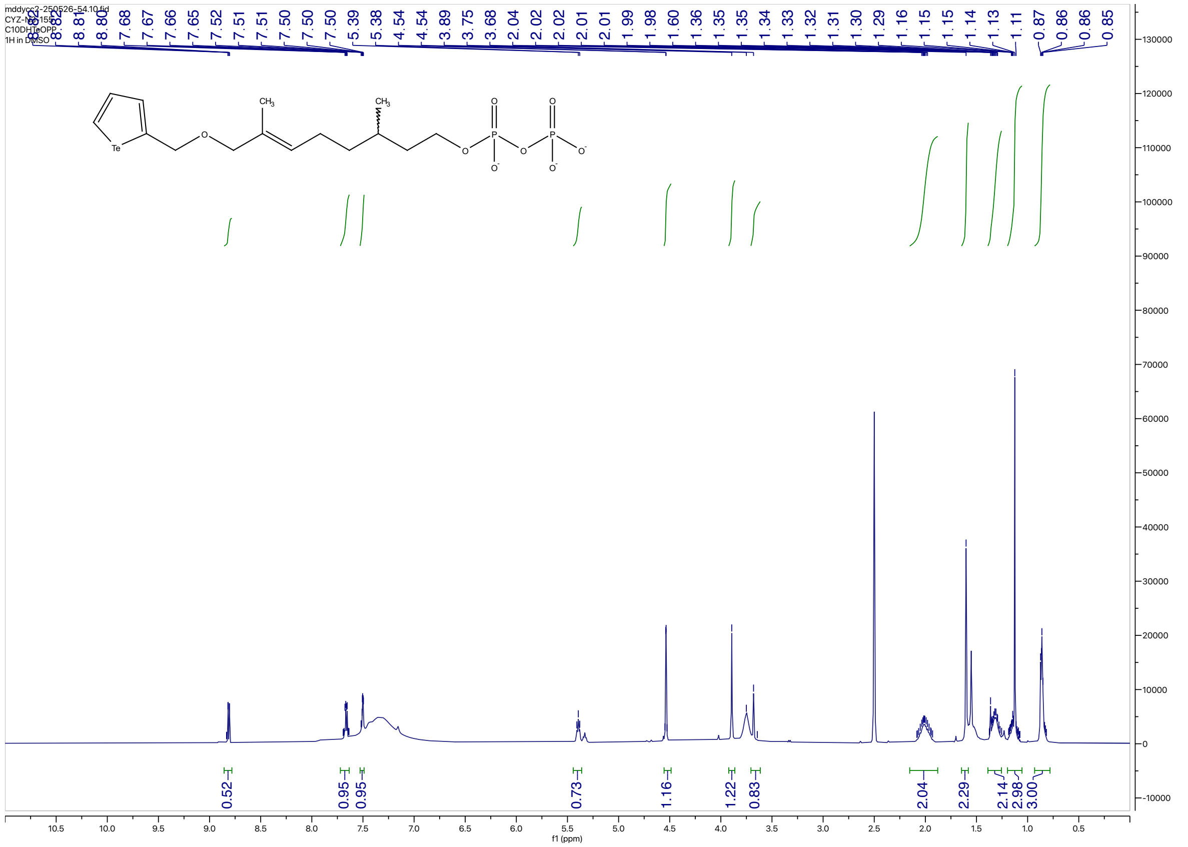

Compound **1b** ^{1}H NMR in DMSO-*d*6

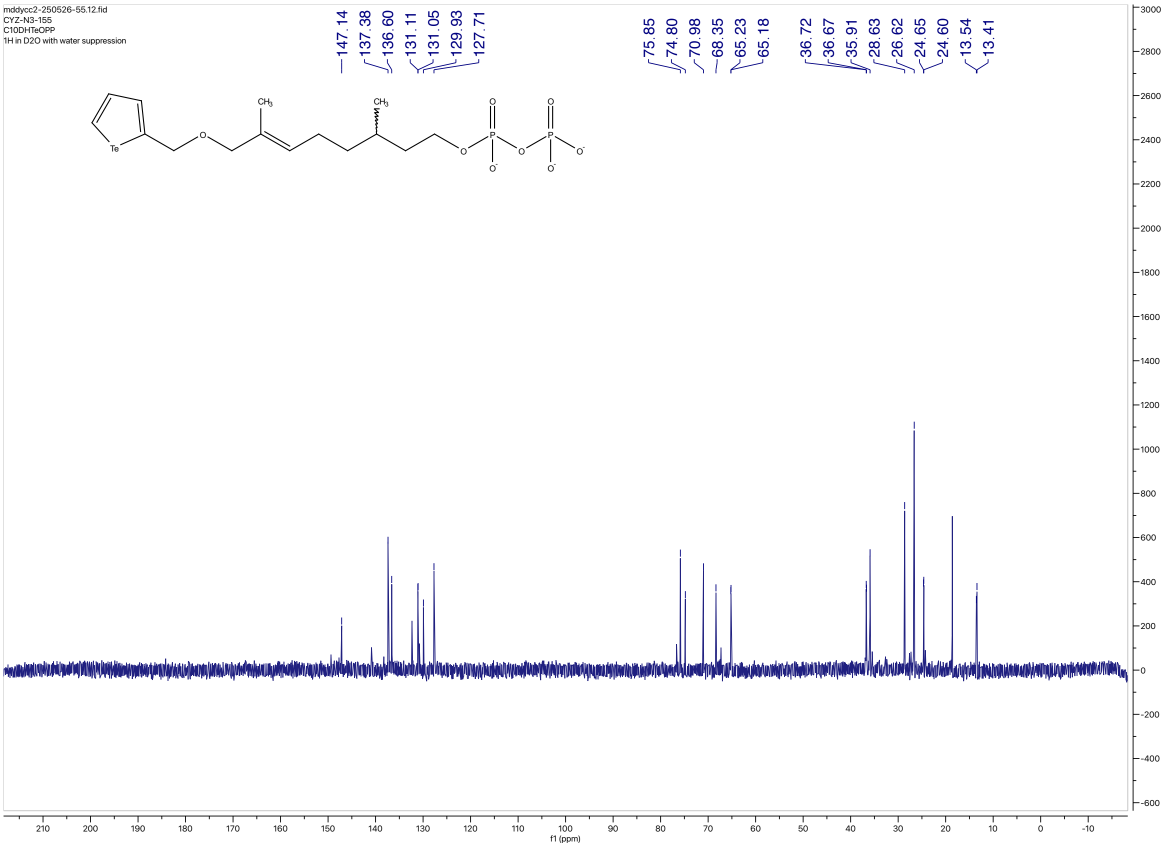

Compound **1b** ^{13}C NMR in D_2O

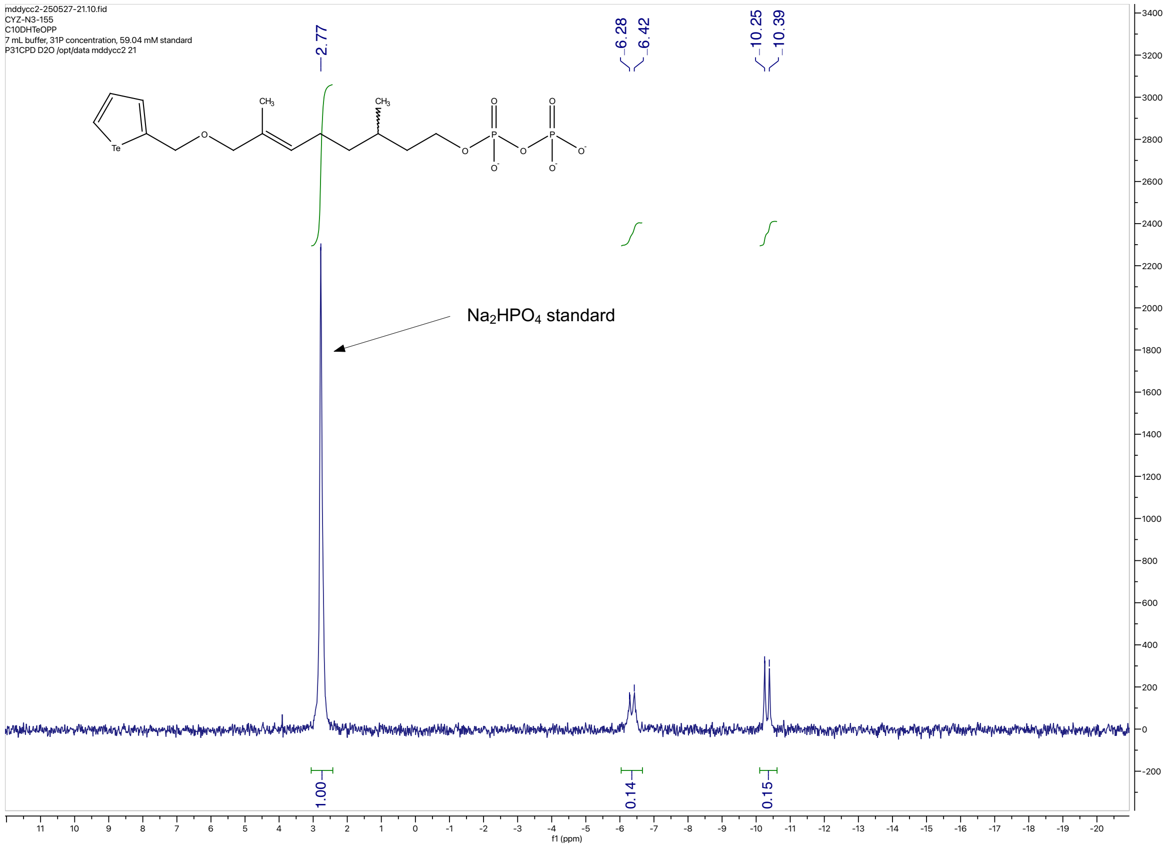

Compound **1b** ^{31}P NMR in D_2O with Na_2HPO_4 as internal standard

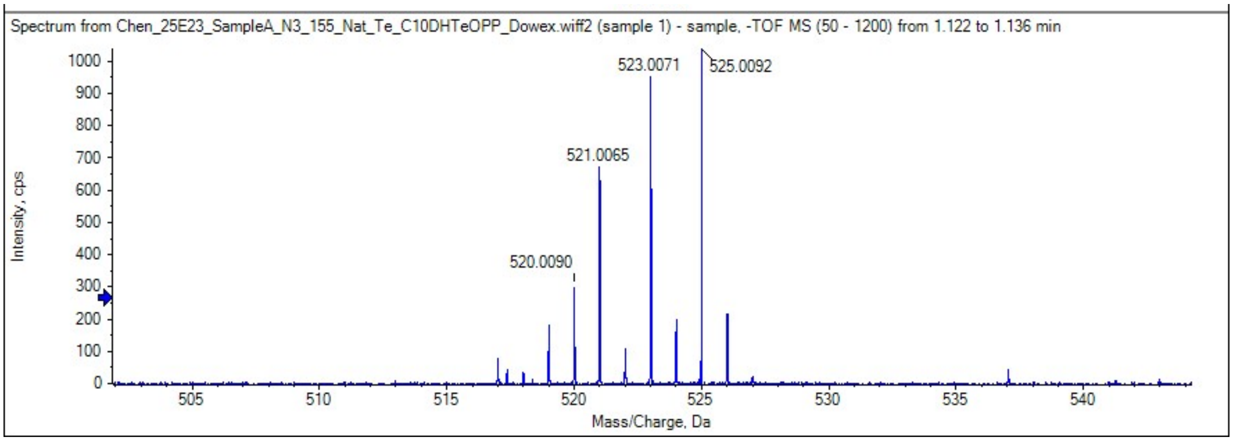

Compound **1b** HR-ESI-MS spectrum

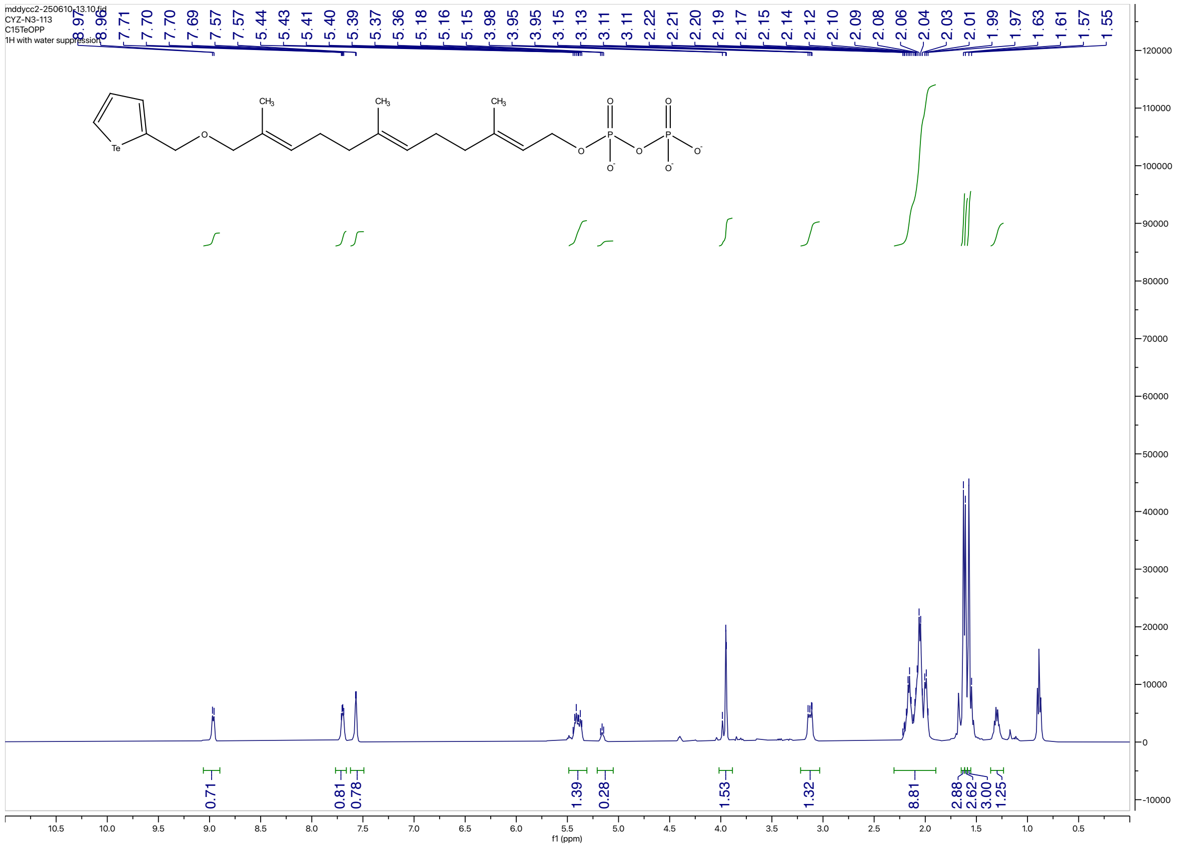

Compound **2a** ^{1}H NMR in D_2O with water suppression

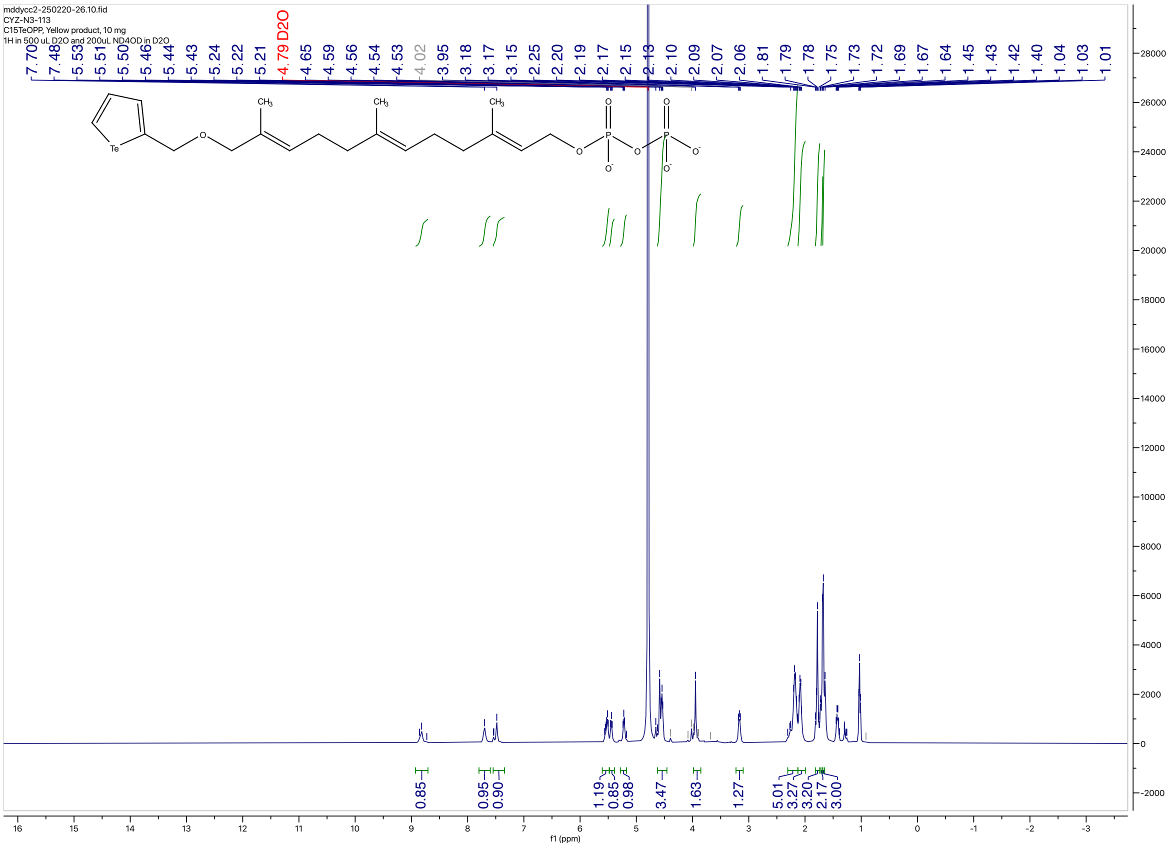

Compound **2a** ^{1}H NMR in D_2O

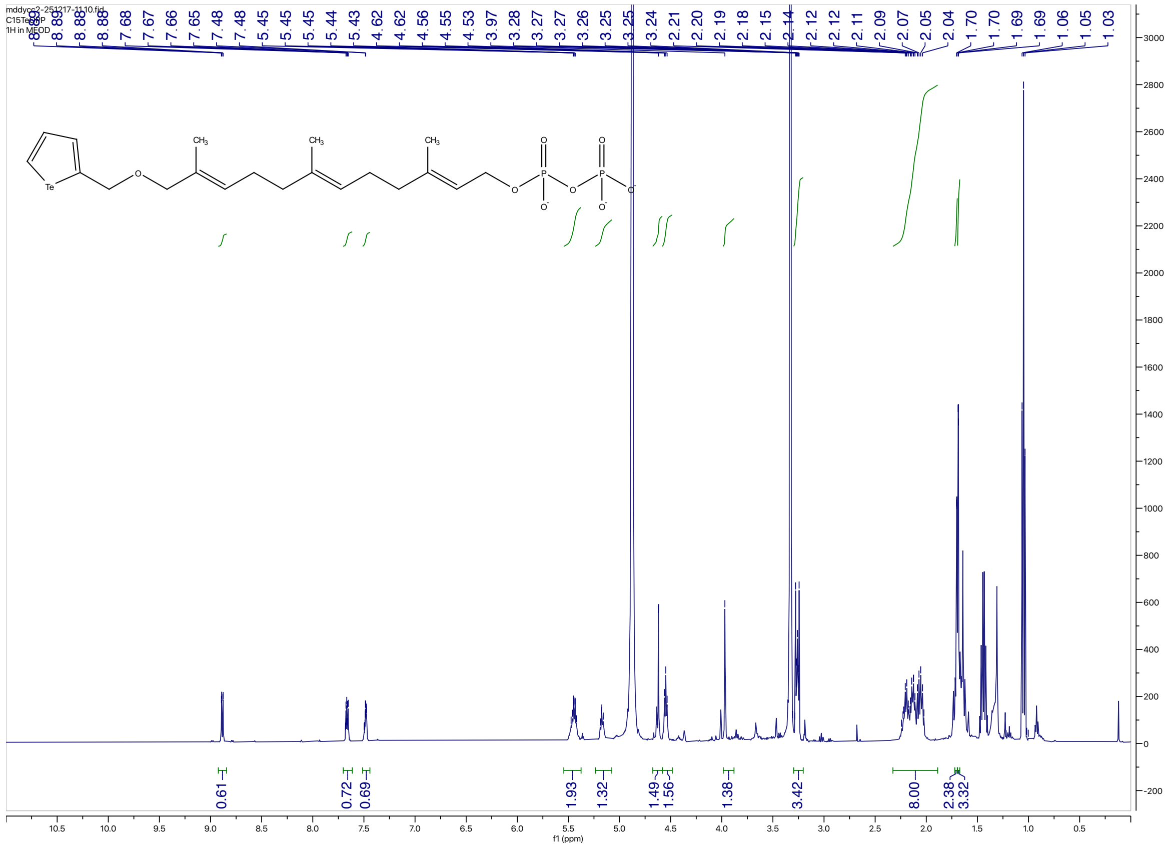

Compound **2a** ^{1}H NMR in CD_3OD

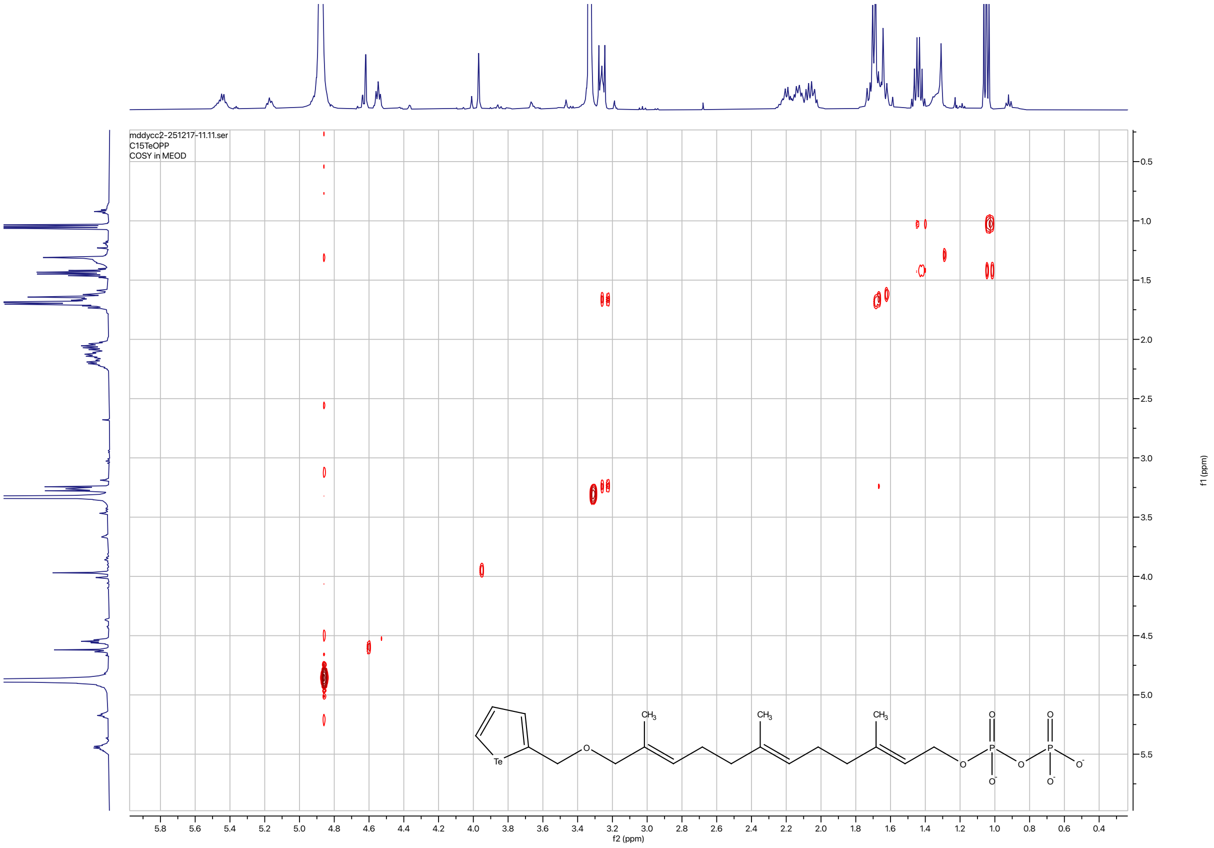

Compound **2a** ^{1}H-^{1}H COSY NMR in CD_3OD

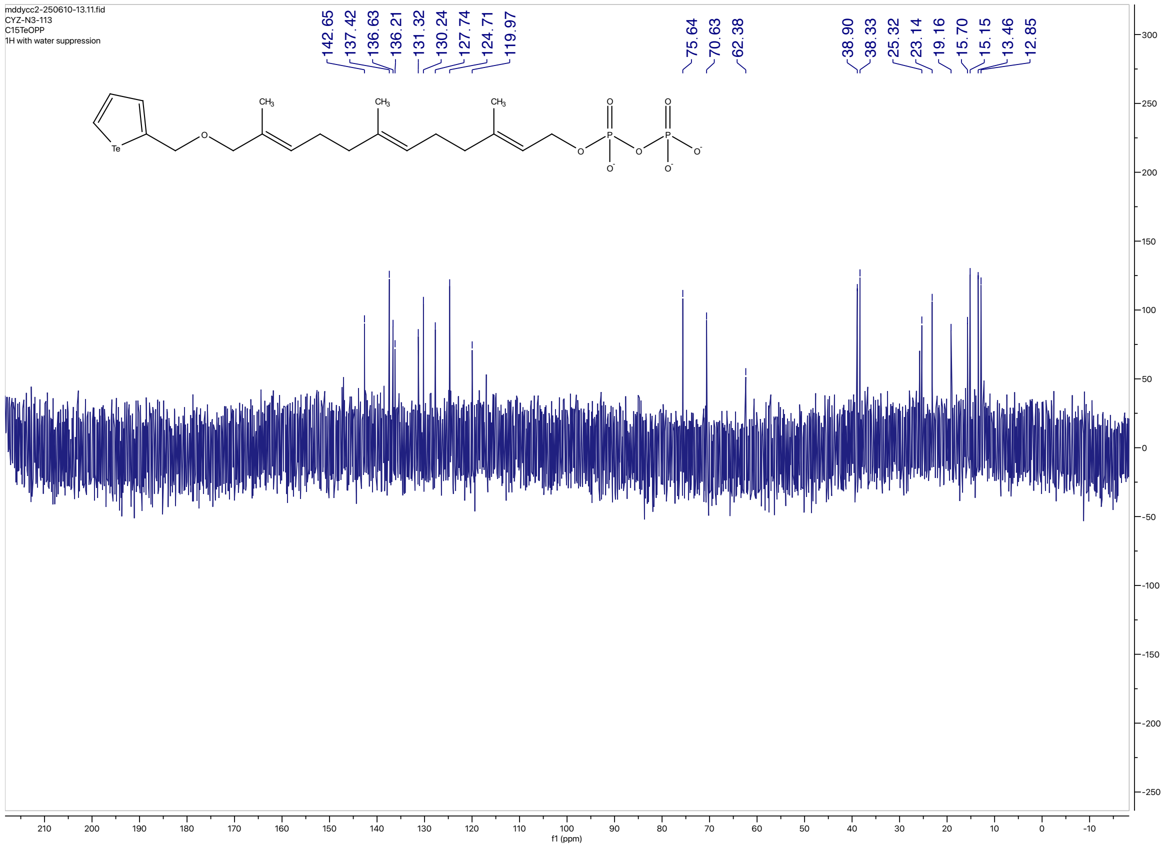

Compound **2a** ^{13}C NMR in D_2O

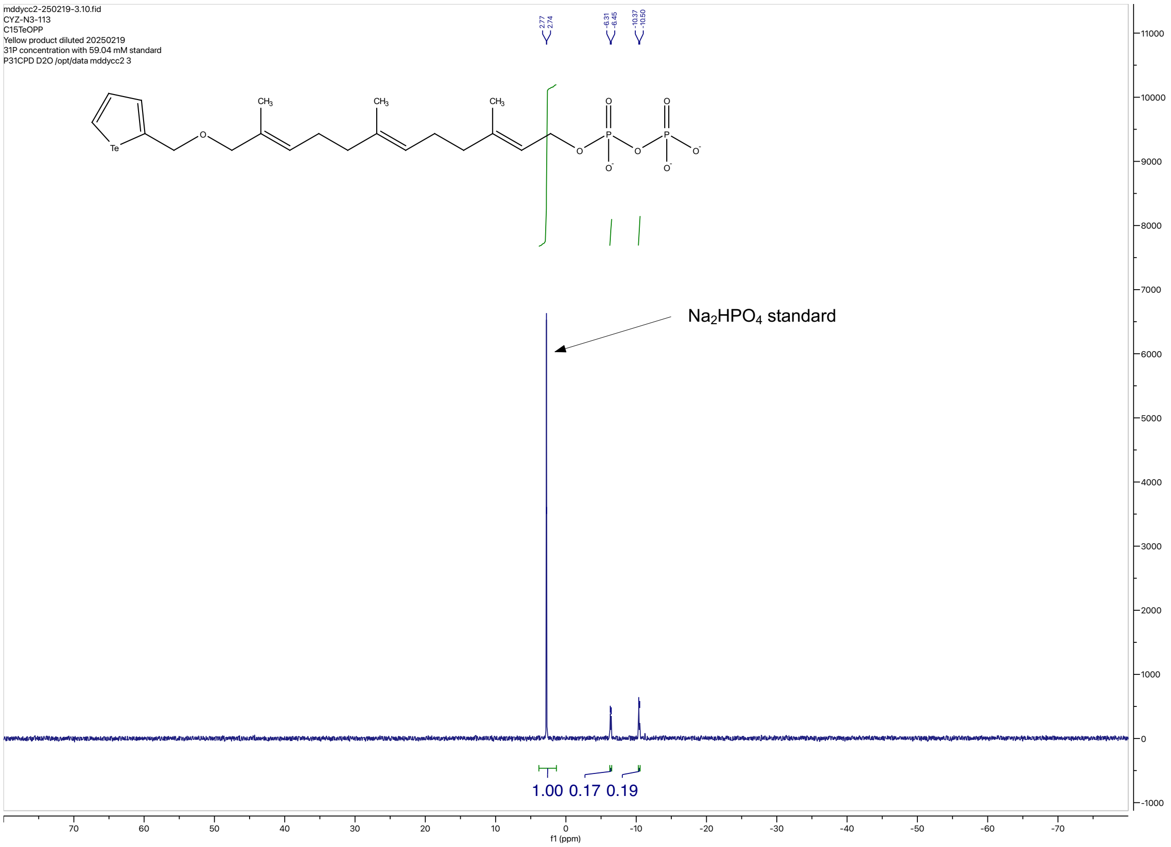

Compound **2a** ^{31}P NMR in D_2O with Na_2HPO_4 as internal standard

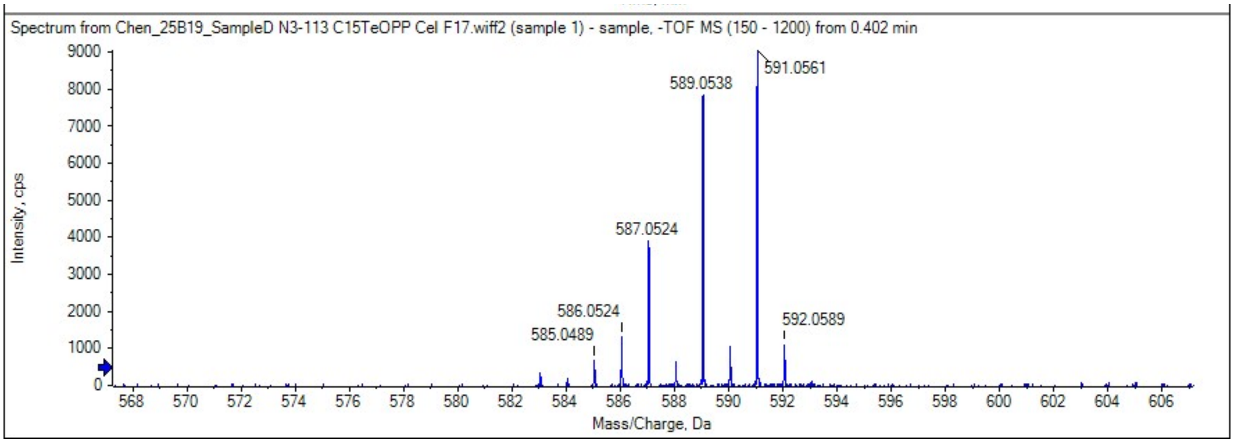

Compound **2a** HR-ESI-MS spectrum

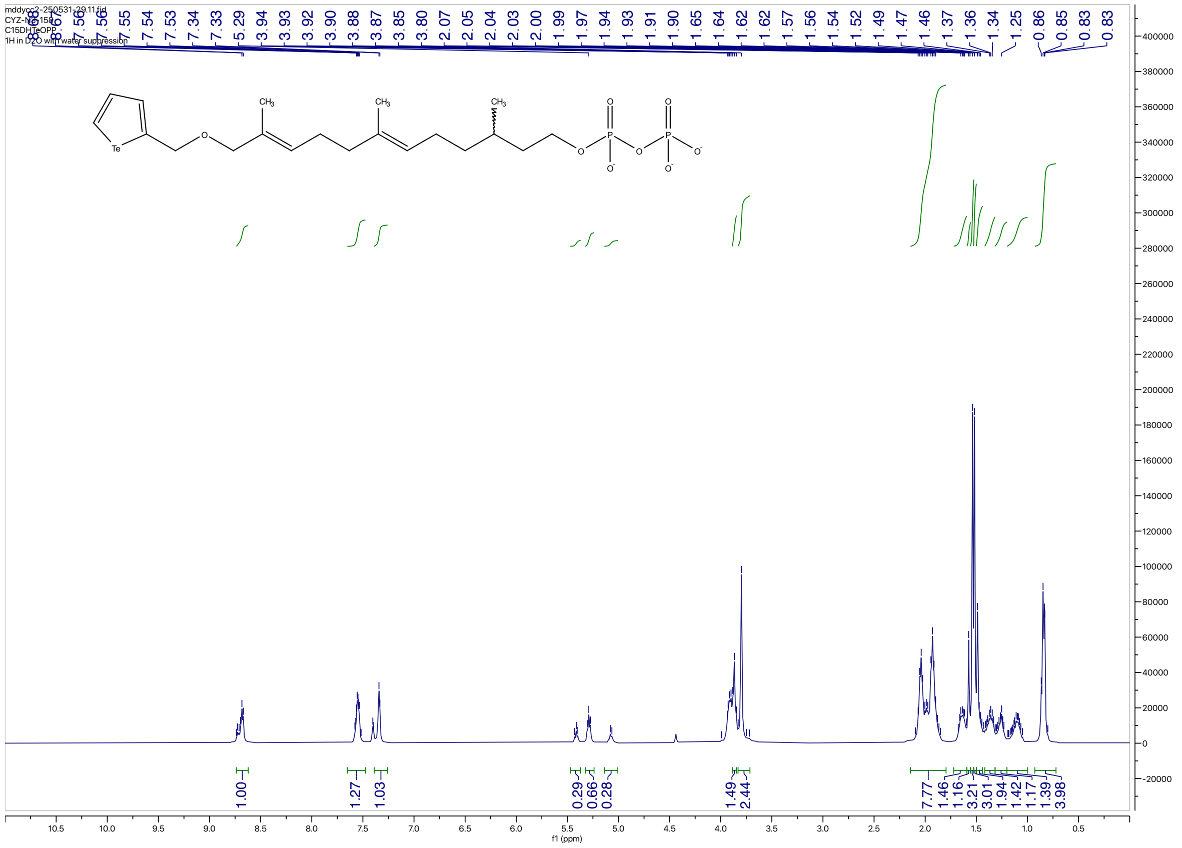

Compound **2b** ^{1}H NMR in D_2O with water suppression

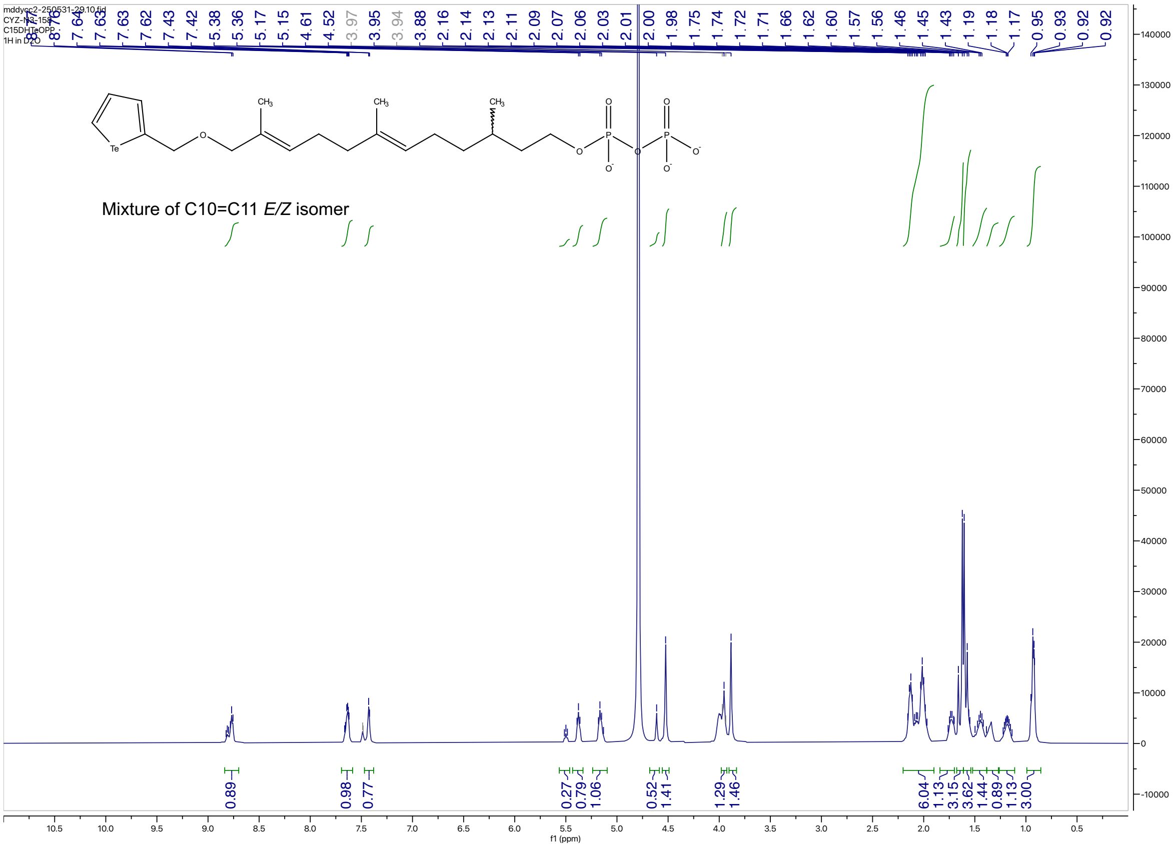

Compound **2b** ^{1}H NMR in D_2O

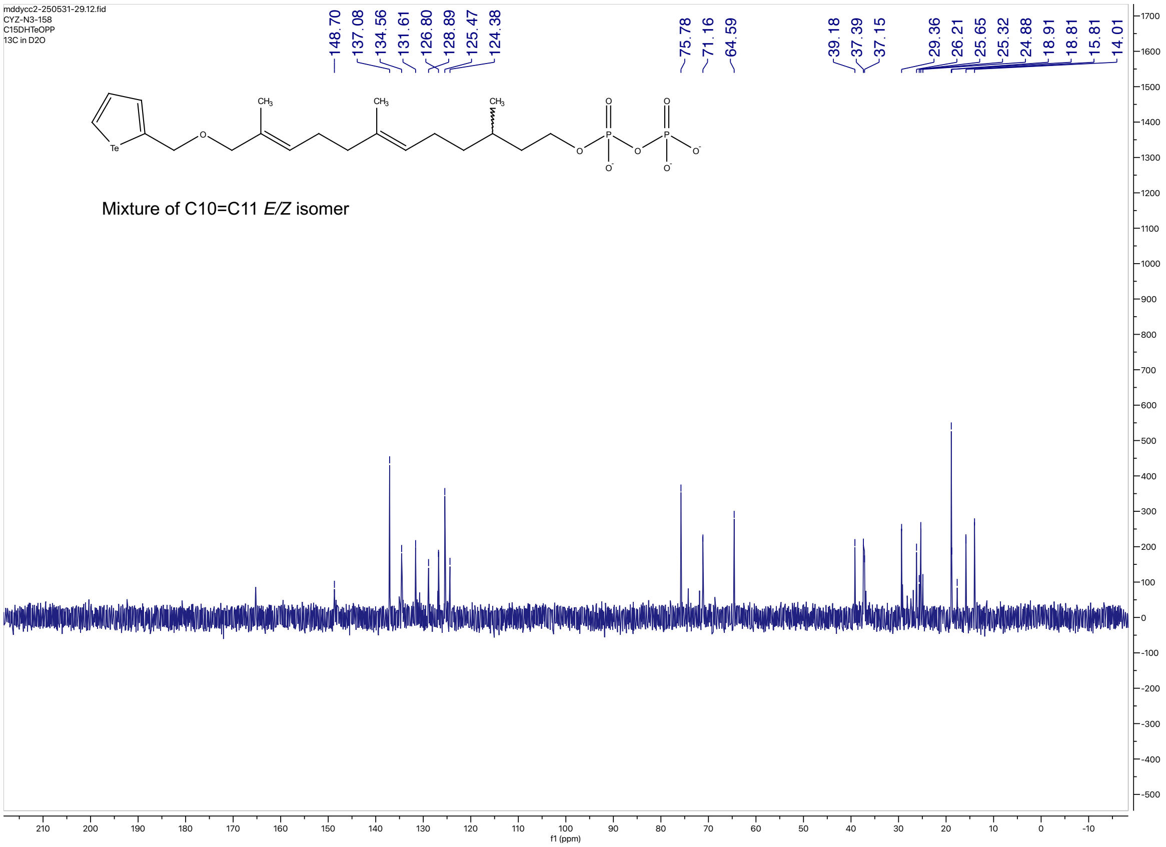

Compound **2b** ^{13}C NMR in D_2O

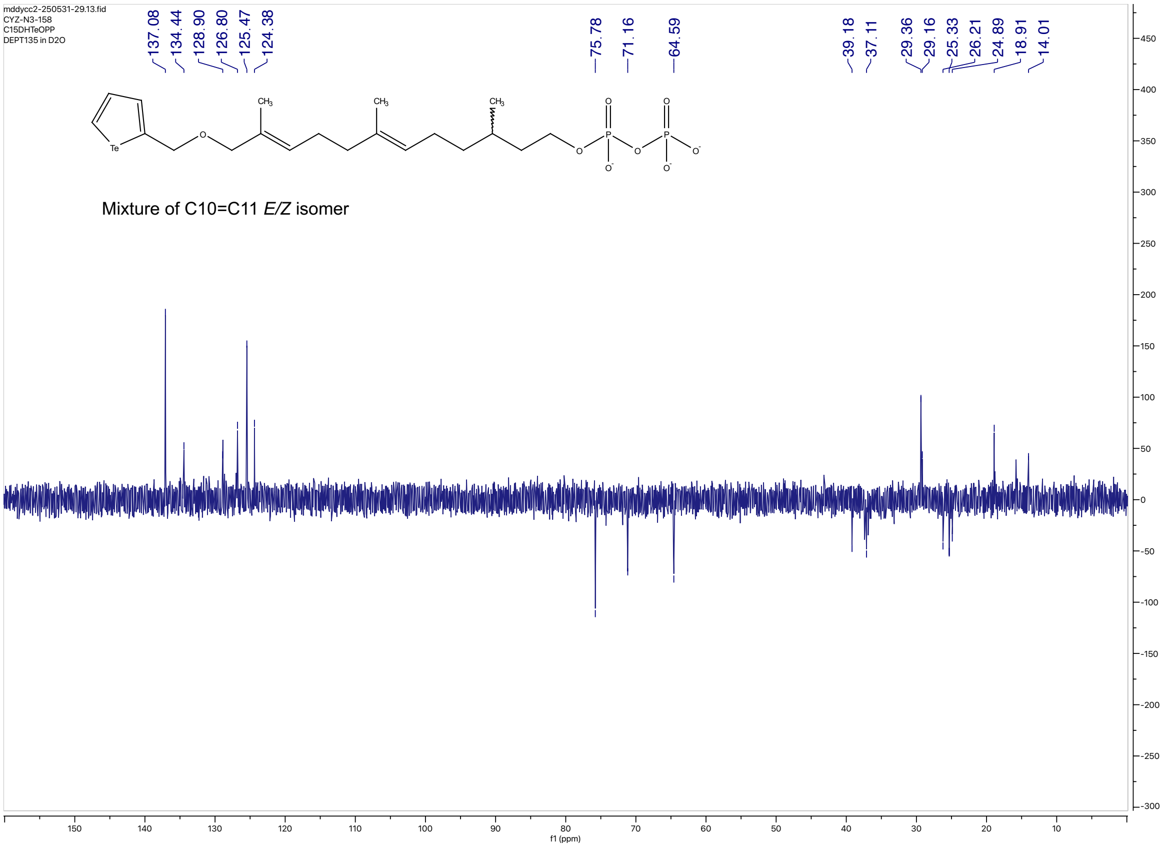

Compound **2b** ^{13}C DEPT135 NMR in D_2O

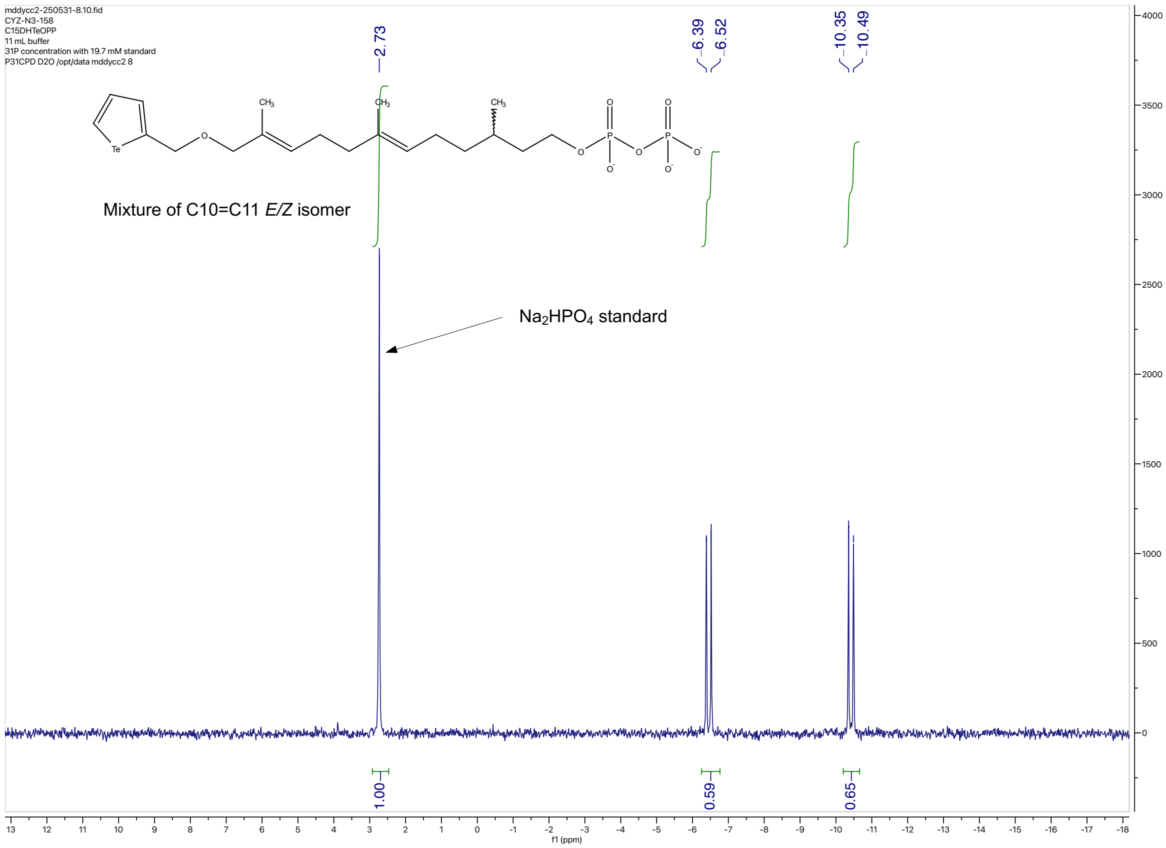

Compound **2b** ^{31}P NMR in D_2O with Na_2HPO_4 as internal standard

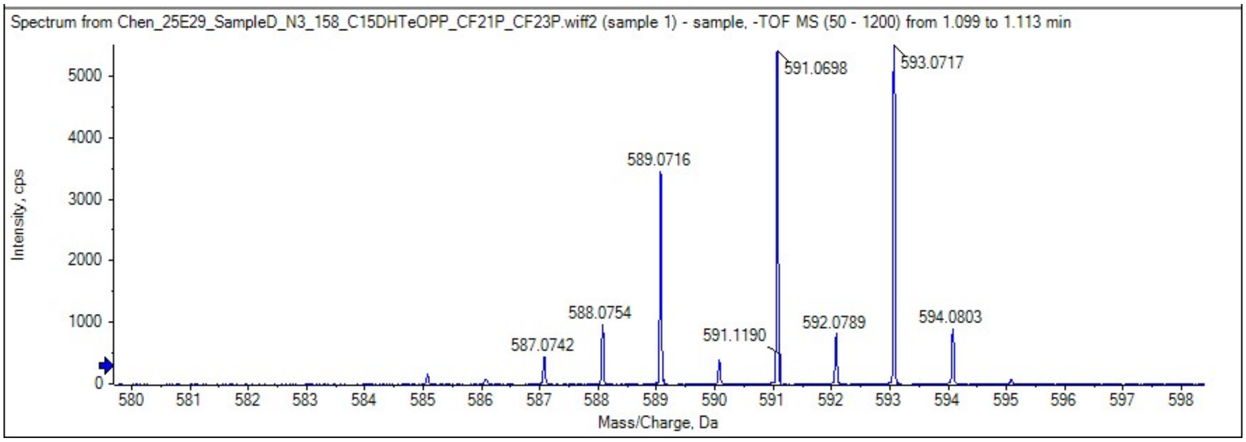

Compound **2b** HR-ESI-MS spectrum

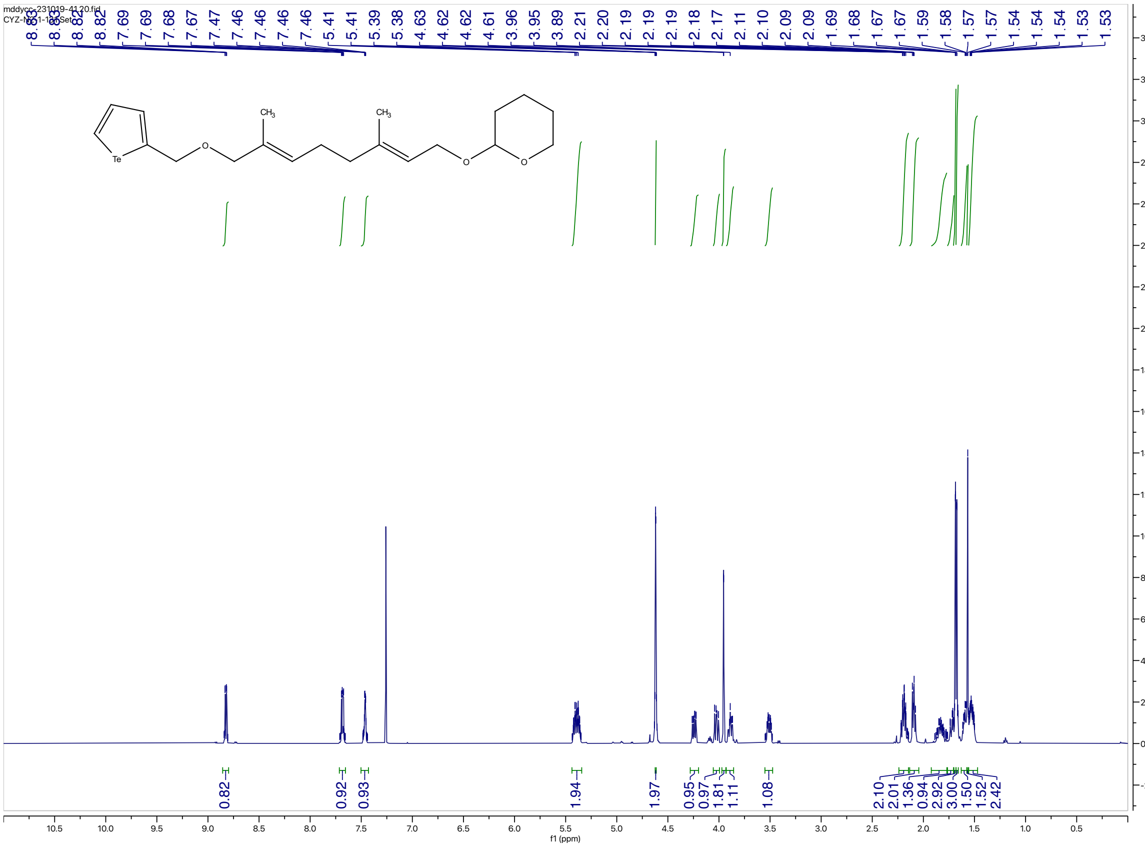

Compound **16** ^1H NMR in $CDCl_3$

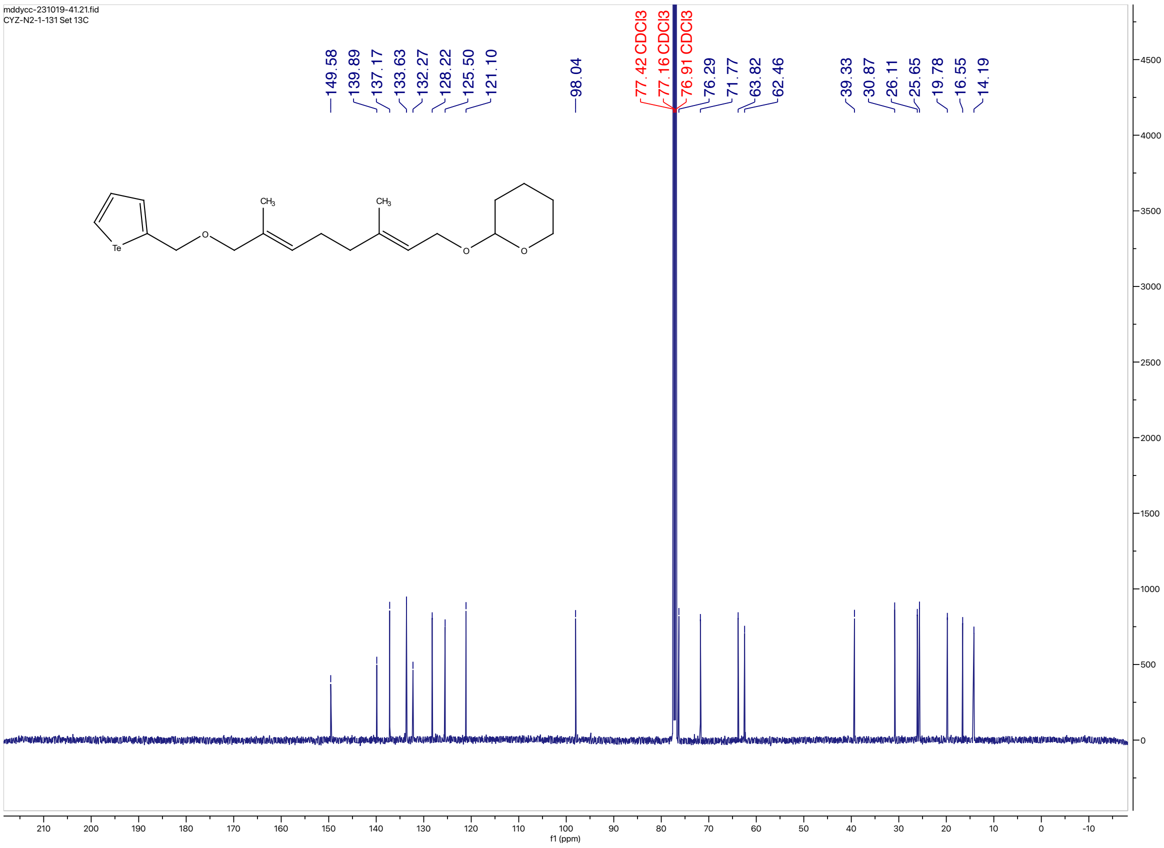

Compound **16** ^{13}C NMR in $CDCl_3$

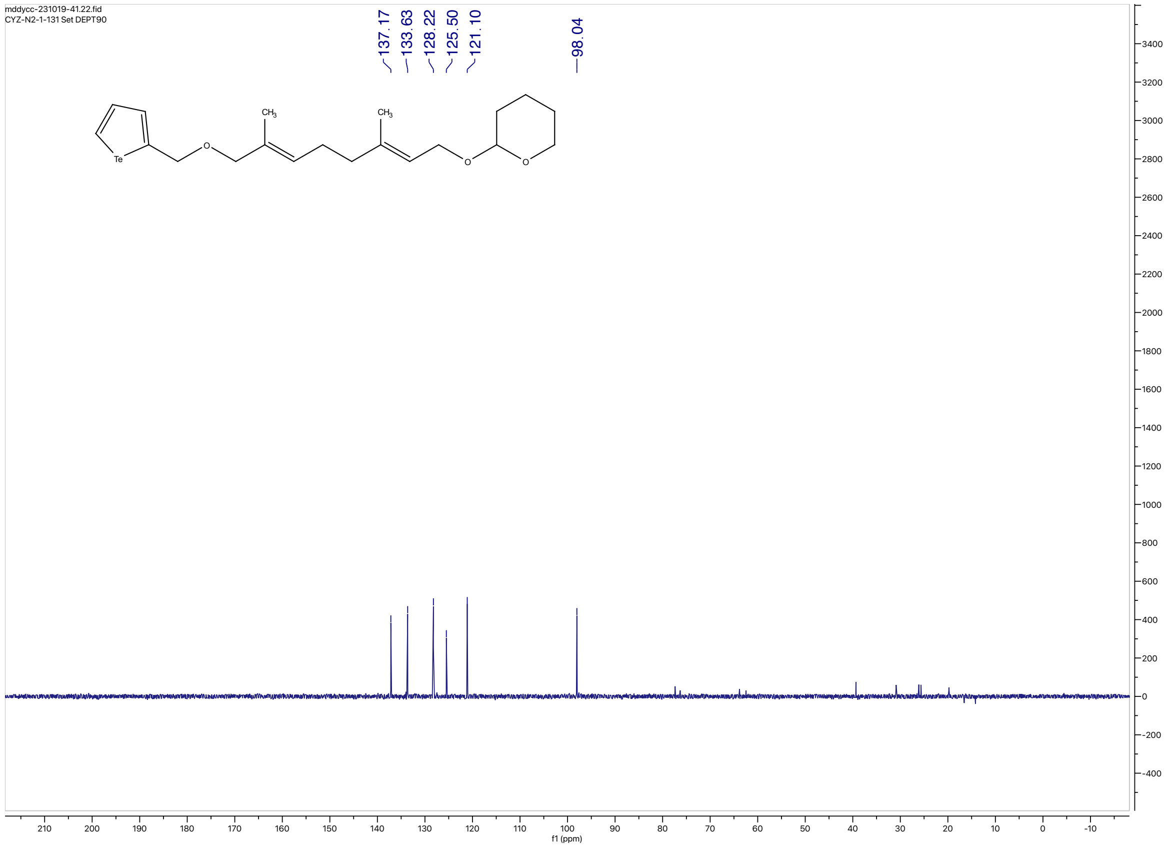

Compound **16** ^{13}C DEPT90 NMR in $CDCl_3$

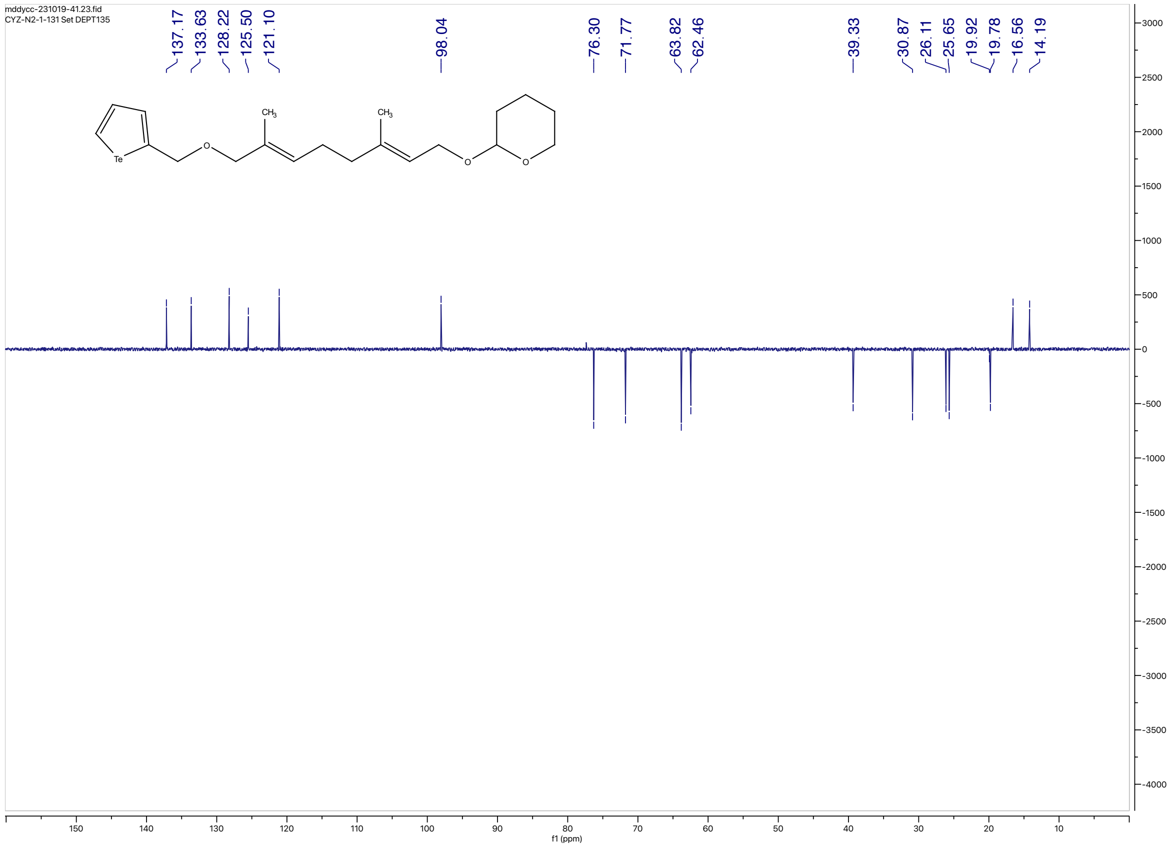

Compound **16** ^{13}C DEPT135 NMR in $CDCl_3$

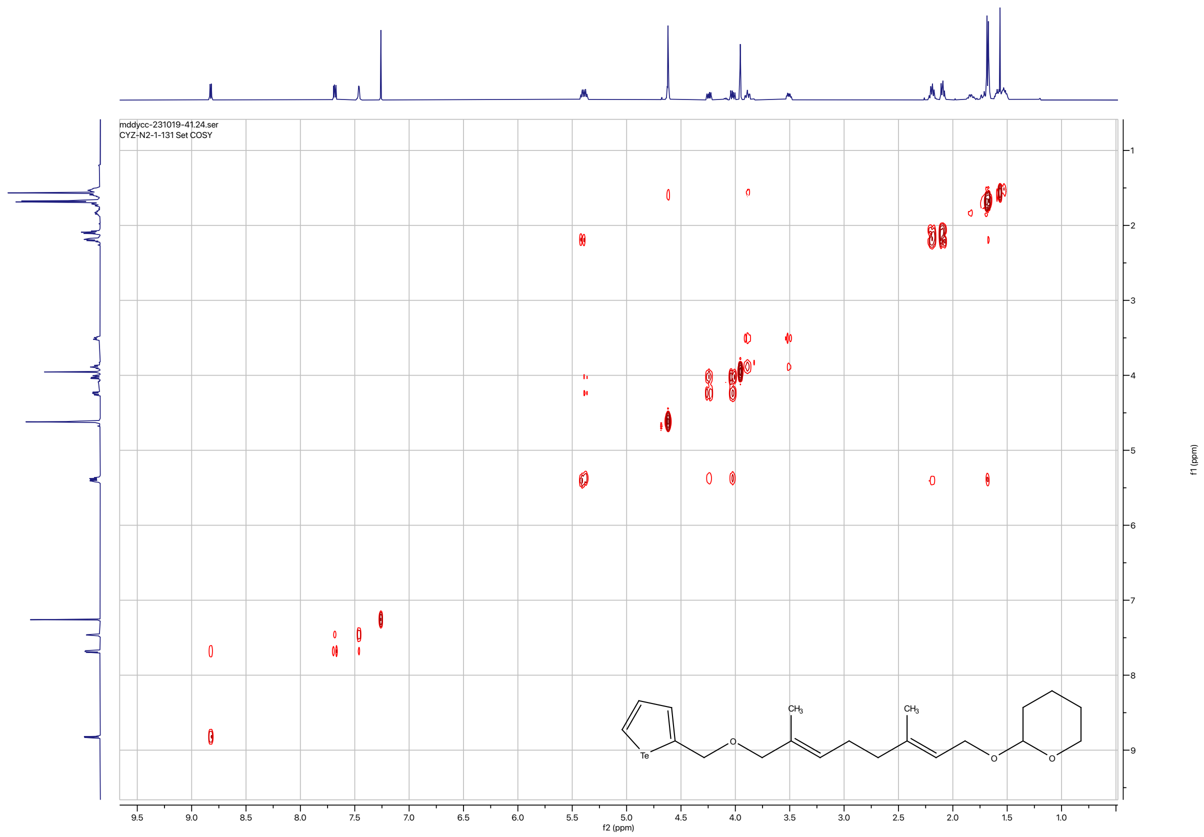

Compound **16** ^{1}H-^{1}H COSY NMR in $CDCl_3$

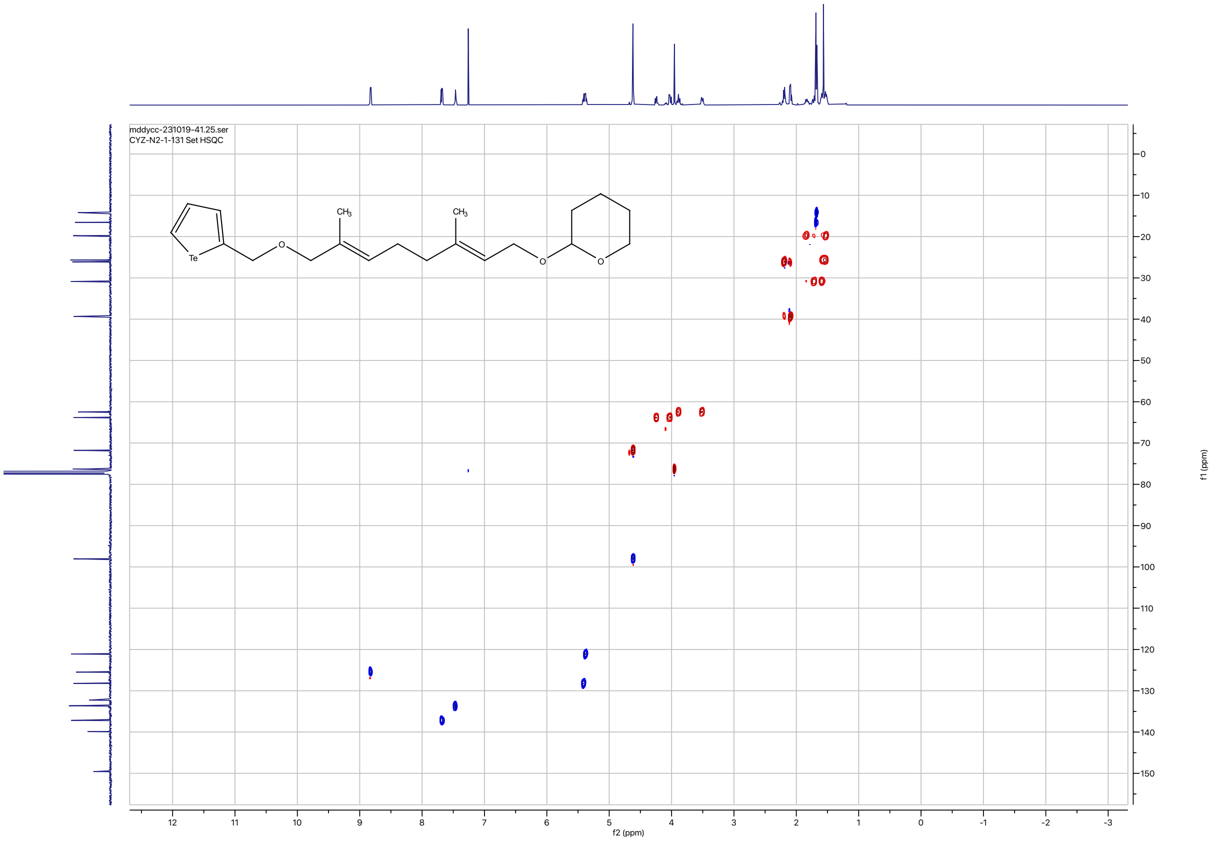

Compound **16** ^{1}H-^{13}C HSQC NMR in $CDCl_3$

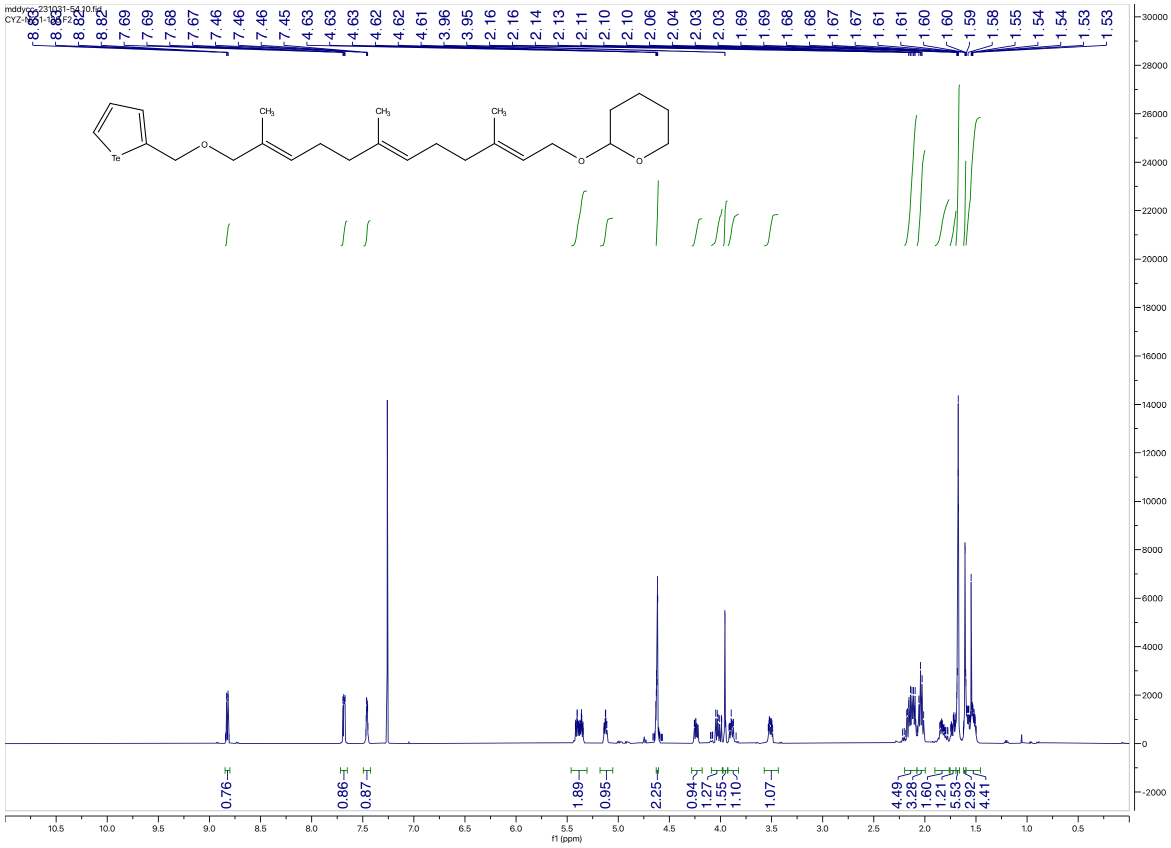

Compound **17** ^{1}H NMR in $CDCl_3$

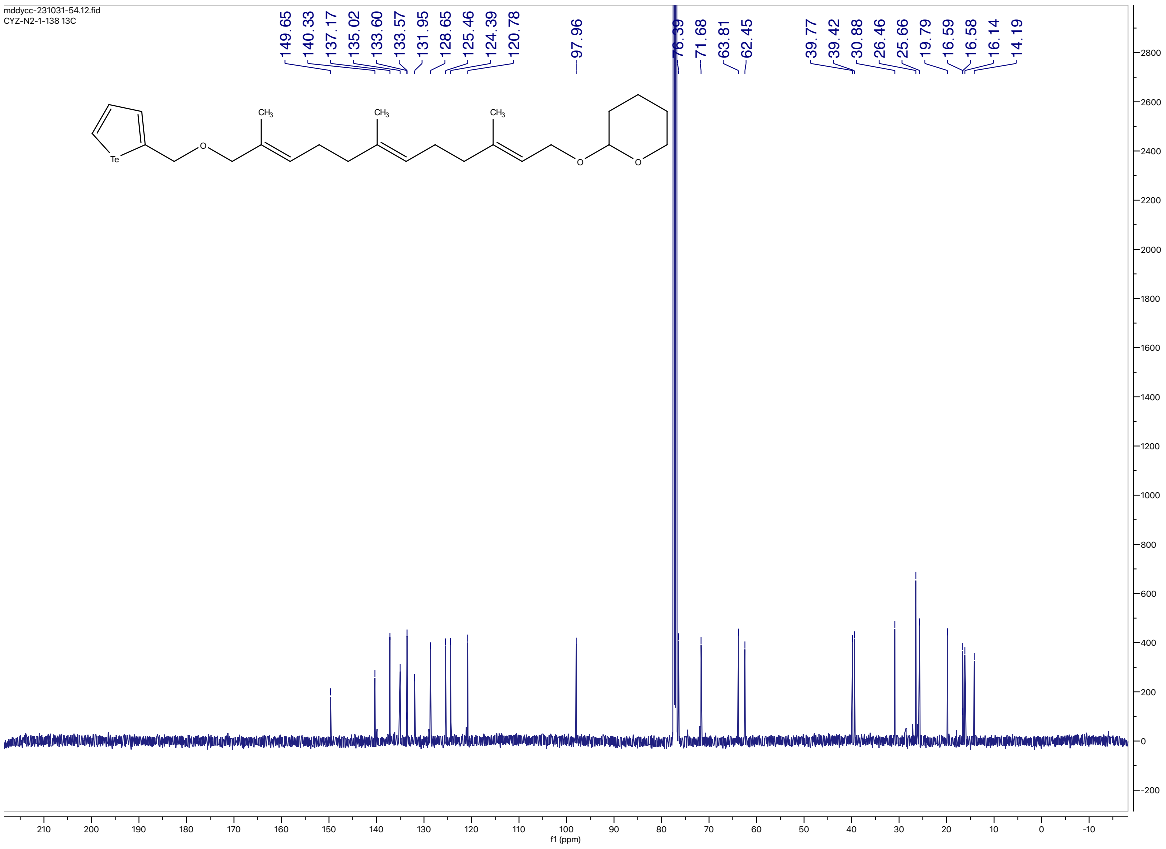

Compound **17** ^{13}C NMR in $CDCl_3$

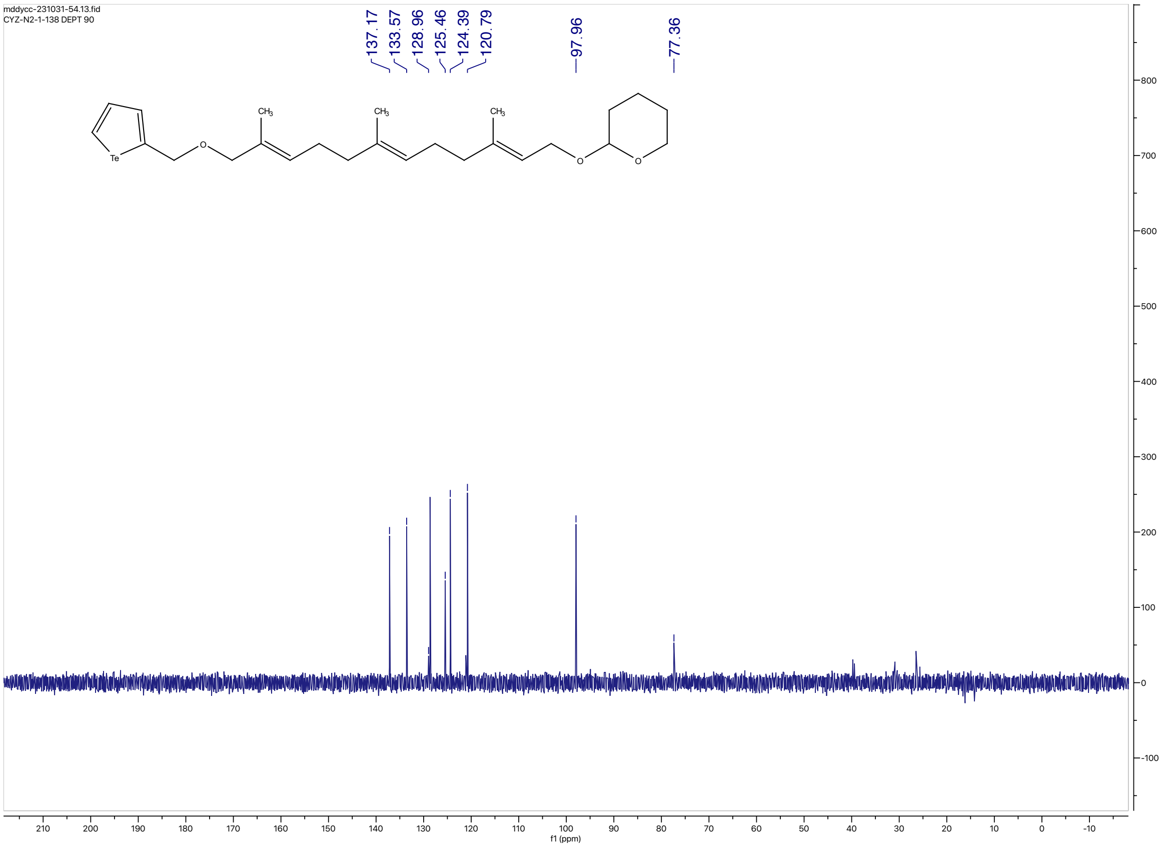

Compound **17** ^{13}C DEPT90 NMR in $CDCl_3$

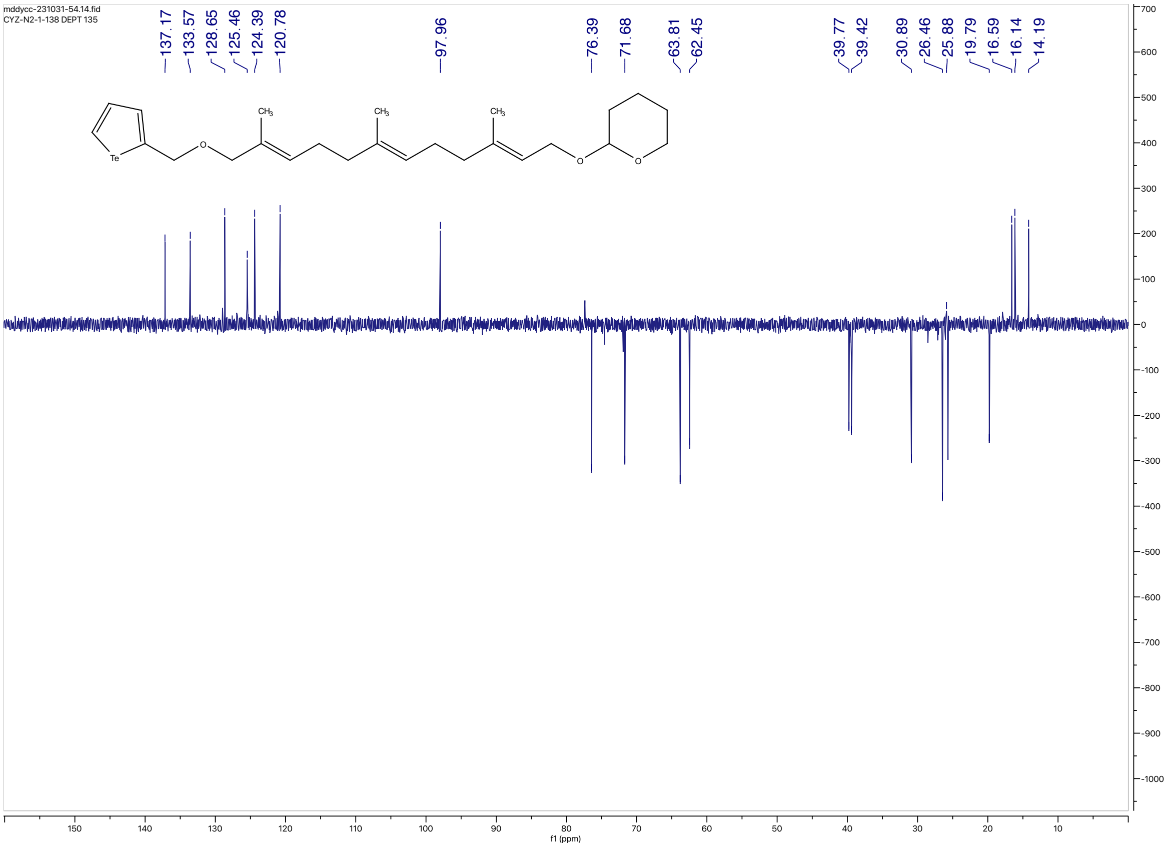

Compound **17** ^{13}C DEPT135 NMR in $CDCl_3$

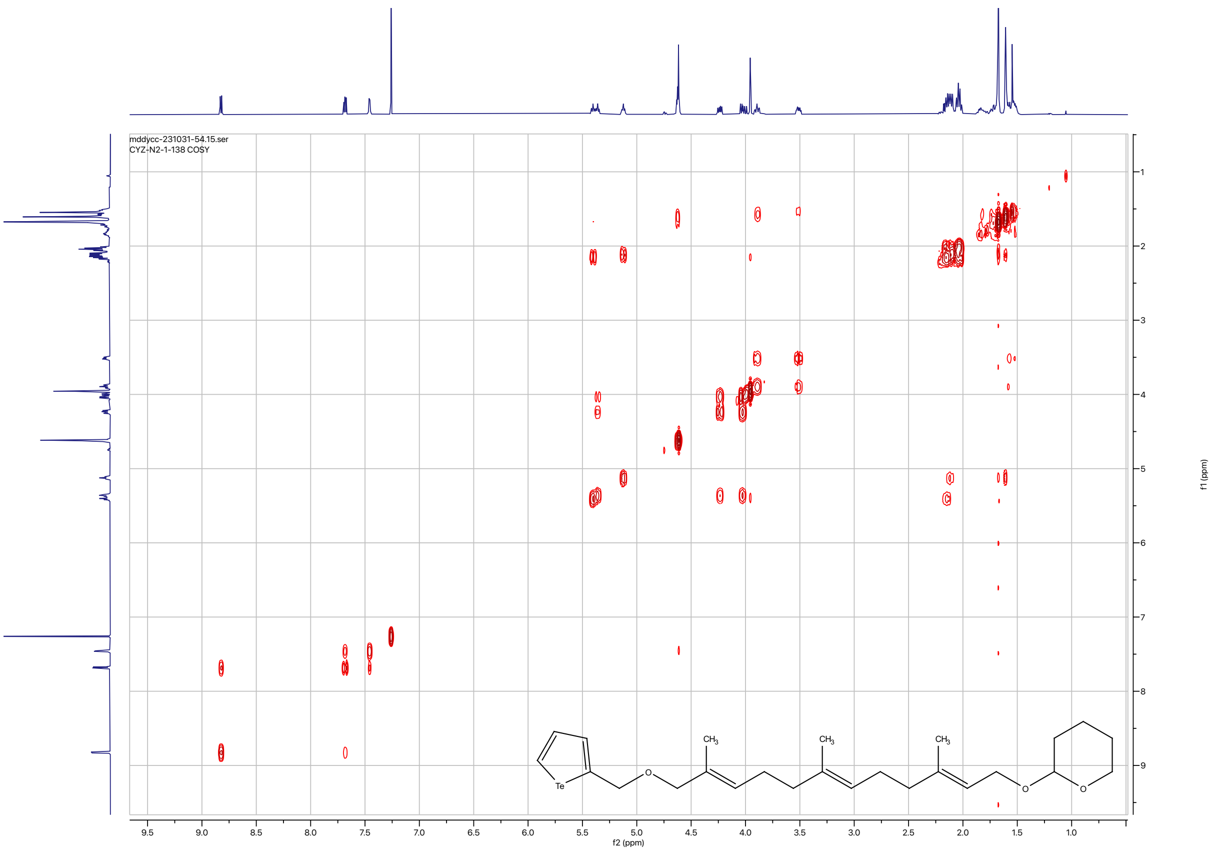

Compound **17** ^{1}H-^{1}H COSY NMR in $CDCl_3$

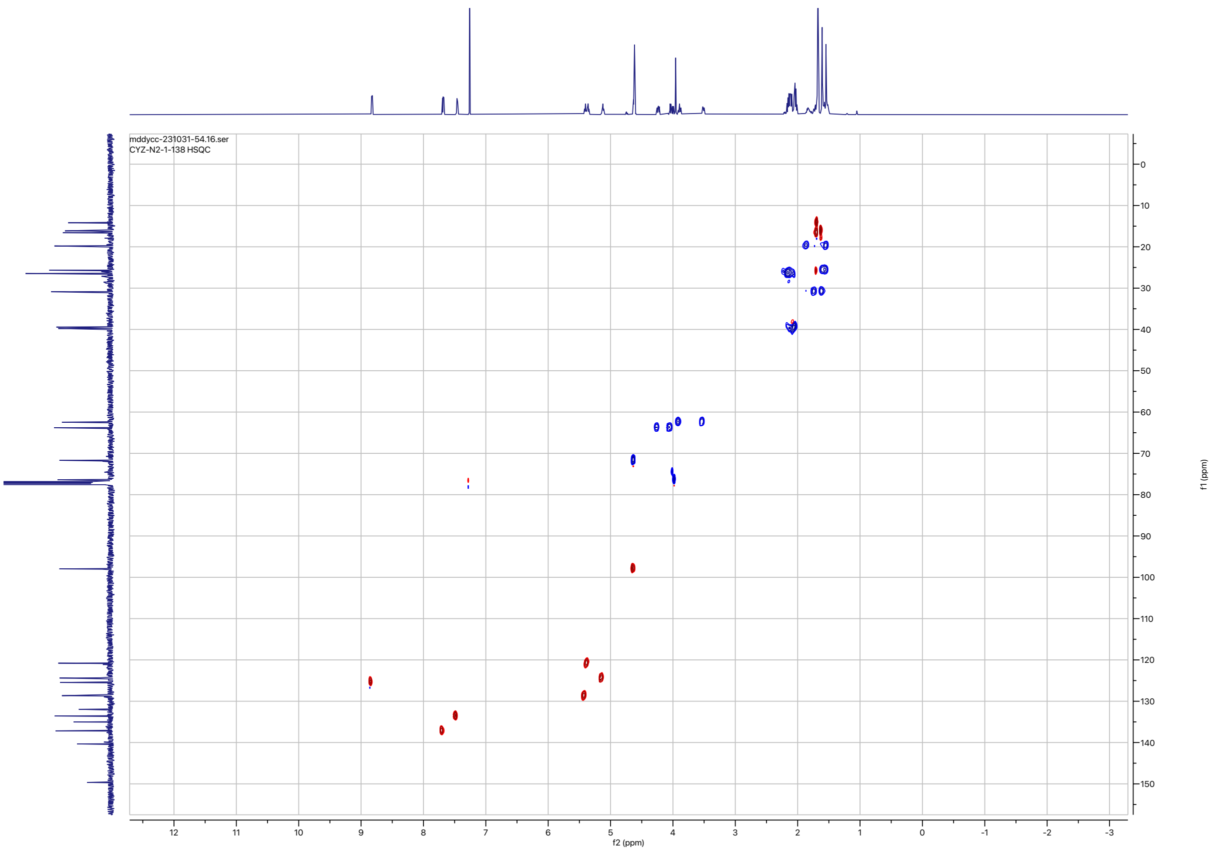

Compound **17** ^{1}H-^{13}C HSQC NMR in $CDCl_3$

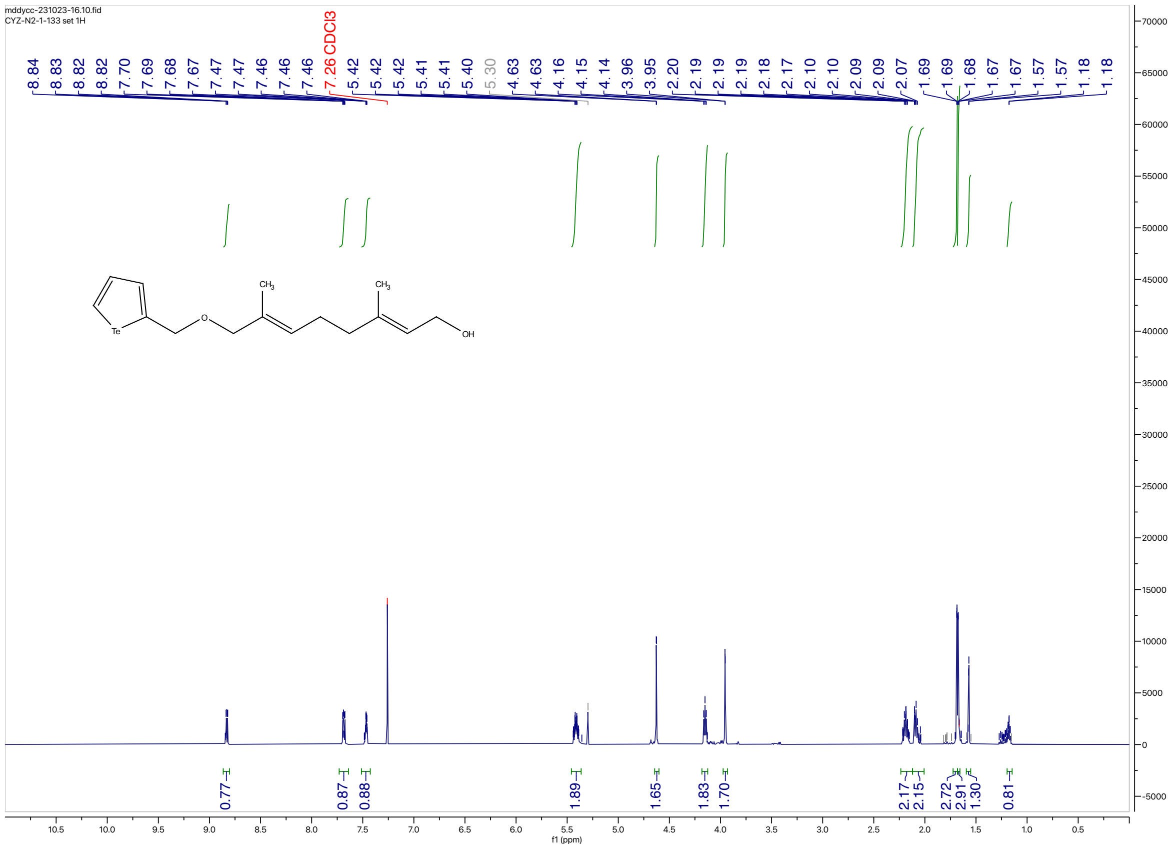

Compound **18** ^{1}H NMR in $CDCl_3$

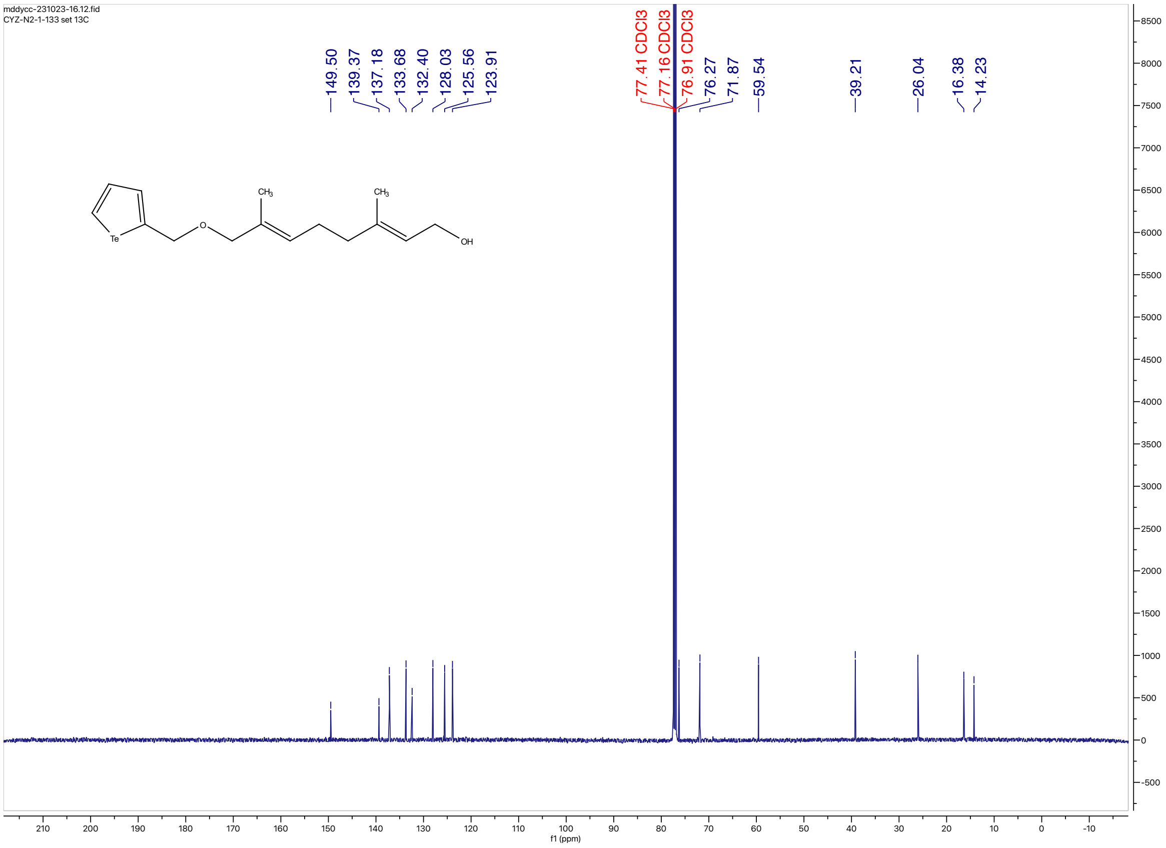

Compound **18** ^{13}C NMR in $CDCl_3$

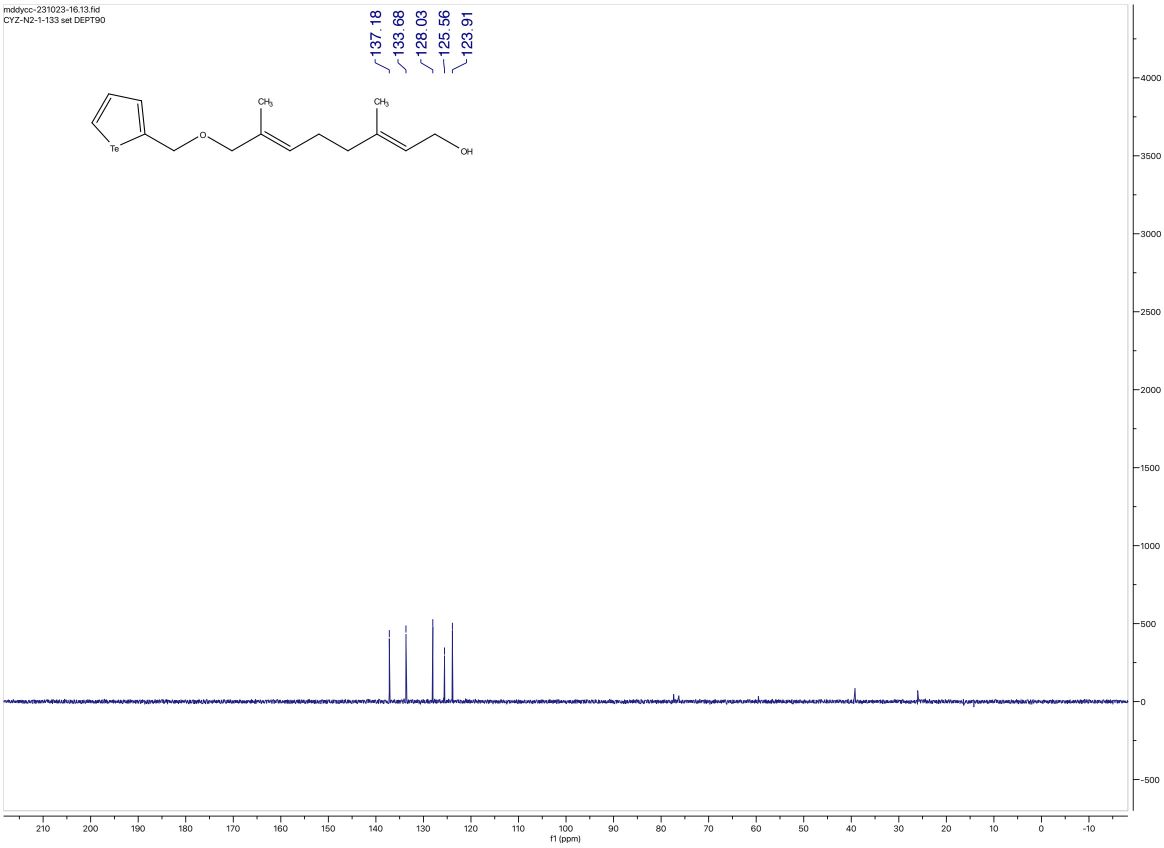

Compound **18** ^{13}C DEPT90 NMR in $CDCl_3$

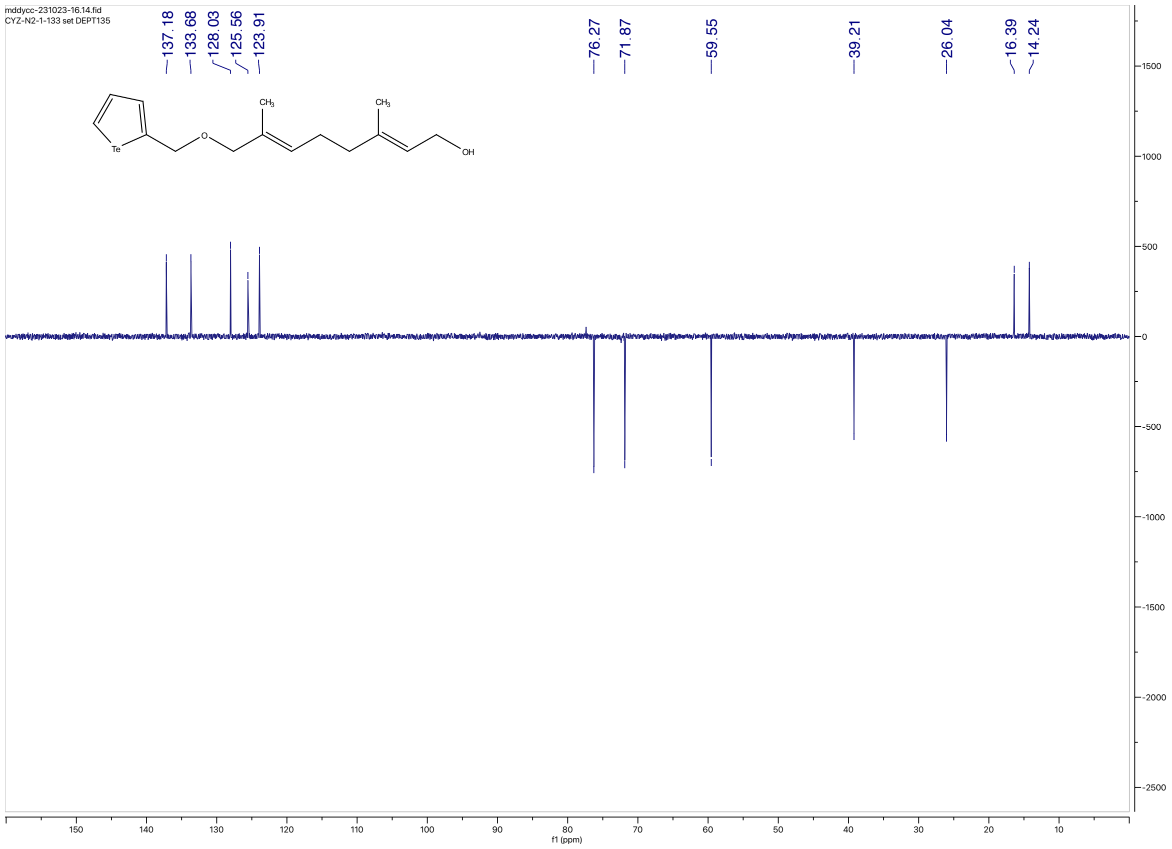

Compound **18** ^{13}C DEPT135 NMR in $CDCl_3$

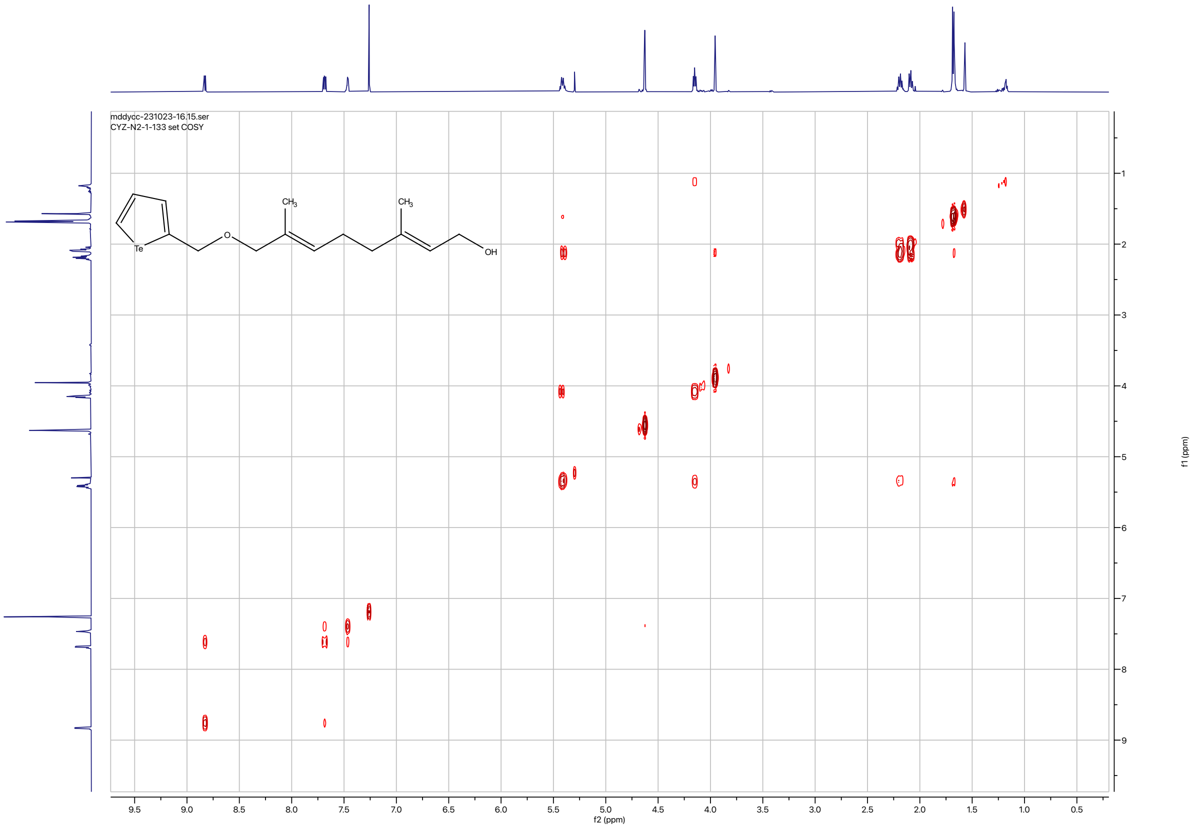

Compound **18** ^1H-^1H COSY NMR in $CDCl_3$

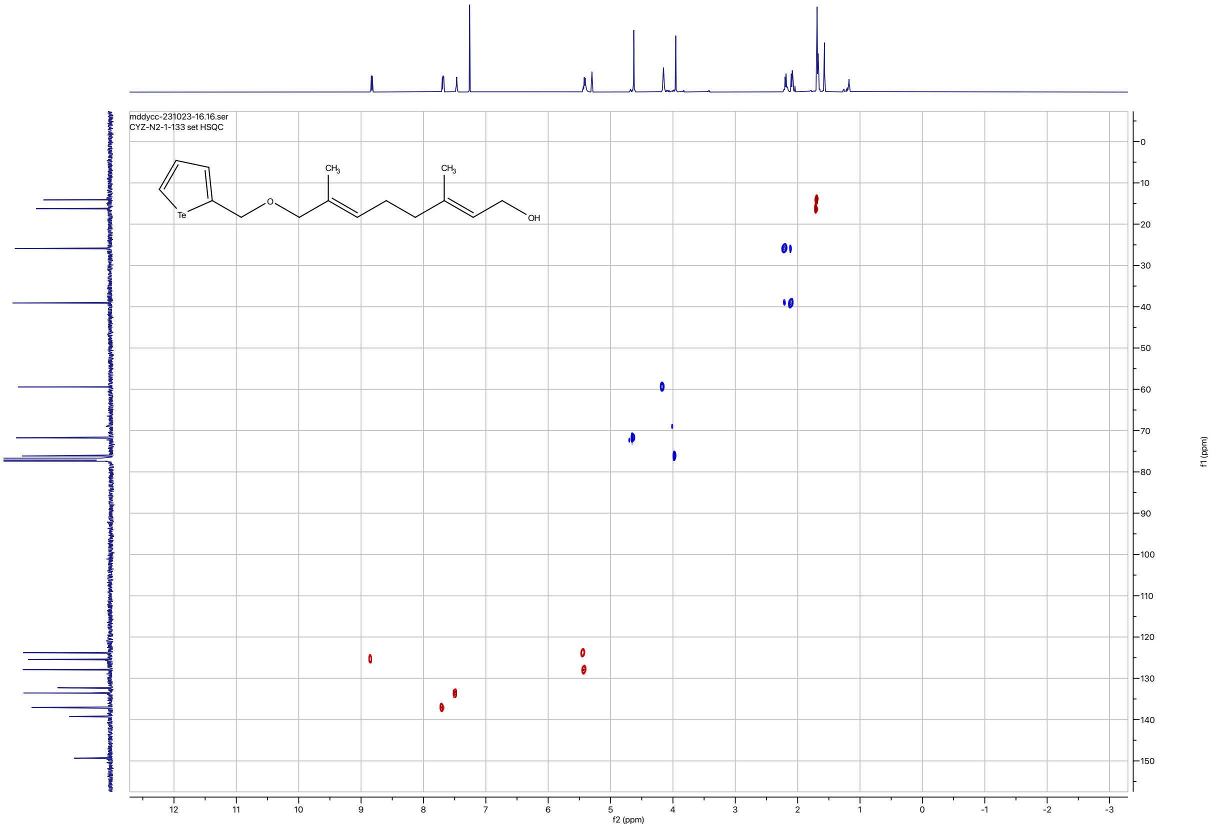

Compound **18** ^{1}H-^{13}C HSQC NMR in $CDCl_3$

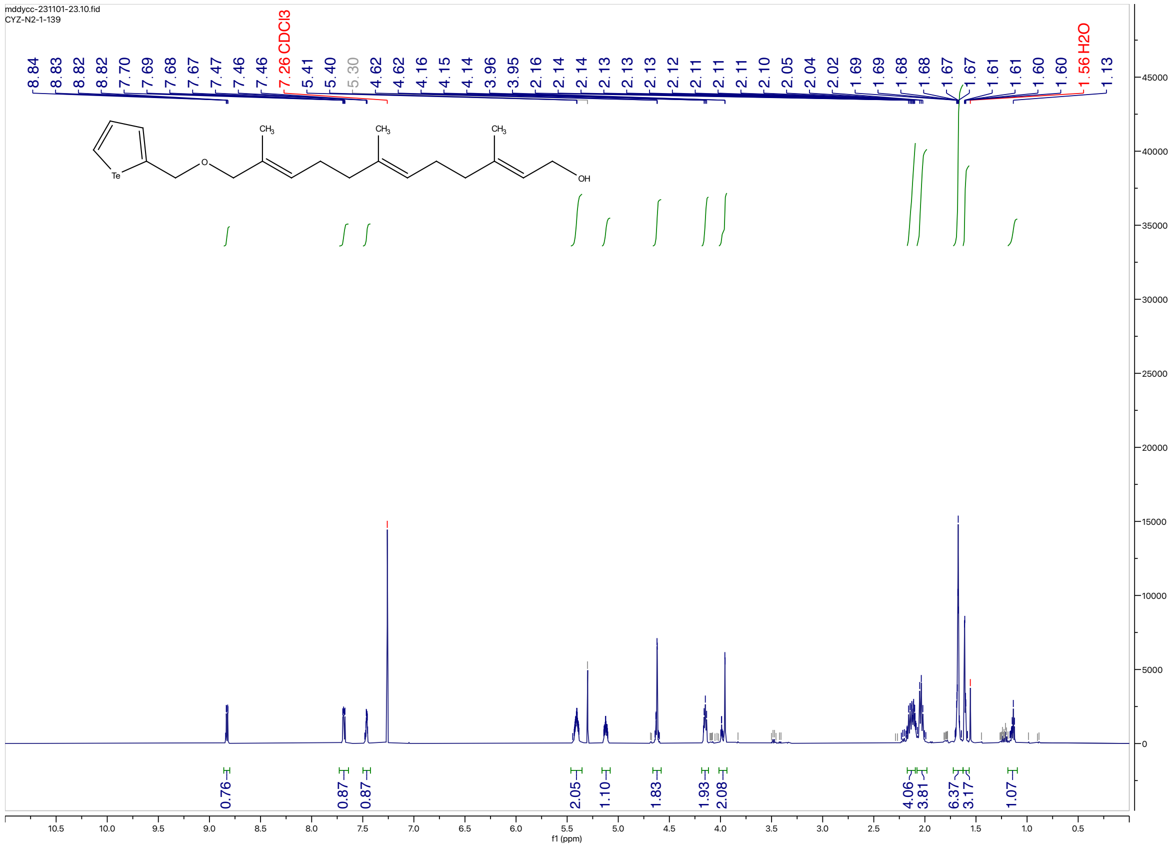

Compound **19** ^{1}H NMR in $CDCl_3$

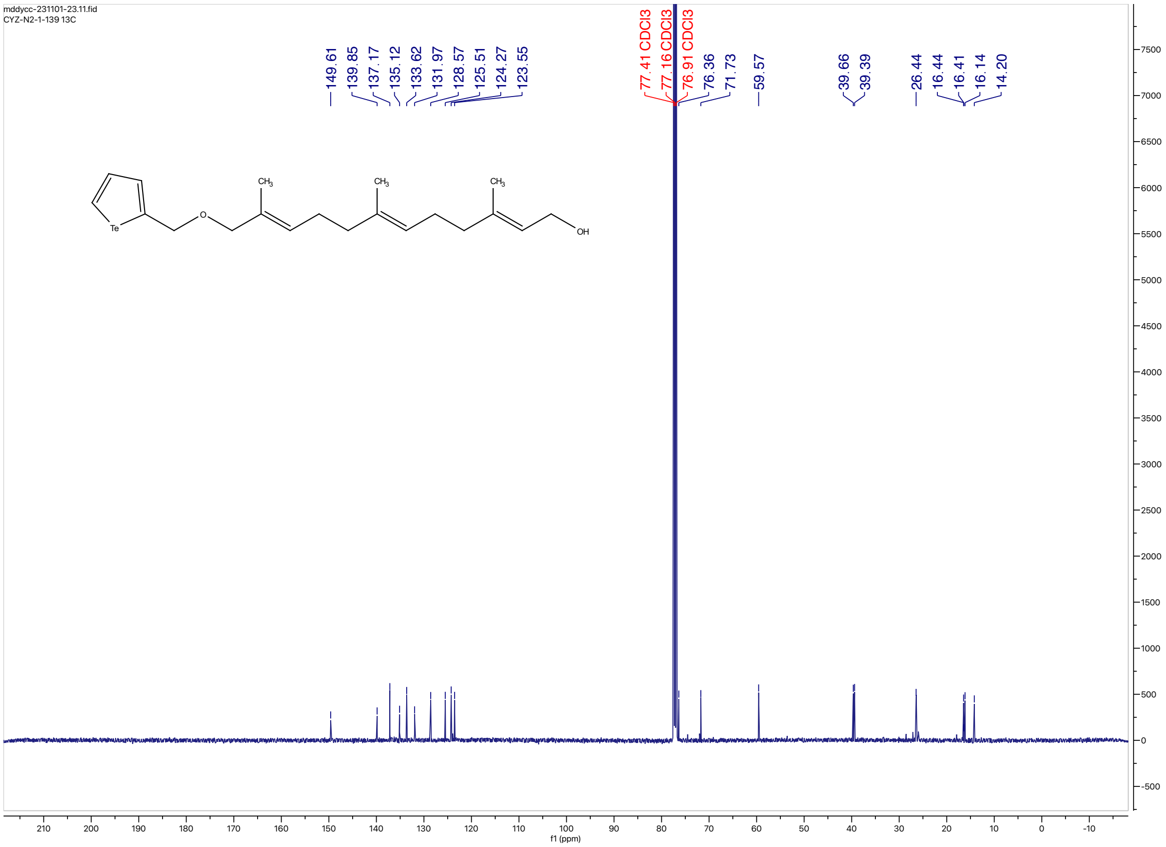

Compound **19** ^{13}C NMR in $CDCl_3$

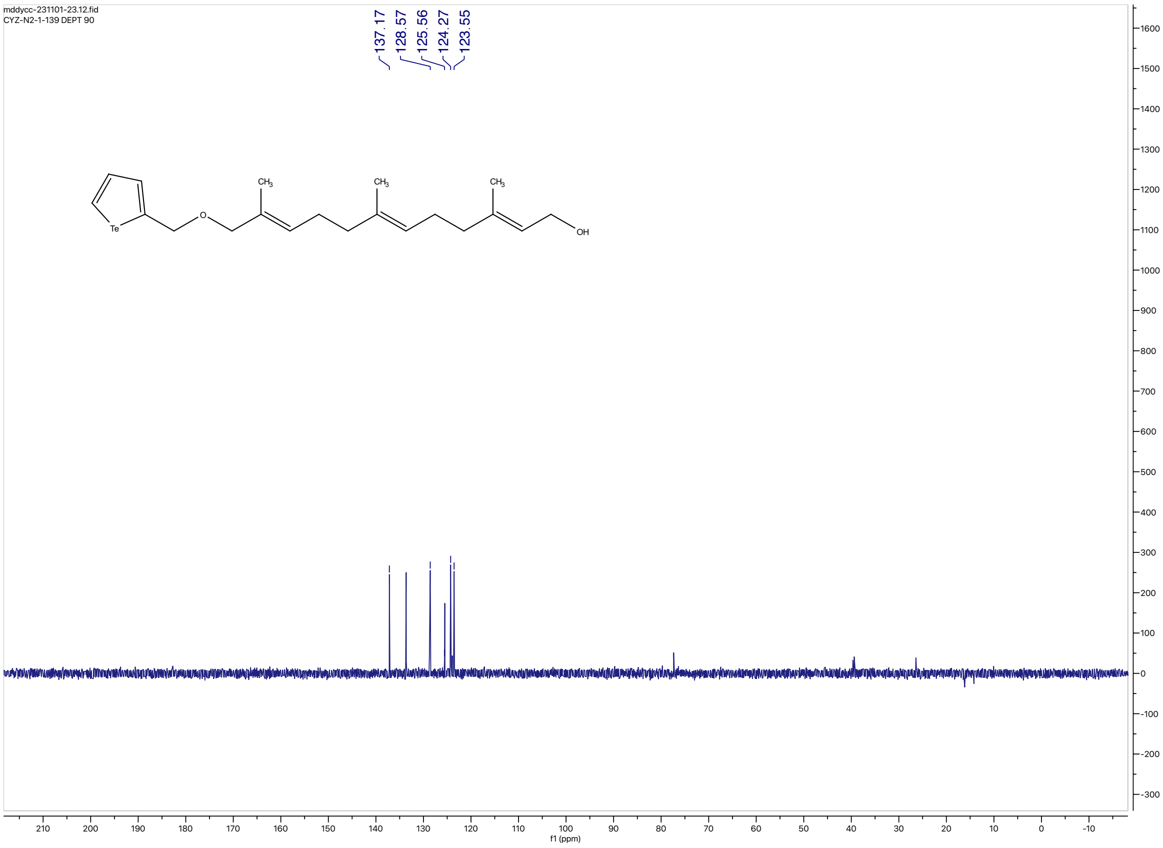

Compound **19** ^{13}C DEPT90 NMR in $CDCl_3$

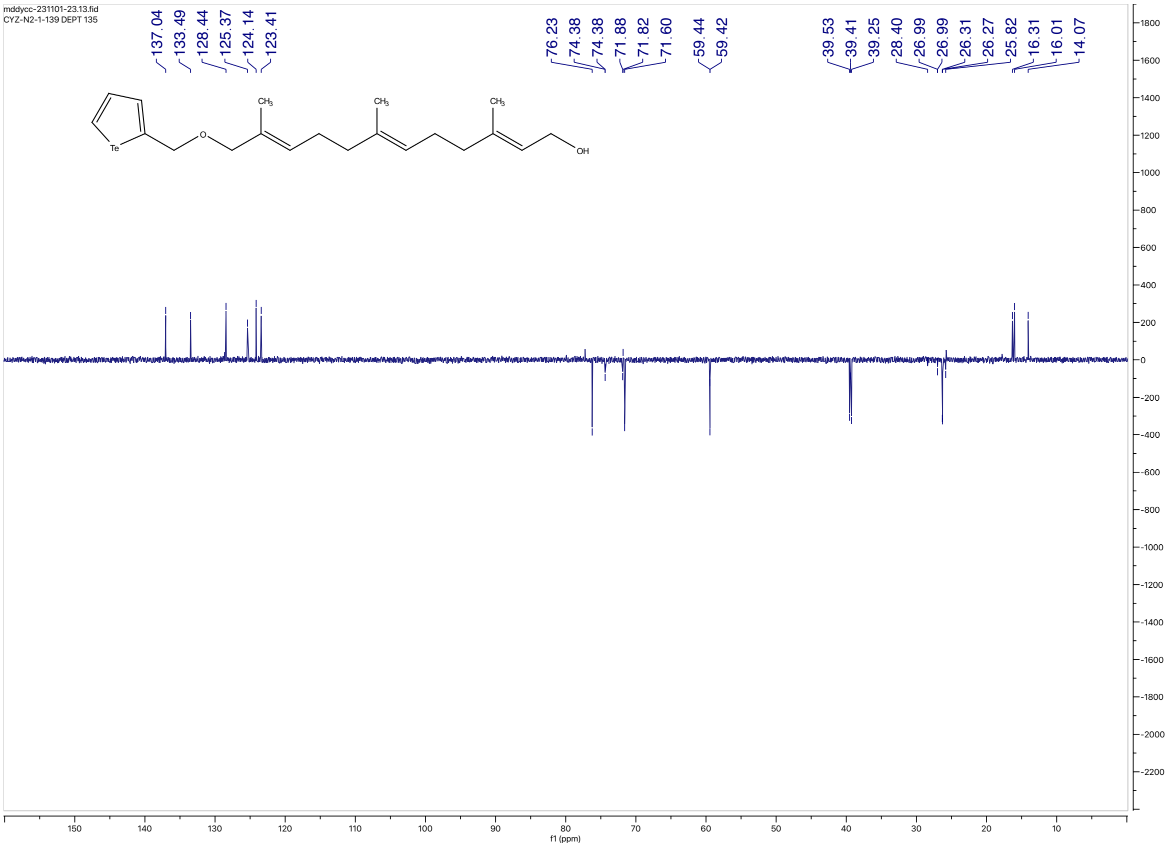

Compound **19** ^{13}C DEPT135 NMR in $CDCl_3$

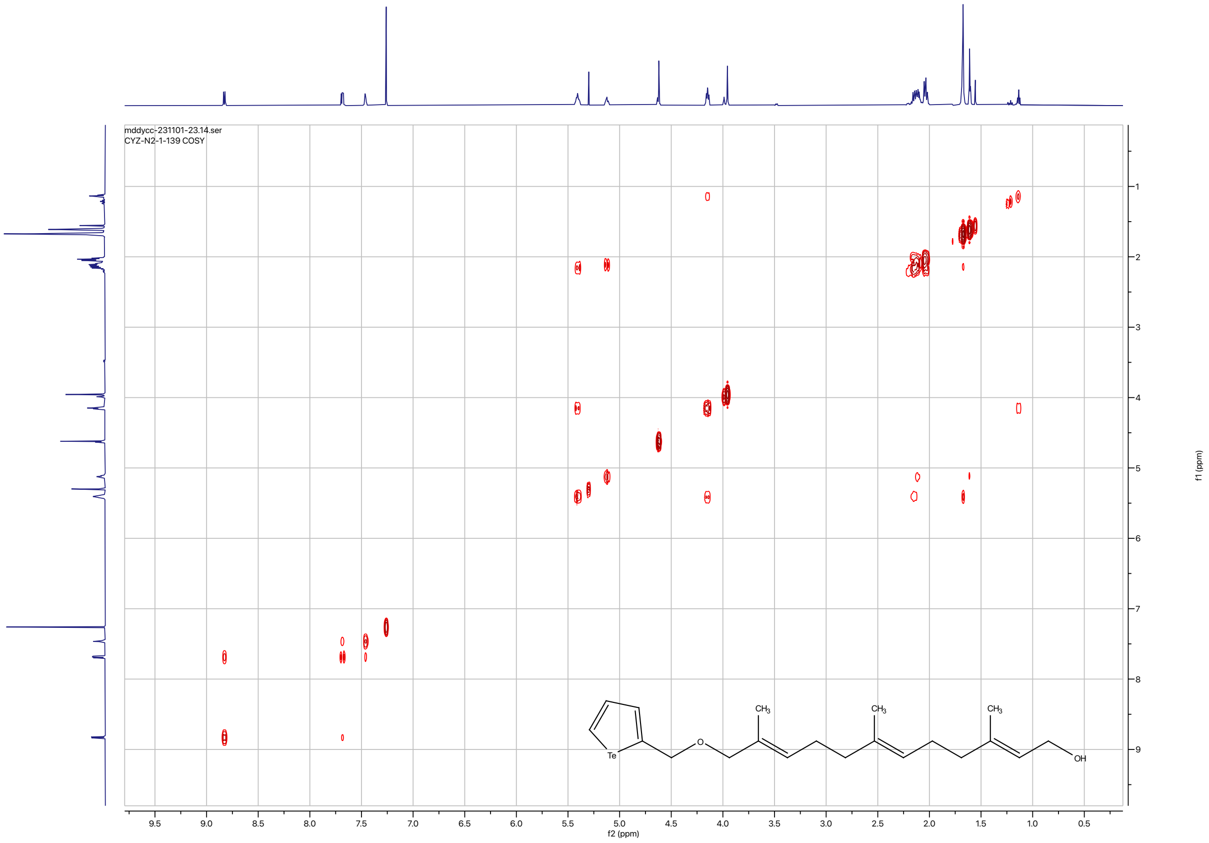

Compound **19** ^{1}H-^{1}H COSY NMR in $CDCl_3$

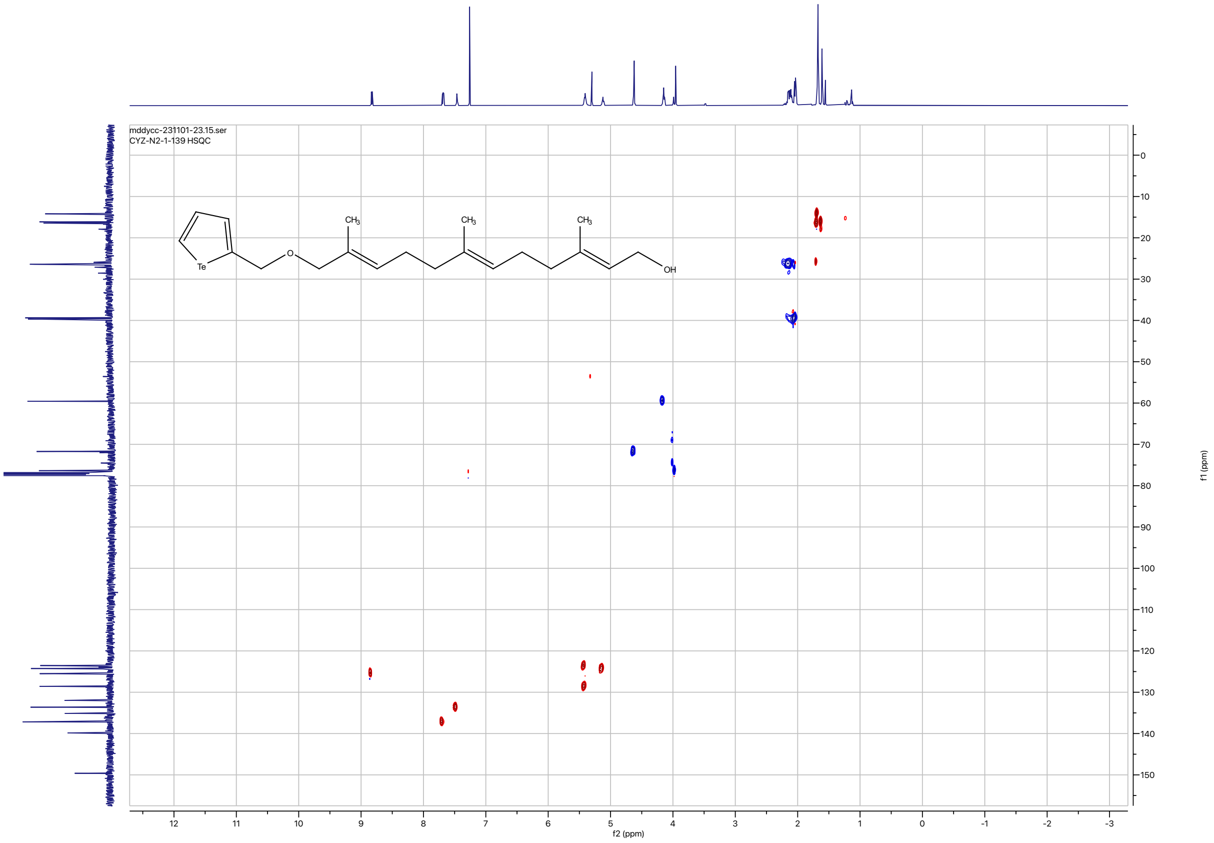

Compound **19** ^{1}H-^{13}C HSQC NMR in $CDCl_3$

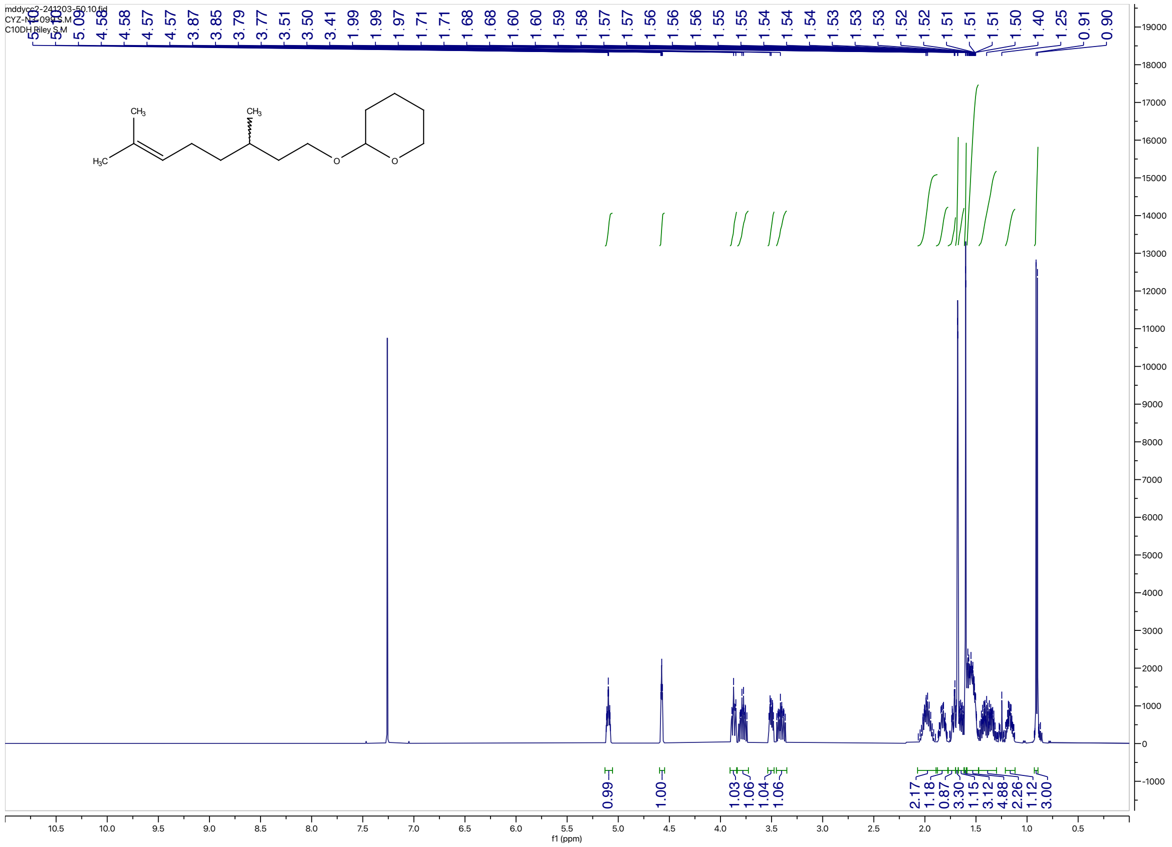

Compound **24** ^{1}H NMR in $CDCl_3$

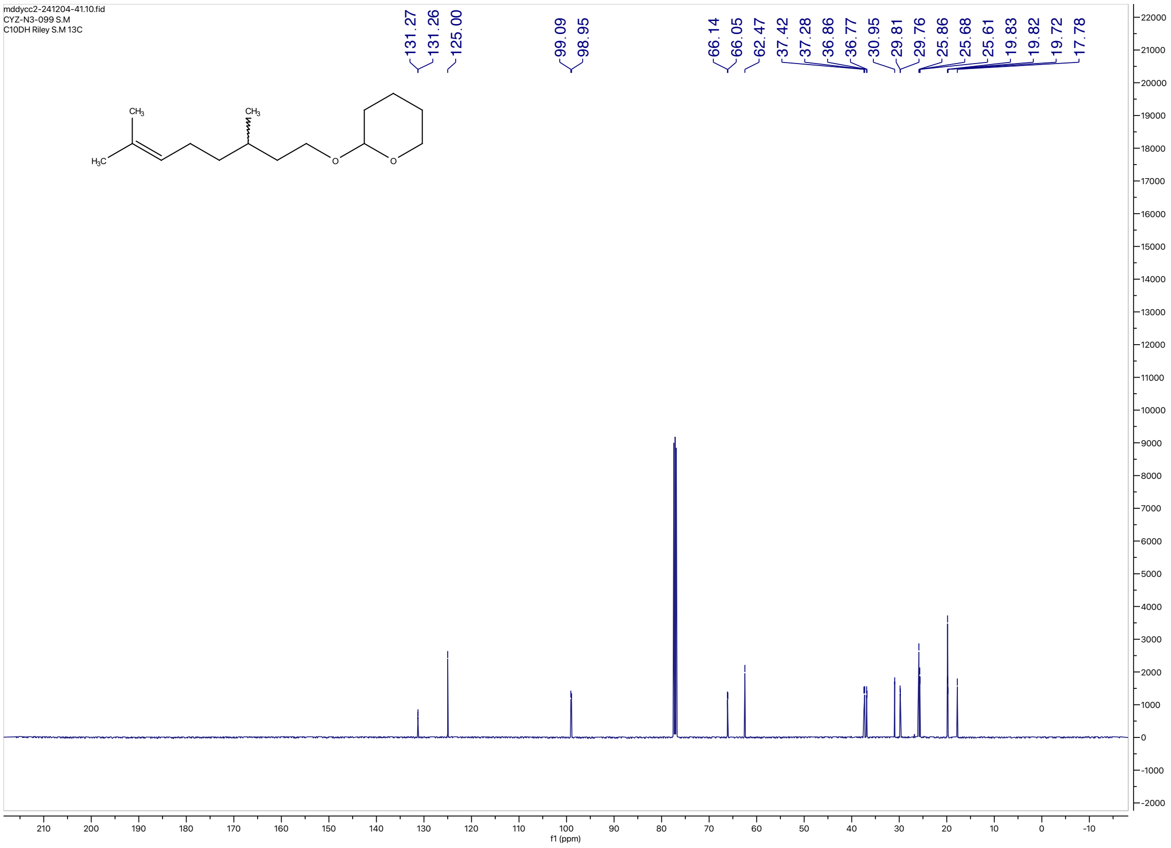

Compound **24** ^{13}C NMR in $CDCl_3$

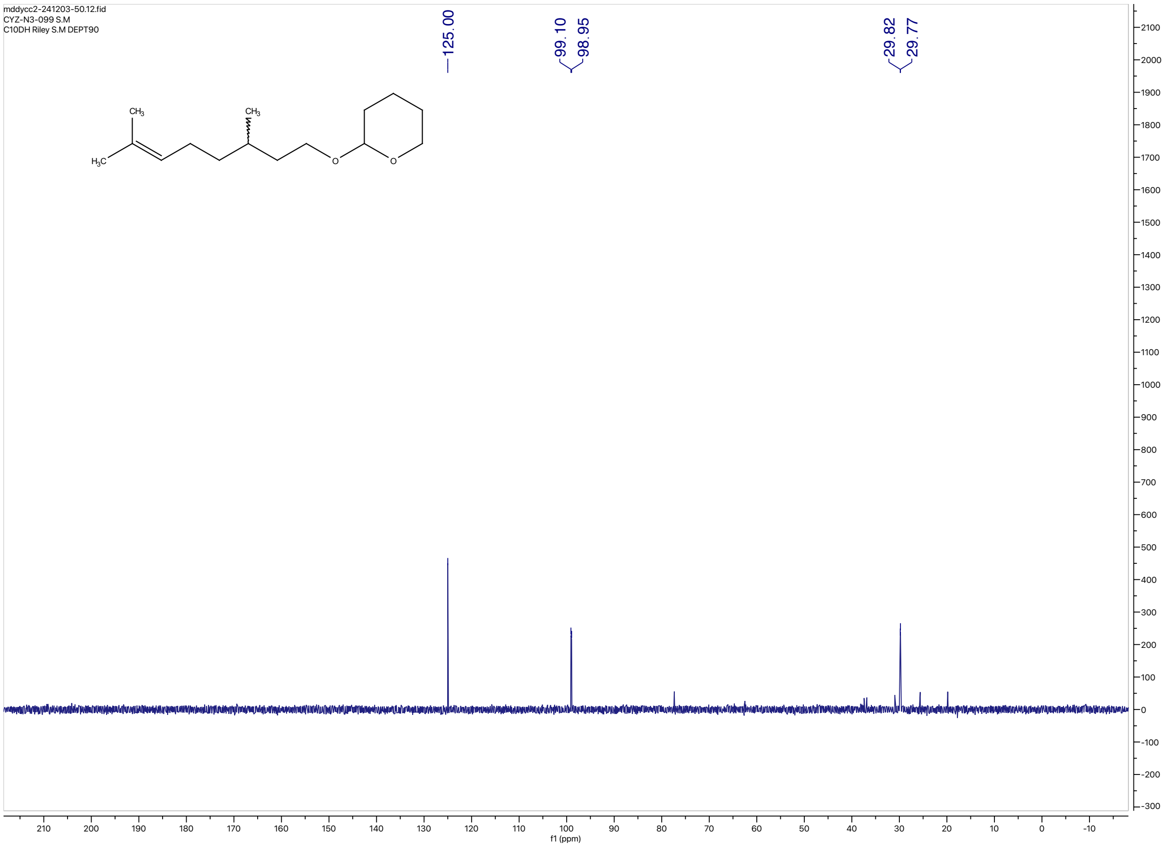

Compound **24** ^{13}C DEPT90 NMR in $CDCl_3$

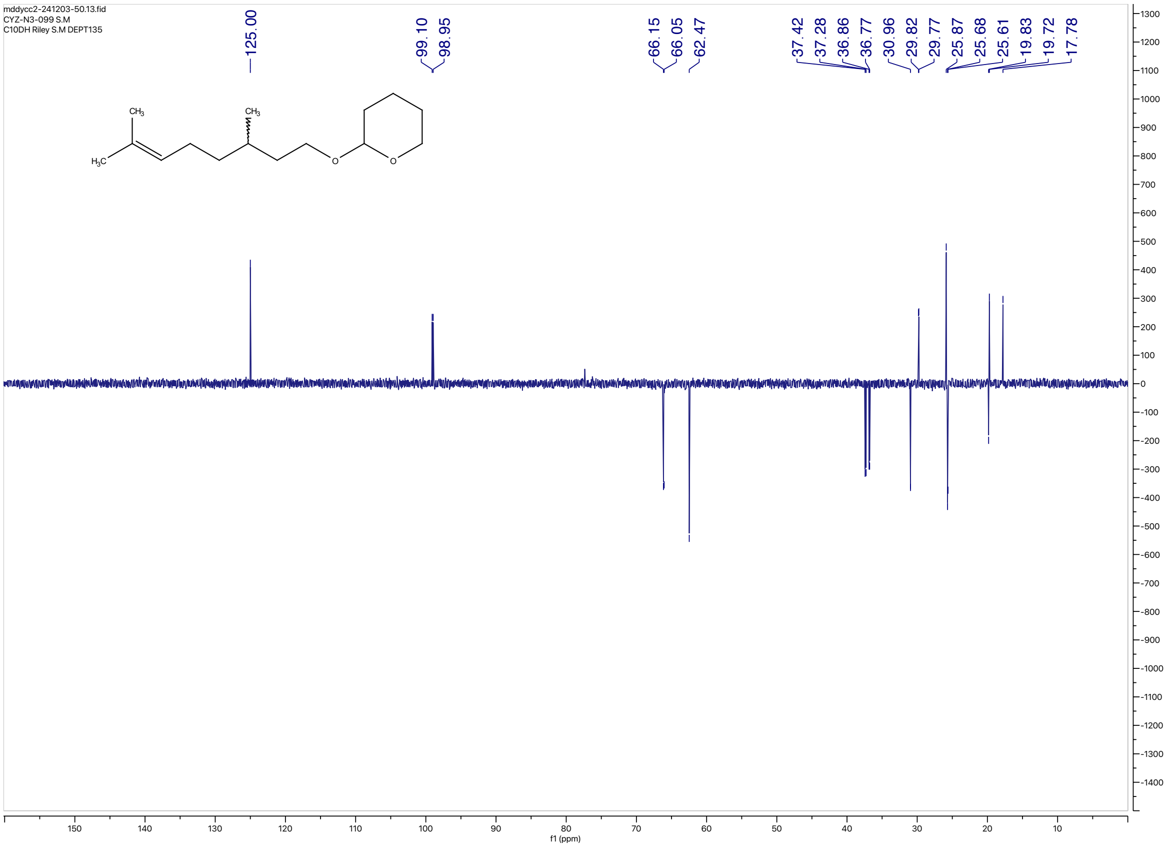

Compound **24** ^{13}C DEPT135 NMR in $CDCl_3$

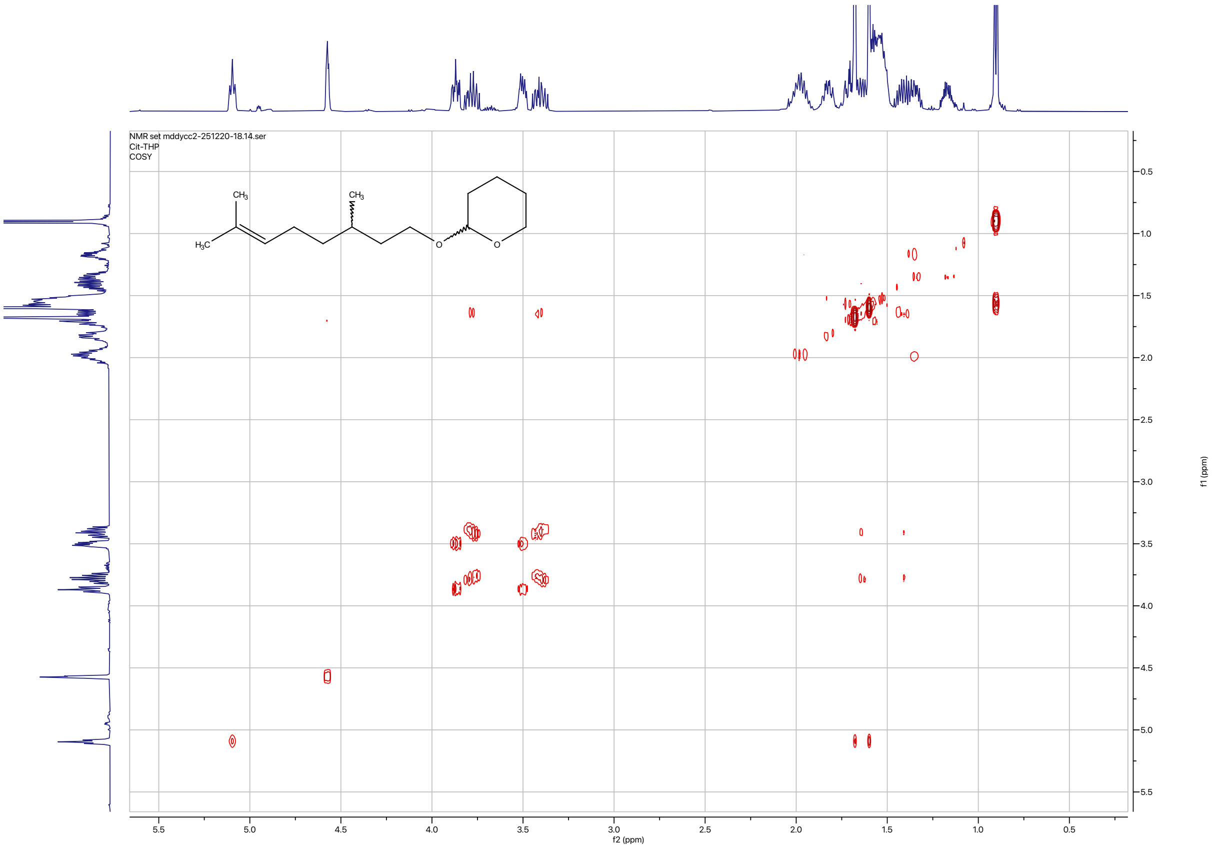

Compound **24** ^{1}H-^{1}H COSY NMR in $CDCl_3$

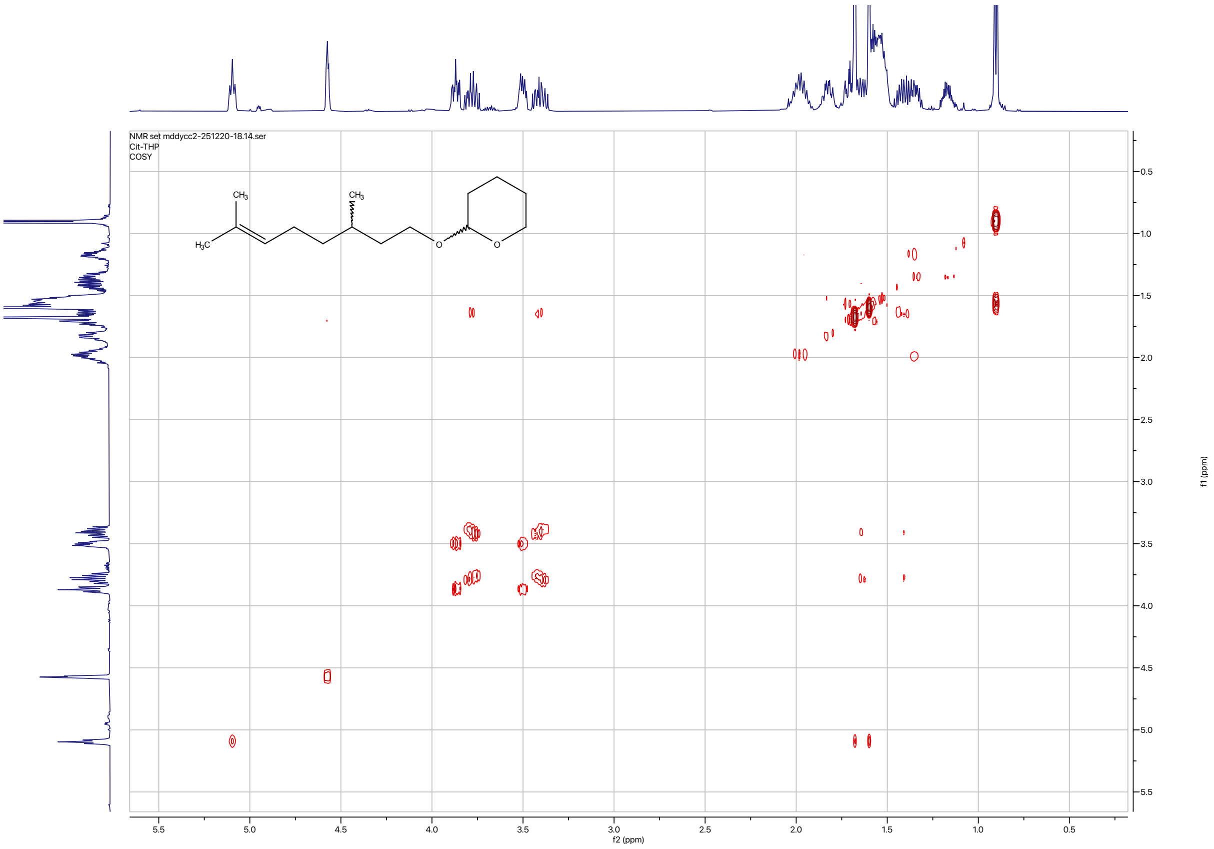

Compound **24** ^{1}H-^{13}C HSQC NMR in $CDCl_3$

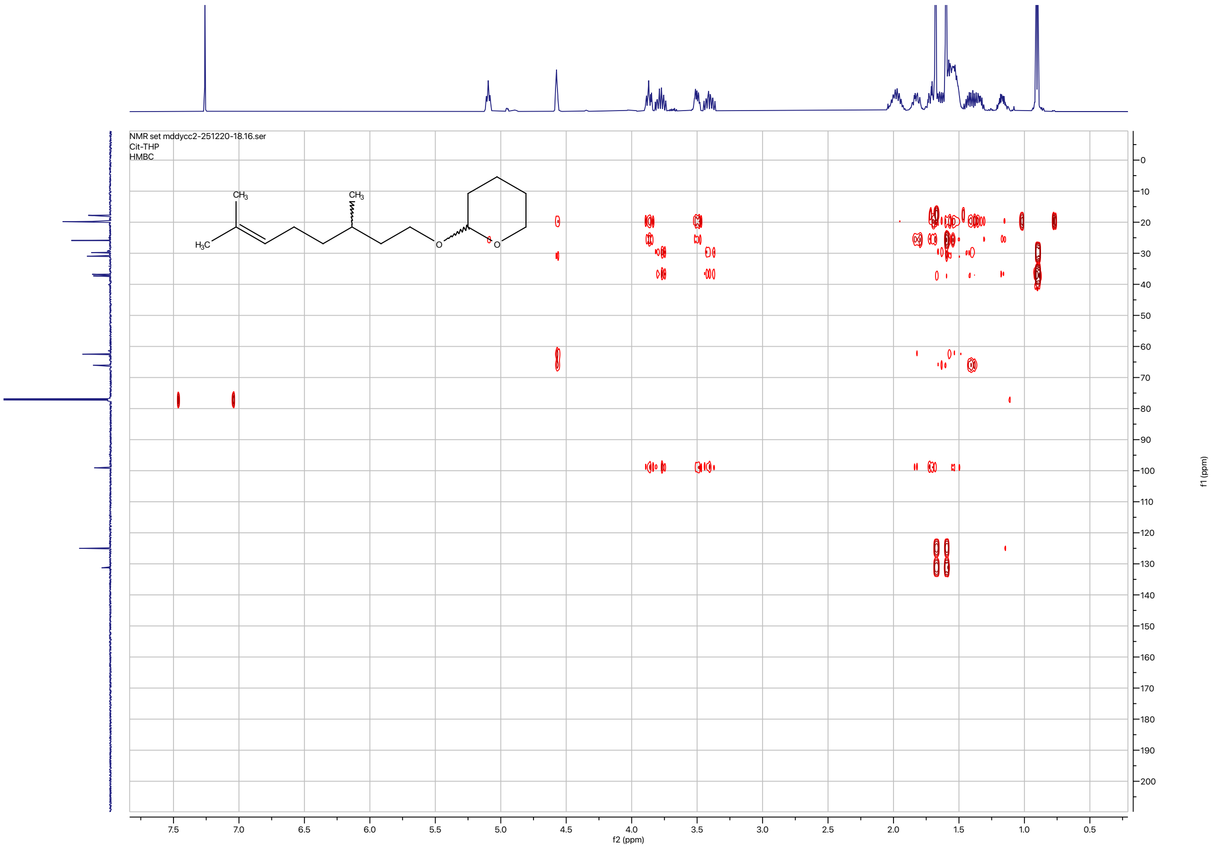

Compound **24** ^{1}H-^{13}C HMBC NMR in $CDCl_3$

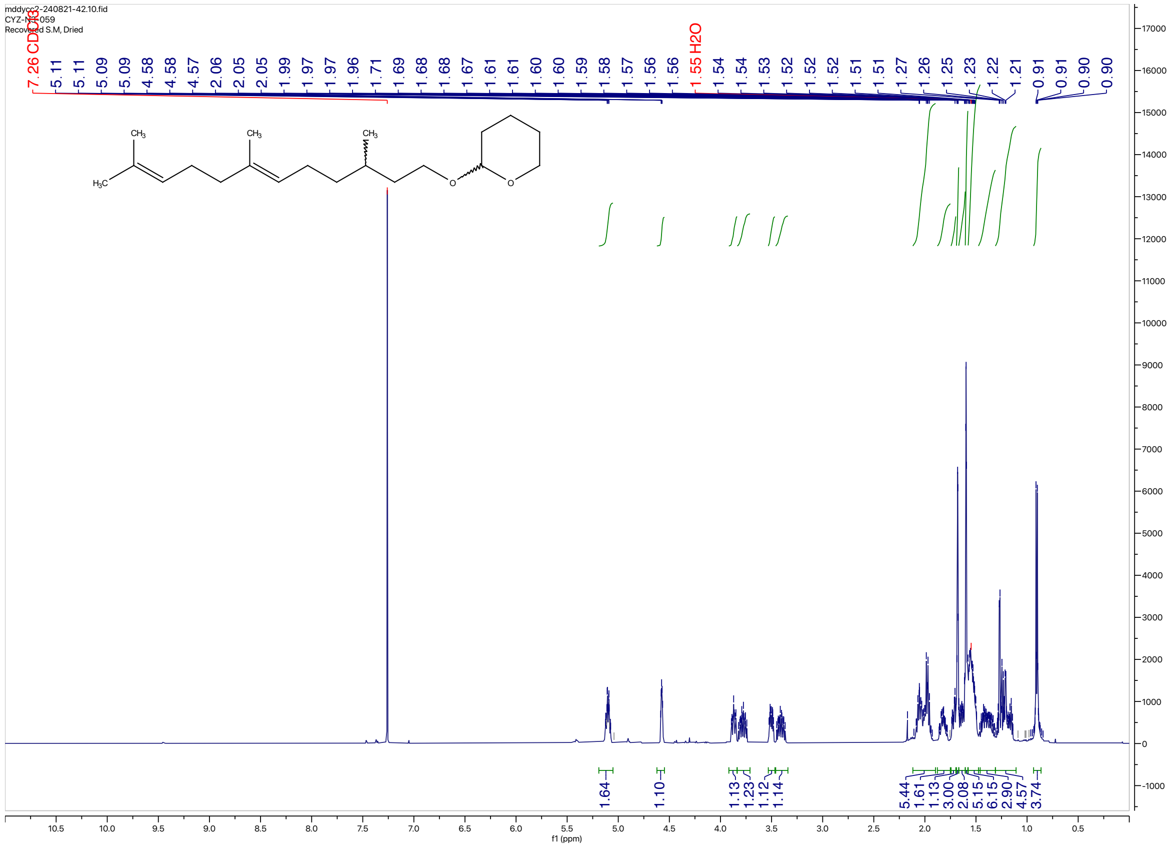

Compound **25** ^{1}H NMR in $CDCl_3$

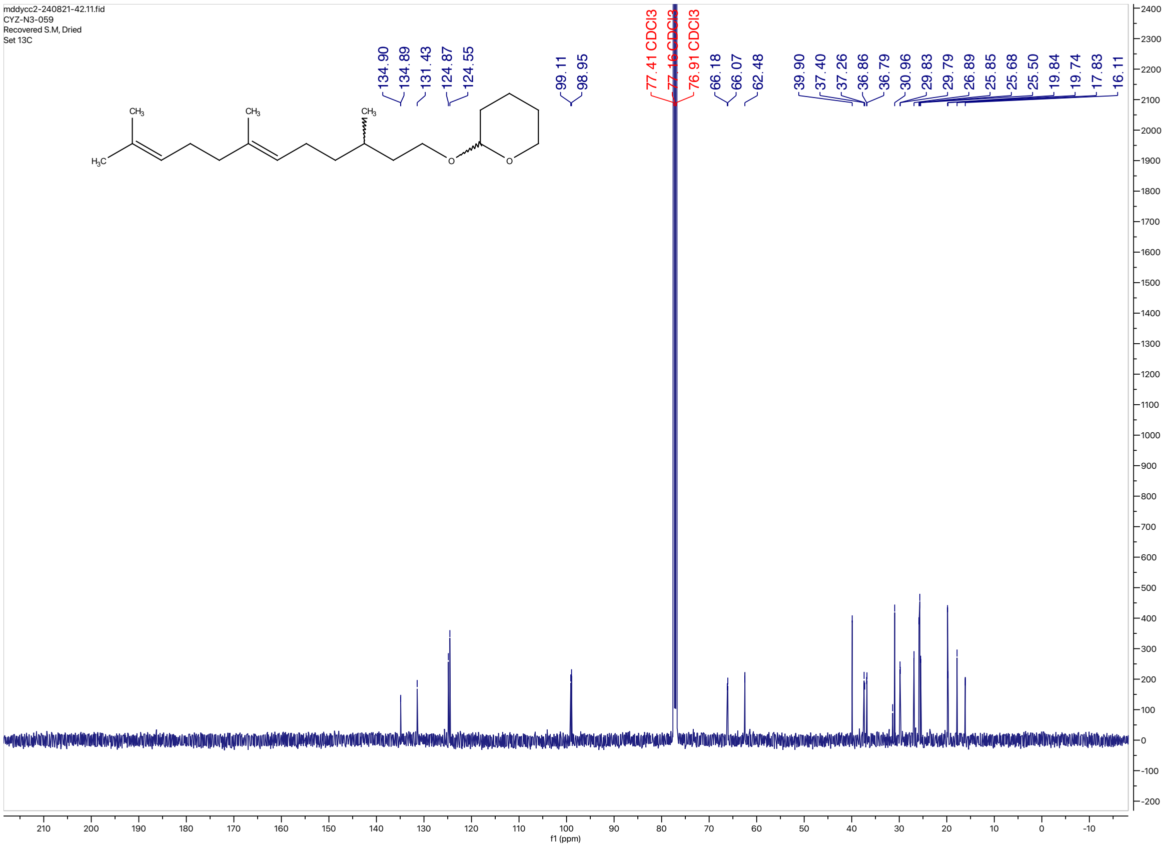

Compound **25** ^{13}C NMR in $CDCl_3$

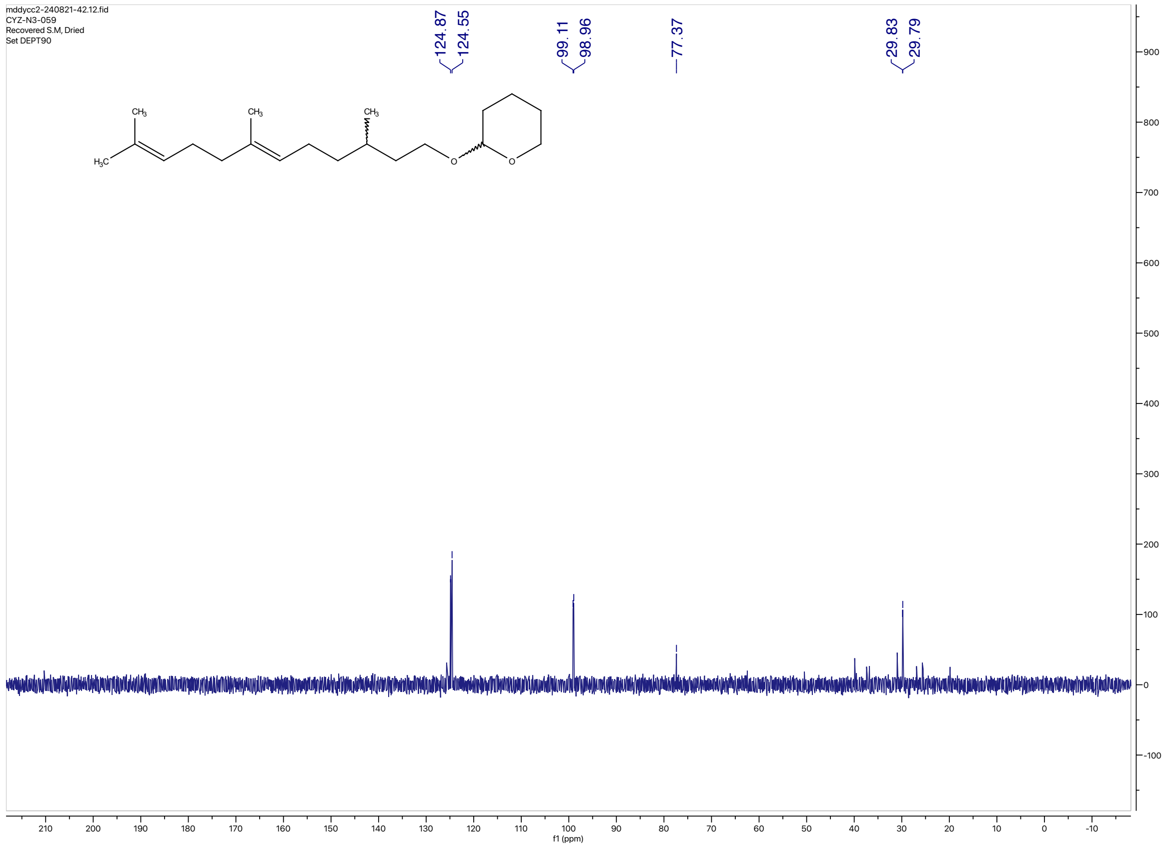

Compound **25** ^{13}C DEPT90 NMR in $CDCl_3$

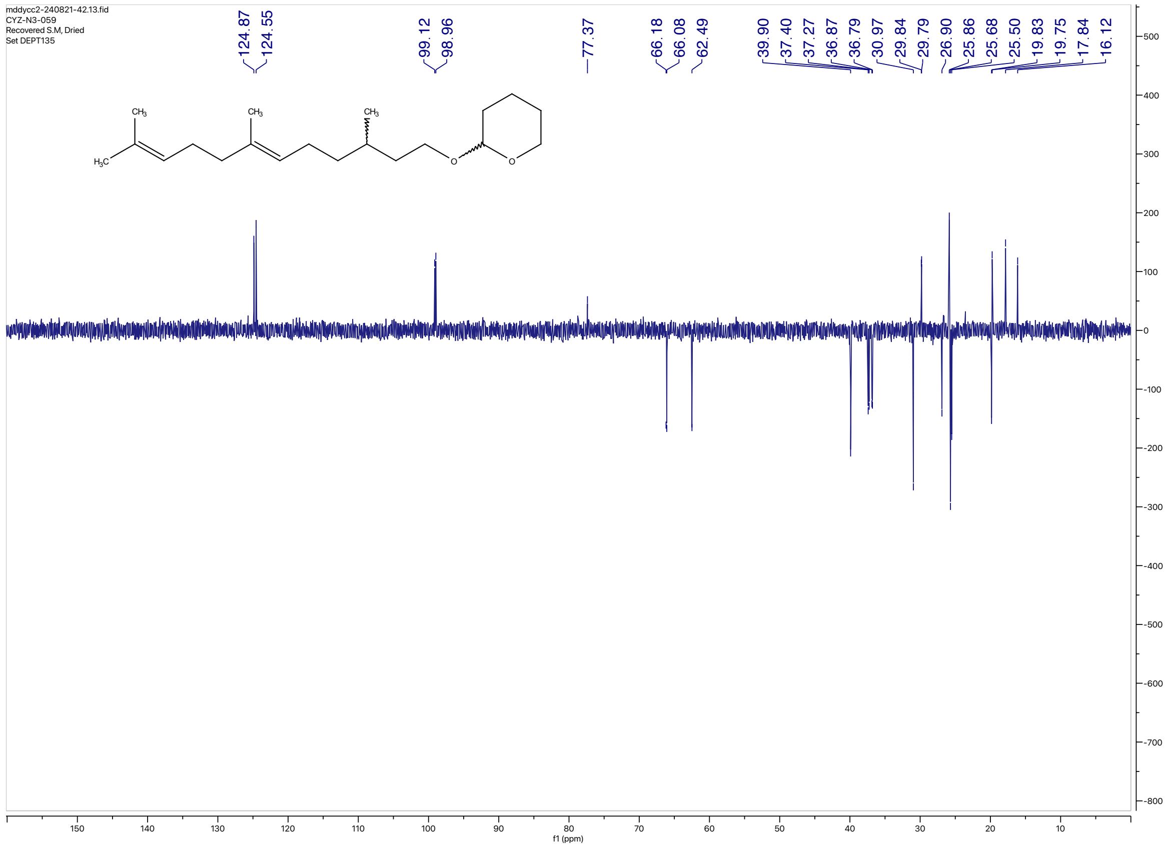

Compound **25** ^{13}C DEPT135 NMR in $CDCl_3$

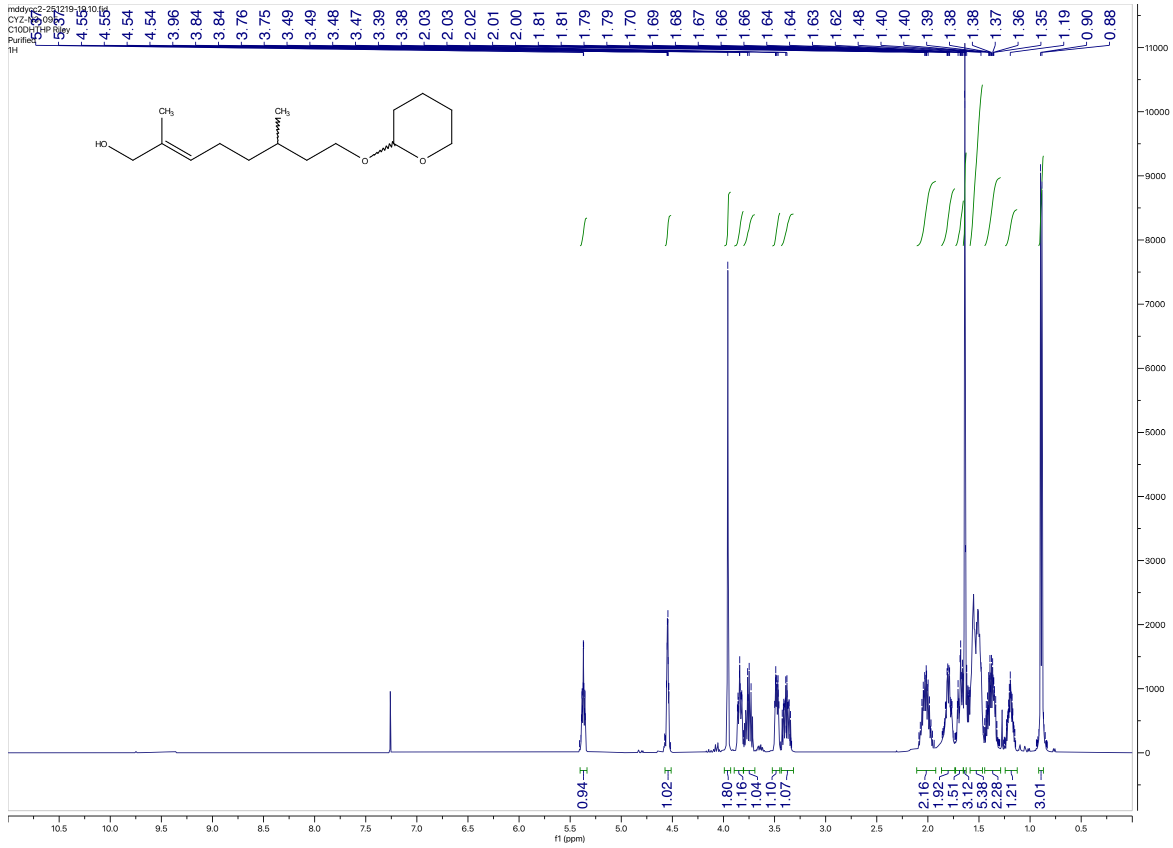

Compound **26** ^{1}H NMR in $CDCl_3$

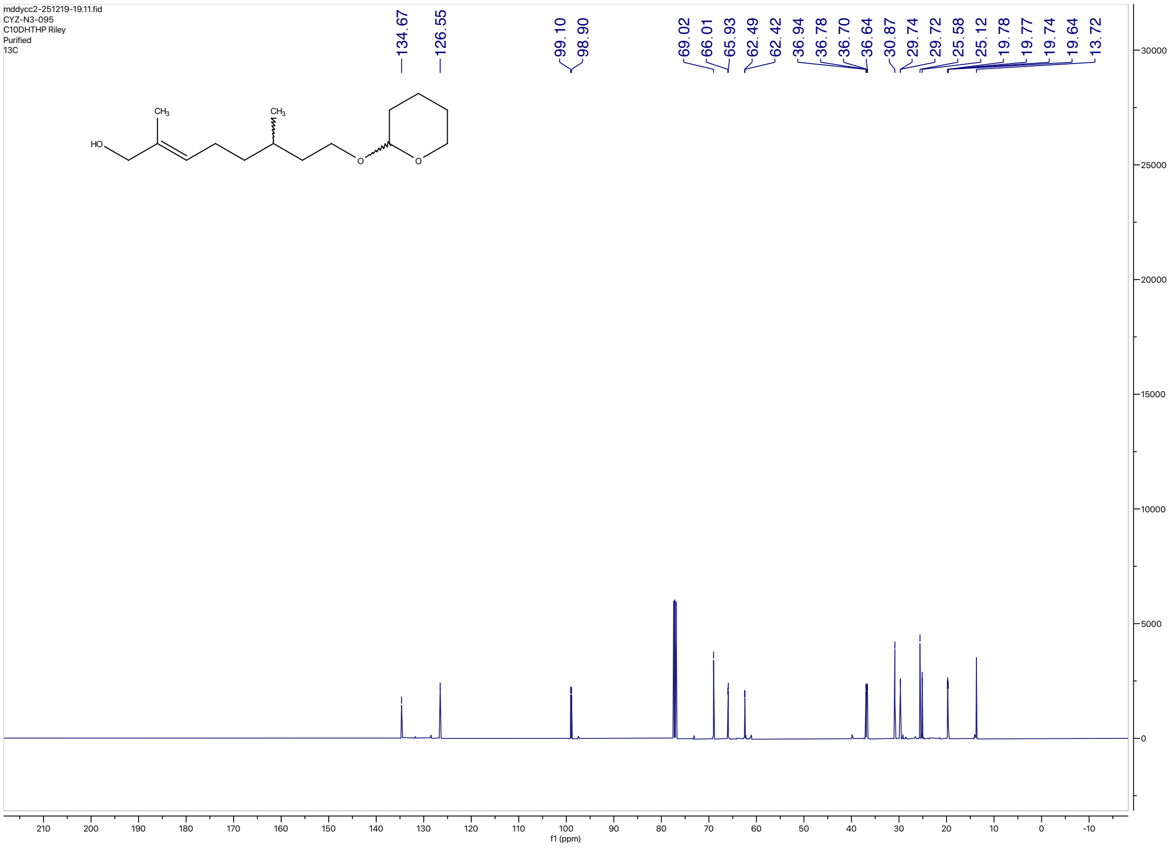

Compound **26** ^{13}C NMR in $CDCl_3$

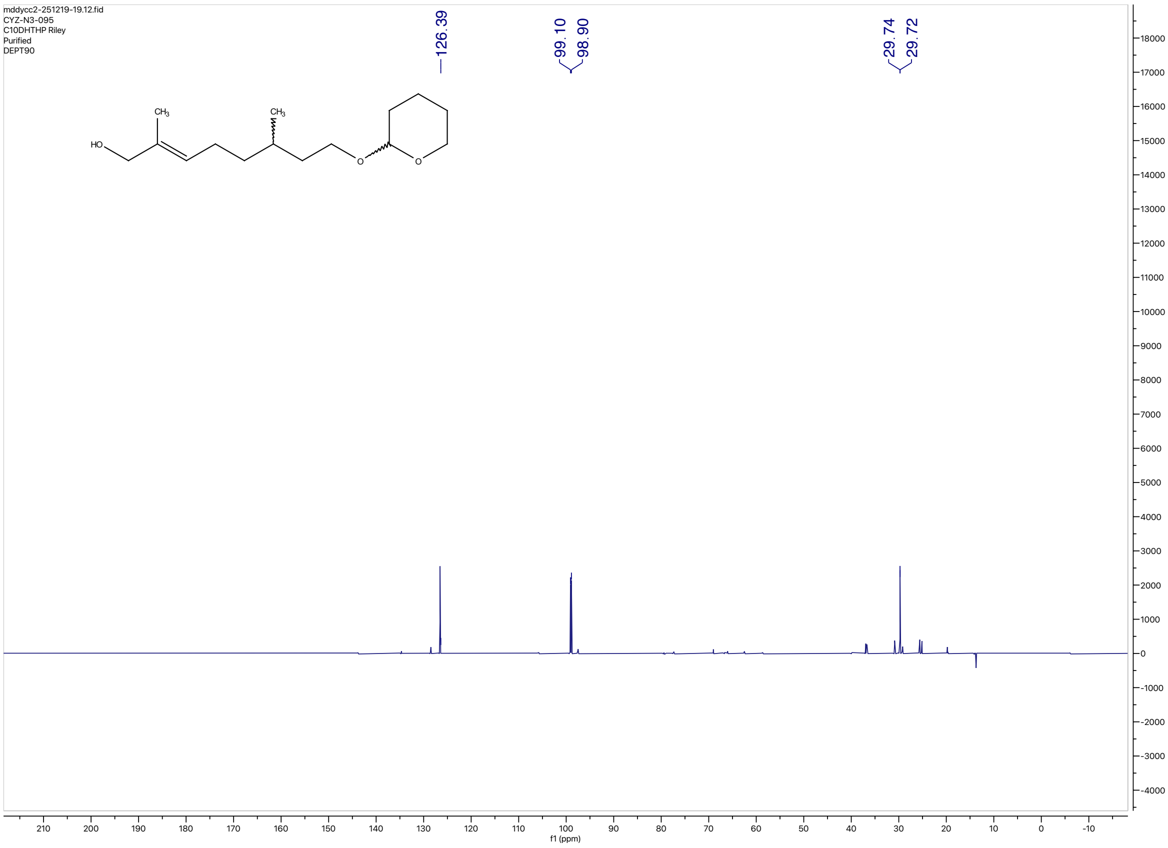

Compound **26** ^{13}C DEPT90 NMR in $CDCl_3$

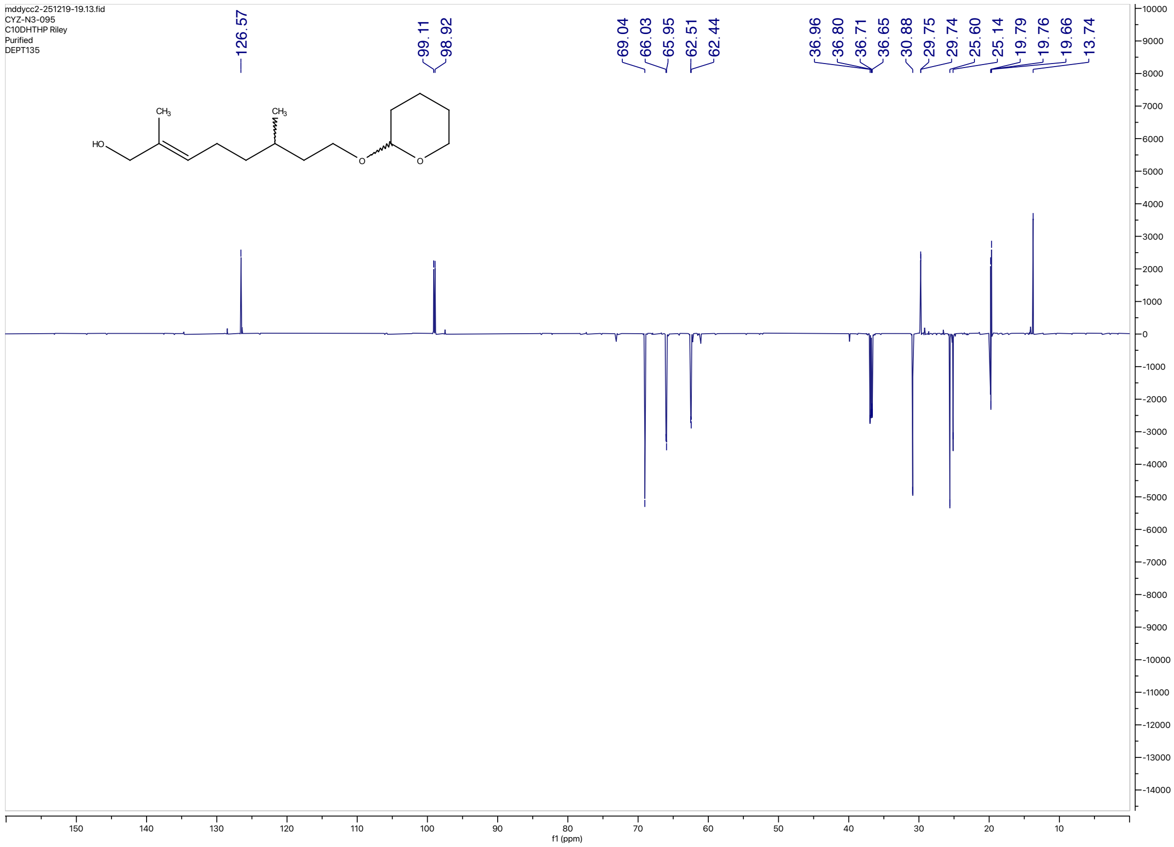

Compound **26** ^{13}C DEPT135 NMR in $CDCl_3$

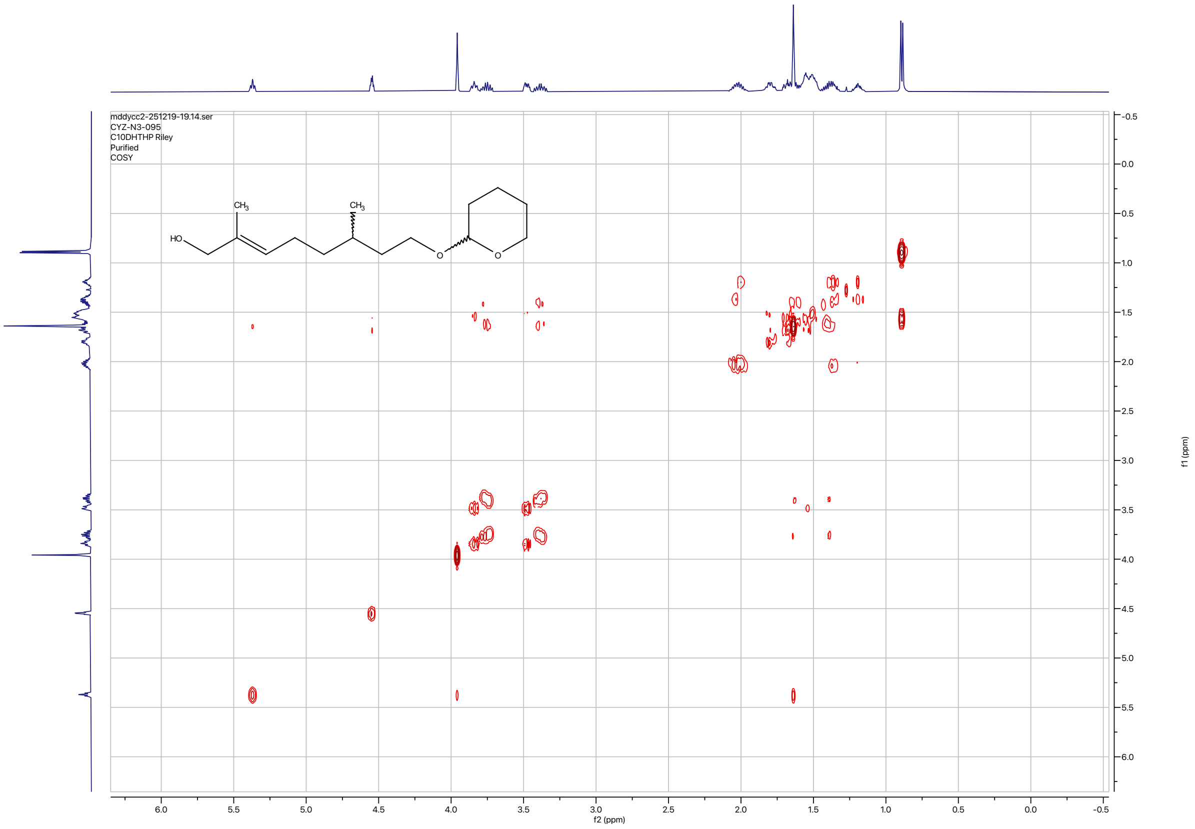

Compound **26** ^{1}H-^{1}H COSY NMR in $CDCl_3$

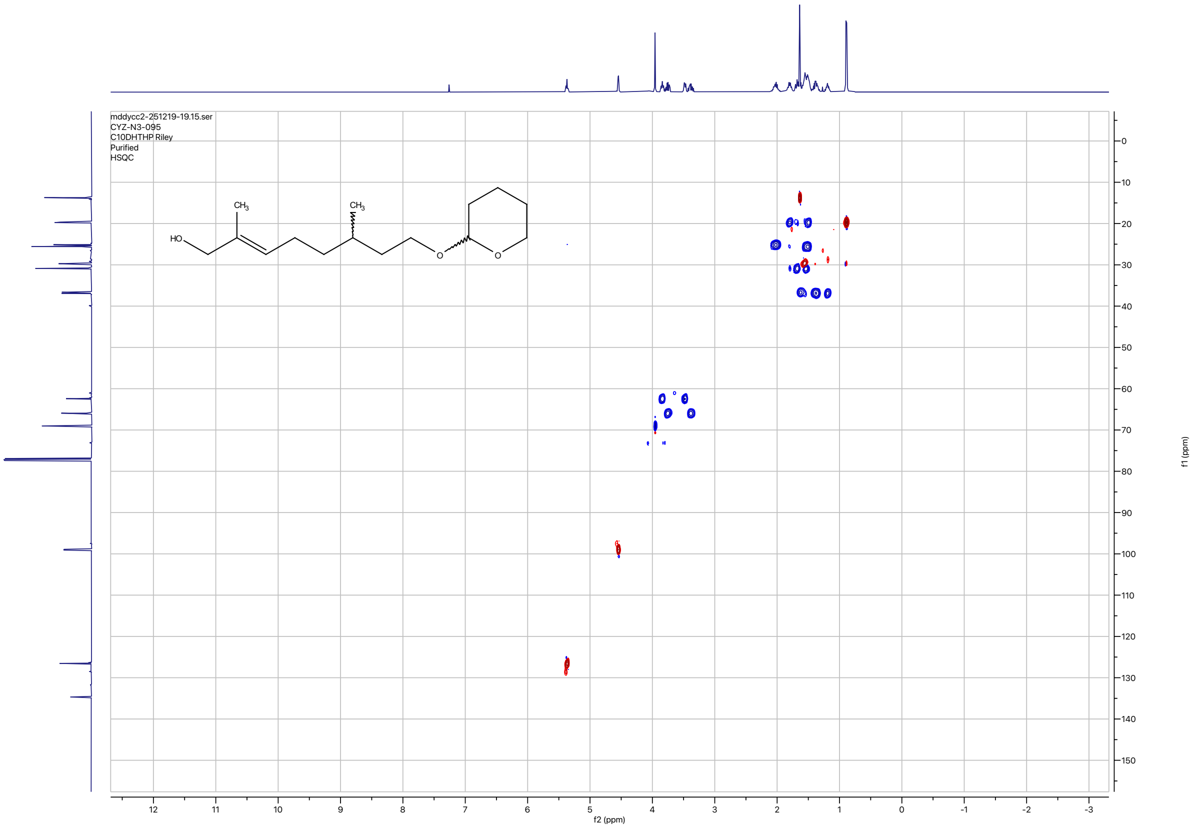

Compound **26** ^{1}H-^{13}C HSQC NMR in $CDCl_3$

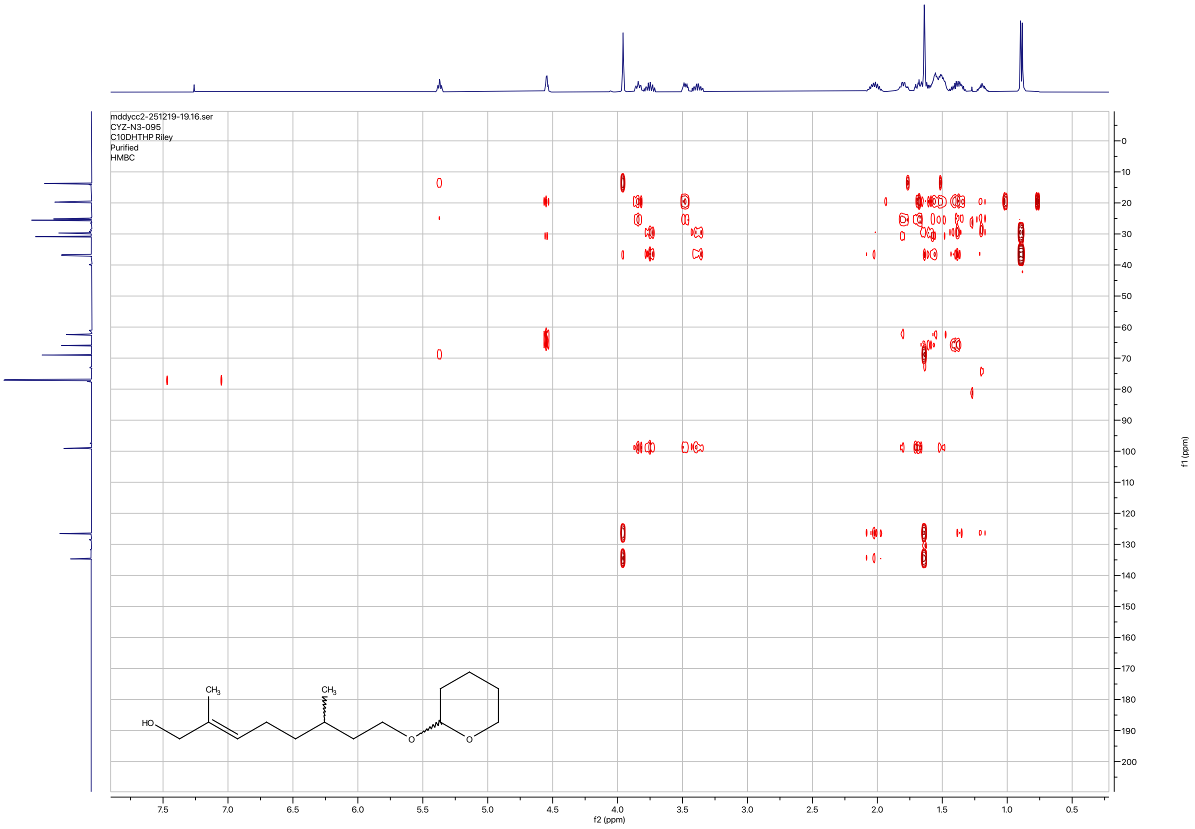

Compound **26** ^{1}H-^{13}C HMBC NMR in $CDCl_3$

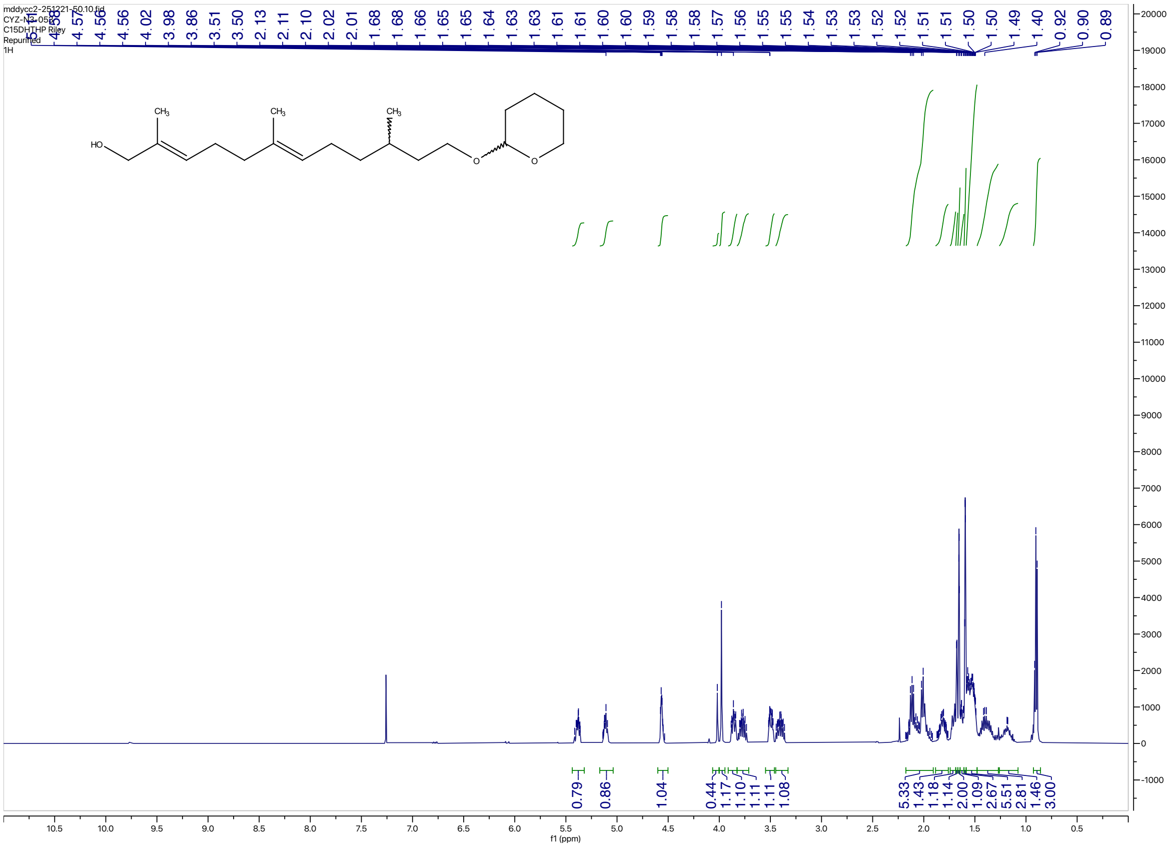

Compound **27** ^{1}H NMR in $CDCl_3$

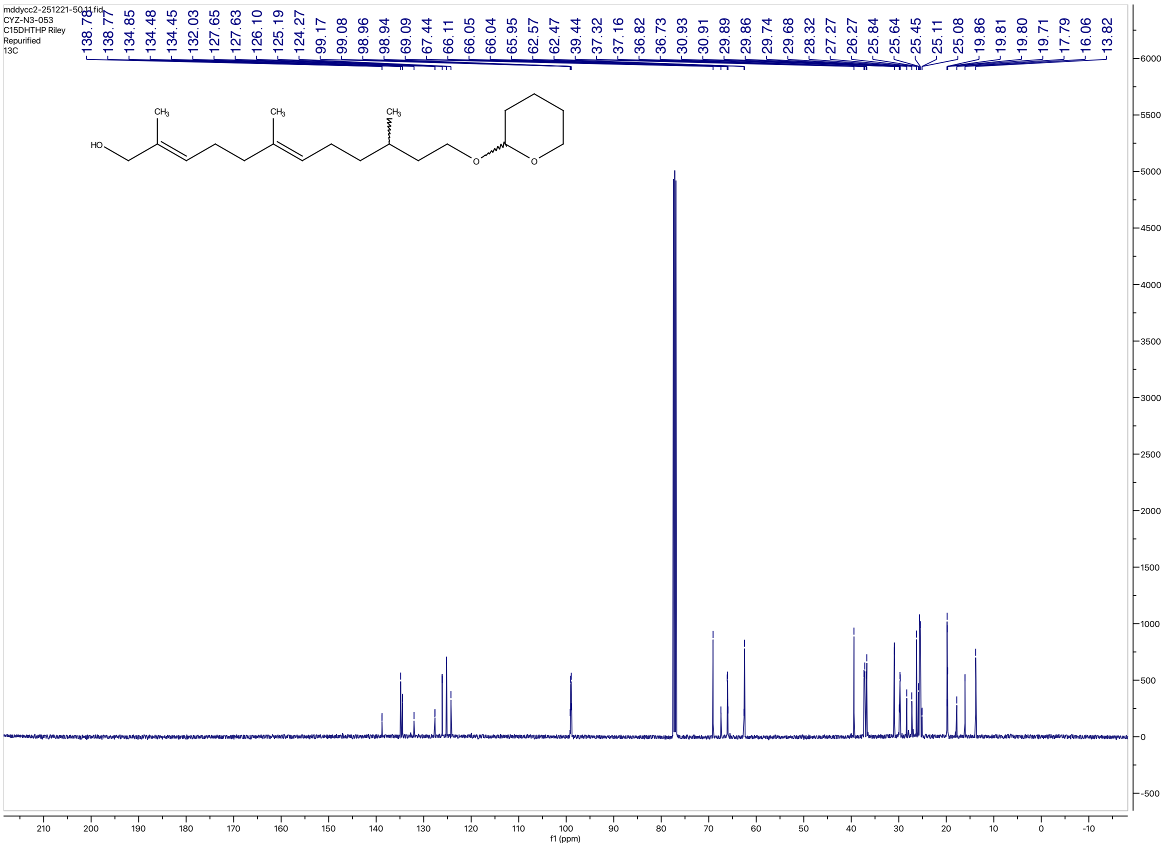

Compound **27** ^{13}C NMR in $CDCl_3$

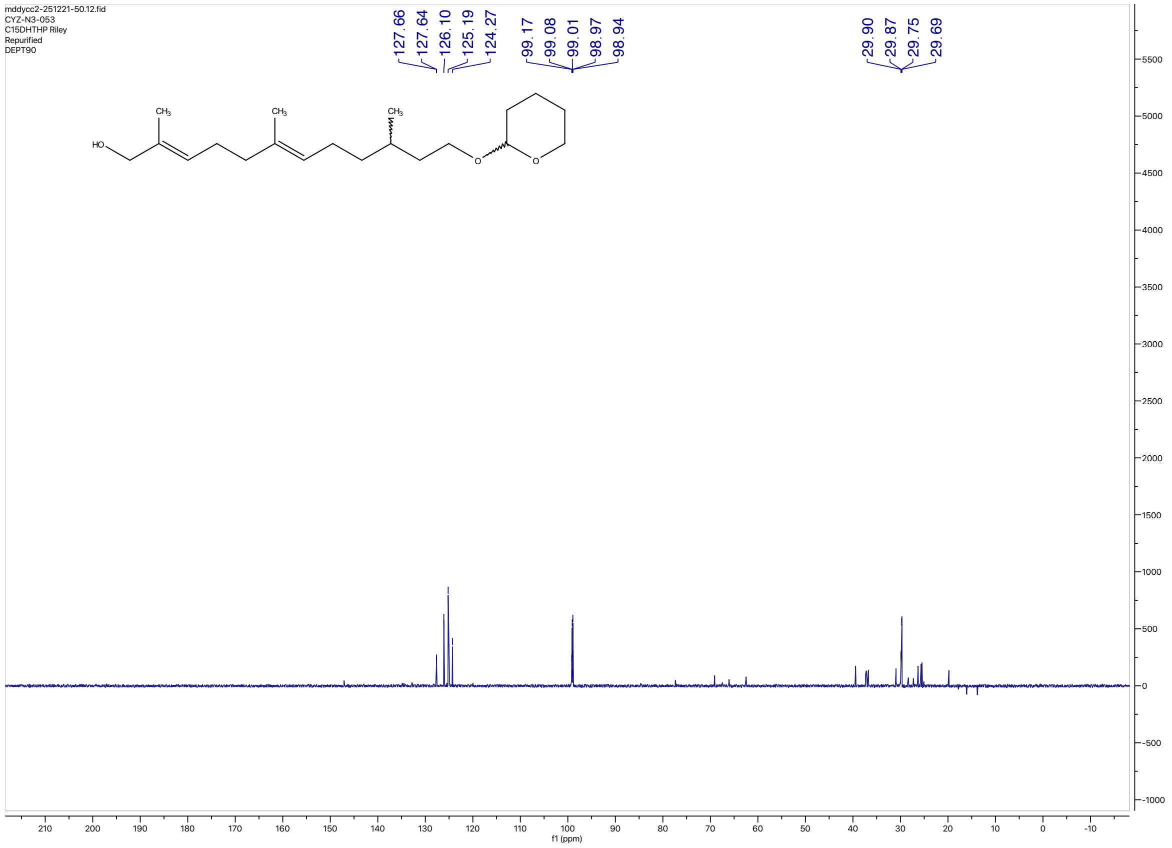

Compound **27** ^{13}C DEPT90 NMR in $CDCl_3$

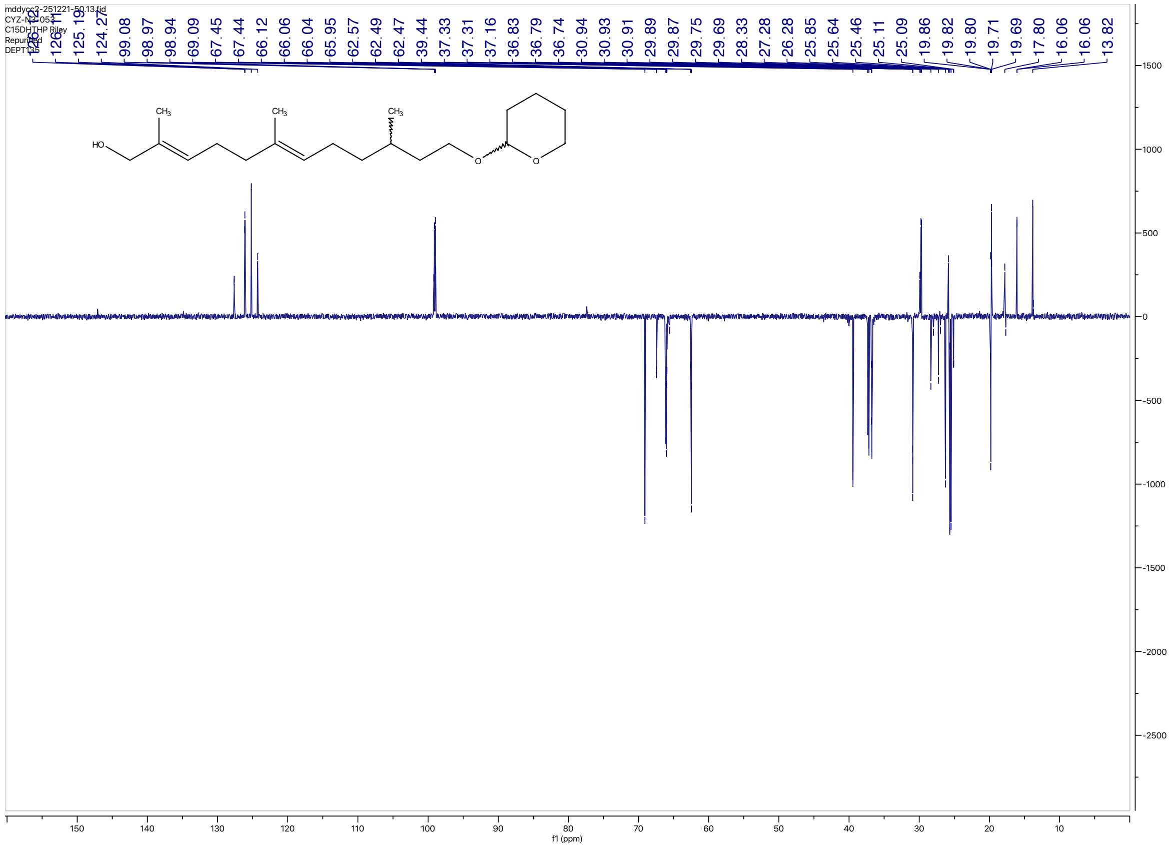

Compound **27** ^{13}C DEPT135 NMR in $CDCl_3$

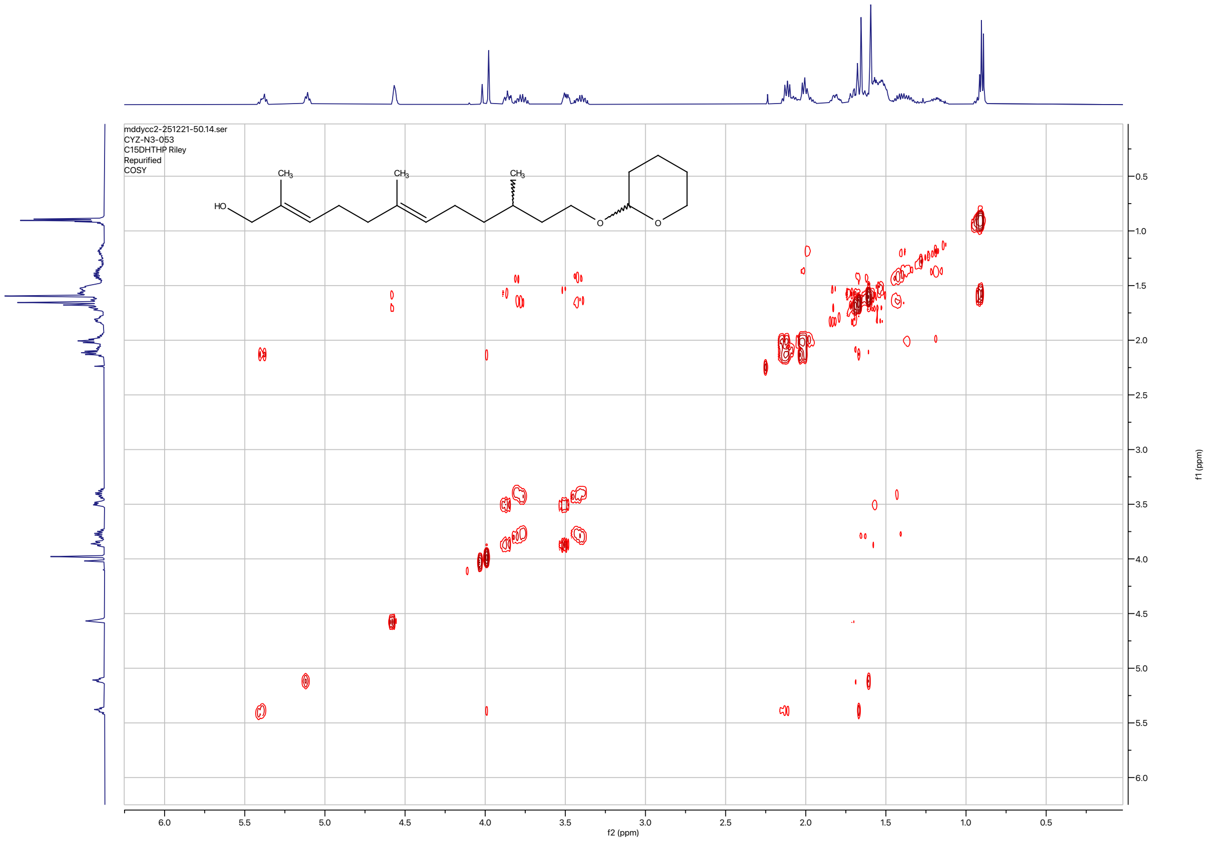

Compound **27** ^{1}H-^{1}H COSY NMR in $CDCl_3$

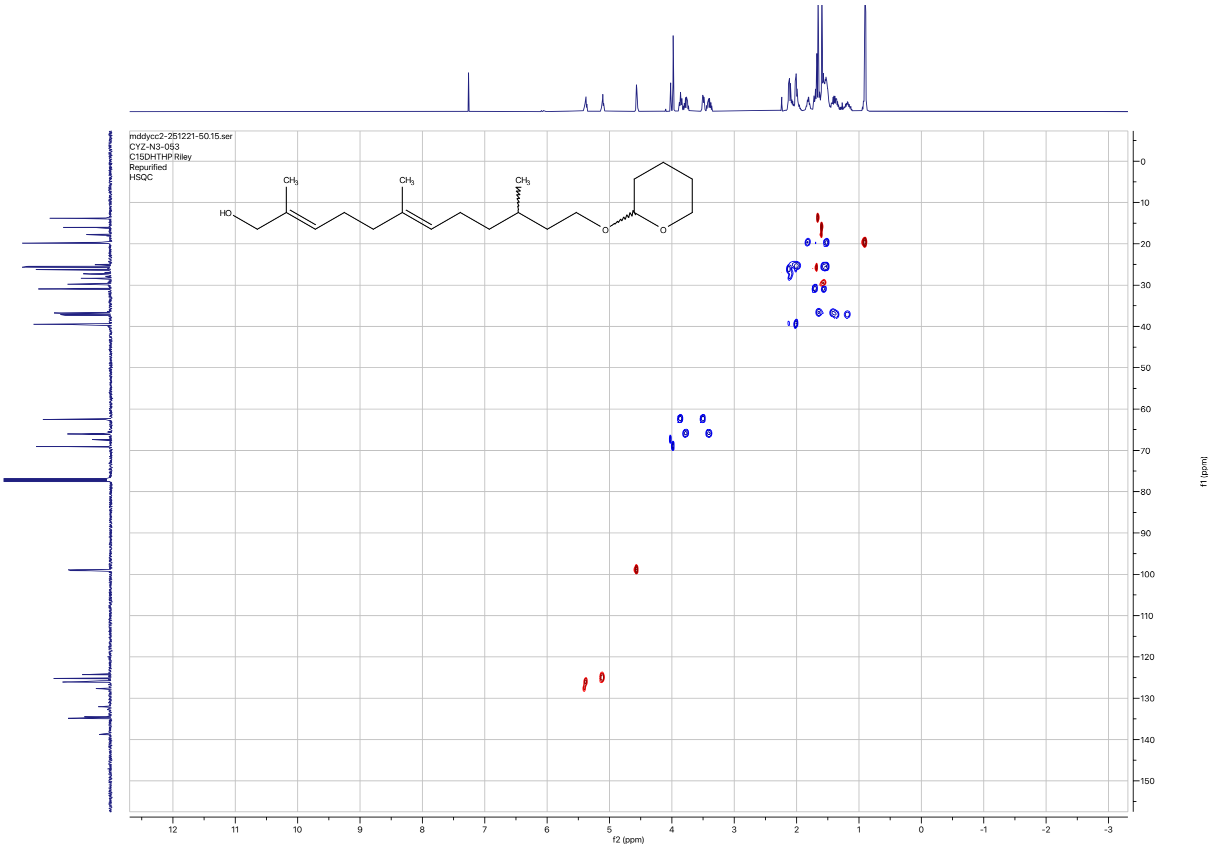

Compound **27** ^{1}H-^{13}C HSQC NMR in $CDCl_3$

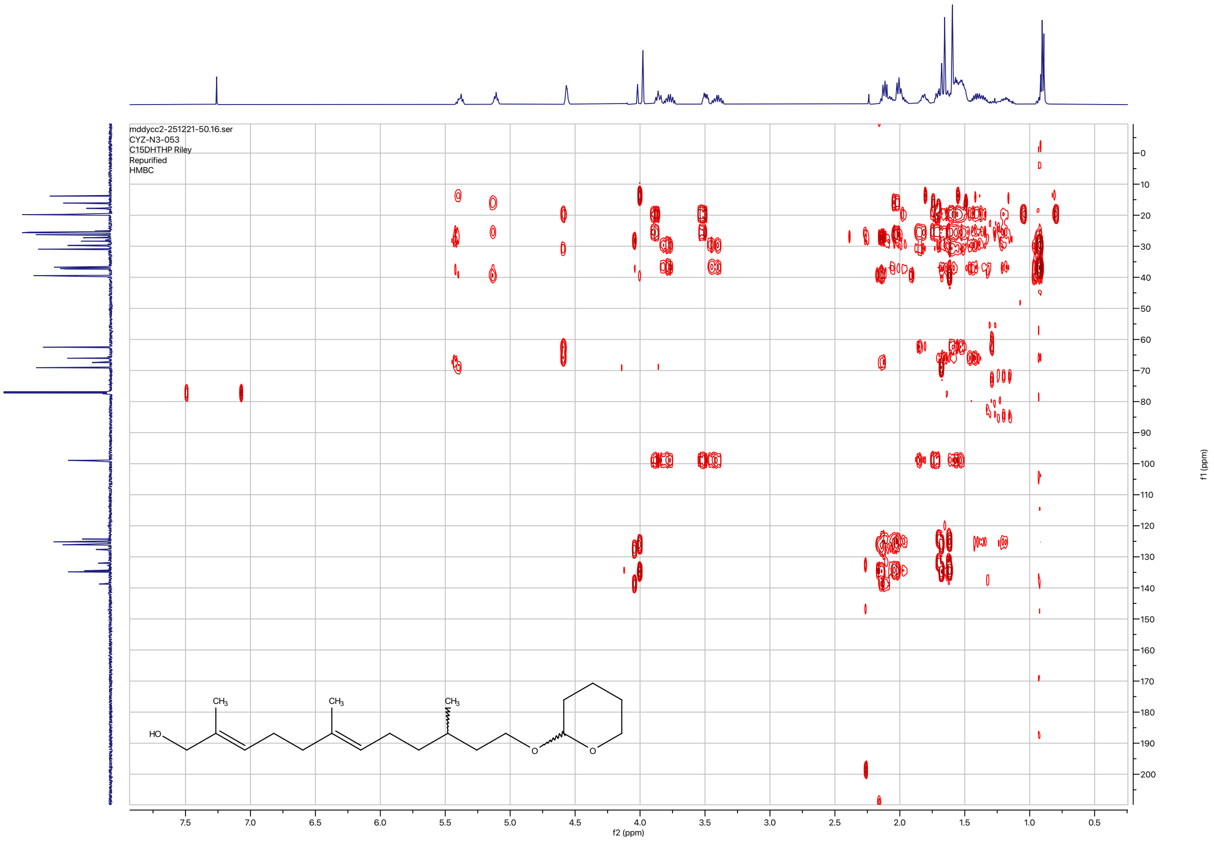

Compound **27** ^{1}H-^{13}C HMBC NMR in $CDCl_3$

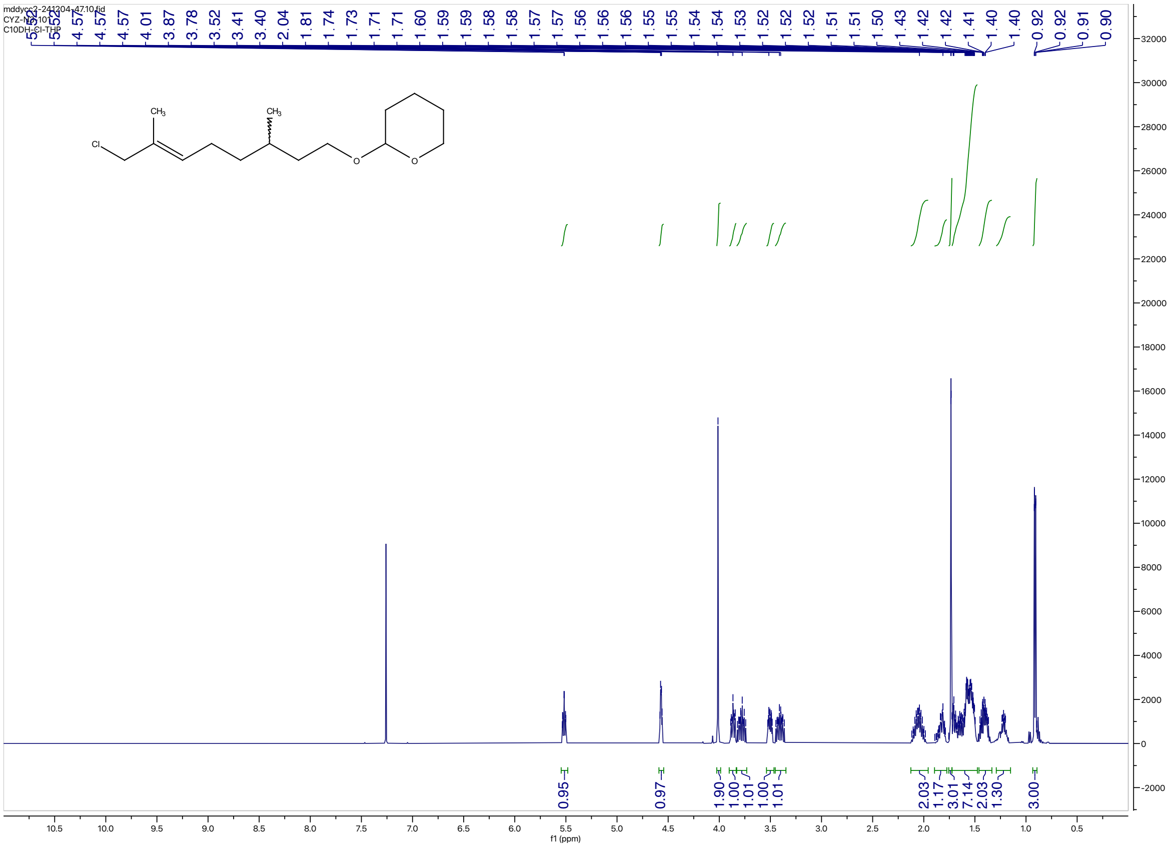

Compound **28** ^{1}H NMR in $CDCl_3$

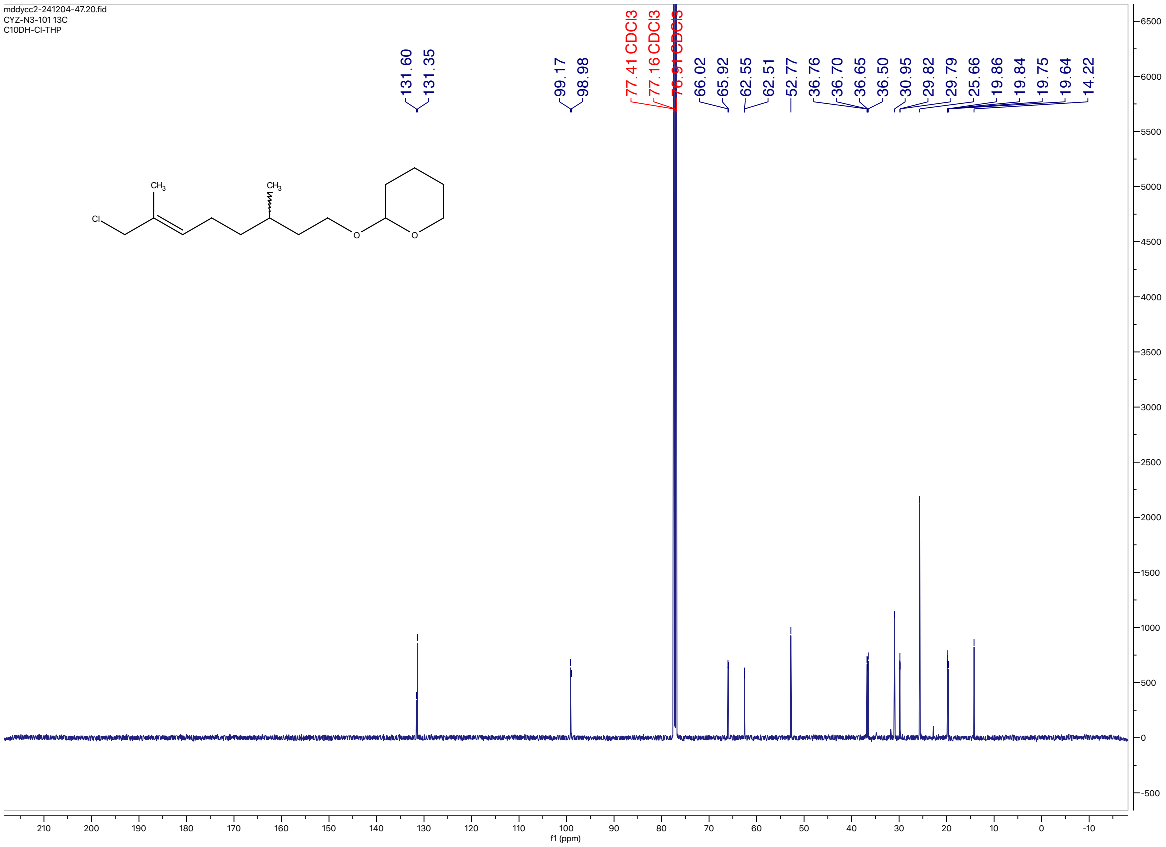

Compound **28** ^{13}C NMR in $CDCl_3$

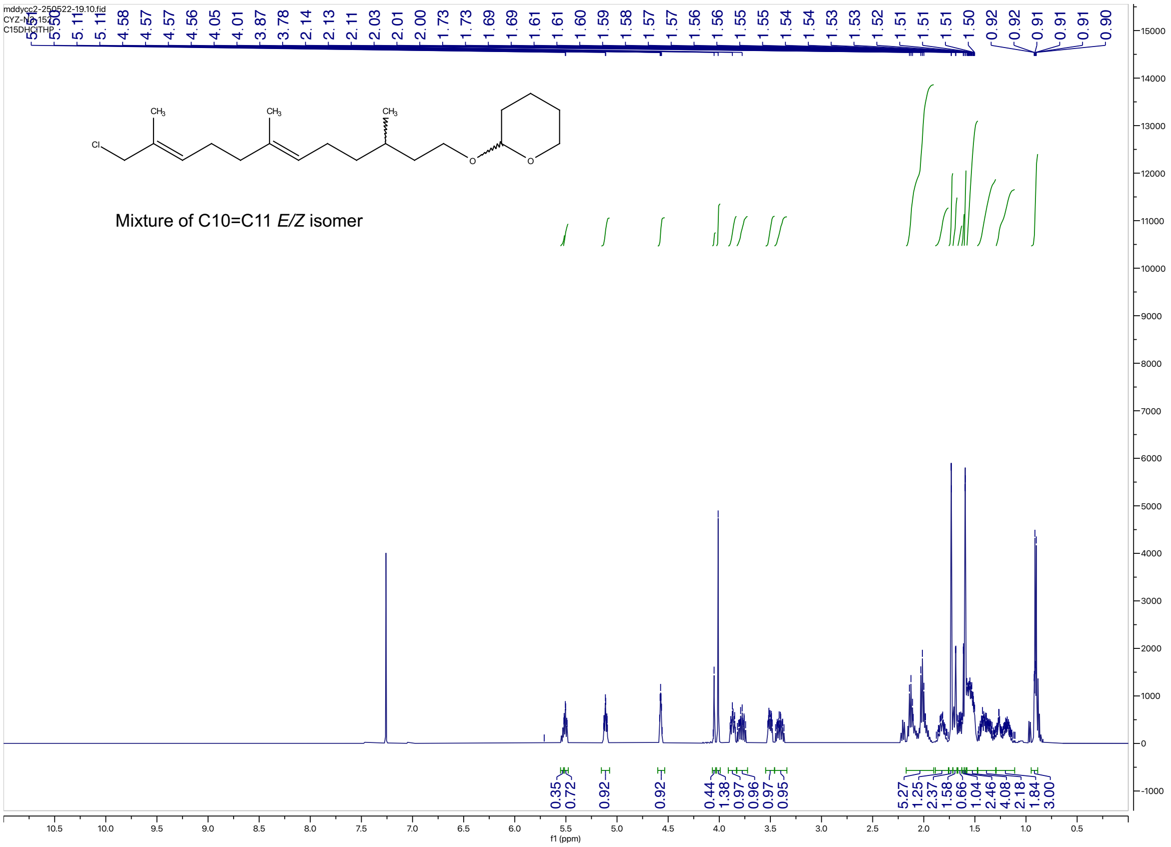

Compound **29** ^{1}H NMR in $CDCl_3$

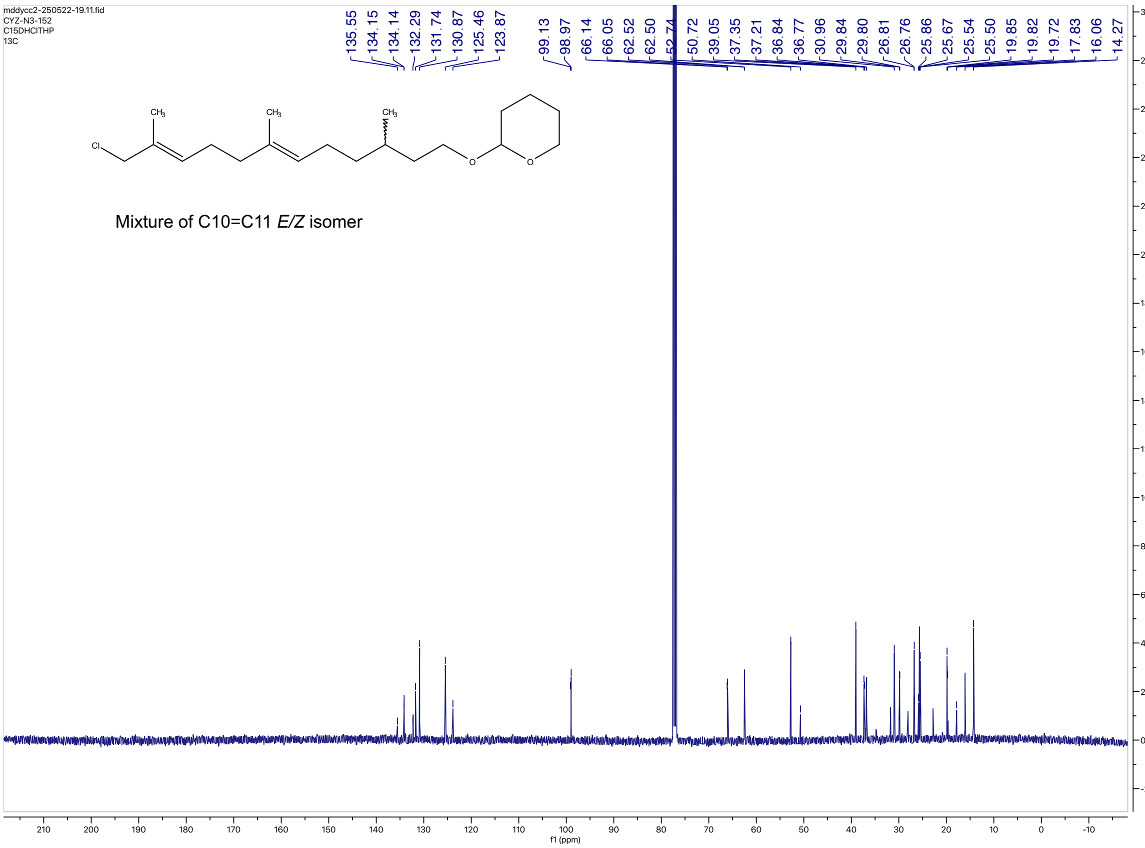

Compound **29** ^{13}C NMR in $CDCl_3$

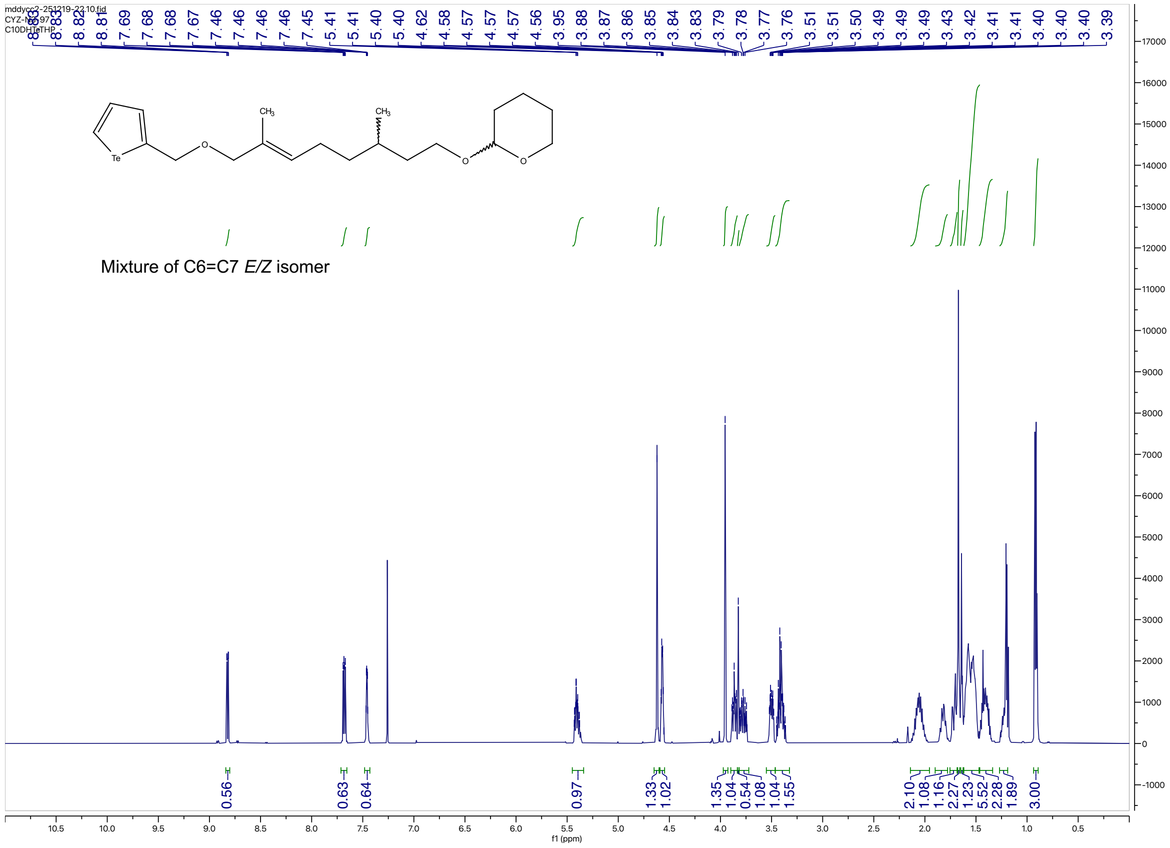

Compound **30** ^{1}H NMR in $CDCl_3$

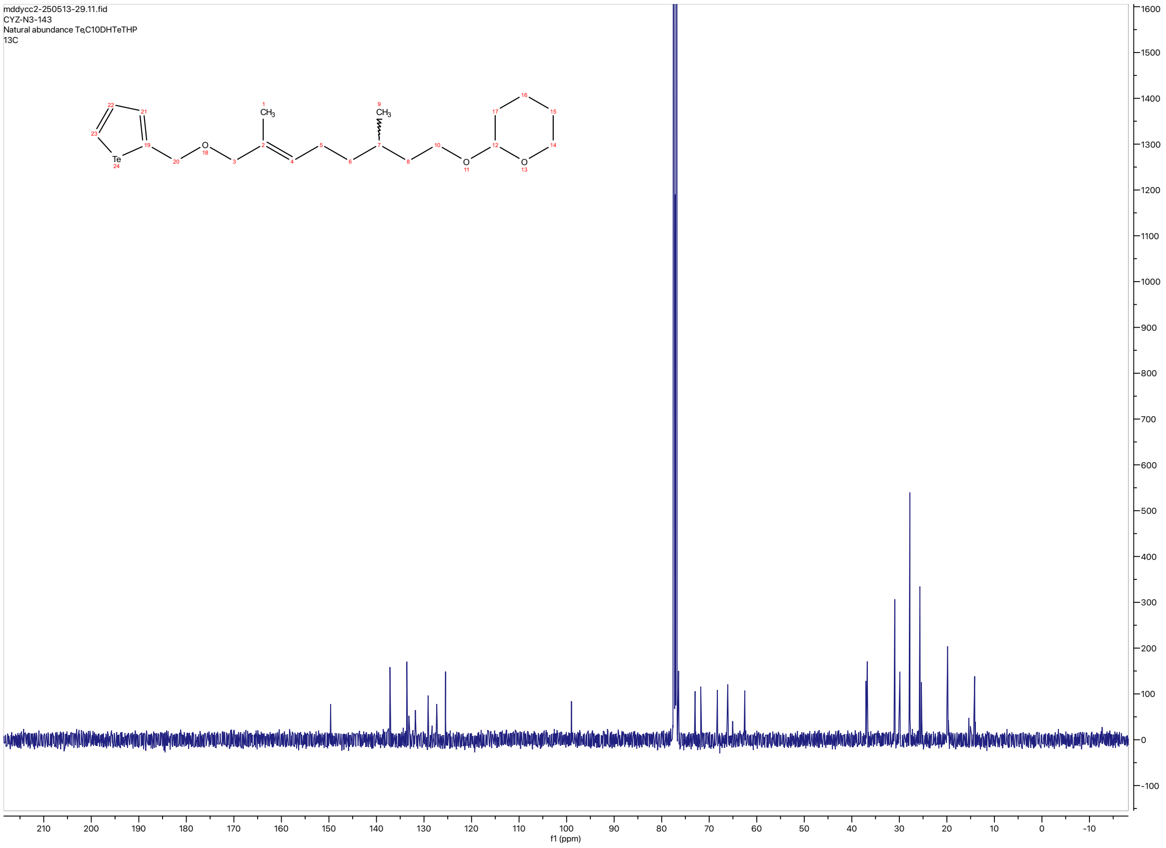

Compound **30** ^{13}C NMR in $CDCl_3$

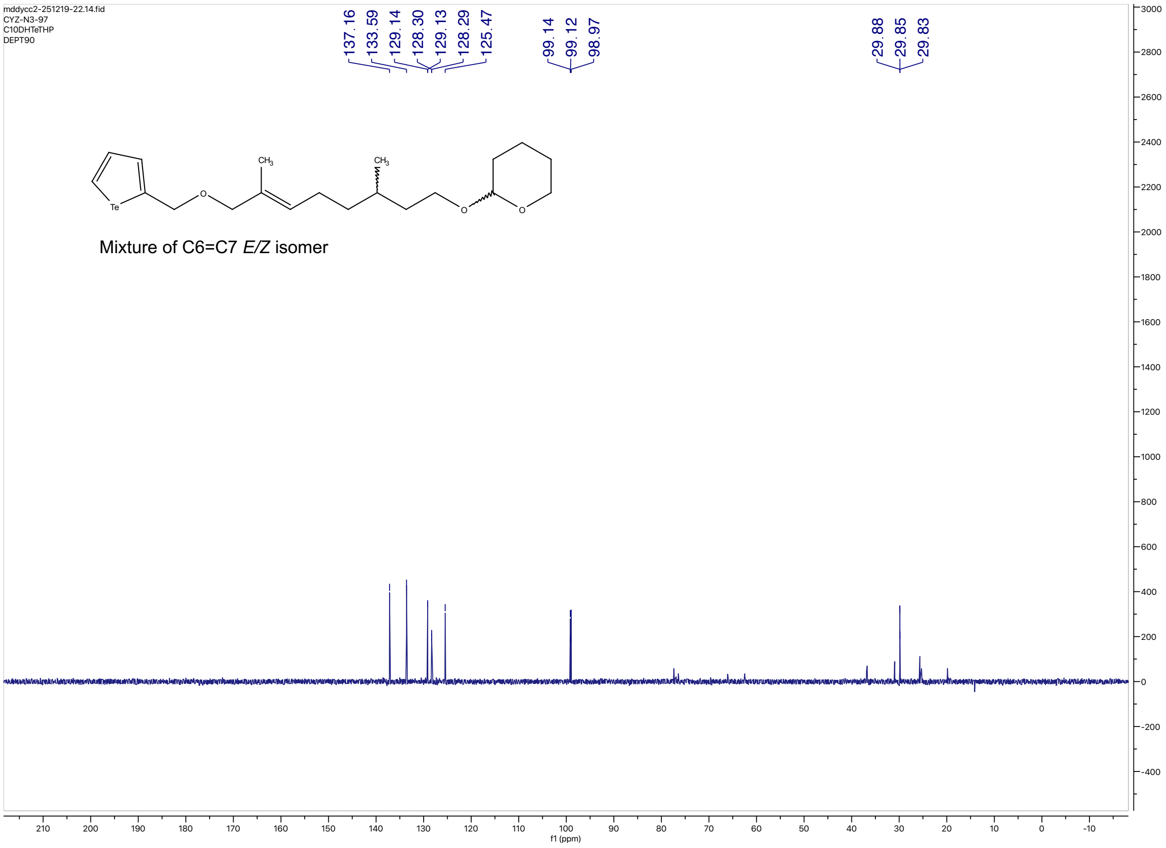

Compound **30** ^{13}C DEPT90 NMR in $CDCl_3$

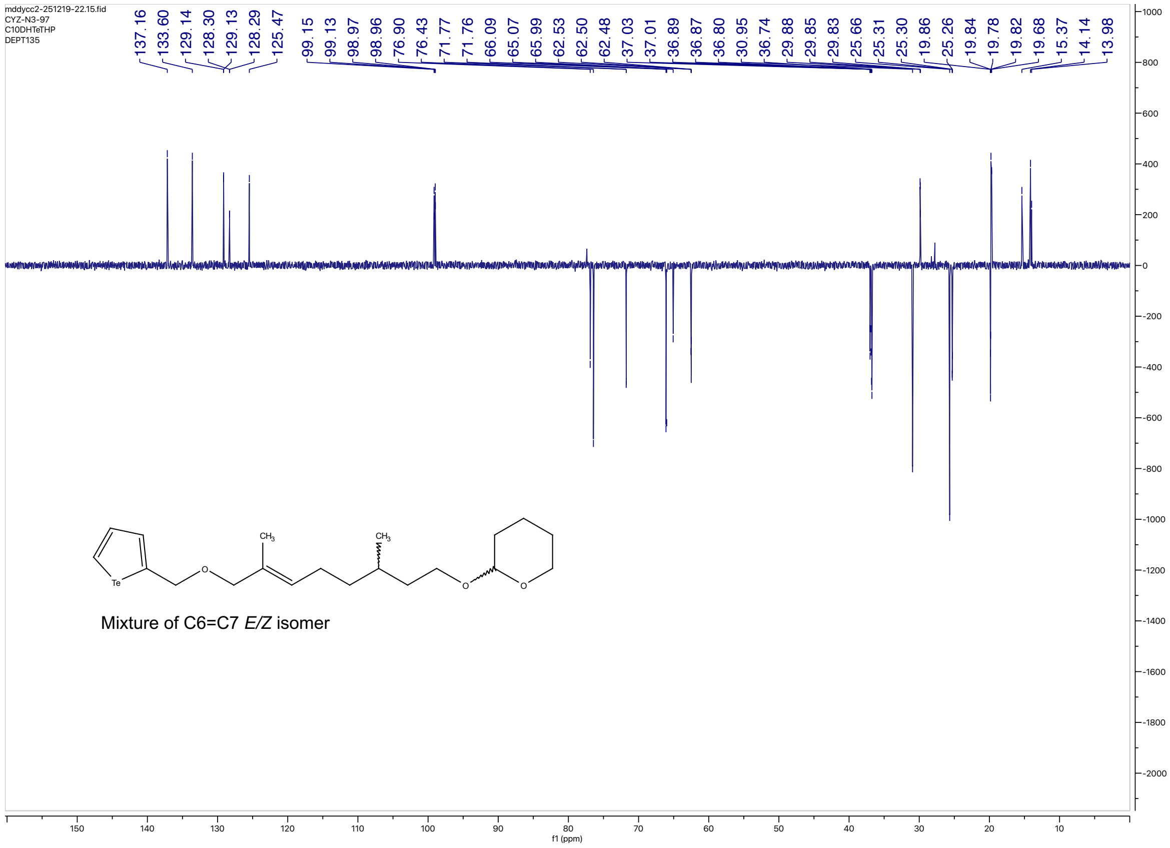

Compound **30** ^{13}C DEPT135 NMR in $CDCl_3$

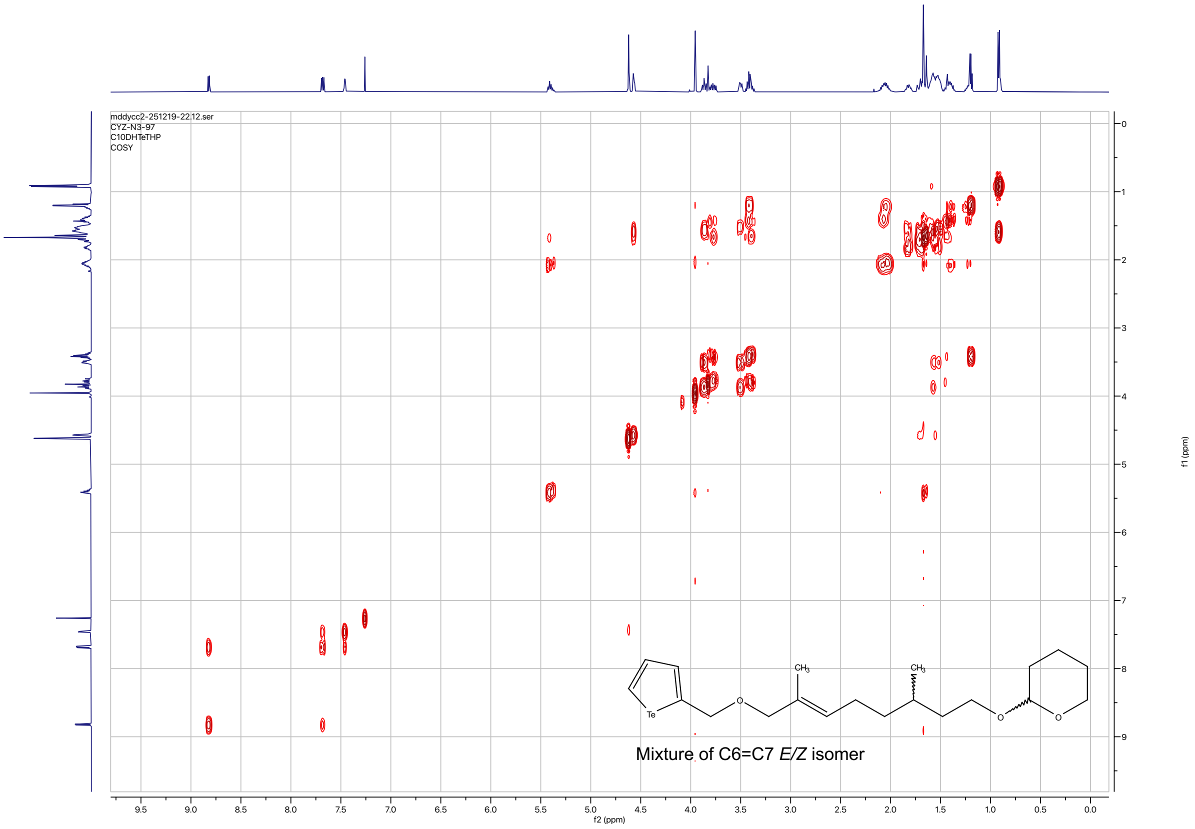

Compound **30** ^{1}H-^{1}H COSY NMR in $CDCl_3$

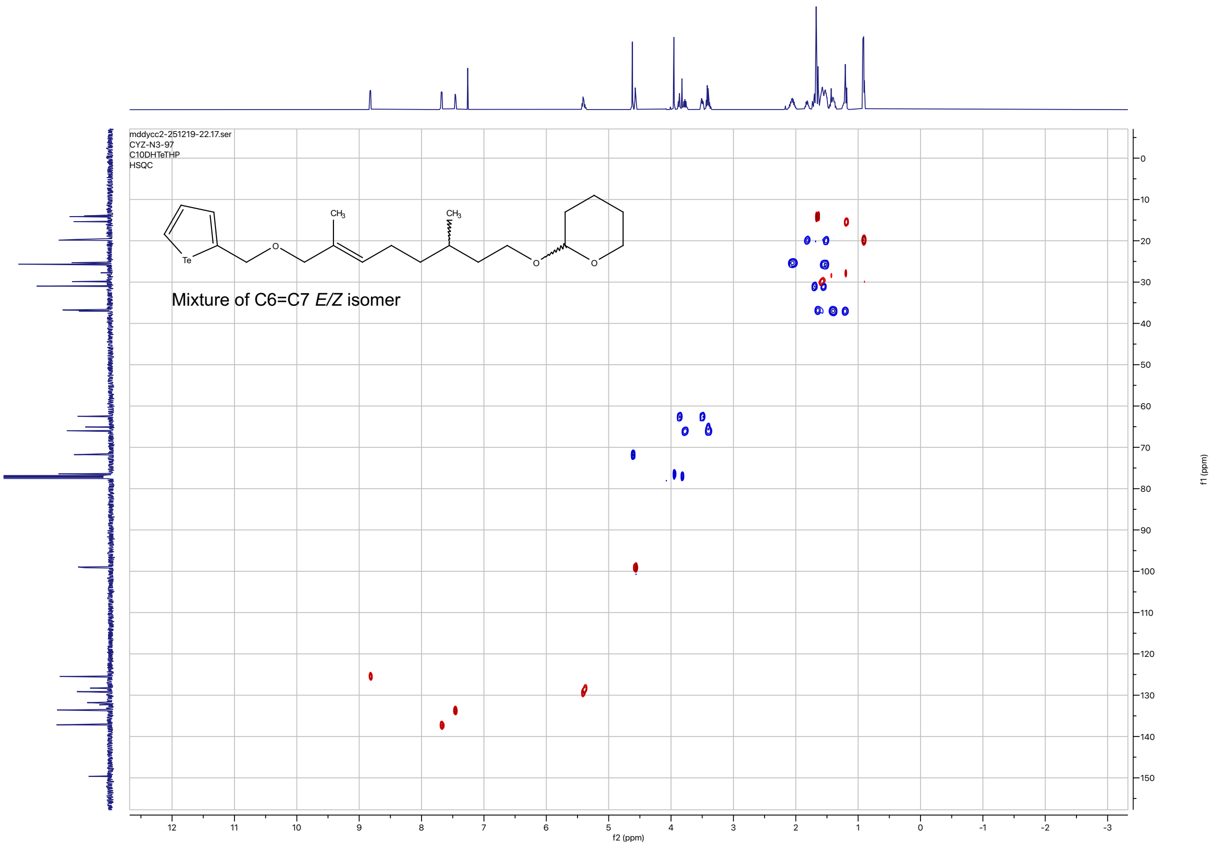

Compound **30** ^{1}H-^{13}C HSQC NMR in $CDCl_3$

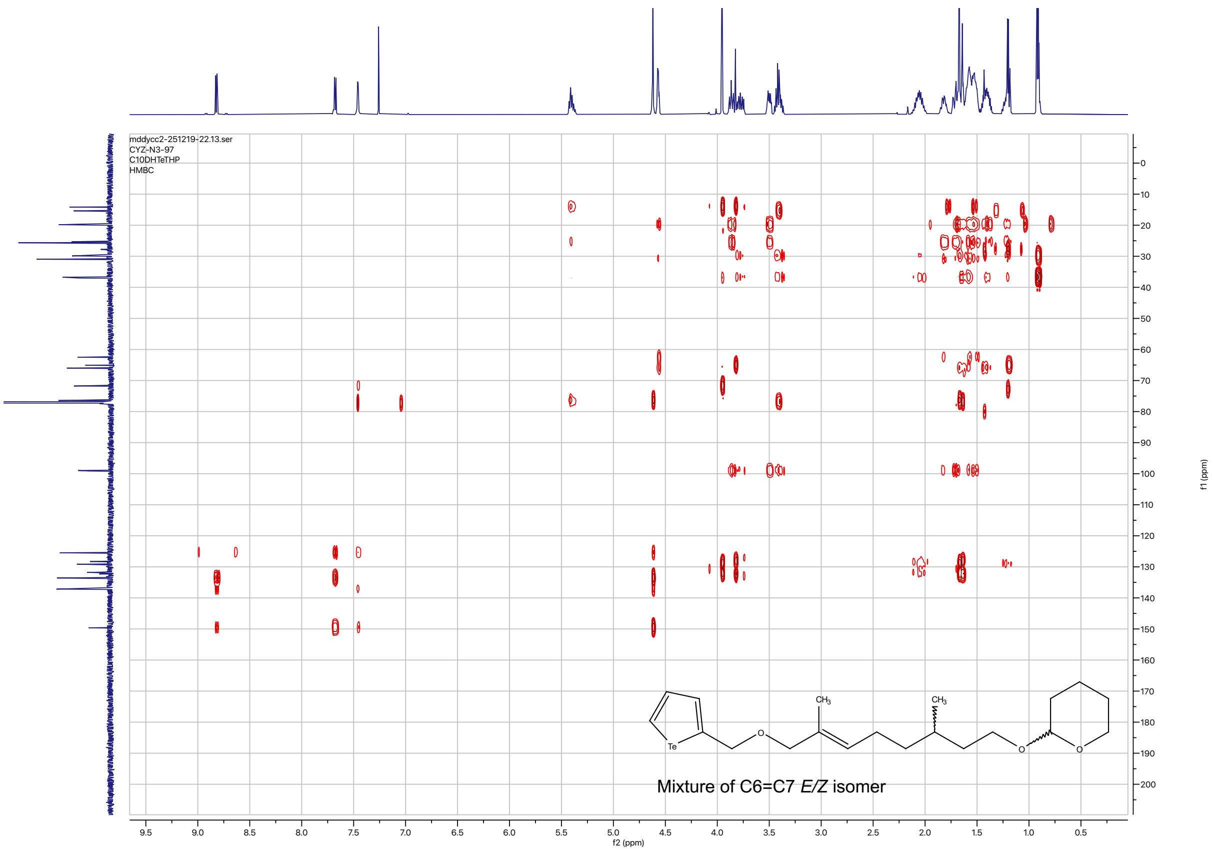

Compound **30** ^{1}H-^{13}C HMBC NMR in $CDCl_3$

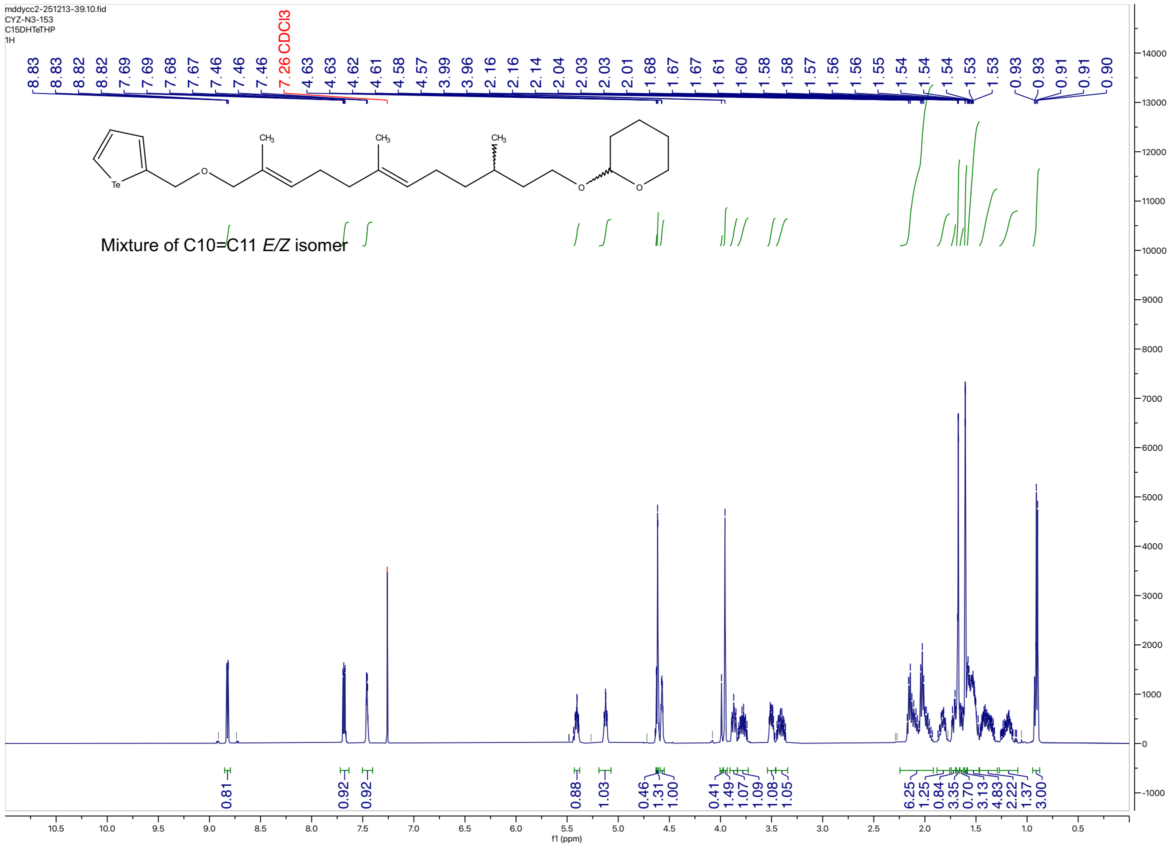

Compound **31** ^{1}H NMR in $CDCl_3$

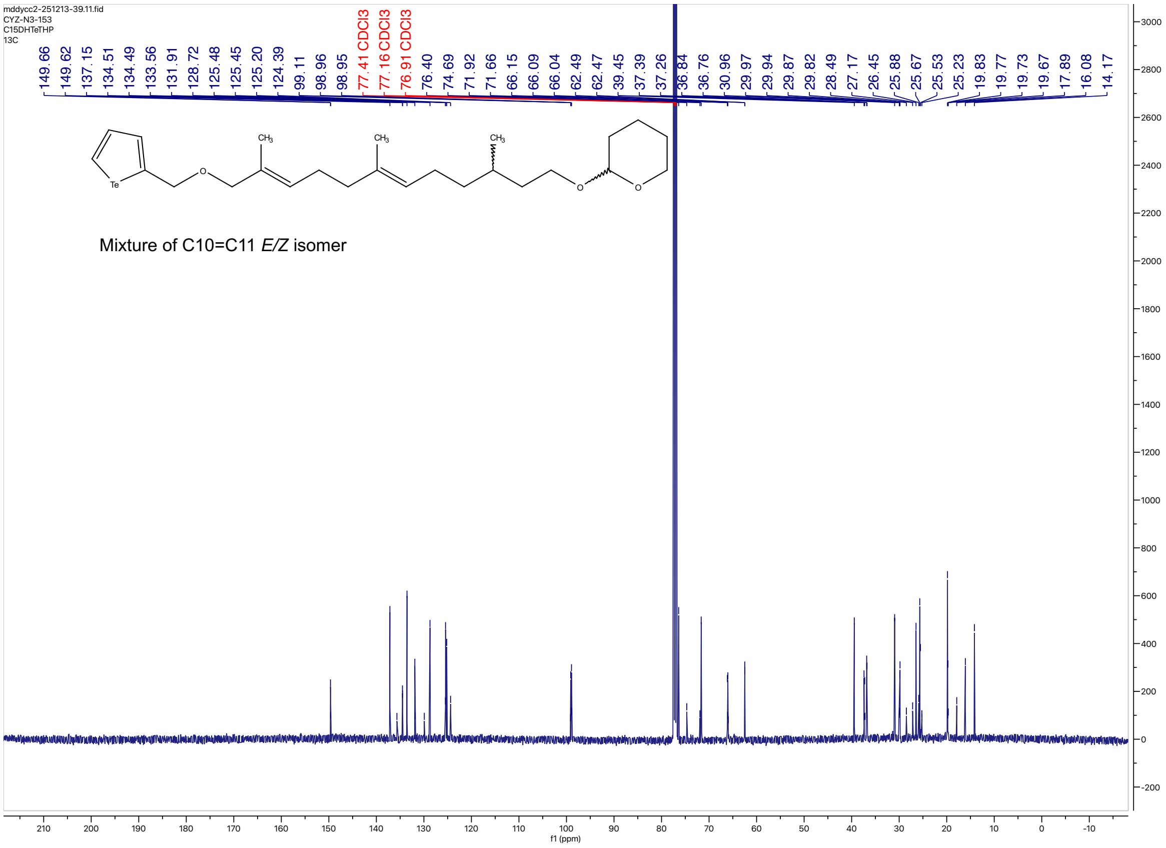

Compound **31** ^{13}C NMR in $CDCl_3$

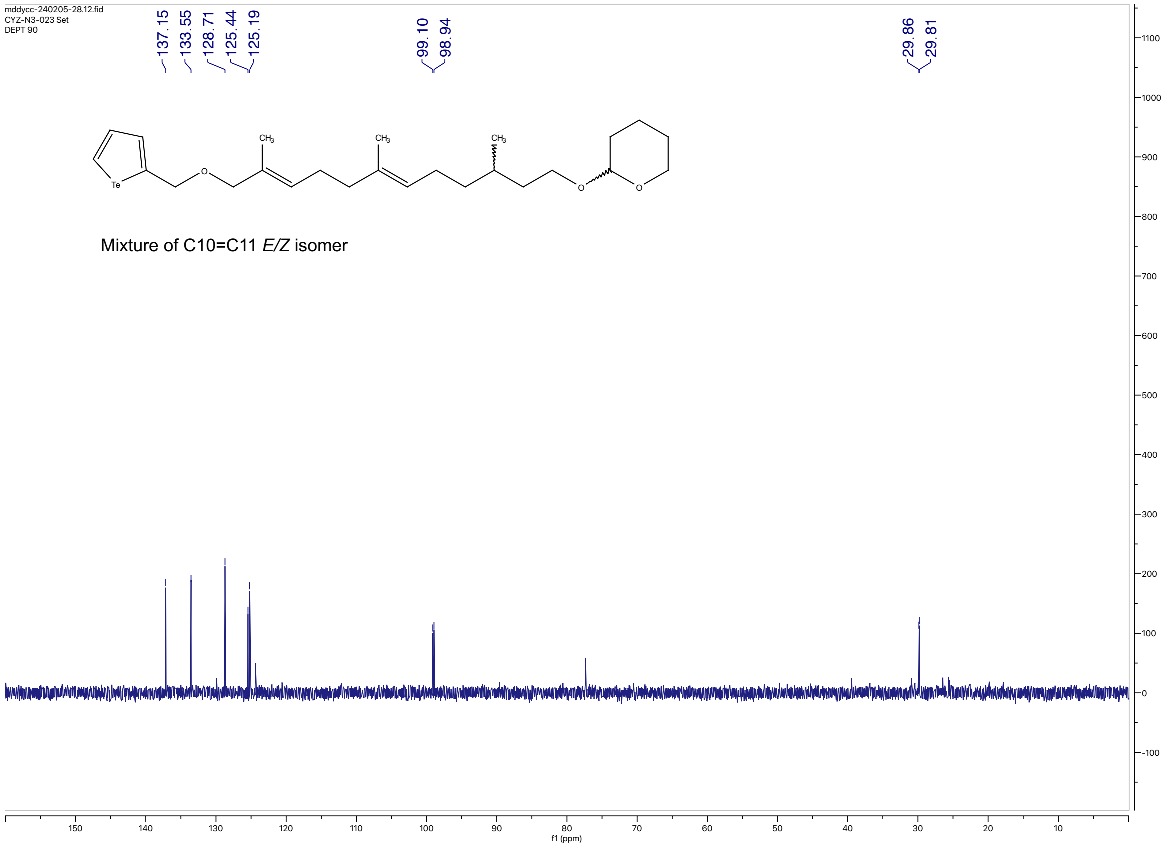

Compound **31** ^{13}C DEPT90 NMR in $CDCl_3$

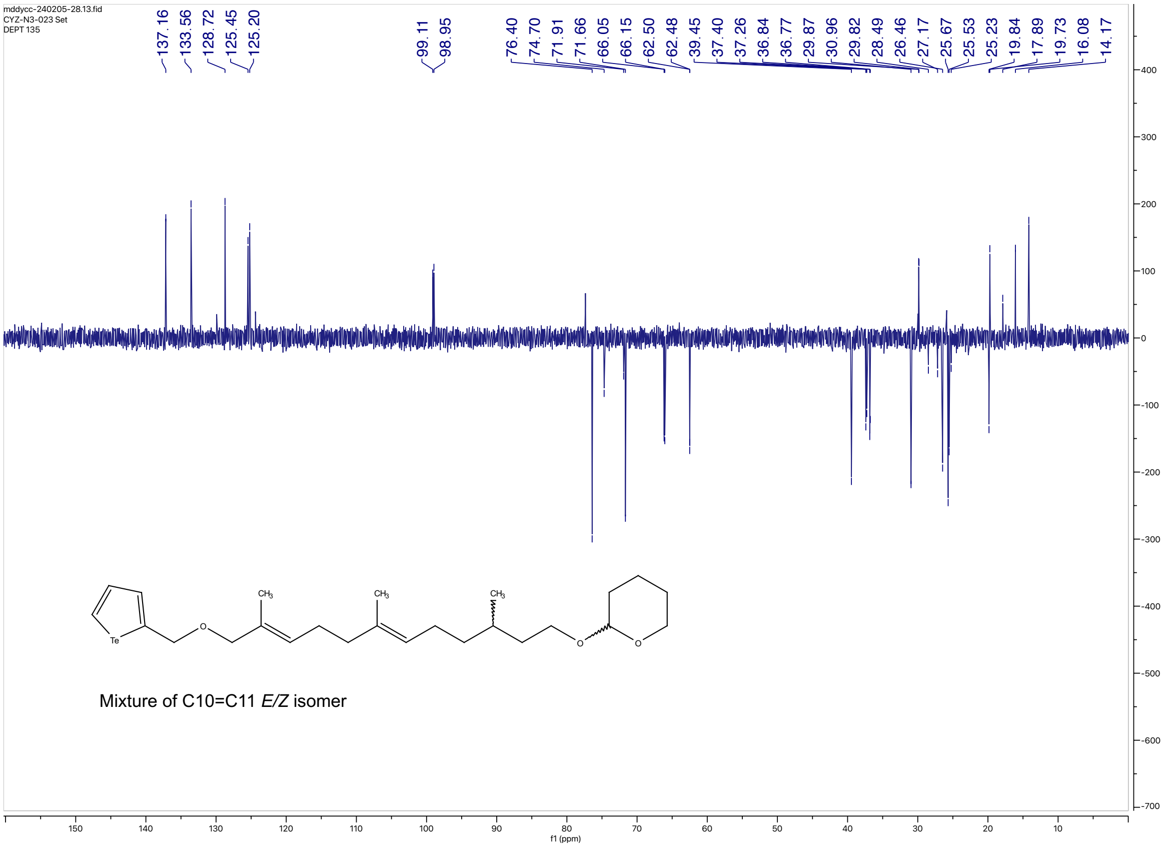

Compound **31** ^{13}C DEPT135 NMR in $CDCl_3$

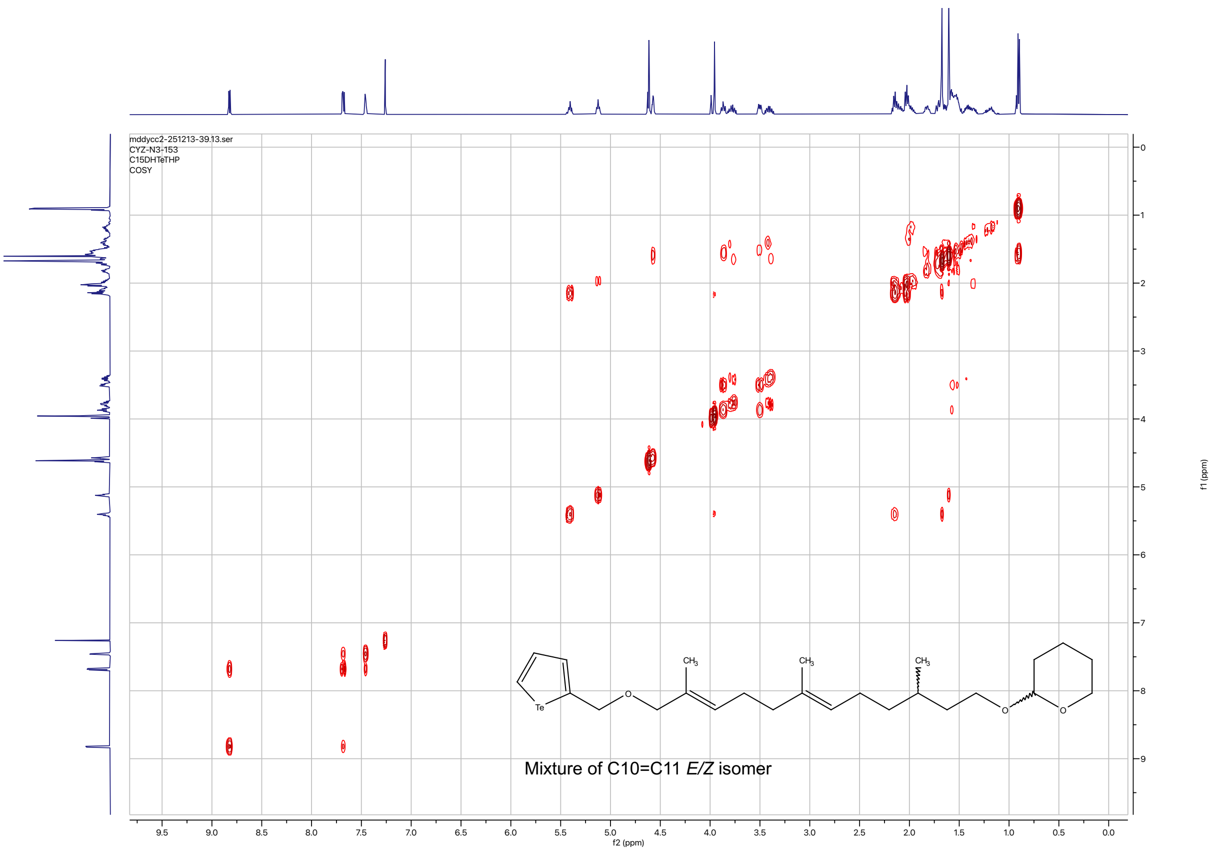

Compound **31** ^{1}H-^{1}H COSY NMR in $CDCl_3$

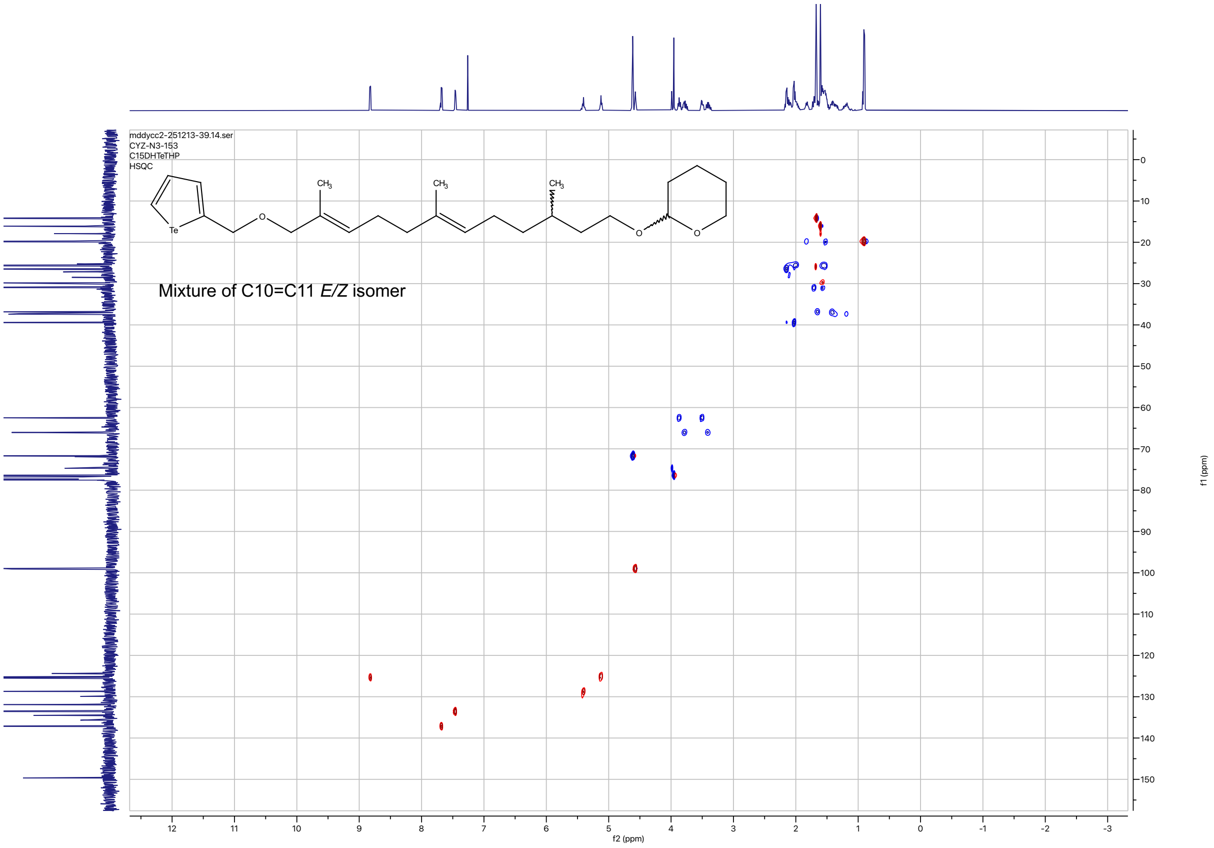

Compound **31** ^{1}H-^{13}C HSQC NMR in $CDCl_3$

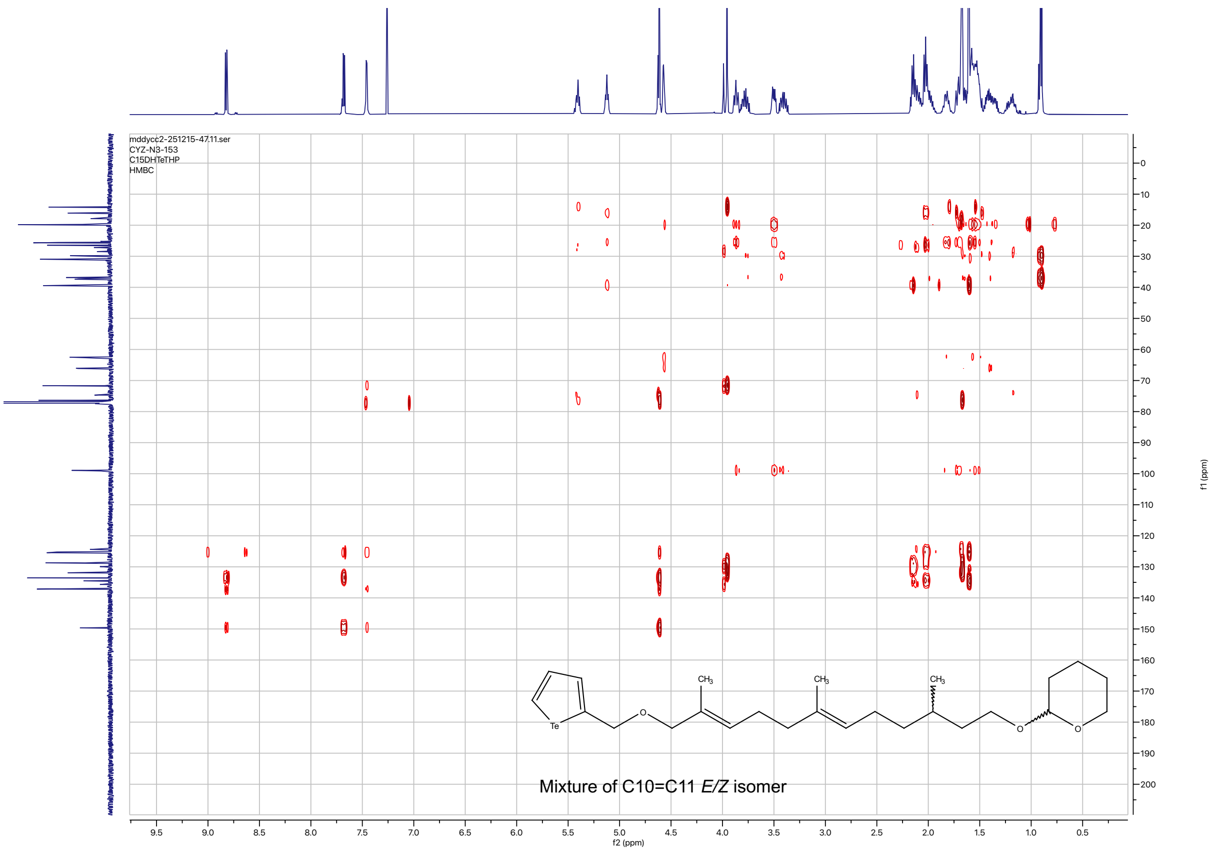

Compound **31** ^{1}H-^{13}C HMBC NMR in $CDCl_3$

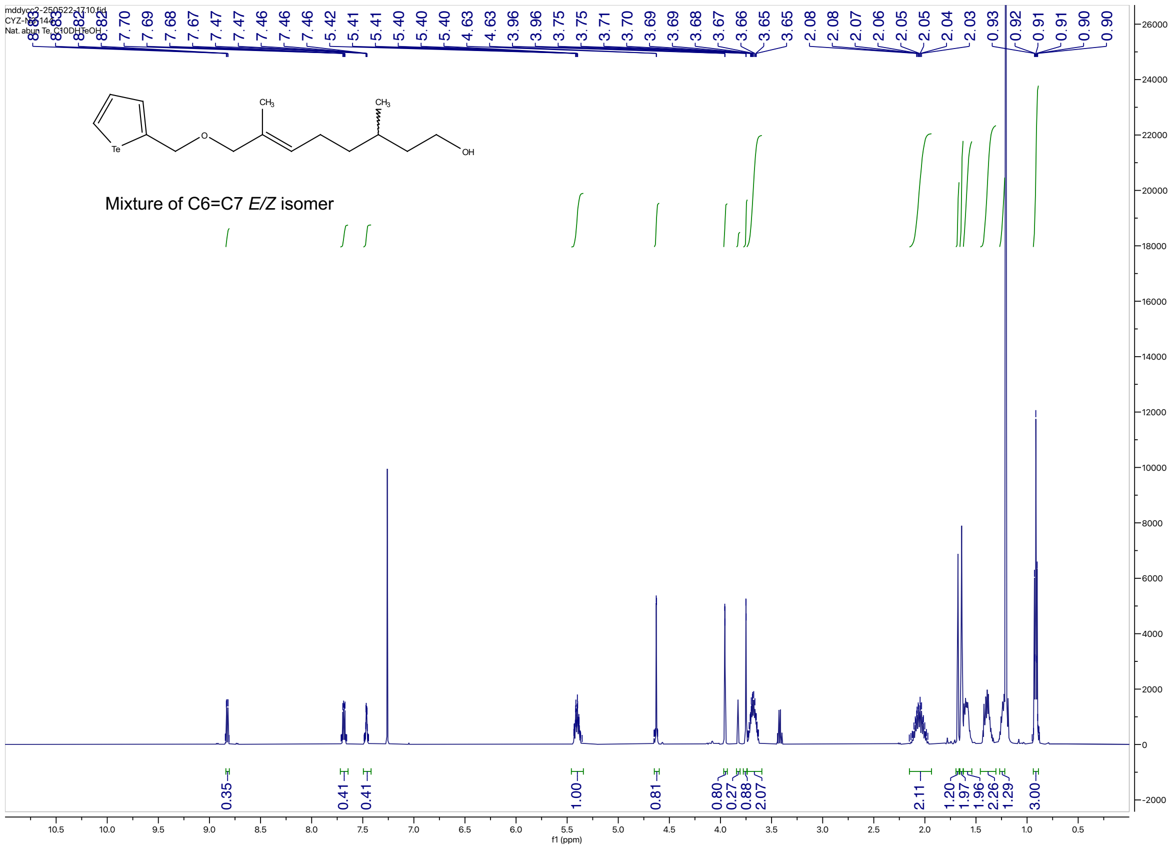

Compound **32** ^{1}H NMR in $CDCl_3$

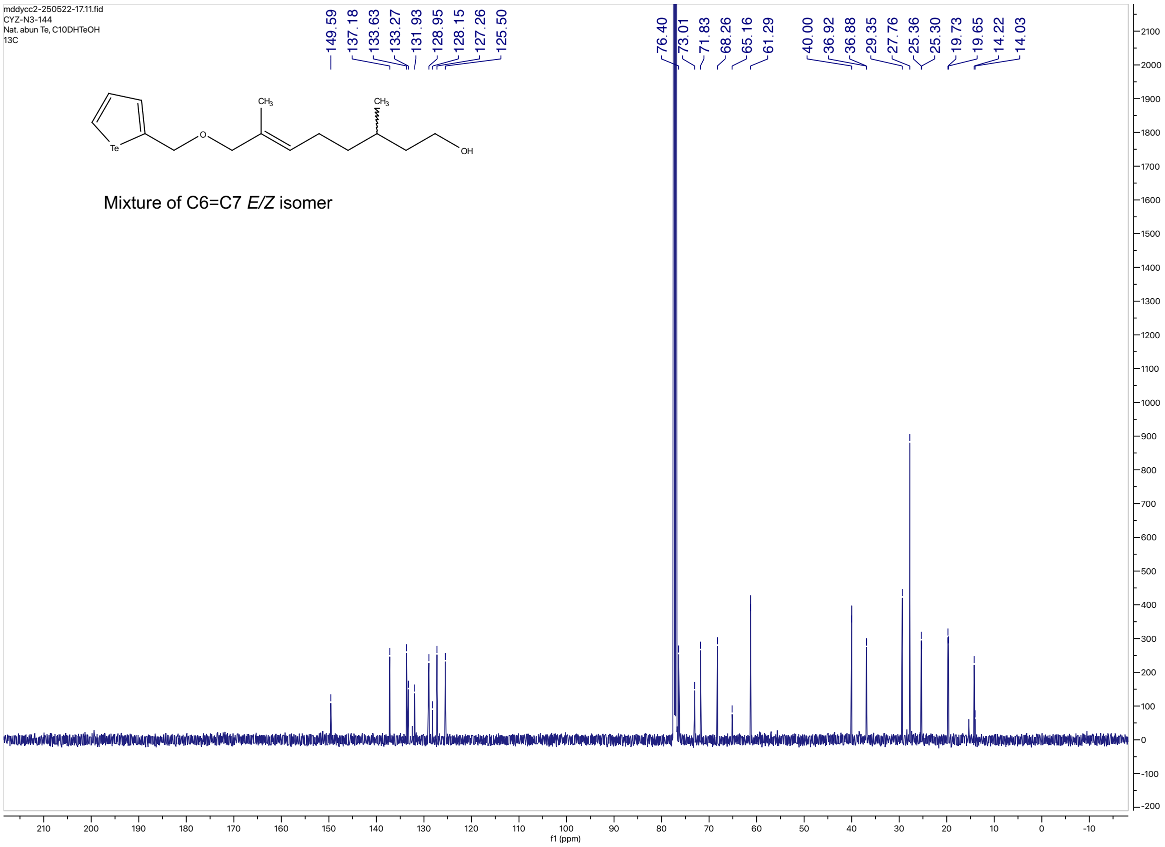

Compound **32** ^{13}C NMR in $CDCl_3$

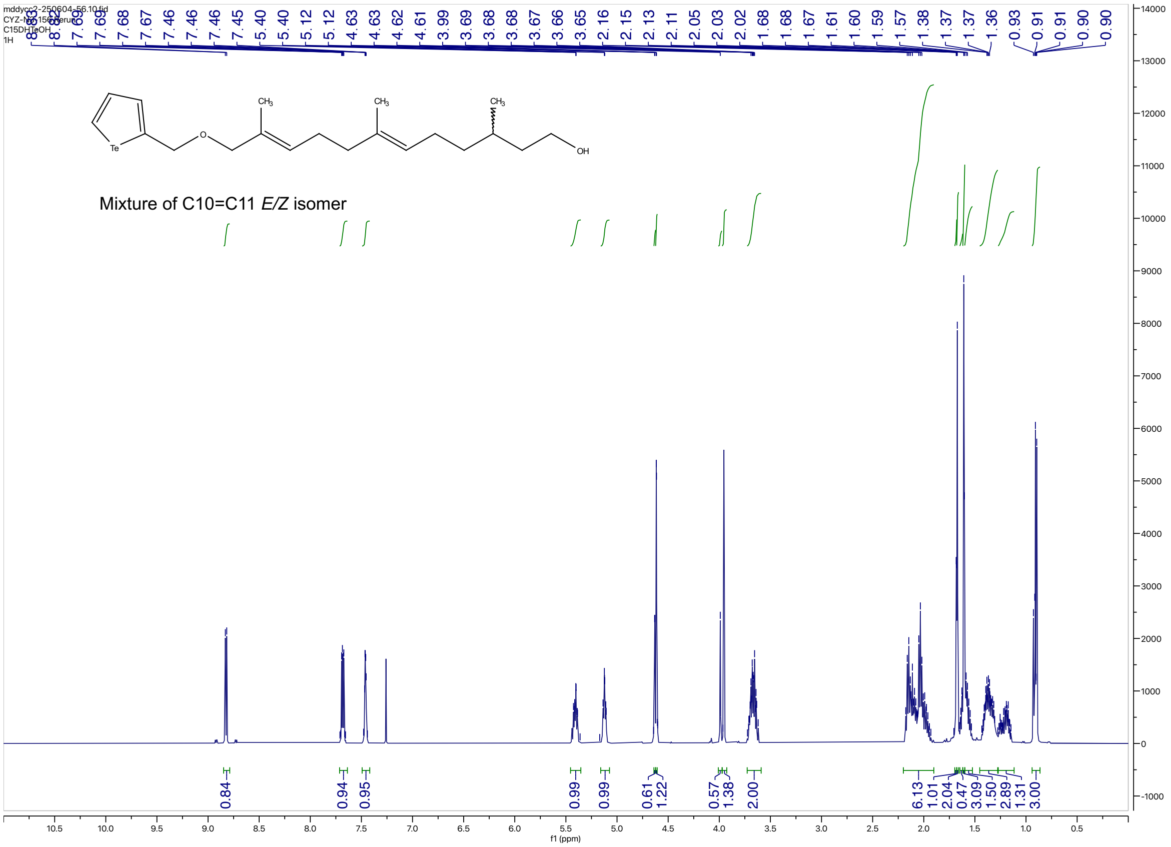

Compound **33** ^{1}H NMR in $CDCl_3$

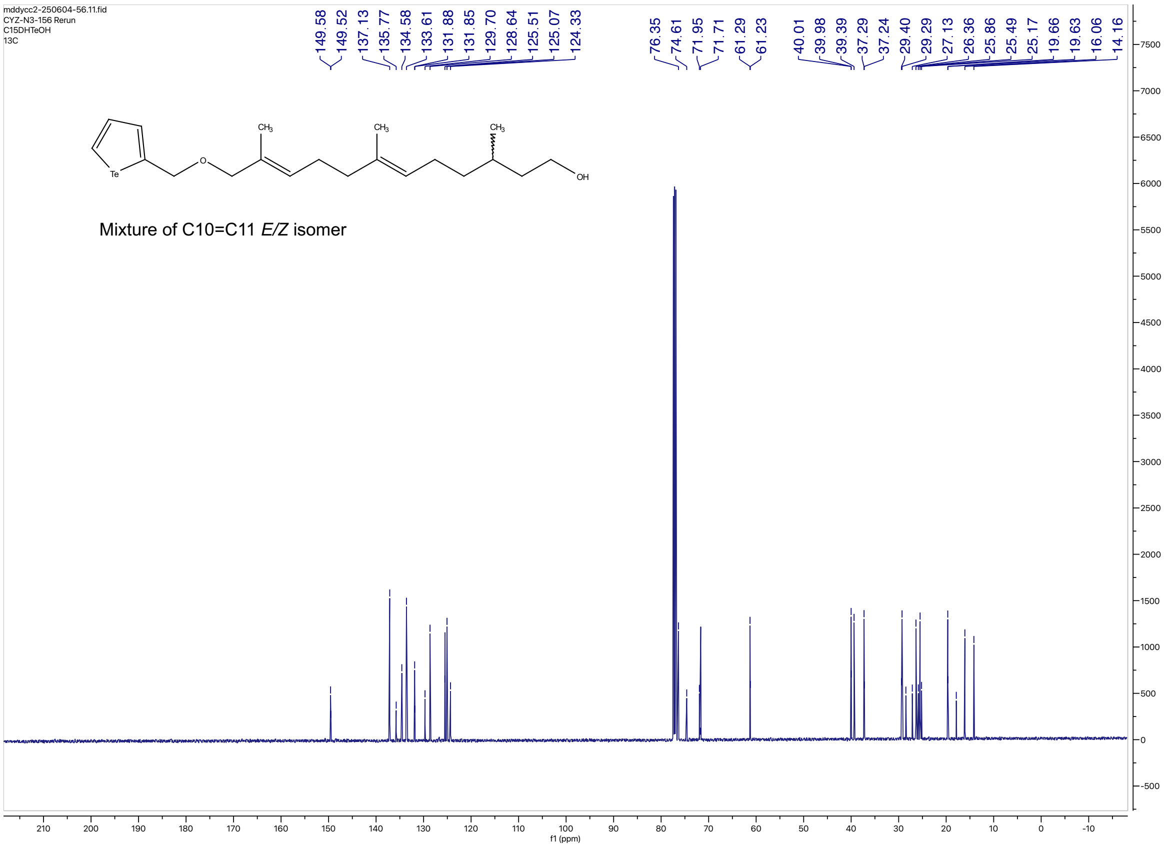

Compound **33** ^{13}C NMR in $CDCl_3$

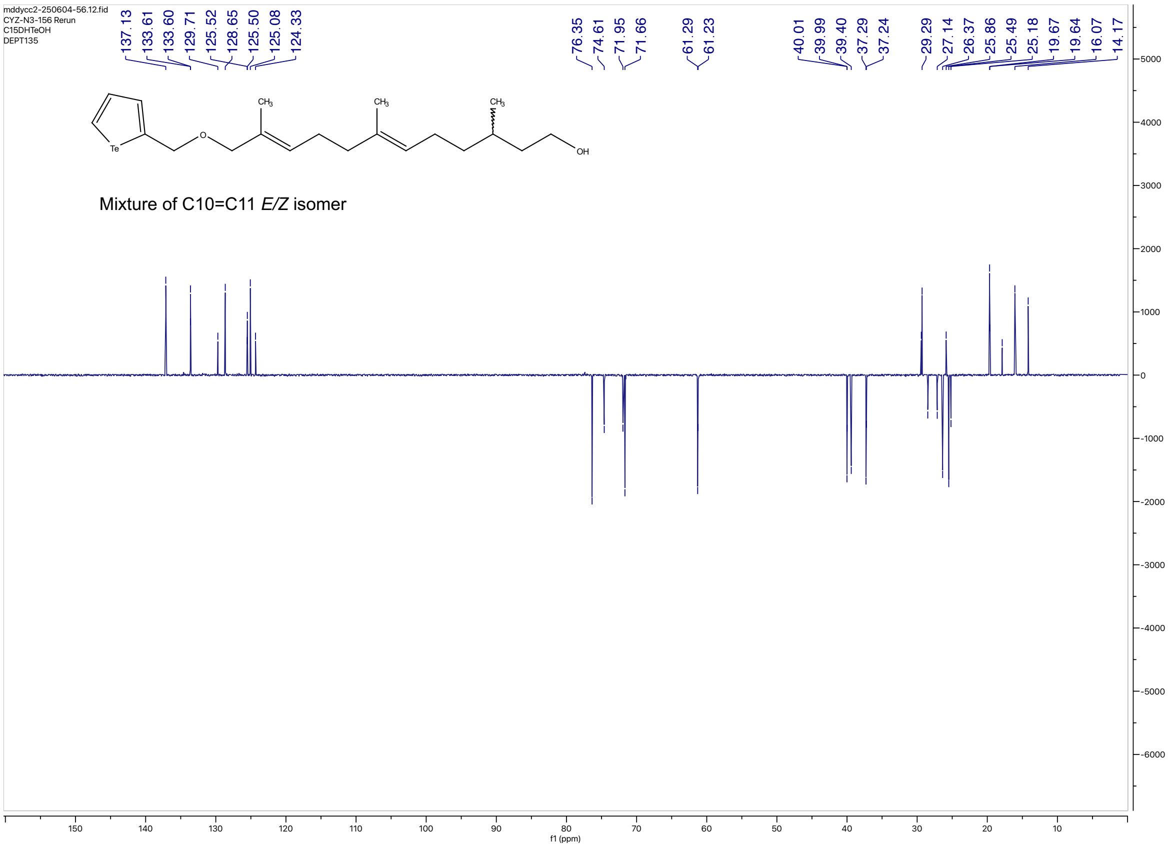

Compound **33** ^{13}C DEPT135 NMR in $CDCl_3$

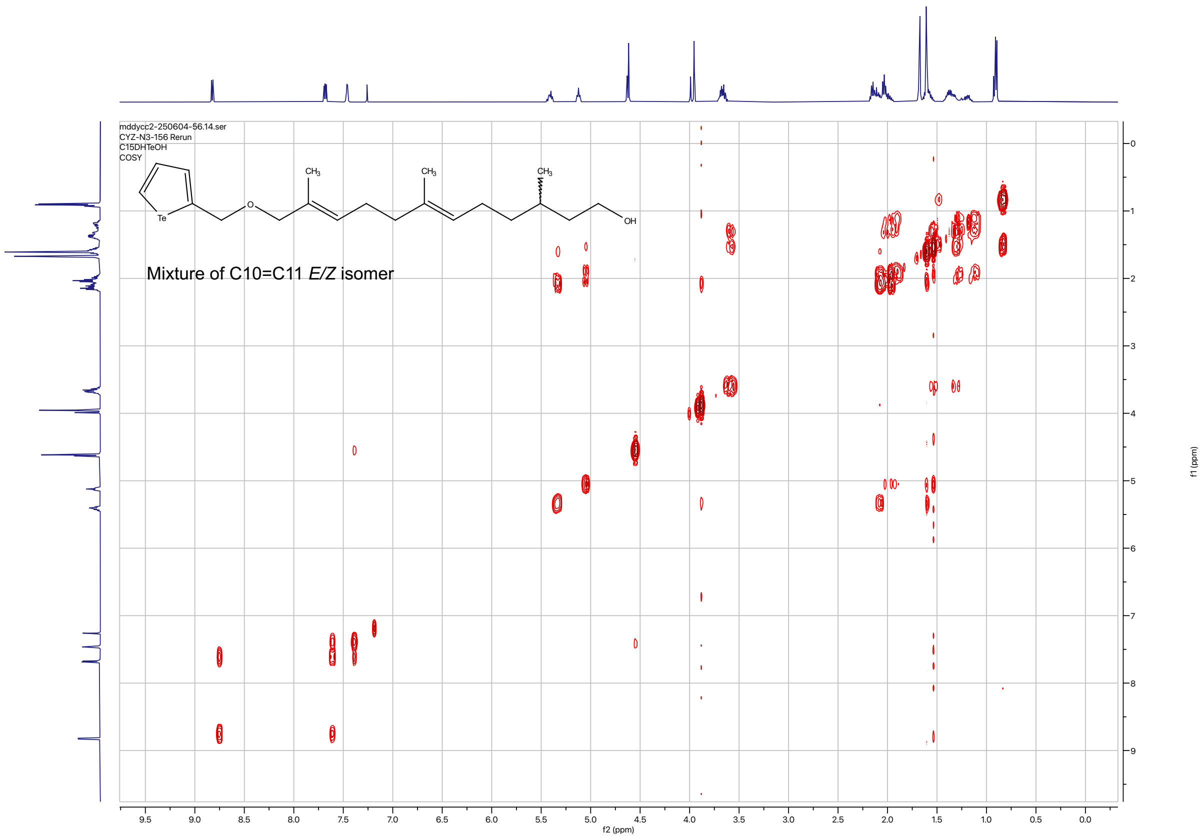

Compound **33** ^{1}H-^{1}H COSY NMR in $CDCl_3$

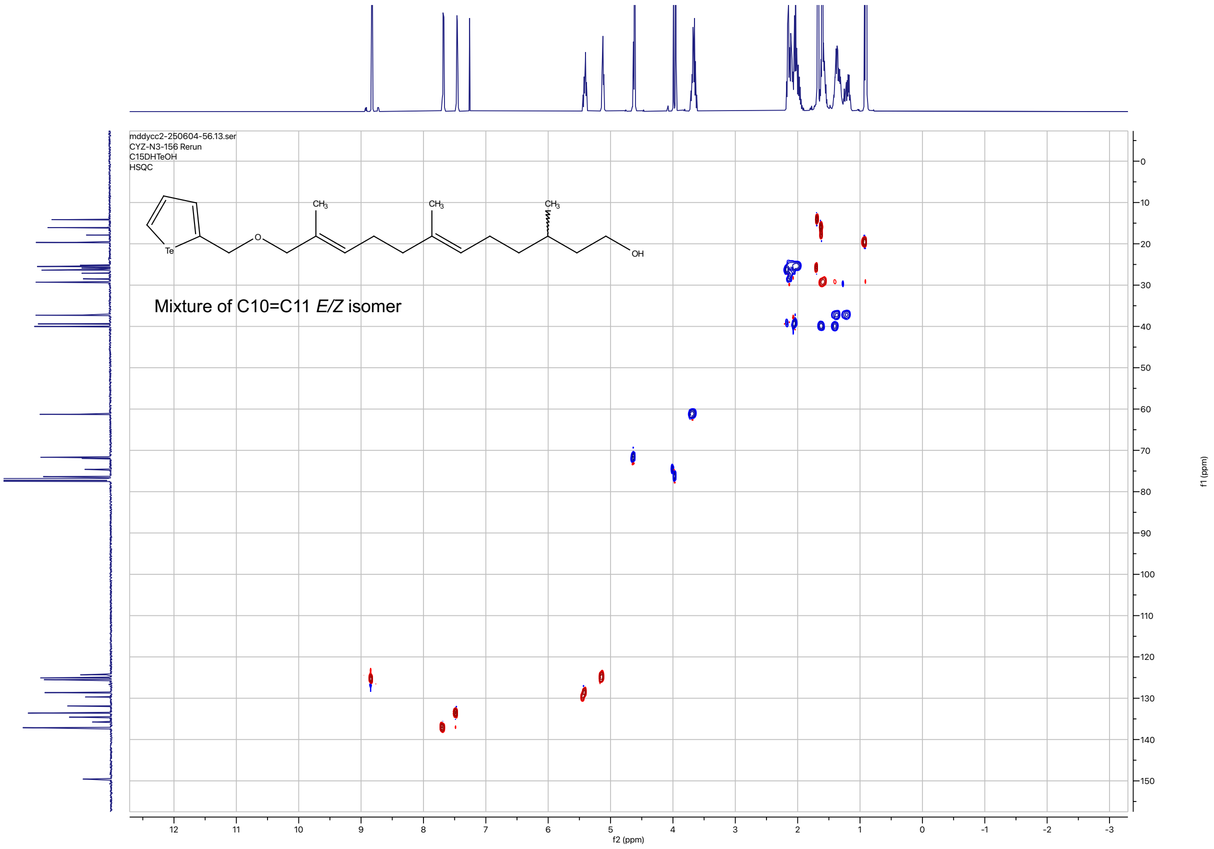

Compound **33** ^{1}H-^{13}C HSQC NMR in $CDCl_3$

References

(1) Arpicco, S.; Canevari, S.; Ceruti, M.; Galmozzi, E.; Rocco, F.; Cattel, L. Synthesis, characterization and transfection activity of new saturated and unsaturated cationic lipids. *Farmaco Sci.* **2004**, *59*, 869–878.
(2) Brown, H. M. G.; Kuhns, M. M.; Maxwell, Z.; Arriaga, E. A. Nonspecific Binding Correction for Single-Cell Mass Cytometric Analysis of Autophagy and Myoblast Differentiation. *Anal. Chem.* **2021**, *93*, 1401–1408.
(3) Blanden, M. J.; Suazo, K. F.; Hildebrandt, E. R.; Hardgrove, D. S.; Patel, M.; Saunders, W. P.; Distefano, M. D.; Schmidt, W. K.; Hougland, J. L. Efficient Farnesylation of an Extended C-Terminal C(x)3X Sequence Motif Expands the Scope of the Prenylated Proteome. *J. Biol. Chem.* **2018**, *293*, 2770–2785.
(4) Park, H.; Edgar, L. J.; Lumba, M. A.; Willis, L. M.; Nitz, M. Organotellurium scaffolds for mass cytometry reagent development. *Org. Biomol. Chem.* **2015**, *13*, 7027–7033.
(5) Rose, M. W.; Rose, N. D.; Boggs, J.; Lenevich, S.; Xu, J.; Barany, G.; Distefano, M. D. Evaluation of geranylazide and farnesylazide diphosphate for incorporation of prenylazides into a CAAX box-containing peptide using protein farnesyltransferase. *J. Pept. Res.* **2005**, *65*, 529–537.
(6) Suazo, K. F.; Mishra, V.; Maity, S.; Auger, S. A.; Justyna, K.; Petre, A. M.; Ottoboni, L.; Ongaro, J.; Corti, S. P.; Lotti, F.; et al. Improved synthesis and application of an alkyne-functionalized isoprenoid analogue to study the prenylomes of motor neurons, astrocytes and their stem cell progenitors. *Bioorg. Chem.* **2024**, *147*, 107365.
(7) Chappe, B.; Musikas, H.; Marie, D.; Ourisson, G. Synthesis of 3 Acyclic All-Trans-Tetraterpene Diols, Putative Precursors of Bacterial Lipids. *Bull. Chem. Soc. Jpn.* **1988**, *61*, 141–148.
(8) Bu, Y. J.; Tijaro-Bulla, S.; Cui, H.; Nitz, M. Oxidation-Controlled, Strain-Promoted Tellurophene-Alkyne Cycloaddition (OSTAC): A Bioorthogonal Tellurophene-Dependent Conjugation Reaction. *J. Am. Chem. Soc.* **2024**, *146*, 26161–26177.
